# Inorganometallics (Transition Metal–Metalloid Complexes) and Catalysis

**DOI:** 10.1021/acs.chemrev.1c00417

**Published:** 2021-12-30

**Authors:** Bogdan Marciniec, Cezary Pietraszuk, Piotr Pawluć, Hieronim Maciejewski

**Affiliations:** † Faculty of Chemistry, Adam Mickiewicz University, Poznań, Uniwersytetu Poznańskiego 8, 61-614 Poznań, Poland; ‡ Center for Advanced Technology, Adam Mickiewicz University, Poznań, Uniwersytetu Poznańskiego 10, 61-614 Poznań, Poland

## Abstract

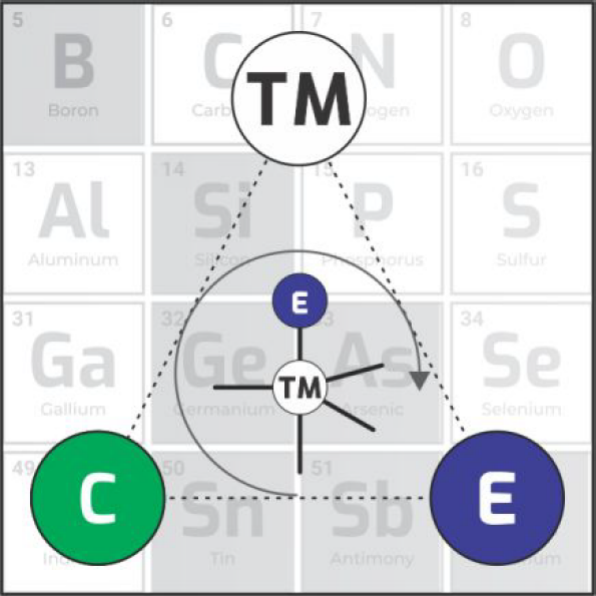

While the formation and breaking of transition metal (TM)–carbon bonds plays a pivotal role in the catalysis of organic compounds, the reactivity of inorganometallic species, that is, those involving the transition metal (TM)–metalloid (E) bond, is of key importance in most conversions of metalloid derivatives catalyzed by TM complexes. This Review presents the background of inorganometallic catalysis and its development over the last 15 years. The results of mechanistic studies presented in the Review are related to the occurrence of TM–E and TM–H compounds as reactive intermediates in the catalytic transformations of selected metalloids (E = B, Si, Ge, Sn, As, Sb, or Te). The Review illustrates the significance of inorganometallics in catalysis of the following processes: addition of metalloid–hydrogen and metalloid–metalloid bonds to unsaturated compounds; activation and functionalization of C–H bonds and C–X bonds with hydrometalloids and bismetalloids; activation and functionalization of C–H bonds with vinylmetalloids, metalloid halides, and sulfonates; and dehydrocoupling of hydrometalloids. This first Review on inorganometallic catalysis sums up the developments in the catalytic methods for the synthesis of organometalloid compounds and their applications in advanced organic synthesis as a part of tandem reactions.

## Introduction: Inorganometallic versus Organometallic Chemistry and Catalysis: General Guidelines

According to the commonly accepted definition, organometallics are compounds that contain at least one chemical bond between a carbon atom of an organic group or molecule and a metal atom or atoms (a main group element, a transition metal (TM) or a lanthanide or actinide). The importance of organometallics in catalysis results from the reactivity of TM–C intermediates, which are active in the crucial steps of important organic synthesis processes (e.g., olefin oxidation, hydroformylation, carbonylation, hydrogenation, olefin metathesis) and the preparation of polymers (Ziegler–Natta polymerization, oligomerization of olefins).

Elements such as boron, silicon, germanium, tin, arsenic, antimony, and tellurium, known in the literature as metalloids, can form chemical bonds with carbon, and the resulting compounds are also classified as organometallic compounds. However, if metalloids form at least one chemical bond with a metal atom or atoms, the resulting species may be called inorganometallics. Such compounds are investigated in a new field of science called inorganometallic chemistry.^1^ Therefore, although distinctly different from organometallics, the compounds that contain TM–metalloid bonds also act as active intermediates, particularly in the transformations of *p*-block compounds, such as those with silicon, boron, germanium, tin, arsenic, antimony, and tellurium.

**Scheme 1:**
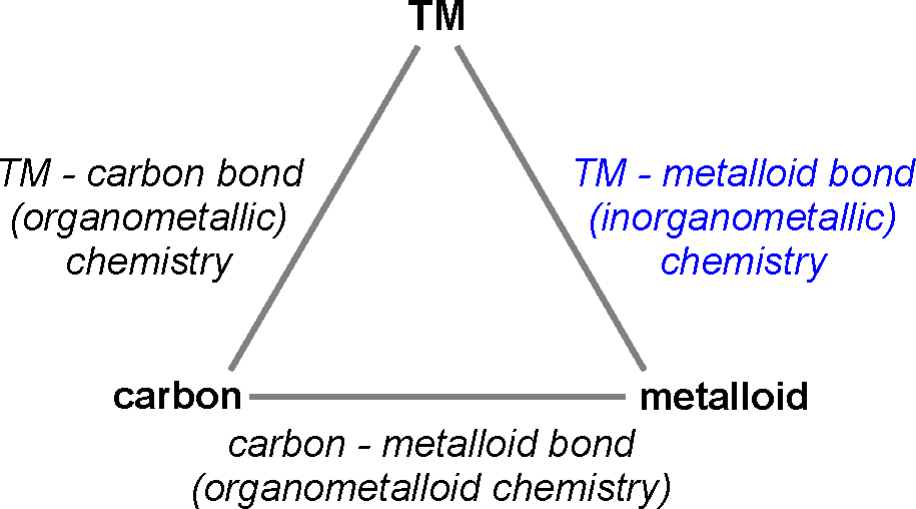
Concept of Inorganometallic Chemistry and its Relationship with Organometallic and Organometalloid Chemistry

Advances in organometallic chemistry, including the results of intensive research on the organometallic chemistry of boron, silicon, and other metalloid compounds, have clearly shown that the chemical properties of compounds containing a metal–metalloid bond, particularly when the metal is a TM, significantly differ from those having a metal–heteroatom (O, S, N, P) bond. The interrelation between organometallic, organometalloid, and inorganometallic chemistry is illustrated by the triangular Scheme 1.^2,3^

According to a number of findings, the reactivity of inorganometallic species, particularly those having a TM–metalloid bond, is of key importance in most conversions of metalloid derivatives catalyzed by TM complexes.

Our review “*Inorganometallic Chemistry*”, published in 2007, focused on the synthesis, structure, and reactivity of inorganometallic compounds containing a bond between a TM and elements of main groups 13–16 (excluding C, N, P, O, and S).^2^ The review discussed the key role of TM–metalloid bonds (inorganometallics) in catalytic transformations as well as their use in materials chemistry.

The purpose of the current Review is to discuss the scientific literature published since 2006 on the catalytic transformations of compounds of selected metalloids (B, Si, Ge, Sn, As, Sb, Te) catalyzed by TM complexes and to present and systematize the most important achievements of inorganometallic catalysis. By way of exception, we will consider tin as well because its electronegativity and first ionization potential values meet the criteria of metalloids and the reactivity of tin compounds in catalytic processes is similar to that of germanium and silicon derivatives. We intend to show the great potential of such inorganometallic compounds in chemical synthesis, thereby drawing special attention to reaction mechanisms and illustrating the role of inorganometallics in catalysis.

The following processes are discussed in this Review:

1. addition of metalloid–hydrogen bonds to unsaturated compounds
2. addition of metalloid–metalloid bonds to unsaturated compounds
3. activation (and functionalization) of C–H bonds with hydrometalloids and bismetalloids
4. activation (and functionalization) of C–X bonds with hydrometalloids and bismetalloids
5. dehydrocoupling of hydrometalloids
6. activation (and functionalization) of C–H bonds with vinylmetalloids
7. activation (and functionalization) of C–H bonds with metalloid halides and sulfonates
8. applications in organic synthesis

Most of the above processes that involve the reactivity of organometalloids in addition and coupling reactions in the presence of TM complexes have been addressed in several reviews and books that will be cited in the discussion of individual reactions. Most of them are written in a similar way and focus mostly on catalyst reactivity and efficiency of synthesis, while the role of inorganometallic compounds as key intermediates has not been given the attention it deserves. In this Review, we address the background of inorganometallic catalysis and present its development over the past 15 years. We also discuss our personal perspectives on the future trends and directions that could make inorganometallic catalysis the new benchmark in homogeneous catalysis. Consequently, the aim of this work is to present an overview of the main achievements in a variety of the above-mentioned reactions of *p*-block element compounds catalyzed by TM complexes leading to organometalloid products. In their transformations, inorganometallics are actually catalysts, but they simultaneously yield products that contain carbon–metalloid and metalloid–metalloid bonds.

Finally, it is noted that the above-mentioned processes may be incorporated in the tandem (sequential) reactions that play an important role in advanced organic synthesis.

## Transformations of Metalloid Compounds Catalyzed by TM Complexes: Mechanistic Aspects of Catalysis

The aim of this paper is to present an overview of the main achievements in a variety of reactions of metalloid compounds of groups 13, 14, 15, and 16 catalyzed by TM complexes. In these transformations, TM–metaloid complexes (inorganometallics) are the actual catalysts, and the E derivatives do not act as ancillary ligands but undergo transformations leading to products containing carbon–metalloid or metalloid–metalloid bonds.

TM-catalyzed carbon–carbon bond formation developed in the 1970s was a milestone in synthetic organic chemistry, and this type of catalysis has been successfully applied in the selective syntheses of a variety of molecular and macromolecular organometallic compounds.^4−9^ However, TM complexes have been recognized as very efficient catalysts in the transformations in which the molecular and macromolecular compounds that contain carbon–metalloid or metalloid–metalloid bonds bonds can be synthesized. The results of mechanistic studies published since 2006 indicate that such processes occur in the presence of catalytic species that contain initially or in situ generated TM–H and TM–E bonds. Therefore, in this paper, we discuss the important role of inorganometallics (transition metal–metalloid complexes) as reactive intermediates in the catalytic transformations.

## 1 Addition of Metalloid–Hydrogen Bonds to Unsaturated Compounds

The most widely used methods for forming E–C bonds in the presence of a TM include the addition reactions of hydrides of group 14 elements to carbon–carbon double and triple bonds (hydrosilylation, hydrostannylation, and hydrogermylation) and of group 13 (hydroboration). The knowledge on hydrometalation with group 14 and group 13 elements was discussed in the first edition of *Comprehensive Organic Synthesis II* in 2014.^10,11^

**Scheme 2:**
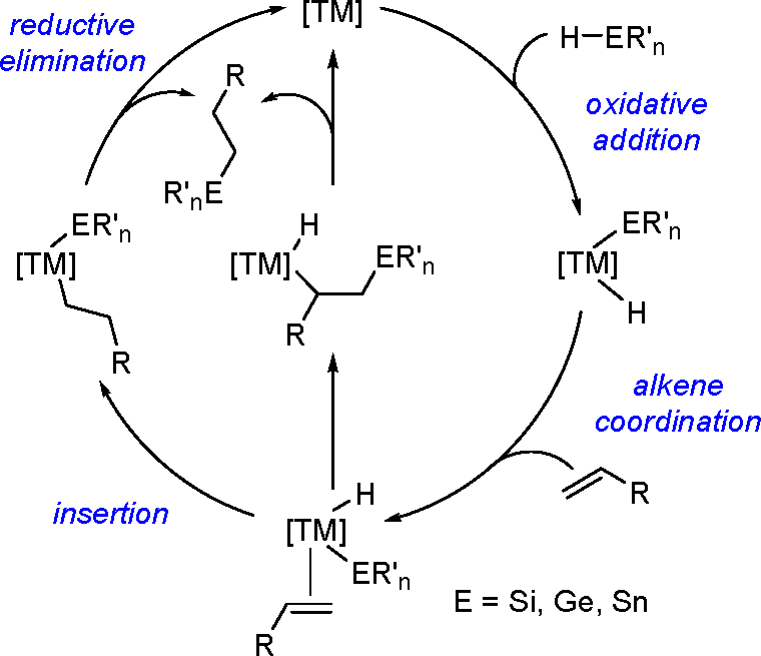
General Mechanism of the Hydrometallation (E = Si, Ge, Sn) of Alkenes by TM Complexes

The general mechanism presented in Scheme 2 for the addition of R_n_E–H (where E is Si, Ge, or Sn) to alkenes catalyzed by late TM complexes, provided by Chalk and Harrod in 1965, is based on hydrosilylation^12^ catalyzed by Pt complexes.

Intermediates with metal–silicon bonds (i.e., silicometallics) or other inorganometallics versus organometallics play a decisive role in the mechanisms of all catalytic reactions of substrates that contain silicon (or other metalloids).^2,13^

The mechanism includes a conventional oxidative addition of Si–H (Ge–H, Sn–H) to a metal–alkene complex configuration (usually d^8^–d^10^), followed by alkene insertion into the TM–H bond. The resulting metal–inorganometallic alkyl complex undergoes reductive elimination by E–C bond formation and regeneration of the TM catalyst. However, a modified version of the mechanism has also been proposed, which involves alkene insertion into the metal–metalloid bond followed by reductive elimination.^14^

TM complexes with a silyl (or germyl or stannyl) ligand as an anionic X-type ligand have been extensively studied as important intermediates in various catalytic silylation (germylation, stannylation) reactions, such as hydrosilylation and C–H silylation.^15−18^

### 1.1 Hydrosilylation of Unsaturated Bonds

Hydrosilylation (also called hydrosilation) is the most significant hydrometalation in group 14. This process of industrial importance is used primarily to synthesize organofunctional silicon monomers and cross-linked silicone polymers and to prepare other molecules and macromolecules, such as saturated and unsaturated organosilicon compounds and inorganic materials. Most industrial hydrosilylation processes occur in the presence of H_2_PtCl_6_, used as the initial precursor (mostly Speier’s catalyst and Karstedt’s complex catalyst, [Pt_2_{(CH_2_=CHMe_2_Si)_2_O}_3_]).

Hydrosilylation is a well-documented process in silicon chemistry discussed in detail (including the catalyst selection) in several excellent reviews and books.^17−32^ In our review of 2009,^18^ we addressed all the mechanistic aspects of alkene and alkyne hydrosilylation catalyzed by nickel and iron and cobalt group complexes, which follows the anti-Markovnikov rule but under some conditions yields α-adducts or dehydrogenative silylation products that are also attractive for synthesis.^13^ In this Review, we discuss only the new mechanistic aspects of TM-catalyzed hydrosilylation published in the past 12 years.

#### 1.1.1 Homogeneous Catalysis of Alkene and Alkyne Hydrosilylation

**Scheme 3:**
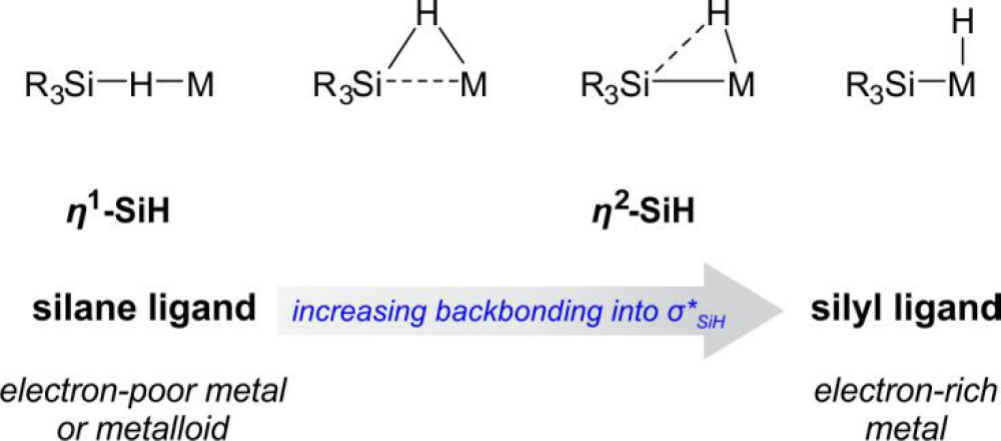
Hydrosilane Binding Mode as a Function of Electron Density of the Metal Site

TM organosilicon complexes have recently drawn much attention, particularly in view of catalysis and the differences between metal–silicon and metal–carbon bonds. Some reviews^15,19,30^ have shown the special role of silicon in catalytic processes. Silicon is less electronegative than carbon, and many of the mid-to-late TMs act mostly as electrophiles and Lewis acids. Owing to this low electronegativity and a relatively large size, silicon develops a variety of bonds exhibiting secondary interactions, for example, with hydrides. Polarization of the Si–H unit in hydrosilanes combined with the steric effect of H makes the Si–H unit a good electron donor for TM complexes. The electron-richness of a metal formally dictates a variety of structures. For an electron-poor acceptor, the interaction with a hydrosilane occurs via η^1^-coordination (see Scheme 3).^30^

**Scheme 4:**
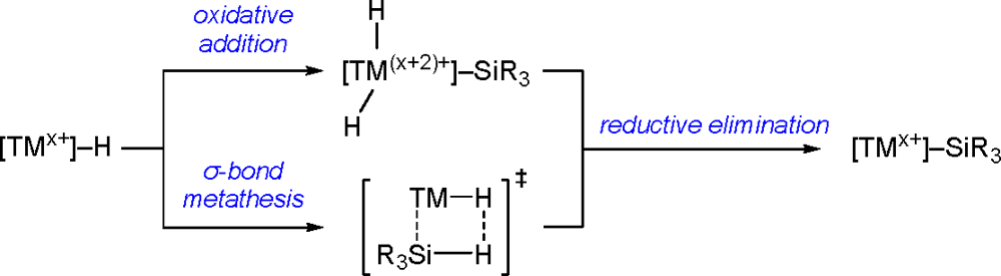
Simplified Comparison of the Oxidative Addition Reaction Pathway and σ-Bond Metathesis Pathway

Most TM compounds with hydrosilanes include some M–Si interactions (π-back bonding into σ*SiH) whose ultimate role is to bring about Si–H oxidative addition. However, for an oxidative addition process to occur, a coordinatively unsaturated metal precursor is required, which can be obtained via a reductive elimination process (H_2_ or HX). When oxidative addition is not favored (especially for early TMs), another reaction pathway can be used: σ-bond metathesis may provide a low-energy step to a TM–silyl complex (Scheme 4).^15^

**Scheme 5:**
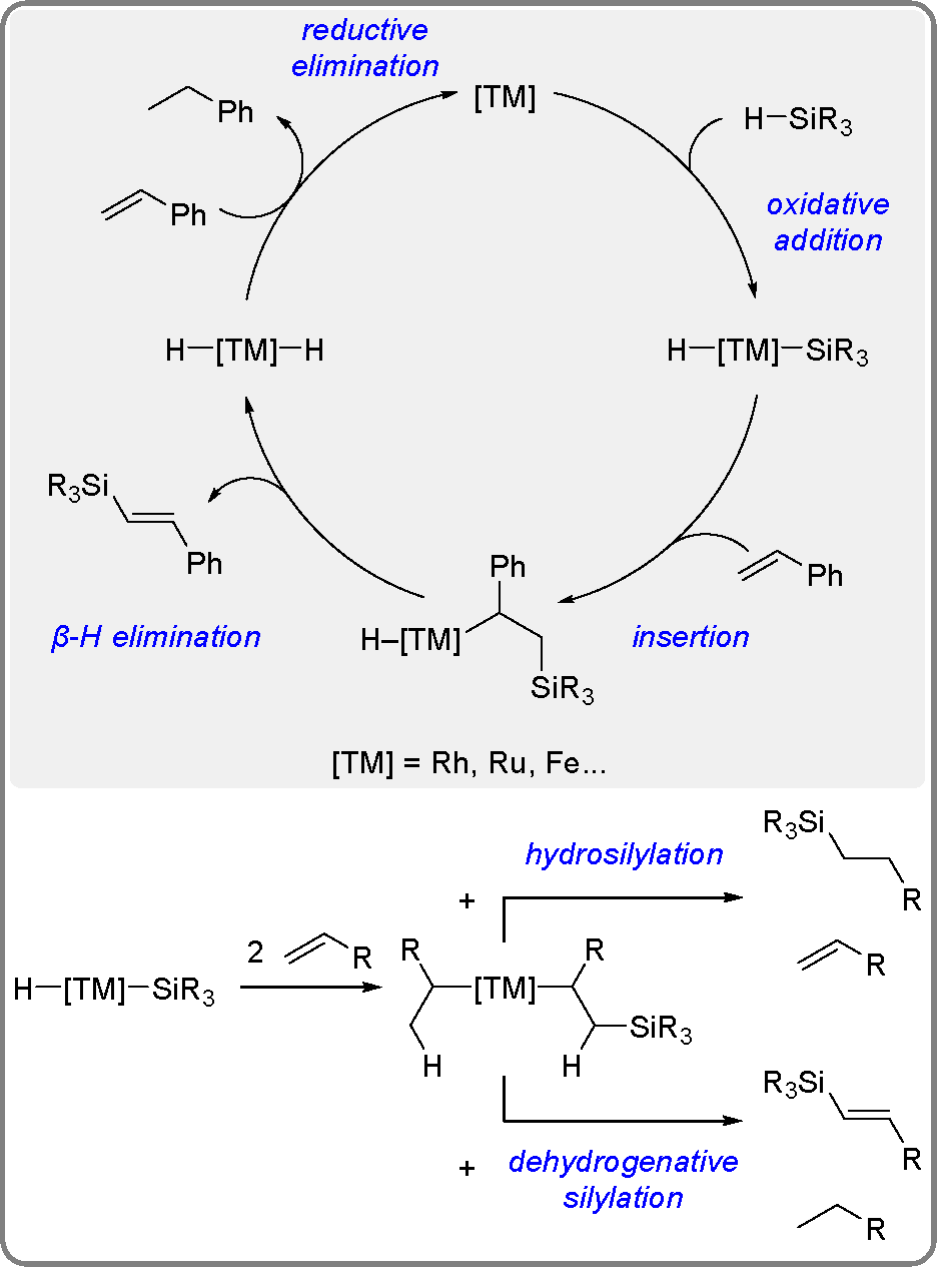
General Mechanism for the Dehydrogenative Silylation of Styrene Catalyzed by the Fe and Co Triad

In contrast to the well-established platinum-catalyzed hydrosilylation, dehydrogenative silylation appears to involve iron and cobalt triad complexes, particularly cationic ones, and nickel complexes as its favored catalysts. A general scheme of the dehydrogenative silylation of styrene catalyzed by Rh, Ru, and Fe^13^ complexes is given in Scheme 5.

The key step of the two alternative reactions is a competitive σ-H transfer from two ligands (σ-alkyl and σ-silylalkyl) of the intermediate formed in the reaction. The mechanism of the dehydrogenative silylation of vinyl derivatives in the presence of a nickel equivalent of Karstedt’s catalyst, with the first documented insertion of an olefin (styrene) into the Ni–Si bond, has been published.^33^

The high cost of Pt-based hydrosilylation catalysts—caused by the low abundance of platinum in the earth’s crust, the consumption of Pt-metals in silicone chemistry, and the low abundance of other noble metals (Pd, Ir, Rh) and their toxicity—has recently motivated experts on the catalysis of hydrosilylation to concentrate on earth-abundant TMs, such as iron, cobalt, and nickel complexes, as efficient homogeneous catalysts. Much progress has been recently seen in the investigation of the first-row TMs in the hydrosilylation of multiple bonds (e.g., alkenes, alkynes, C=O).

**Scheme 6:**
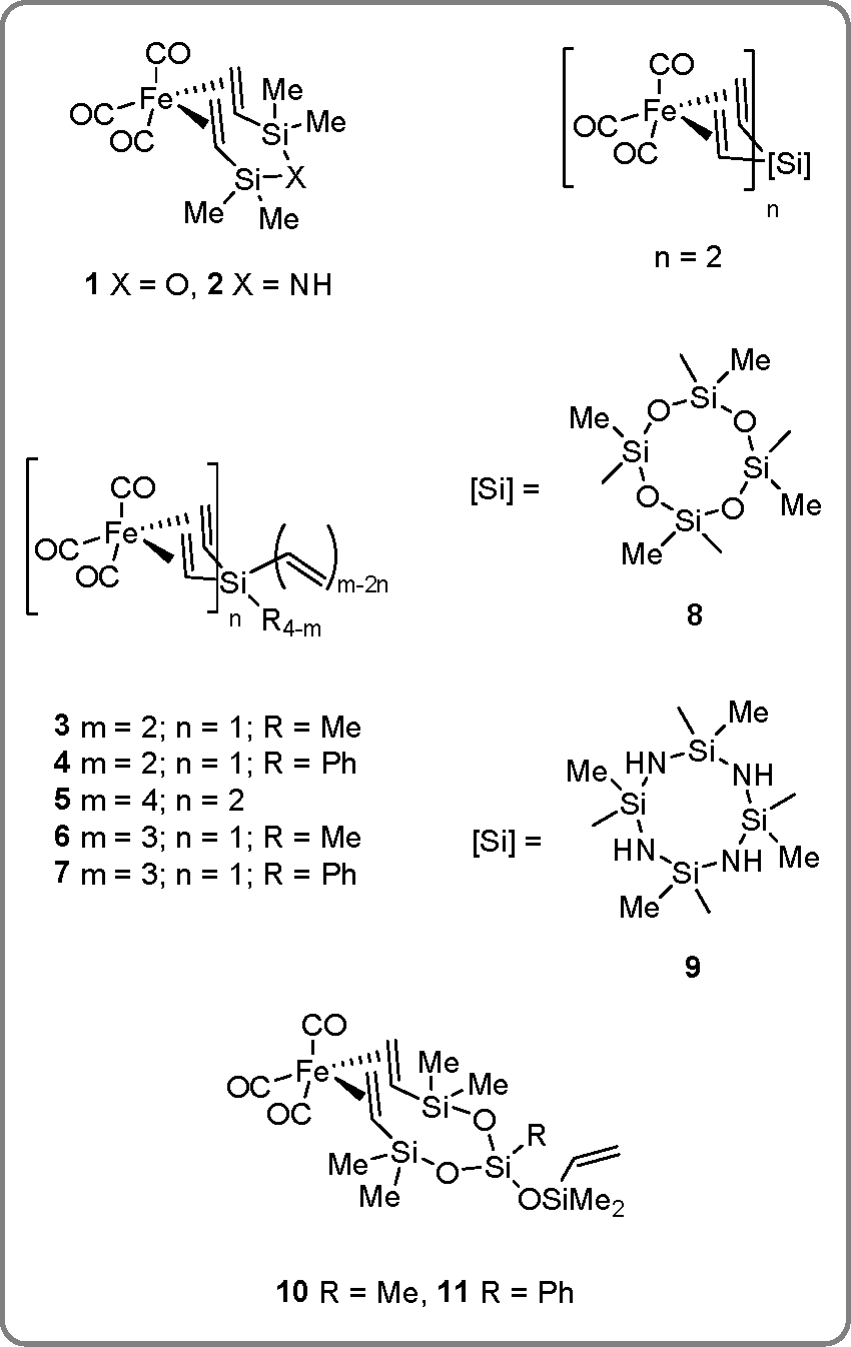
A New Class of Well-Defined Fe(CO)_3_-Based Complexes Stabilized with Multivinylsilicon Ligands

Therefore, based on the information from the past decade as cited in numerous reviews and original publications, we concentrate here predominantly on the coordination chemistry of earth-abundant metals (mainly Fe, Co, and Ni) and their role in the catalysis of the hydrosilylation of alkenes and alkynes and other multiple bonds, which can explain their wide application in synthesis and silicone industry. However, examples of other novel TM–silicon complexes introduced as homogeneous and heterogeneous catalysts for efficient and selective hydrosilylation and dehydrogenative silylation have also been discussed. The studies on the reactivity of iron(0) carbonyl complexes of the 1960s led to the synthesis of many organometallic compounds by the substitution of carbonyl ligands with olefins, dienes, and alkynes.^21,23,24,34^ Diene ligands stabilize Fe(CO)_3_ reactive species as hydrosilylation catalysts.^17^ We developed a general strategy for the synthesis of new, well-defined iron(0) carbonyl complexes [Fe(CO)_3_(L)] and [Fe(CO)_3_]_2_L, stabilized by multivinylsilicon ligands (by analogy to Karstedt’s catalyst; see Scheme 6), which appeared to be very active catalysts for the hydrosilylation and/or dehydrogenative silylation of alkenes as well as the cross-linking of silicone fluids.^35−37^ The X-ray structures of **1**, **2**, **4**, **5**, and **9** have also been published.

**Scheme 7:**
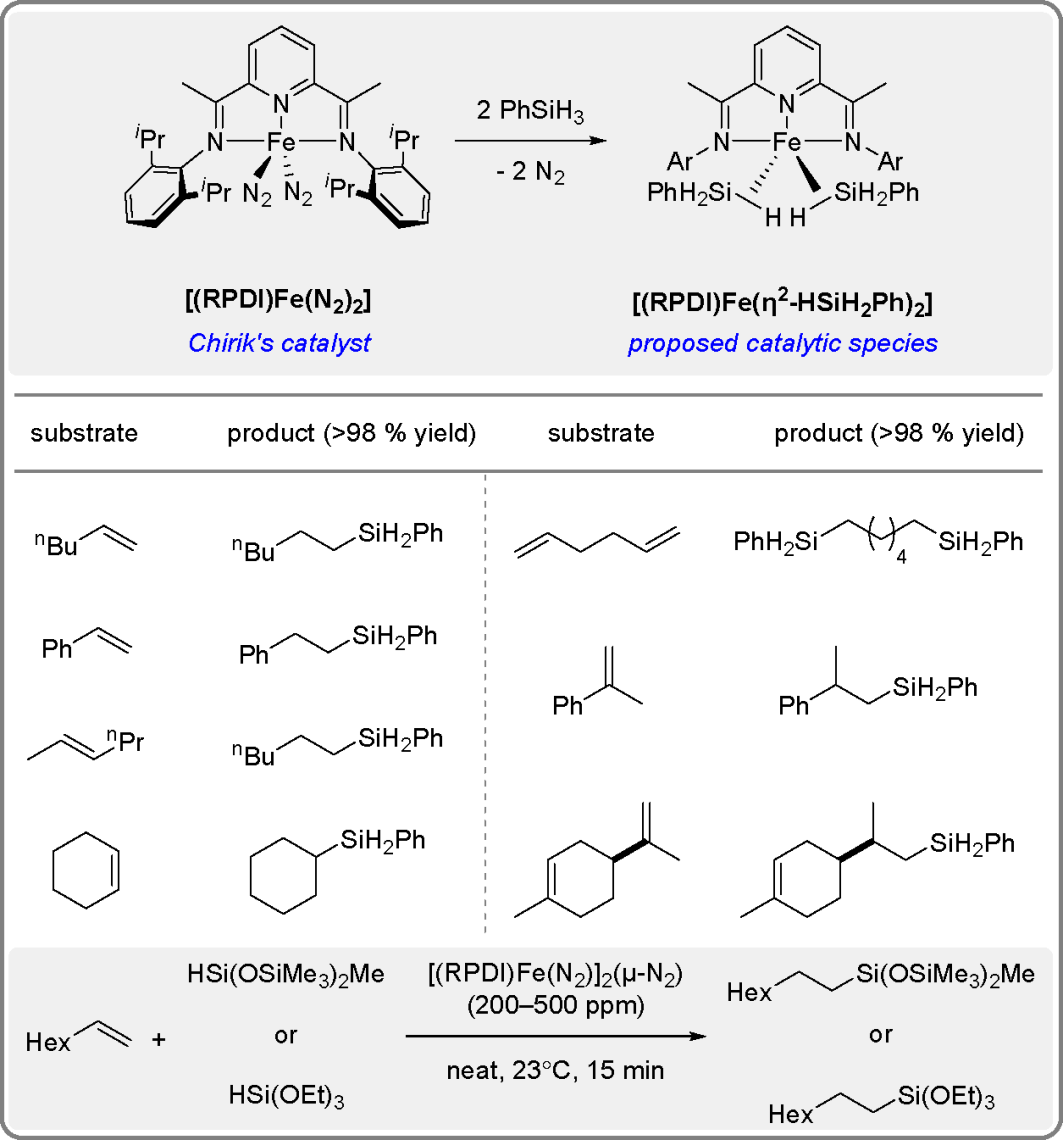
Selective Iron-Catalyzed Hydrosilylation of Alkenes in the Presence of [(RPDI)Fe(N_2_)_2_] Catalysts^24,27^

The UV-accelerated reaction of iron(0) carbonyl precursors with various multivinyl-substituted silicon derivatives, as shown in Scheme 7, resulted in a series of well-defined, five-coordinated Fe(0) complexes in which highly reactive Fe(CO)_3_ species were stabilized by two vinyl groups.^35^ The catalytic activity of all the Fe(CO)_3_ surrogates (see Scheme 7) has been examined in transformations in molecular and macromolecular Si–H/olefin systems. Detailed studies of the catalytic processes that occur between the above-mentioned compounds have shown that depending on the type of the olefin used or reaction conditions, two reactions may proceed effectively: hydrosilylation and dehydrogenative silylation.^36^ Iron disilyl dicarbonyl complexes bearing two weakly coordinated η^2^-Si–H moieties appeared to be good catalysts for the hydrosilylation of olefins and reduction of carbonyl compounds.^38^

The possibility of replacing expensive platinum by catalytically active but cheaper derivatives of Fe, Co, Ni, and other TMs and novel applications of potent hydrosilylation and dehydrogenative silylation products in the synthesis of fine chemicals in materials science have been shown over the past decade by the research groups of Obligacion and Chirik,^25^ Nakazawa,^34^ Huang,^20^ Wiesbrock,^23^ Beller,^19^ Lu,^24^ Liu,^28^ Findlater,^21^ and Nagashima,^38^ among others.

The traditional study of alkene and alkyne hydrosilylation and dehydrogenative silylation by primary silanes has been extended to include secondary and tertiary silanes and (poly)hydrosiloxanes, as well as the hydrosilylation of dienes, ketones, aldehydes, and imines.

However, the most attractive aspect from the scientific point of view is the introduction of novel TM complexes including not only monodentate and bidentate ligands but also tridentate (pincer) ligands (for recent reviews, see refs (19 and 21)). Using a ligand with the desired electronic and steric properties has appeared to be crucial for high catalytic activity and product selectivity and yield. Syntheses of new TM complexes that proved to be specific catalysts for some organosilicon products have often become the starting points for the mechanistic studies of hydrosilylation and dehydrogenative silylation processes.

**Scheme 8:**
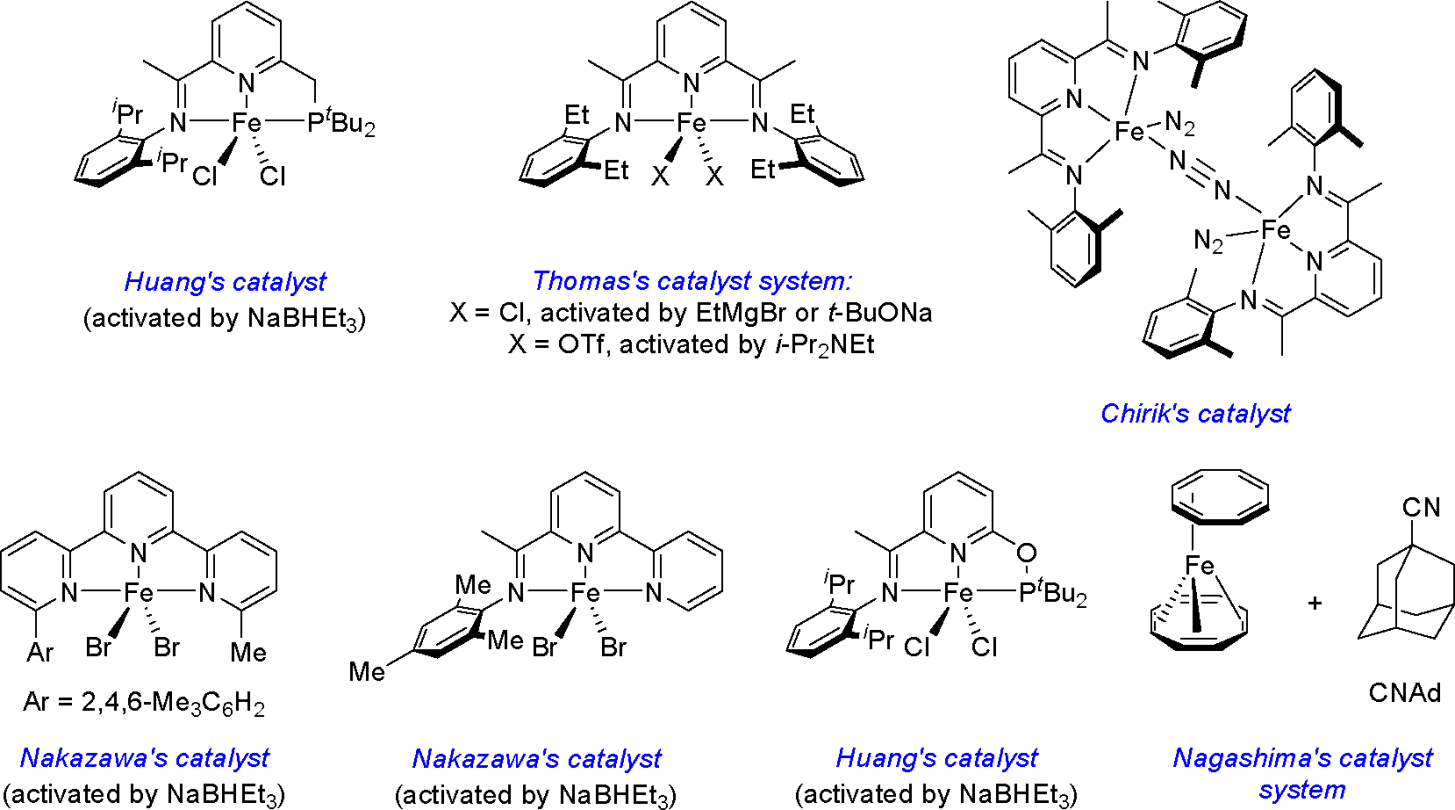
Examples of Iron Complexes for the Hydrosilylation of Alkenes (for a Review, See Ref (24))

**Scheme 9:**
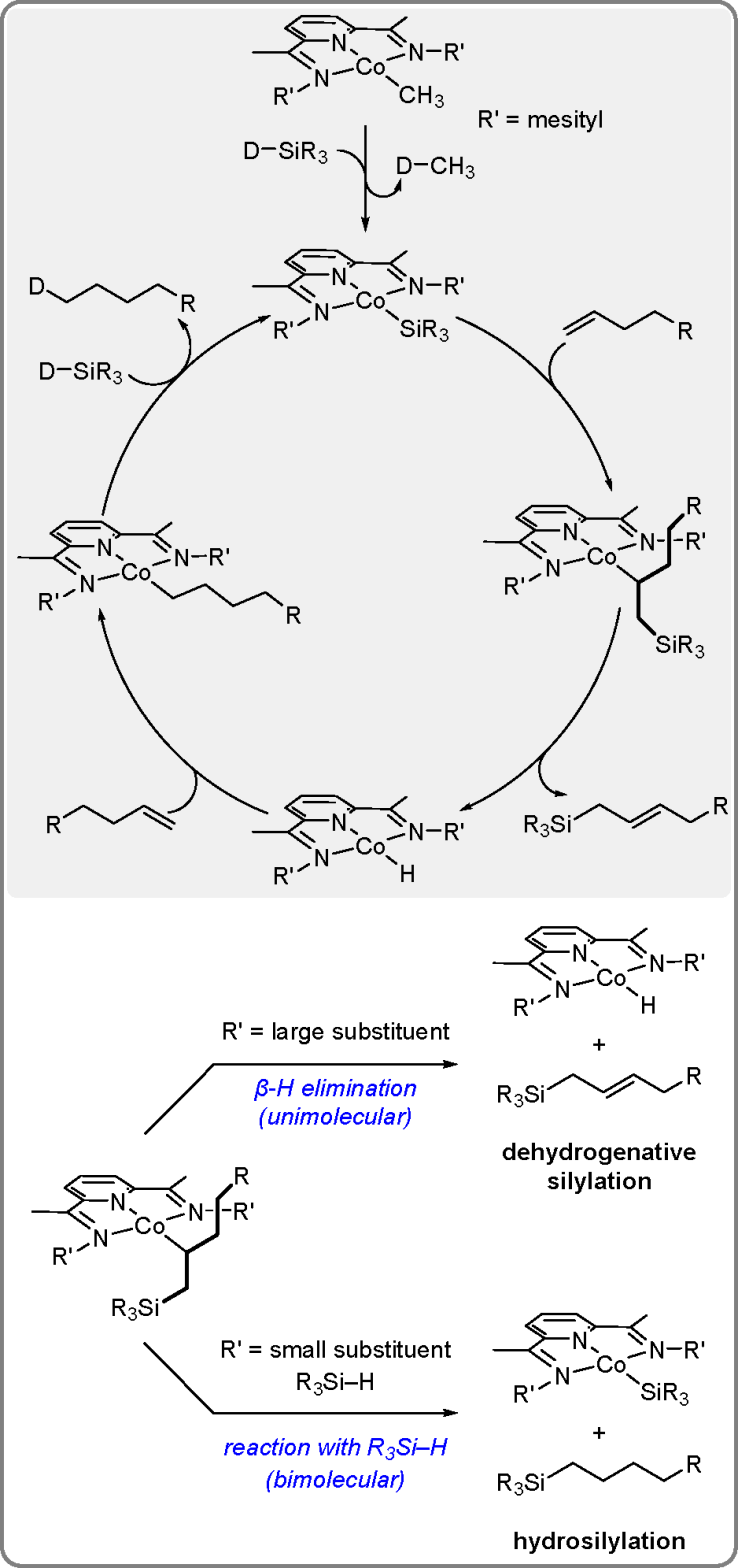
Pyridine(diamine)Co-Catalyzed Dehydrogenative Silylation of 1-Olefins

Example iron and cobalt pincer catalysts used in the hydrosilylation of alkenes, alkynes, aldehydes, and ketones are shown in Schemes 7–9 and 13–15 (see the above reviews). Certain examples of the mechanistic implications of these new systems are given below (see Schemes 7 and 9). A recent review by the Obligacion and Chirik group^25^ is a comprehensive report on the application of Fe and Co catalysts in commercially important alkene hydrosilylation, and industrially relevant tertiary silanes are predominantly used. Chirik’s experiments using precatalysts in the form of [(RPDI)Fe(N_2_)_2_], where RPDI denotes 2,6-diiminopyridine and R represents substituents at the imine atom (Scheme 7), helped find the conditions for selective anti-Markovnikov alkene hydrosilylation in a very selective synthesis of functionalized silanes and effective cross-linking of silicone fluids. Preliminary treatment of the initial complex with excess silane yielded iron bis(silane) species, proposed as active intermediates in hydrosilylation catalysis.

New iron catalysts for these reactions were developed through experiments continued by other groups (Scheme 8).

The Co–alkyl complex [(RPDI)Co(R)] (where R is CH_3_) promotes the highly selective dehydrogenative silylation of linear olefins to yield allylsilanes.^22^ The starting alkene acts as the H acceptor to be converted to the corresponding alkane (Scheme 9). Large R′ substituents at the imine promote unimolecular dehydrogenative silylation, whereas small substituents promote bimolecular reactions with hydrosilylation.

The proposed mechanism involves initiation of a precatalyst by the silane to yield a Co–silyl intermediate, followed by selective alkene 2,1-insertion and β-H elimination to form a Co–H intermediate and allylsilane. Subsequently, alkene insertion is followed by the reaction of the silane with a Co–alkyl to regenerate the Co–silyl intermediate. The characteristic feature of this mechanism is that there is no need for a formal oxidative addition step of the silane; 1e^–^ oxidation of the metal and ligand is only required, which is unavailable for heavier transition elements.

Another example of the above rule is the application of bidentate aryl-substituted and alkyl-substituted α-diimine (DI) ligands to form Ni(DI) fragments,^25,26^ which are isoelectronic to the active Fe(PDI) species. Combining the 2,6-diisopropyl phenyl-containing ligand (DIPO DI) with hydrocarbon-soluble Ni(II) carboxylates produces active and anti-Markovnikov selective catalysts for the hydrosilylation of 1-octene with tertiary alkoxysilanes and siloxysilanes.

**Scheme 10:**
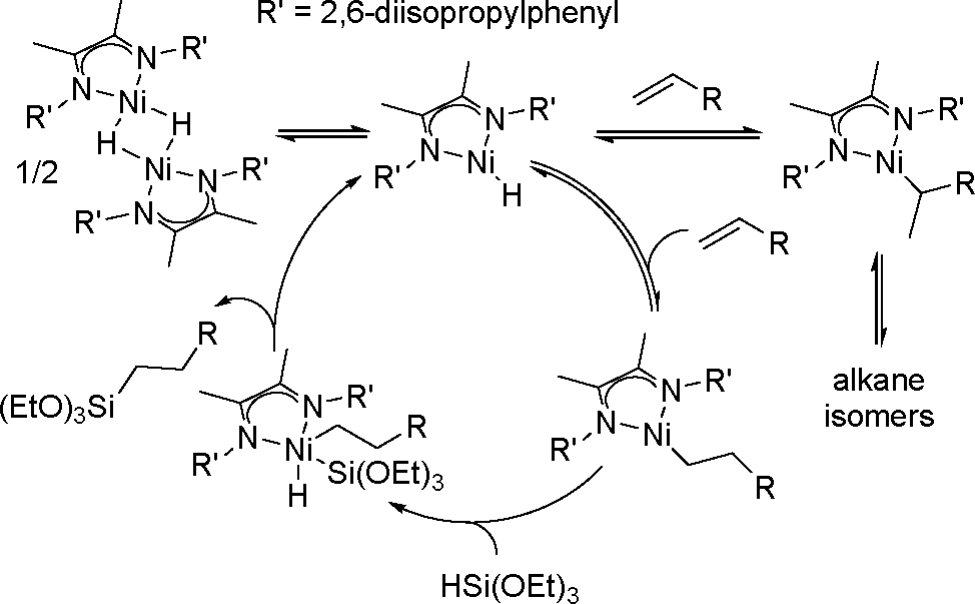
Mechanism of Alkene Hydrosilylation Catalyzed by the (α-Diimine)Ni Complex

The mechanism presented in Scheme 10 is supported by stoichiometric and deuterium-labeling studies as well as DFT calculations, and involves Ni-hydride dimer dissociation into monomers, followed by fast reverse alkene insertion and turnover that limits C–Si bond formation.^25^

**Scheme 11:**
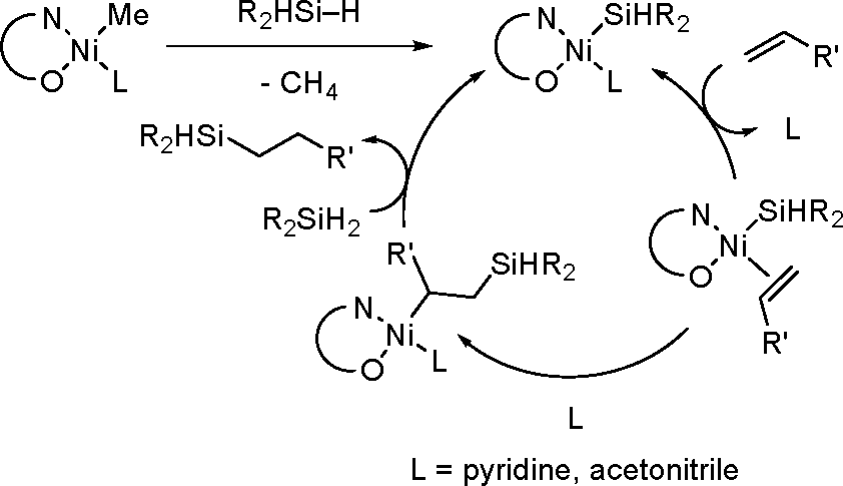
Mechanism of Olefin Hydrosilylation by Secondary Silanes Using (Salicylaldiiminato)Ni(II) Catalysts

A different mechanism of olefin hydrosilylation by secondary silanes has been suggested by the Shimada and Nakajima group, using highly active and selective salicylaldiiminate Ni catalysts^39^ (Scheme 11). The hydrosilylation occurred at room temperature, but tertiary and primary silanes could not be used. The internal alkenes were also successfully hydrosilylated at 50 °C. ^1^H and ^29^Si NMR spectroscopic studies provided direct evidence that Ni–Si complexes were generated from the initial complexes with Ph_2_SiH_2_. Therefore, the proposed mechanism involves an active Ni–Si intermediate instead of the Ni–H complex.

The TM-catalyzed hydrosilylation of alkynes is one of the best methods for the synthesis of vinylsilanes, used as valuable starting fine chemicals for organic synthesis. The hydrosilylation process of terminal alkynes can produce three possible isomers: (*E*)-β-, (*Z*)-β-, and α-vinylsilanes. (*E*)-β-isomers are usually thermodynamically favored and therefore generated with high selectivity (for a review, see ref (28).).

**Scheme 12:**
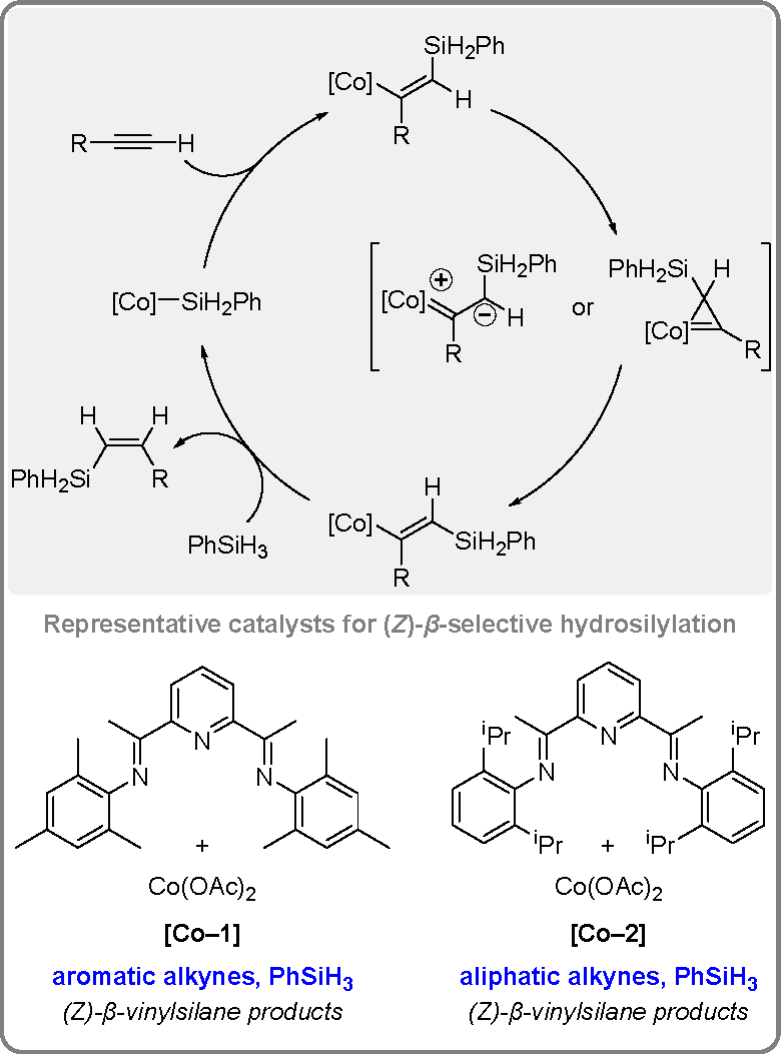
Mechanism of the Z-Selective, Anti-Markovnikov Hydrosilylation of Terminal Alkynes by Phenylsilanes

The (*Z*)-selective hydrosilylation of terminal alkynes is more challenging than the synthesis of E-isomers. In a recent study by the Ge group, sterically congested pyridine-2,6-diimine (Mes PDI) (Co-1) and i-PrPDI (Co-2) pincer ligands have been found to be effective in the Co-catalyzed (Z)-selective anti-Markovnikov hydrosilylation of many terminal alkynes^40^ (Scheme 12).

Deuterium labeling and kinetic measurements supported the catalytic cycle starting from a Co(I)–silyl intermediate.

**Scheme 13:**
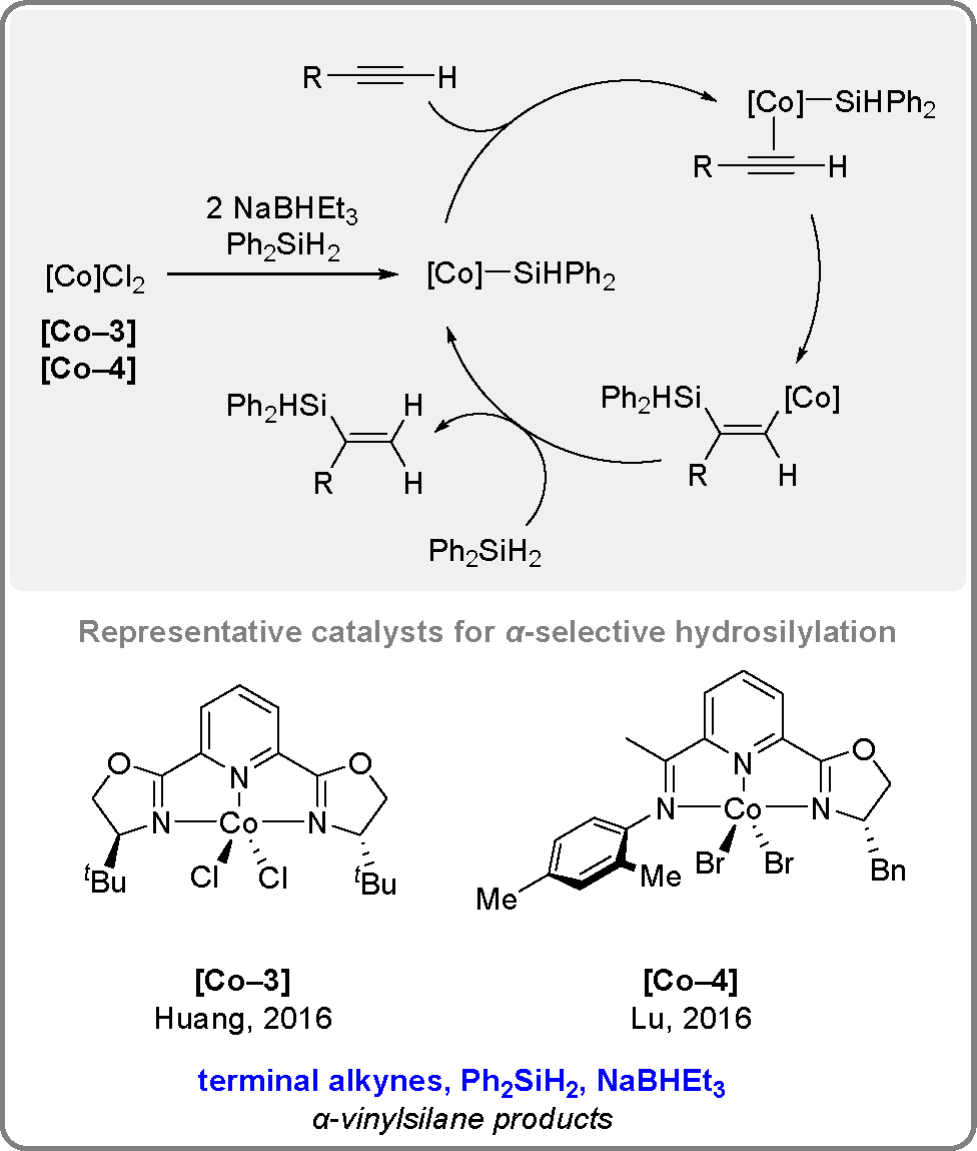
Postulated Catalytic Cycle for the α-selective Hydrosilylation of Terminal Alkynes by Secondary Silanes Using Co-3 and Co-4 Catalysts

However, the Huang^41^ and Lu^42^ groups have independently reported the α-selective hydrosilylation of terminal alkynes by secondary silanes using Co-3 and Co-4 catalysts. The mechanistic scheme (Scheme 13) proposes selective formation of a Co–silyl intermediate, but because of steric effects, selective 1,2 insertion proceeds to form a Co–alkenyl intermediate and to finally eliminate the α-adduct.

#### 1.1.2 Homogeneous Hydrosilylation of Carbonyl Compounds and Imines

**Scheme 14:**
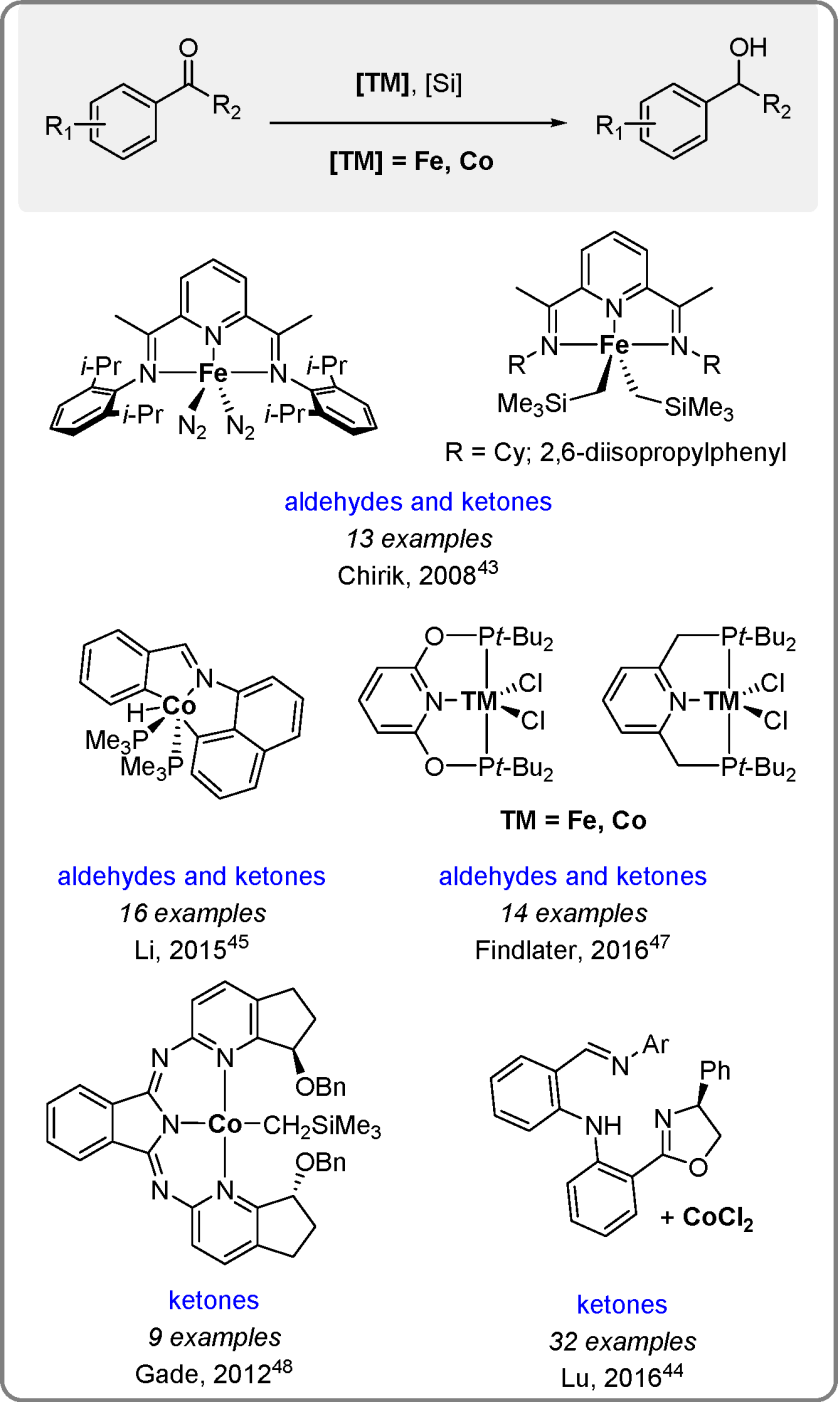
Selected Examples of Iron and Cobalt Pincer Catalysts for the Hydrosilylation of Aldehydes and Ketones

**Scheme 15:**
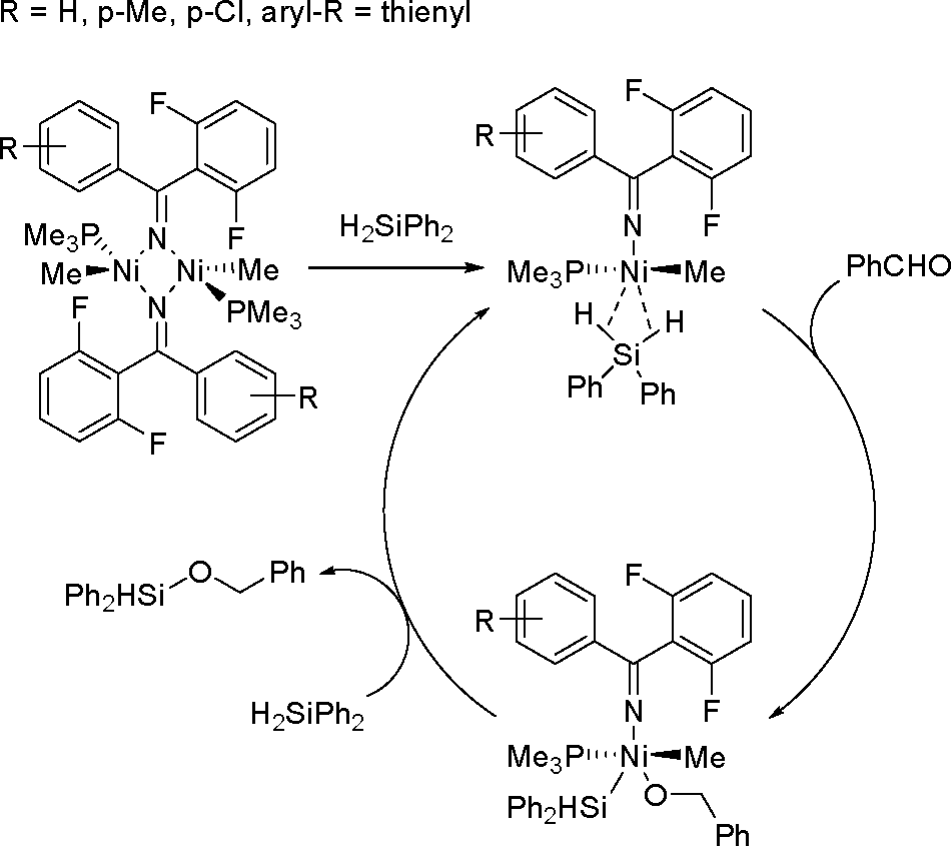
Mechanism of the Nickel Complex Catalysis of Aldehyde Hydrosilylation by Secondary Silanes

**Scheme 16:**
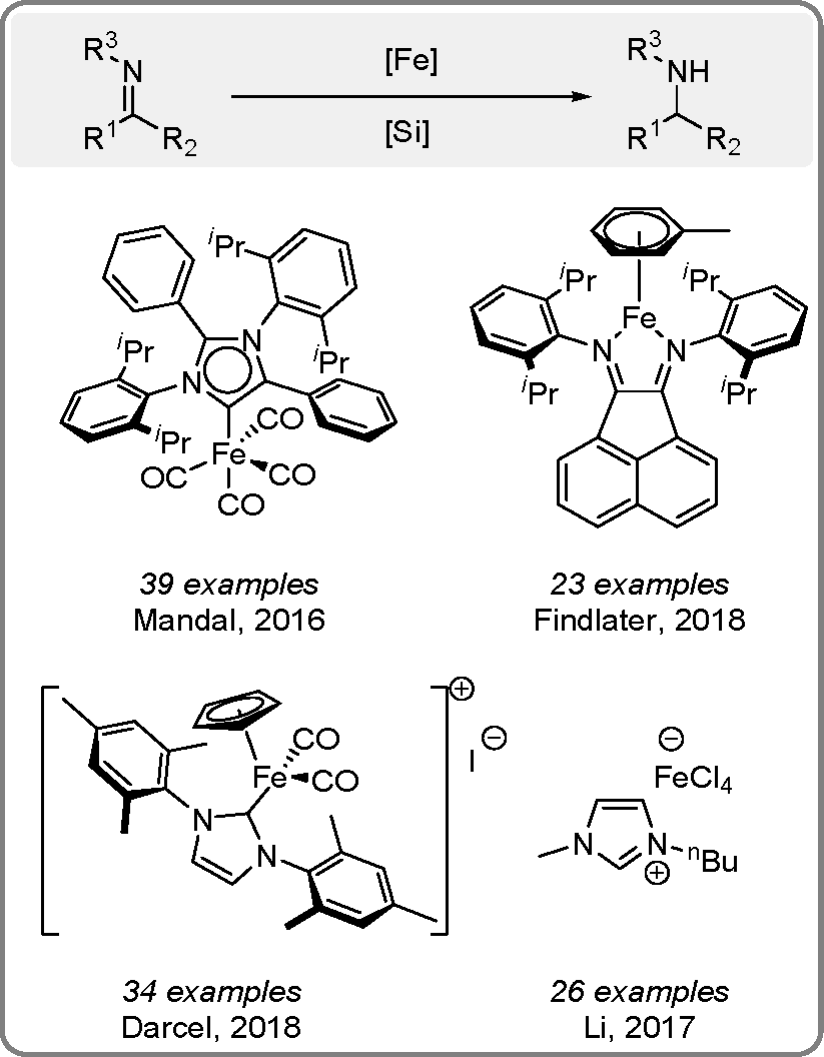
Hydrosilylation of Imines Catalyzed by Fe Complexes^21^

Continuing the review on the new developments in catalysis (by Fe, Co, and Ni complexes) from the previous decade, we also present the hydrosilylation of aldehydes, ketones, esters, and imines (for reviews, see refs (21, 23)). The reviews summarize novel noninnocent redox-active ligands and mostly pincer ligands of the above metal triad (see Schemes 14–16).

Some examples of iron pincer complexes as catalysts for the hydrosilylation of ketones and aldehydes have been reported by the Chirik group.^43^ The number of mechanistic studies is limited, and most studies have not been confirmed. Yet some evidence for the generation of active species, including Fe–H and Co–H bonds, has been reported. In situ activation of the catalysts with NaHBEt_3_ to generate Co–H bonds has been reported by the Lu^44^ and Findlater^21,47^ groups, but the activation mechanism proposed by Li’s group^45^ using a hydrido-cobalt complex does not require NaHBEt_3_.^21^

On the other hand, a broad iron-hydride resonance peak detected by the Enders and Gade group^46^ using ^1^H NMR provides reliable evidence for the TM–H catalysis of carbonyl hydrosilylation.

Cobalt pincer complexes have been recently applied for enantioselective hydrosilylation of carbonyl moieties (see ref (21)). Gade et al. reported catalysis by a cobalt complex with a chiral 1,3-bis(2-pyridylimino)isoindolate ligand (see Scheme 14), which gave high yields and enantioselectivity up to 91% ee.^48^ The combination of CoCl_2_ with chiral iminophenyloxazolinylphenylamines (Scheme 14) reported by Chen and Lu^44^ showed excellent activity with enantioselectivity up to 99%.

The Li group used a binuclear imine/nitrogen-bridged nickel catalyst for aldehyde hydrosilylation.^49^ The mechanism they proposed includes preliminary cleavage of the binuclear bond to form a mononuclear species containing the (η^2^–Si-H)Ni(II) motif. One Si–H bond is cleaved and then an aldehyde molecule is inserted. A second H_2_SiPh_2_ molecule is involved in the reductive elimination, while the silane–nickel intermediate is recovered in the cycle^49^ (Scheme 15).

Hydrosilylation of imines catalyzed by iron complexes has recently been developed as a reductive method for the synthesis of amines. Carbene ligands and ionic liquids are predominantly used for iron catalysts (see Scheme 16).^50,51^ A mechanistic study by Mandal group^51^ showed that the hydrosilylation process occurred according to the Chalk–Harrod mechanism, involving the oxidative addition of Si–H followed by hydride migration in the imine and reductive elimination of the product.

The Findlater group observed general tolerance to substrates having various functional groups when the [(dpp)(BIAN)Fe(η^6^-toluene)] complex was used as a catalyst for aldimine reduction into secondary amines.^21^ Photo- and peroxide-initiated catalysis by metal complexes is another method applied in hydrosilylation.^13,18^

#### 1.1.3 Immobilized TM Complexes as Catalysts

**Scheme 17:**
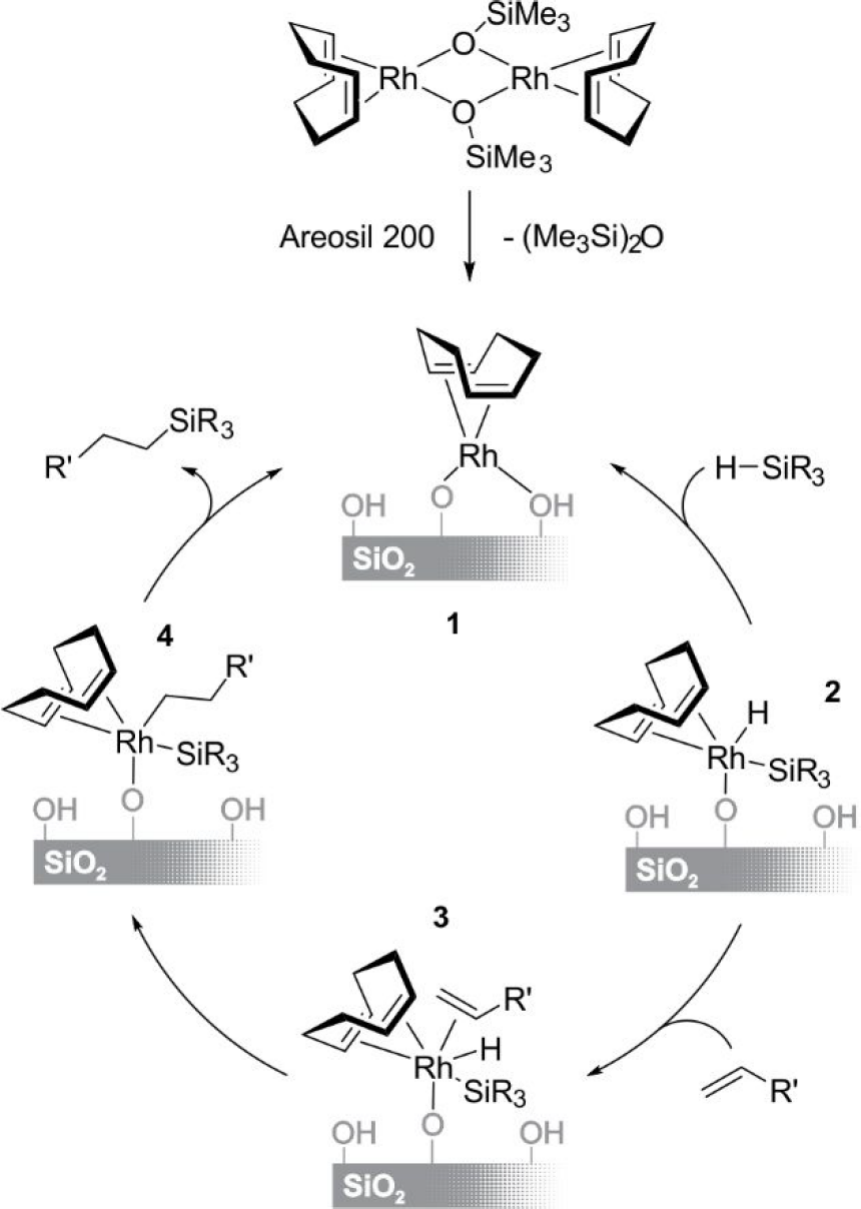
Mechanism of the Heterogeneous Catalysis of Alkene Hydrosilylation by a Surface Rhodium (Diene)siloxide Complex

Although most of the recent reviews address the important developments on cheaper homogeneous complex catalysts, some of the reviews emphasize the need for recyclable heterogeneous catalysts, that is, TM-complexes immobilized on appropriate polymers or inorganic supports such as silica. We reported the synthesis and characterization of a well-defined rhodium–siloxide complex immobilized by the direct reaction of a molecular siloxide precursor with Aerosil^52^ (Scheme 17).

Solid-state NMR results confirmed the presence of a surface siloxide complex (**2**) identified as a product of the oxidative addition of PhSiMe_2_H to the surface siloxide complex (**1 → 2**). The absence of disiloxane elimination (confirmed by GC-MS) also shows involvement of such a key intermediate in the heterogeneous system, which apparently differs from the intermediate in the homogeneous system. The subsequent alkene coordination to the surface siloxide–Rh complex (**2 → 3**) is followed by its insertion into a Rh–H bond (**3 → 4**), with the final elimination of the product and regeneration of the stable surface complex (**1**). The interaction of the silanol group in **1** is responsible for the high stability of such a single-site rhodium catalyst which can be recycled at least 10–20 times without a decrease in high yield and selectivity.

**Scheme 18:**
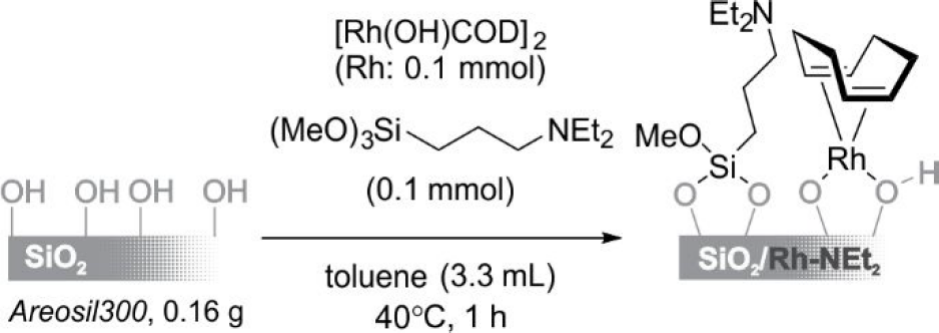
Preparation of a SiO_2_/Rh–NEt_2_ Catalyst

The simultaneous immobilization of a Rh complex and a tertiary amine on the SiO_2_ surface afforded a highly active supported catalyst for the hydrosilylation of terminal olefins^52^ (see Scheme 18). The interaction between the Rh complex and tertiary amines is a key factor in the preparation of a highly active catalyst. The TON was close to 1.9 × 10^5^ over 24 h, and a variety of silanes and olefins were shown to be effective substrates.^53^

The catalytic mechanism by supported metal complexes is not yet clear. Thus, it is possible that the anchored complex could reversibly detach from the support during the reaction and that after detachment it would act in either a homogeneous or a heterogeneous manner depending on the system.

Although several supported Pt nanoparticles (NPs) have been developed for hydrosilylation, in some cases, poor recyclability caused by leaching was observed. Therefore, a new concept for catalytic hydrosilylation, which can be applied in industry and academia, was introduced by the Beller group. They proposed simple atom catalysts (SACs), that is, materials with isolated metal centers stabilized by neighboring salts (for example, impregnation of platinum salts on aluminum oxide nanorods).^54^ These Pt-SACs showed high stability owing to the strong binding of individual Pt atoms with their neighboring oxygen atom; therefore, the single atom-based catalyst shows significantly higher activity compared with related Pt-nanoparticles.^19^

However, a detailed mechanistic study on this type of heterogenized systems in view of TM-Si and TM-H activity in catalysis at the molecular level has been only initiated.

### 1.2 Hydrostannation of Alkenes and Alkynes

Organostannanes are tremendously useful synthetic building blocks in organic chemistry. The current most frequently used reactions for the synthesis of functionally rich rings and allyl organostannanes are the TM-catalyzed hydrostannation (hydrostannylation) of alkynes, alkenes and allenes, and stannylmetalation that affects their protonation.^10^ A comprehensive review of metal-catalyzed hydrostannation before the year 2000 was published by Lautens et al.^55^

**Scheme 19:**
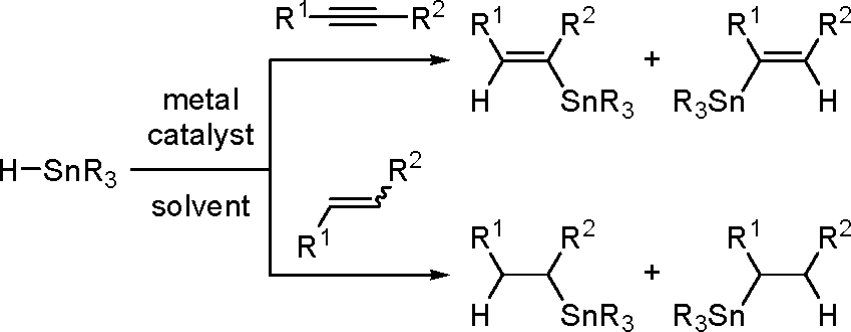
Hydrostannation of Alkynes and Alkenes

The general equation for the hydrostannation of the above-mentioned unsaturated compounds is shown in Scheme 19.

**Scheme 20:**
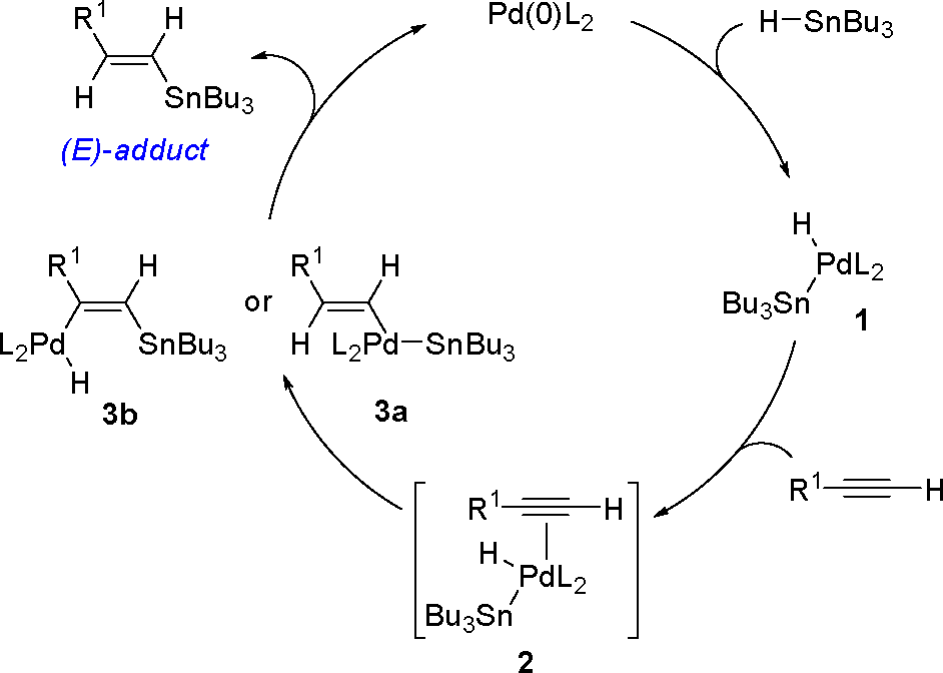
Proposed Mechanism of the Pd-Catalyzed Hydrostannation of Alkynes

The reaction presented above usually occurs with *cis* stereoselectivity as a consequence of the reaction mechanism. The use of organostannanes is often hampered by their toxicity. Trimethyl and triethyltin compounds are especially poisonous, and their toxicity decreases with increasing alkyl chain length. The most commonly used tributyltin compounds are moderately toxic, while trioctyltin compounds exhibit almost no toxicity.^56^ The palladium-catalyzed hydrostannation of alkynes is the most widely used procedure for the synthesis of (*E*)-alkenylstannanes.^55^ The regiochemistry of addition is controlled by many factors, of which the structure of the alkyne substrate is the most critical. The mechanism (Scheme 20) assumes an oxidative addition of R_3_Sn–H to an L_2_Pd(0) complex (**1**), followed by coordination of the alkyne with a vacant orbital on the metal atom and subsequent addition of the coordinated Pd–H bond of **2** into the alkyne π-bond to give complexes **3a** and/or **3b** via the competing pathway of hydropalladation and stannylpalladation. Finally, however, a β-(*E*)-alkenylstannane is formed, and the Pd(0) catalyst is regenerated.

**Scheme 21:**
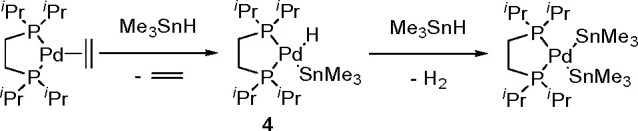
Isolation of Pd(II) Hydride, Stannyl Intermediate

The proposed formation of an intermediate (**1**) has been supported by the isolation of a *cis* Pd(II) hydride trialkylstannyl intermediate (**4**) when bulky bidentate phosphine ligands were added (Scheme 21).^57^ However, numerous subsequent experiments were proposed to test whether hydropalladation or stannylpalladation of specific alkynes occurred.^58,59^

**Scheme 22:**
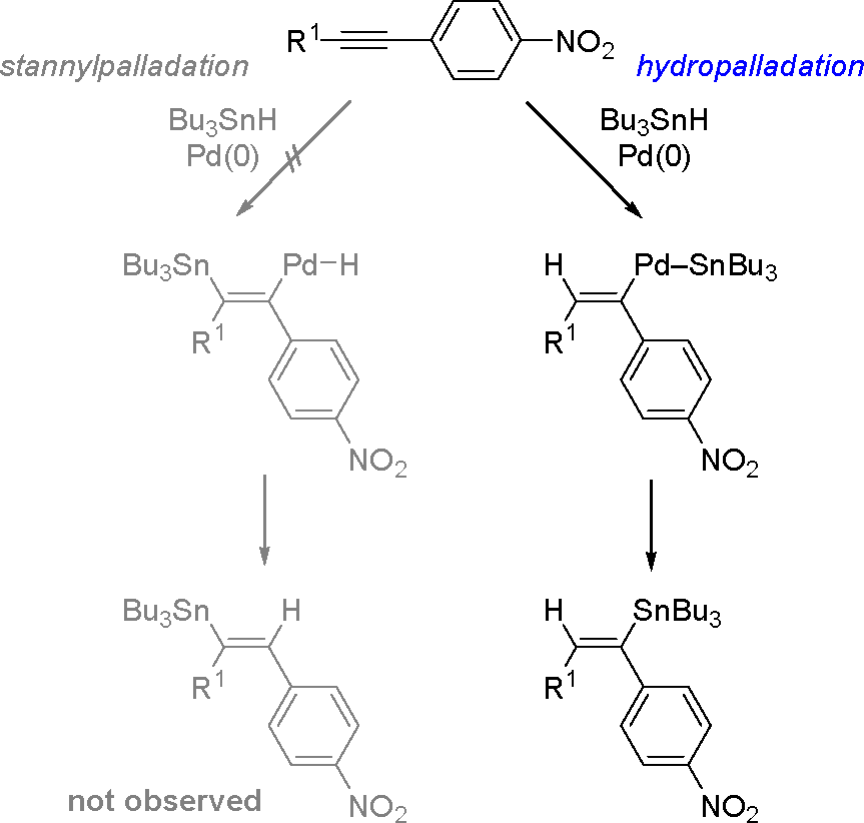
Pd-Catalyzed Hydrostannation of *p*-NO_2_ Arylalkynes

Scheme 22 presents the Pd-catalyzed hydrostannylation of terminal or internal aromatic alkynes in which the triple bond is significantly polarized because of a nitro group on the aryl ring.^55^ Thus, the results illustrate the electronic effect of the C≡C bond on regioselectivity. Besides, Bu_3_Sn–H formally acts as a hydride donor.

**Scheme 23:**
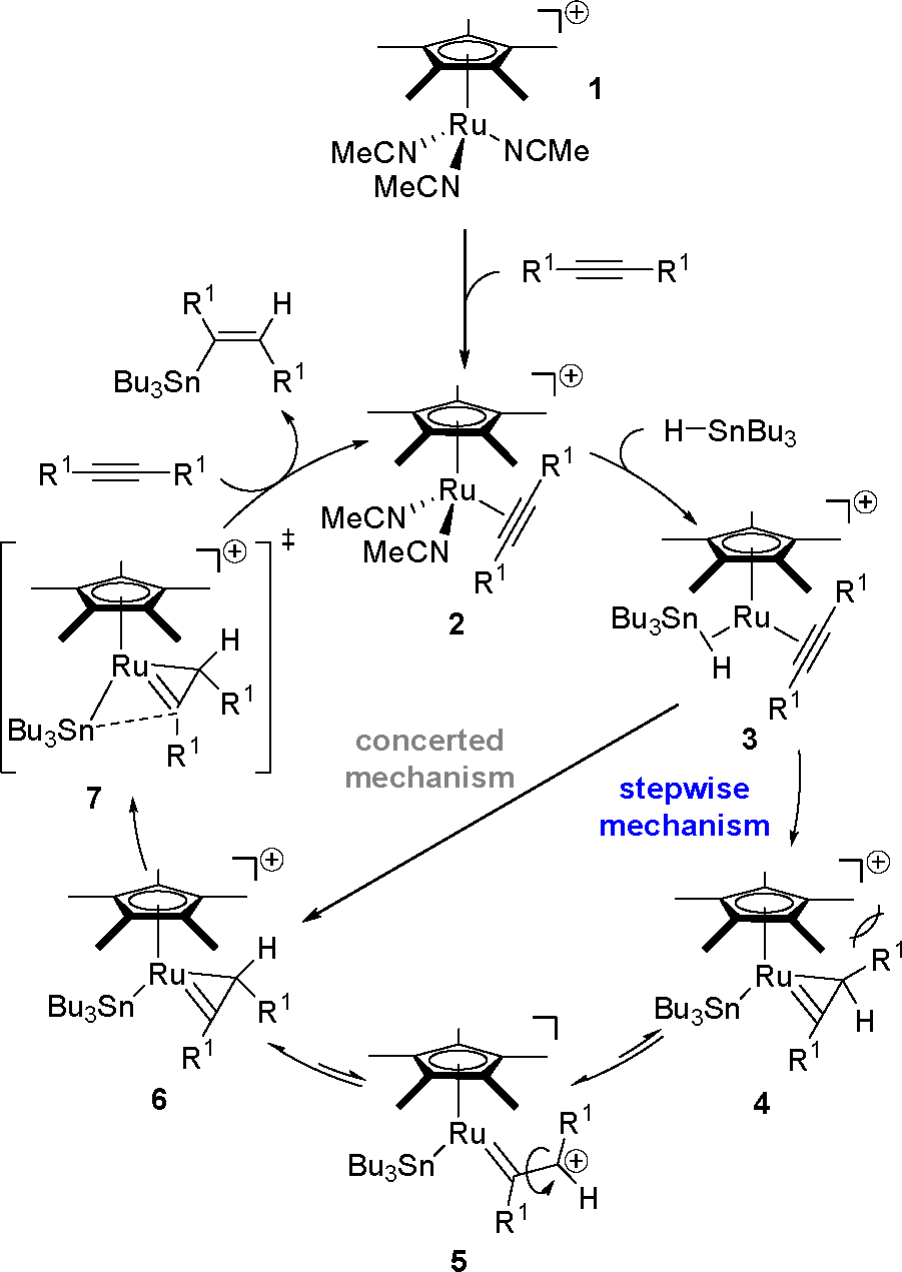
Mechanism of the *trans*-Hydrostannation of Symmetrical and Unsymmetrical Internal Alkynes Using a Ru-Complex

In addition to the most widely used numerous palladium complexes with various ligands, other metal complexes, such as cobalt, molybdenum, nickel, platinum, rhodium, ruthenium, and tungsten, have been employed in the hydrostannation of alkynes for the past 10 years.^57^ In particular, in contrast to all the other TM-catalyzed hydrostannation reactions documented in the literature, the reaction of symmetrical and unsymmetrical internal alkynes in the presence of ruthenium complexes^60−62^ (Scheme 23), originally proposed by the Trost group,^63^ was discussed in terms of mechanisms by the Fürstner.^62^

The first step of the reaction is the coordination of the alkyne with the electrophilic metal center of **1** to give intermediate **2**, followed by the coordination of Bu_3_SnH to provide **3**, in which the R^1^ group of the alkyne acts as a four-electron donor. Subsequently, after inner-sphere hydride delivery, intermediate **4** is formed, in which the R^1^ group is oriented toward the bulky Cp* ligand. Metallacyclopropene (**4**) may reversibly isomerize into **6** in which the R^1^ substituent is farther away from the Cp* ligand. These steric factors induce *trans*-hydrostannation and final reductive elimination of **6** via **7**, which places Sn in an *anti* position to the hydride and leads to (*E*)-vinylstannane. These considerations cannot exclude a concerted mechanism from **3** to **6**.

Recently, an experimental and theoretical study by the Fürstner^62^ has extended the above detailed picture to the ruthenium-catalyzed hydrometalation of internal alkynes by R_3_M–H (where M is Si, Ge, Sn). Ruthenacyclopropene appeared as a key intermediate in the final *trans*-addition product.

### 1.3 Hydrogermylation of Alkynes

Like organosilanes, organogermanes have recently been found to undergo reactions different from the Stille–Suzuki reactions for Pd-catalyzed cross-coupling (for a review, see ref (64).). Organogermanes are synthesized via the TM-catalyzed hydrometalation of alkene and alkyne derivatives by hydrogermanes according to the Chalk–Harrod mechanism presented in Scheme 3.

**Scheme 24:**
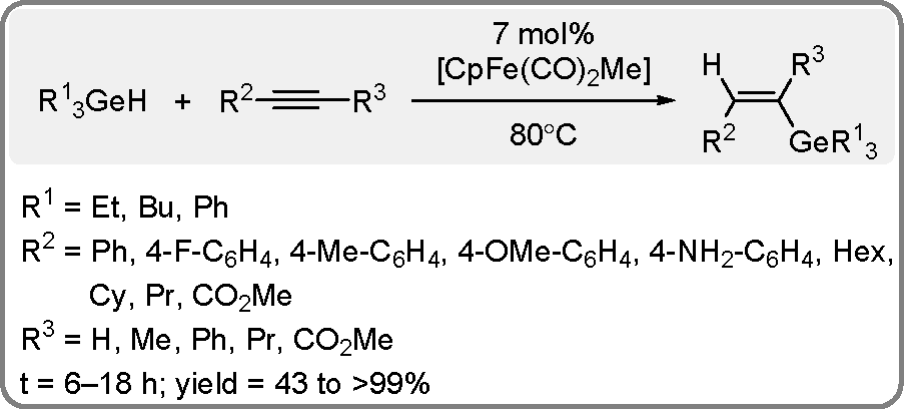
Iron Complex-Catalyzed Hydrogermylation of Alkynes

The catalytic *trans*-selective hydrogermylation of both terminal and internal alkynes has been reported by Nakazawa (Scheme 24).^65^ The crystal structure of the intermediates confirms the mechanistic considerations.

**Scheme 25:**
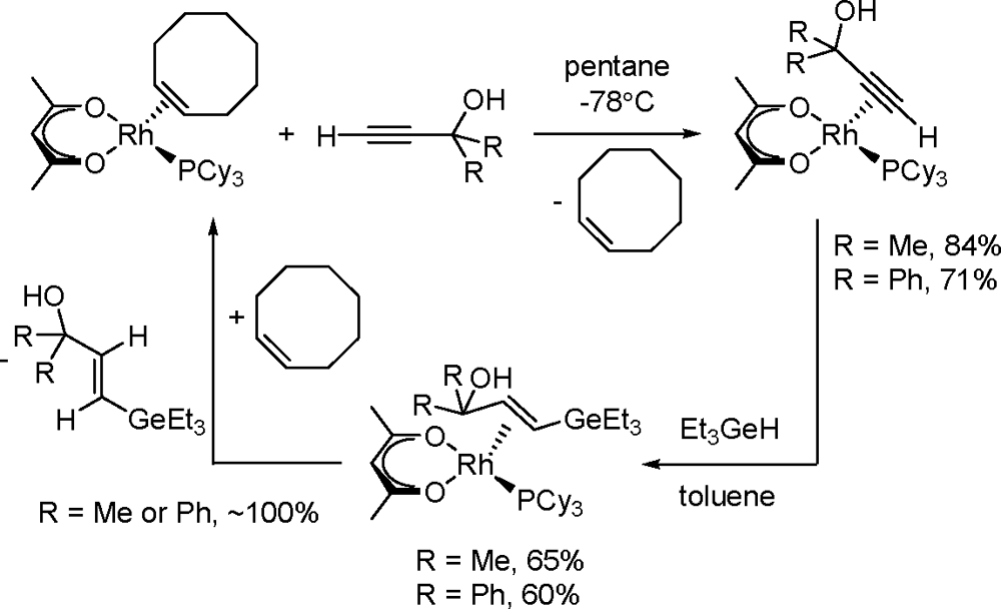
Hydrogermylation of Alkynes and Alkynols Catalyzed by a Rh-Complex

Furthermore, the hydrogermylation mechanism of alkynols or alkynes by Et_3_GeH catalyzed by rhodium complexes was confirmed by the isolation of a rhodium intermediate (Scheme 25).^66,67^

**Scheme 26:**
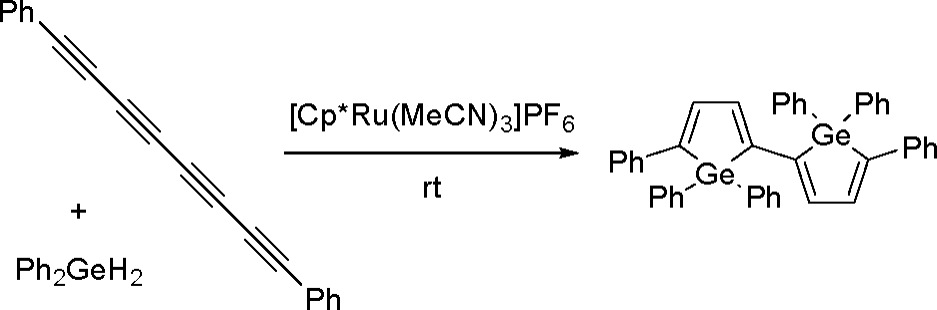
Double *trans*-Hydrogermylation Catalyzed by a Ru complex

By analogy with the *trans*-hydrogermylation of 1,3-diynes with dihydrogermanes (e.g., Ph_2_GeH_2_), [Cp*Ru(MeCN)_3_][PF_6_] allows the synthesis of 2,5-disubstituted germoles. A double addition process was applied to the synthesis of 2,2′-bigermole (Scheme 26).^68^

Hydrogermylation products have several multidisciplinary applications, for example in electronics, germanium nanowires,^69^ and materials science at large.

### 1.4 Silyl(germyl)formylation of Alkynes

**Scheme 27:**
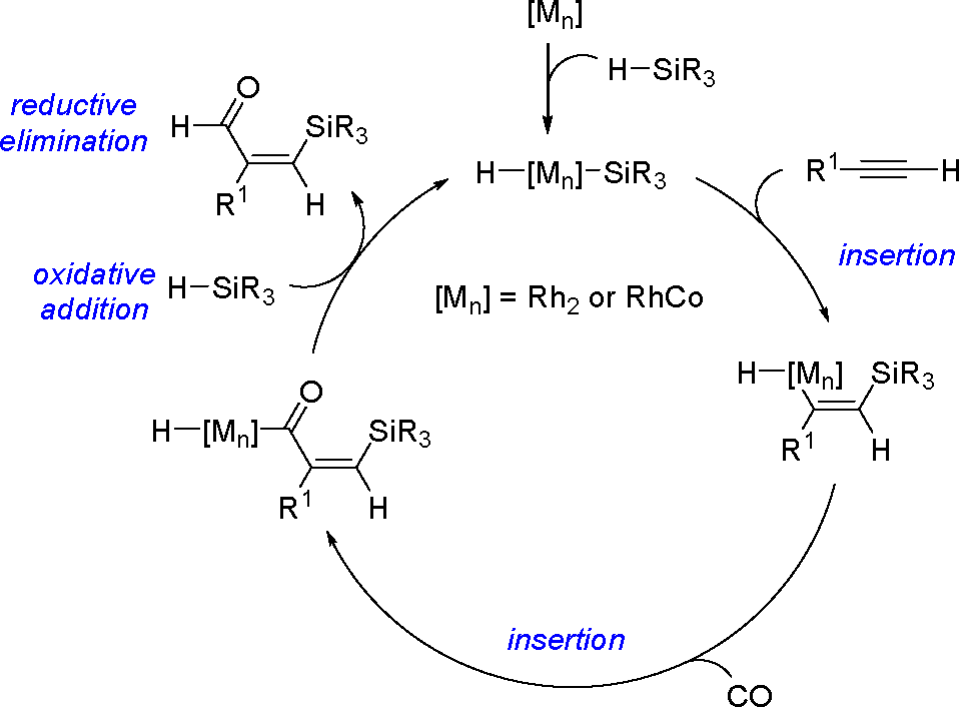
Mechanism of the Silylformylation of Alkynes

A general catalytic cycle for the silylformylation of 1-alkynes catalyzed by rhodium–cobalt clusters has already been presented^2,13^ (see Scheme 27) (for a review, see ref (70)). The mechanism involves the selective insertion of a 1-alkyne into the [M]–Si bond of the (hydrido)metal intermediate to form a silyl–vinyl–TM complex, followed by a facile insertion of CO into the vinyl–TM bond. A subsequent hydride shift gives the silylformylation product. The first catalytic germylformylation has also been reported, but only for terminal alkynes.^70^

### 1.5 Hydroboration

Hydroboration is the second most popular and suitable process for the industrial application of hydrometalation after hydrosilylation. Hydroboration of unsaturated bonds serves as a powerful strategy for the preparation of organoboranes, which are excellent reaction surrogates as they provide important reaction intermediates that could be transformed into a myriad of value-added products. They may have promising applications in the pharmaceutical, petroleum, and fragrance industries.

The borane reagents for originally used hydroboration included highly reactive boranes, such as diborane (B_2_H_6_), the BH_3_–THF complex or BH_3_–S(Me)_2_ and some alkylboranes (mainly 9-borabicyclo[3.3.1]nonane), and they allowed hydroboration of alkenes at ambient temperatures without a catalyst. It should be noted, however, that both the borylating reagent and the resulting borylated product require careful handling as they are sensitive to air and moisture, which limits their use in practical synthesis. On the other hand, dialkoxyboranes HB(OR)_2_, such as catecholborane (HBcat) and pinacolborane (HBpin), are much easier to handle because of their relative stability, but their reactions usually require elevated temperatures and/or a catalyst because of the diminished electrophilicity of the boron atoms.

#### 1.5.1 Hydroboration and Dehydrogenative Borylation of Alkenes and Alkynes

**Scheme 28:**
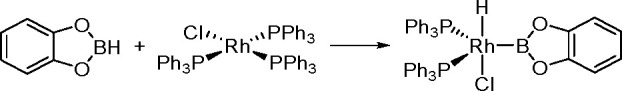
A Rhodium Complex Undergoing Oxidative Addition with Catecholborane

Using metal complexes as hydroboration catalysts became possible owing to the work of Kono and Ito who showed that Wilkinson’s catalyst in the presence of catecholborane undergoes oxidative addition^71^ (Scheme 28).

**Scheme 29:**
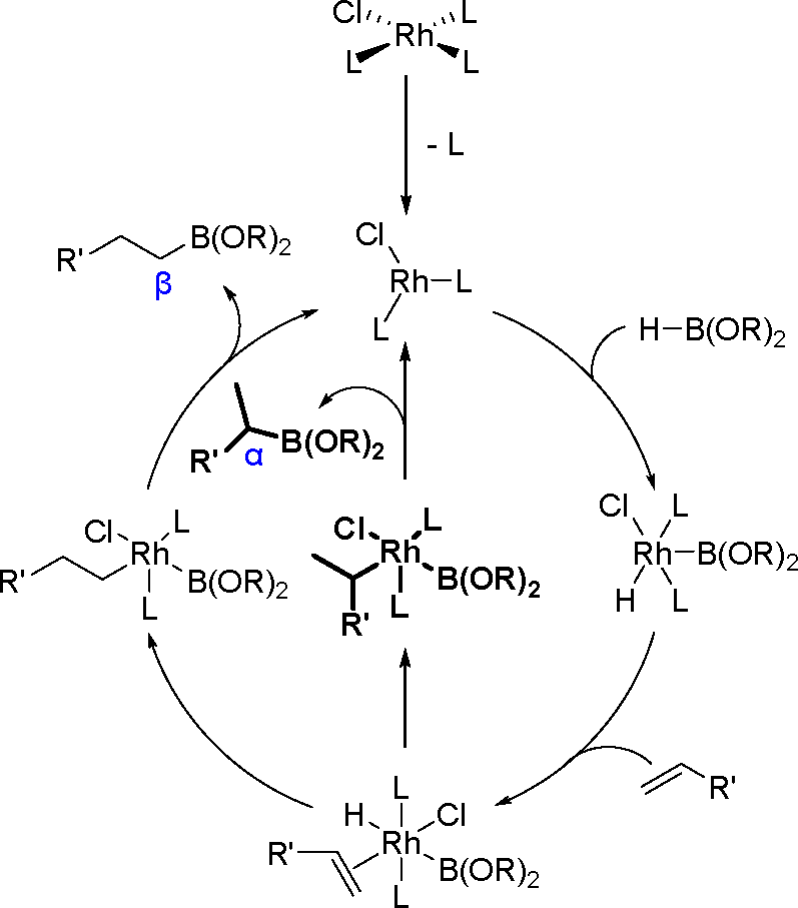
General Mechanism for the Rhodium-Catalyzed Hydroboration of Alkenes

Therefore, the first example of the catalytic hydroboration of alkenes was a reaction in the presence of Wilkinson’s catalyst, [RhCl(PPh_3_)_3_], reported by Männing and Nöth in 1985.^72^ It initiated intensive research into the catalytic aspects of hydroboration, in particular with rhodium catalysts, which led to the development of the mechanism of this reaction (Scheme 29).

The rhodium-catalyzed hydroboration reaction is thought to be initiated by the dissociation of triphenylphosphine from the Rh(I) center. The oxidative addition of the B–H bond from the borane reagent to this 14 e^–^ species is then followed by coordination of the alkene to the 16e^–^ Rh(III) hydride complex. Formation of the oxidative addition product was confirmed a few years later when the [RhCl(P*i*-Pr_3_)_2_(H)(Bcat)] complex was isolated and structurally characterized.^73^ Subsequent migratory insertion of the alkene into the rhodium–hydride bond can give two regioisomeric alkyl–rhodium(III)–boron complexes. Reductive elimination of the boronate ester regenerates the catalyst. The catalyst prepared and handled under anaerobic conditions reverses the selectivity to favor the secondary boronate ester. Although the reaction mechanism of the TM-catalyzed hydroboration of unsaturated C–C bonds has been extensively investigated through labeling experiments and theoretical calculations, there is no consensus on its mechanistic steps or transition states. What has been debated is the coordination of the alkene. In the dissociative mechanism, proposed by Männig and Nöth^72^ and supported by Evans and co-workers,^74^ the coordination is accompanied by a loss of one triphenylphosphine ligand. In the associative mechanism, proposed by Burgess et al.,^75^ the alkene binds *trans* to the chloride without the dissociation of the triphenylphosphine ligand. The mechanism has been studied by computational methods.^76,77^ Dorigo and Schleyer excluded the associative mechanism by an *ab initio* study on the dissociative mechanism,^78^ whereas Morokuma and co-workers supported the associative mechanism.^79^ Theoretical studies also predict alkene insertion into Rh–H or Rh–B bonds; however, calculations of the possible pathways indicated that the insertion barrier in the Rh–H bond is much lower than that in the Rh–B bond. On the other hand, the reaction catalyzed by iridium complexes involves alkene insertion into metal–boron bonds.^80^

**Scheme 30:**
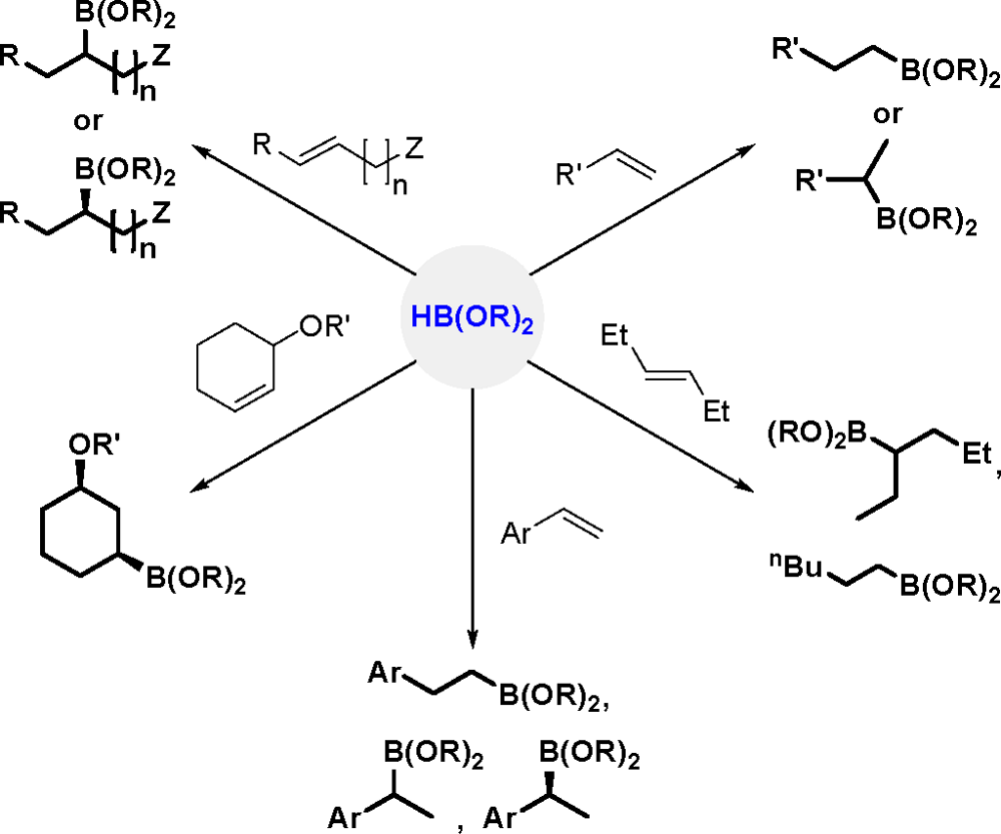
Various Organoboranes Obtained by Hydroboration

Since then, there have been many excellent reviews over the past 35 years that addressed various aspects of this reaction and formation of specific products.^11,24,25,81−85^ The chemoselectivity, regioselectivity, and enantioselectivity of the catalytic reactions vary depending on the metal center, ligands, and substrates. Rhodium is usually identified as the metal of choice to promote the efficiency of such reactions. Regioselectivity, which is relevant to a typical hydroboration reaction of terminal alkenes with alkoxyboranes, leads to two resulting forms: linear products (β-isomer, anti-Markovnikov selectivity) and branched products (α-isomer, Markovnikov selectivity)^72^ (see Scheme 30).

Linear selectivity is more commonly observed in the hydroboration of aliphatic alkenes catalyzed by rhodium complexes, while branched selectivity is observed in the reactions with vinylarenes.^74,75^ This remarkable selectivity has been attributed to a metal-stabilized benzylic intermediate. The results indicate the influence of electronic effects on regioselectivity, which also depends on the reagent and catalyst.

Although high Markovnikov selectivity was observed when cationic rhodium complexes were used in combination with tertiary phosphine ligands, the corresponding primary alkyl boronic esters were produced selectively in the presence of neutral rhodium complexes, such as Wilkinson’s complex.

**Scheme 31:**
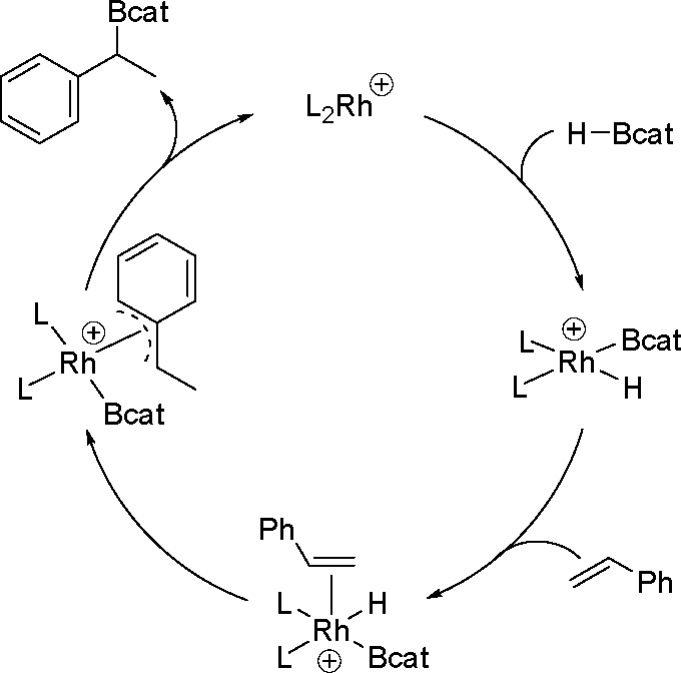
Mechanistic Rationale for the Cationic Rhodium-Catalyzed Hydroboration of Styrene Derivatives

Hayashi and Ito postulate that alkene insertion into the rhodium–hydride bond occurs regioselectively for styrene derivatives to place the rhodium center at the benzylic position, and a stabilized η^3^-benzylrhodium complex forms (see Scheme 31).^86^ Such a process is possible only for the cationic complex, which has a vacant coordination site that facilitates formation of the η^3^-benzyl complex. The corresponding neutral complex, which lacks such a site, is unable to form this complex, thus leading to anti-Markovnikov selectivity. Quite recently, an excellent review that summarizes the asymmetric methods for the introduction of boronic esters into organic molecules has been published.^87^

**Scheme 32:**
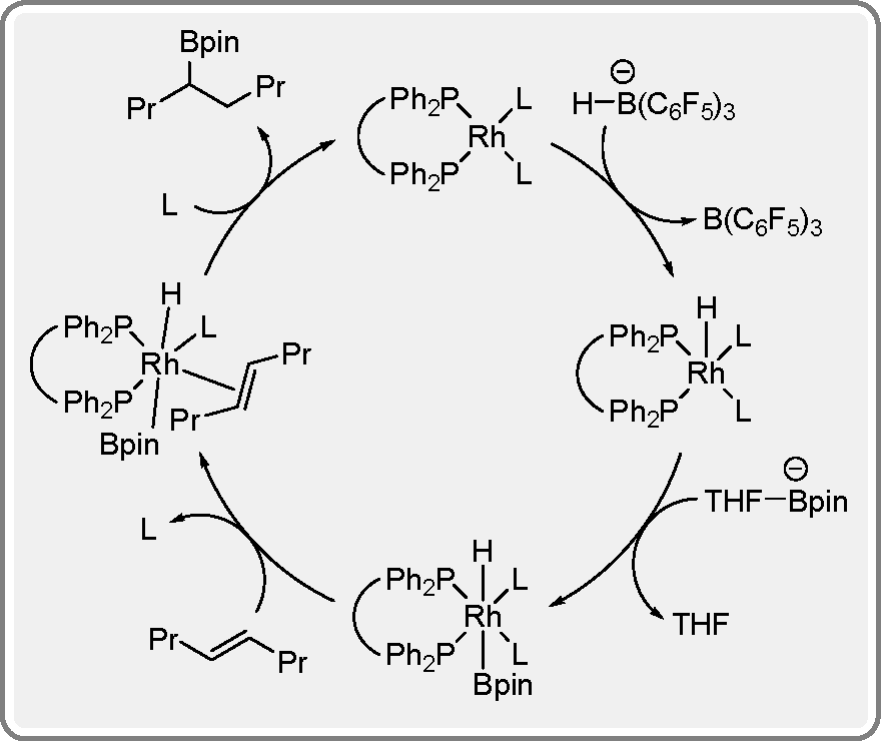
Catalytic Cycle for Tris(pentafluorophenyl)boron-Promoted Hydroboration Catalyzed by a Rh Complex

Apart from the influence of the type of catalyst on the course of reaction, it may also be changed by other factors. It has been found that the addition of Lewis acids significantly influences the rate, efficiency, and regioselectivity of the Rh-catalyzed hydroboration reaction. For example, the addition of (C_6_F_5_)_3_B to the reaction of vinylarenes (or aliphatic olefins) with HBpin, catalyzed by [Rh(cod)(dppb)][BF_4_]·THF, facilitates oxidative addition (as shown in Scheme 32), which increases the reaction rate and in some cases makes the reaction possible, which would not take place without the addition of a Lewis acid (for example, a reaction with *cis*-cyclooctene).^88^ The mechanism is based on the formation of HB(C_6_F_5_)_3_^–^ hydride by hydride transfer from HBpin to B(C_6_F_5_)_3_. However, it has been confirmed that it only occurs in the presence of the rhodium complex. Therefore, the transfer and hydride formation is not clear because it does not occur in the absence of the rhodium complex.

Initially, hydroboration reactions were mostly catalyzed by rhodium complexes, but interest in other metals, particularly first-row TMs, has increased significantly over time.^89^ Unfortunately, these metals often promote the course of side processes, which significantly affects the efficiency of the actual process. In order to limit this phenomenon, it is important to select an appropriate metal–ligand system. Well-designed and properly selected ligands for a given metal make it possible to obtain an efficient and selective catalytic system.^90^ Over the last 5 years, many articles in this field have appeared, and the conducted research has allowed the creation of a database of effective complexes, which are a combination of a metal and a specially designed ligand. A few reviews were published in 2018–2019 in which the general (asymmetric) hydrofunctionalization of alkenes and alkynes catalyzed by earth-abundant metals^21,24,25,91−98^ and lanthanide and actinide complexes^99^ was discussed. An excellent review recently presented complexes of elements from the first row of d-block as catalysts for the formation of a carbon–boron bond.^100^ Diverse main group metal catalysts, such as Li,^101^ K,^102^ Mg,^103^ and Al,^104,105^ and other TM catalysts, such as Pd,^106^ Ir,^107^ Ag,^108^ and Zn,^109^ were also designed to efficiently and selectively promote the hydroboration of unsaturated bonds.

**Scheme 33:**
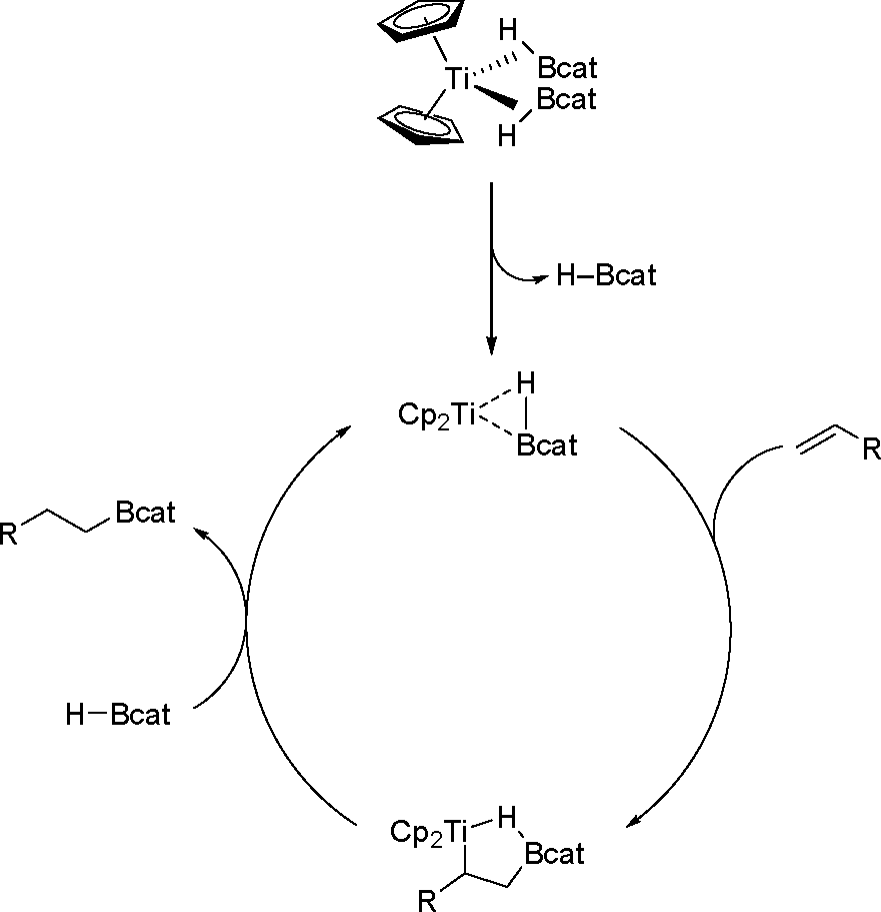
Catalytic Cycle for Hydroboration Catalyzed by a Ti Complex

In most cases, the hydroboration of alkenes follows the mechanism proposed for Rh complexes. However, the hydroborating agent sometimes plays an additional role. For example, in the reaction catalyzed by a titanium complex, HBcat is the ligand in the initial state of the catalyst (see Scheme 33). The mechanism has been studied by computational methods. Initial dissociation generates an intermediate that reacts with the olefin to give a five-membered intermediate. In the last step, the addition product is formed by reductive elimination. A less-hindered α-position of the R group to the Cp_2_Ti group determines reaction regioselectivity.^110^

**Scheme 34:**
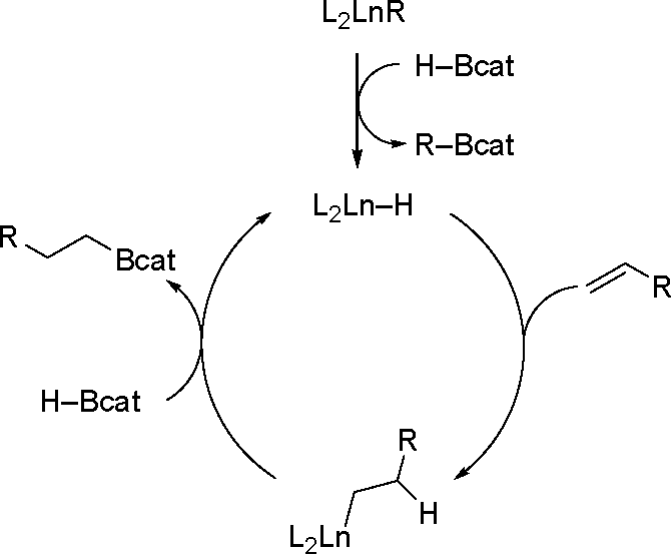
Proposed Mechanism of Organolanthanide-Catalyzed Olefin Hydroboration

For the reactions catalyzed by lanthanide complexes, Marks and co-workers^111^ have proposed a completely different mechanism (Scheme 34). The entire catalytic cycle takes place without any changes in the oxidation state of the central metal. In the first activation step, protonolysis of the lanthanide alkyl catalyst with HBcat generates a lanthanide hydride (the active species), followed by olefin insertion in an anti-Markovnikov manner to form a complex, which gives the final product with another HBcat molecule.

Since the mechanism is significantly different, it is not surprising that the regioselectivity and scope are also distinct. Unlike the reaction catalyzed by Rh, the reaction with vinylarenes gives predominantly the linear isomer. Most remarkably, trisubstituted olefins can be hydroborated with high yield.

**Scheme 35:**
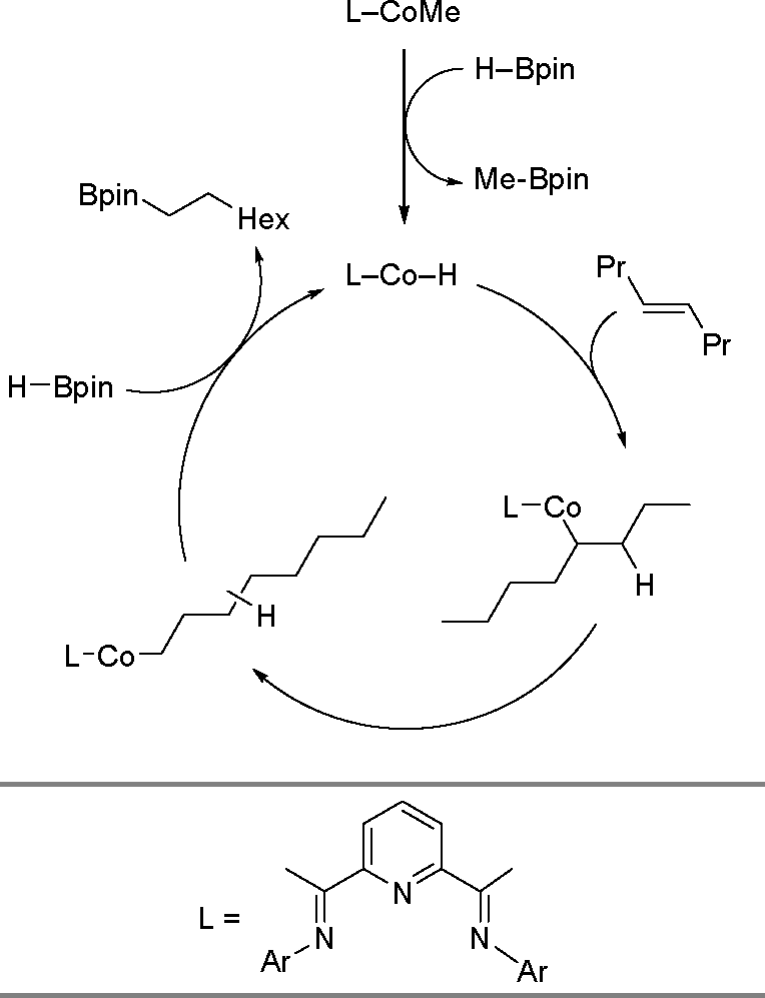
Cobalt-Catalyzed Isomerization-Hydroboration of Alkenes

During hydroboration, internal alkenes can isomerize, and internally and terminally functionalized products could be obtained, which was previously possible only in the presence of precious metal catalysts.^88^ Recently, the first-row metal catalysts (especially Fe and Co) have also been reported as catalysts with high activity in isomerization–hydroboration reactions.^25^ Deuterium-labeling experiments using a Co precatalyst support the mechanism (see Scheme 35), in which an alkene reversibly inserts into the Co–H bond to give a secondary alkyl intermediate. Chain walking occurs through the β-H elimination step, and the two steps can be repeated to furnish a primary alkyl intermediate, which is intercepted by HBpin to yield a terminal alkylboronate ester.^112^

**Scheme 36:**
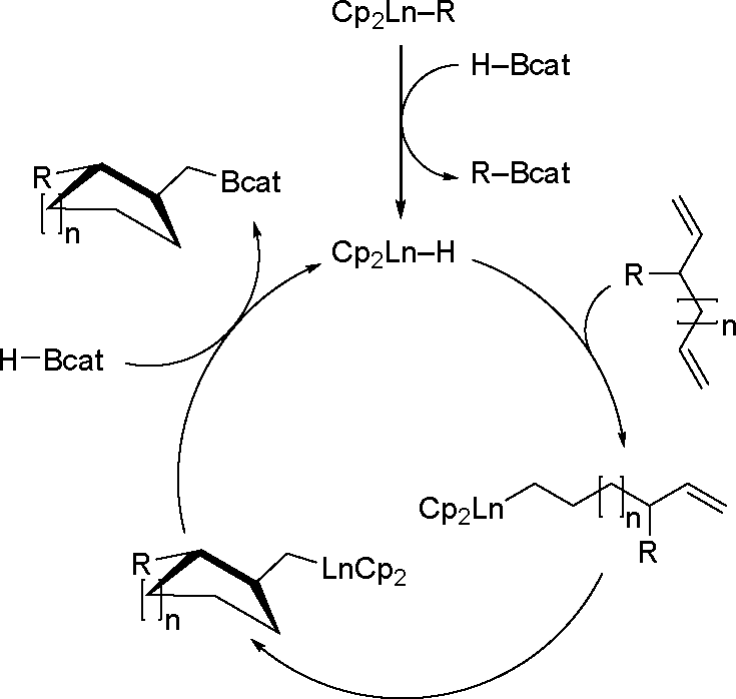
Proposed Mechanism for Organolanthanide-Catalyzed Cyclization Boration

This alkene isomerization–functionalization strategy has been applied in the conversion of internal alkenes into linear α-olefins. Hydroboration of monosubstituted cyclic alkenes (i.e., cyclohexenes or cyclooctenes) can proceed in the presence of Rh or Ir complexes in a diastereoselective manner to yield a 1,3-*anti*-isomer,^113^ but similarly directed hydroboration can also be effective in the presence of Fe and Co complexes.^25^ In the reaction with 1,5- or 1,6-dienes, lanthanide complexes catalyze the cyclization/boration reaction (Scheme 36).

**Scheme 37:**
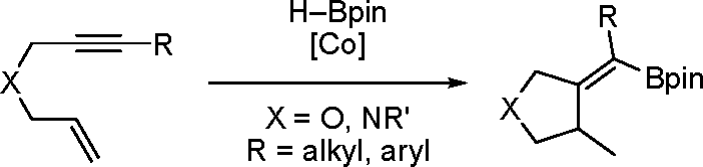
Hydroboration/Cyclization of 1,6-Enynes in the Presence of a Cobalt Complex

The first step is the same as in Scheme 34. The lanthanide hydride is then regioselectively inserted into one of the terminal double bonds to give a metal–hydrocarbyl compound. Subsequently, intramolecular cyclization between the metal species and another double bond, followed by reaction with another HBcat molecule, gives the desired cyclic product.^114^ In 2017, Lu reported the cobalt-catalyzed ligand-controlled regioselective hydroboration/cyclization of 1,6-enynes with HBpin (Scheme 37) that gave cyclic alkenylboronates.^115^

**Scheme 38:**
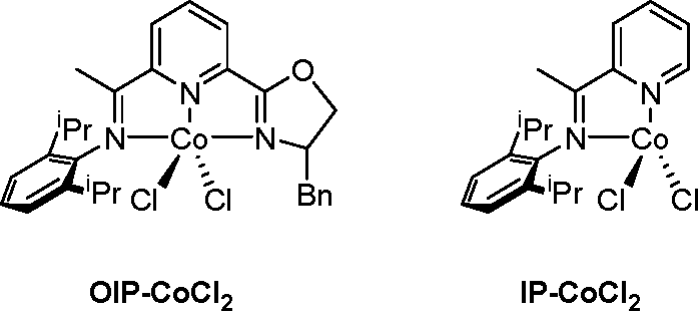
Two Cobalt Complexes with Different Ligands as Ligand-Controlled Catalysts

Different reaction products were obtained depending on the type of ligand present in the complex (Scheme 38).

When an OIP ligand was used, the product was mainly an alkylboronate. However, when a bidentate IP was used as the ligand, selective formation of alkenylboronate was observed.

**Scheme 39:**
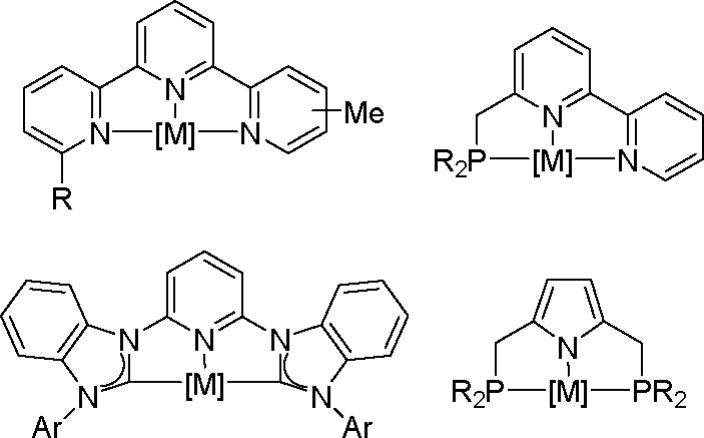
Examples of Tridentate Metal Complexes for the Hydrofunctionalization of Olefins

There has recently been a great interest in earth-abundant metals as potential chemo- and regioselective hydroboration catalysts. As in the hydrosilylation processes (see above), complexes containing tridentate ligands are of particular importance. Various complexes with NNN, PNN, CNC, and PNP ligands are known, as shown in Scheme 39.

Such ligands may stabilize low-valent metal centers by chelating effects, while leaving vacant coordination sites at the metal center for the bond activation of substrates. These types of complexes have been summarized in excellent review articles.^28,97,98^

**Scheme 40:**
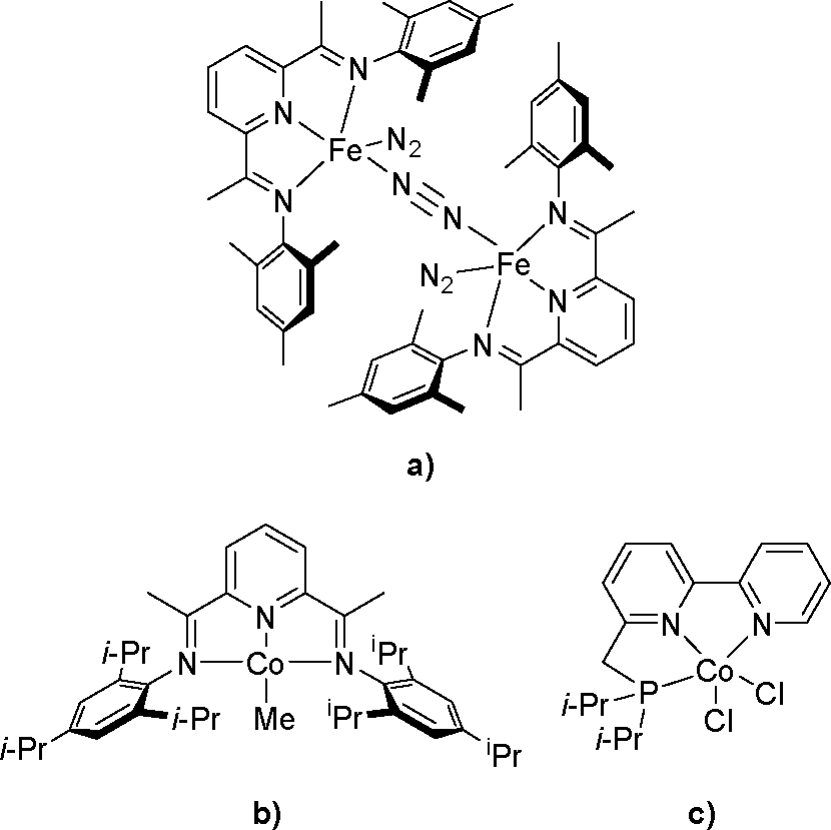
Some Types of Tridentate Iron and Cobalt Complexes as Efficient Hydroboration Catalysts

Presently, many examples of these types of complexes are known, mainly Fe, Co, and Cu, and the Chirik and Huang groups have made a large contribution to progress in this field. For example, dinitrogen complexes (Scheme 40a) enabled the chemo- and regioselective hydroboration of monosubstituted, 1,1-disubstituted and 1,2-disubstituted alkenes without the need for an organic solvent, an external activator, or excess alkenes and afforded anti-Markovnikov products in high yield.^25^

In comparison with the above complex, an analogous cobalt complex (Scheme 40b) was easier to prepare and showed comparable or improved activity in the hydroboration of terminal alkenes and disubstituted internal alkenes.^112^ In turn, the cobalt complex (see Scheme 40c) catalyzed anti-Markovnikov hydroboration that occurred exclusively for various aryl and aliphatic alkenes.

**Scheme 41:**
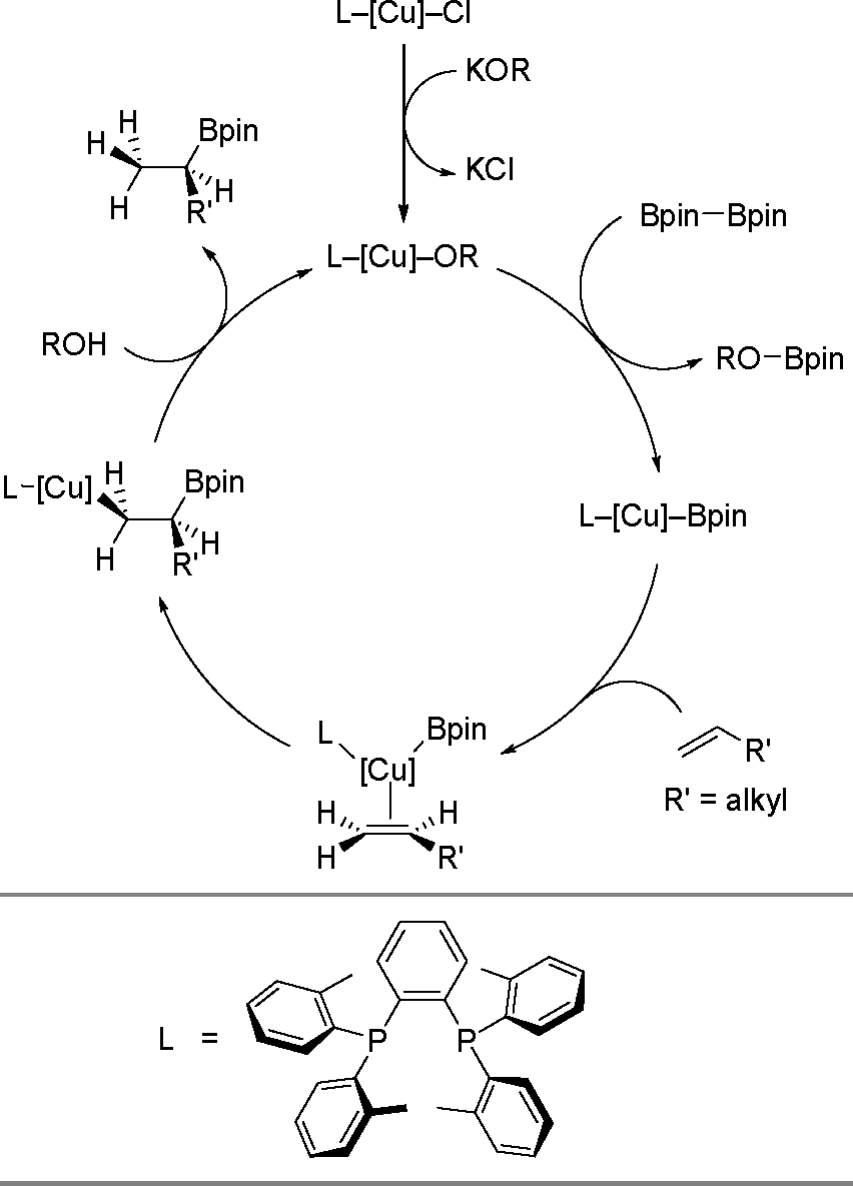
Plausible Catalytic Cycle of the Copper(I)-Catalyzed Branched-Selective Hydroboration of Aliphatic Alkenes

Although several tridentate (pincer) earth-abundant metal complexes have been developed for catalytic alkene hydroboration, only a few of them could provide high selectivity for Markovnikov addition (branched-selective hydroboration).^116^ As discussed above, the rhodium cationic complex selectively catalyzes Markovnikov addition. However, branched-selective hydroboration is limited to vinylarene substrates (also in the case of earth-abundant metal catalysts), except for several Cu catalysts.^117−119^ The first copper(I)-catalyzed highly branched-selective hydroboration of alkyl-substituted terminal alkenes was achieved by the Ito group.^117^ In this case, B_2_pin_2_ instead of HBpin had to be used as the boron source, and alcohol was required for providing the proton (Scheme 41).

In this scheme, a combination of CuCl with a sterically hindered bisphosphine ligand is used to promote the hydroboration of aliphatic terminal alkenes.

In the hydroboration of alkynes, significant differences in the course of the reaction are found depending on the type of catalyst. Many TM catalysts are known to convert terminal alkynes into (*E*)-vinylboronate esters, perhaps because of the prevalence of metal hydrides and the facile nature of alkyne hydrometalation.^120^ In 2015, Chirik and co-workers^121^ developed catalytic hydroboration of terminal alkynes with HBpin, using a cobalt catalyst. This complex can produce (*Z*)-vinylboronates with regio- and stereoselectivity.^121^

**Scheme 42:**
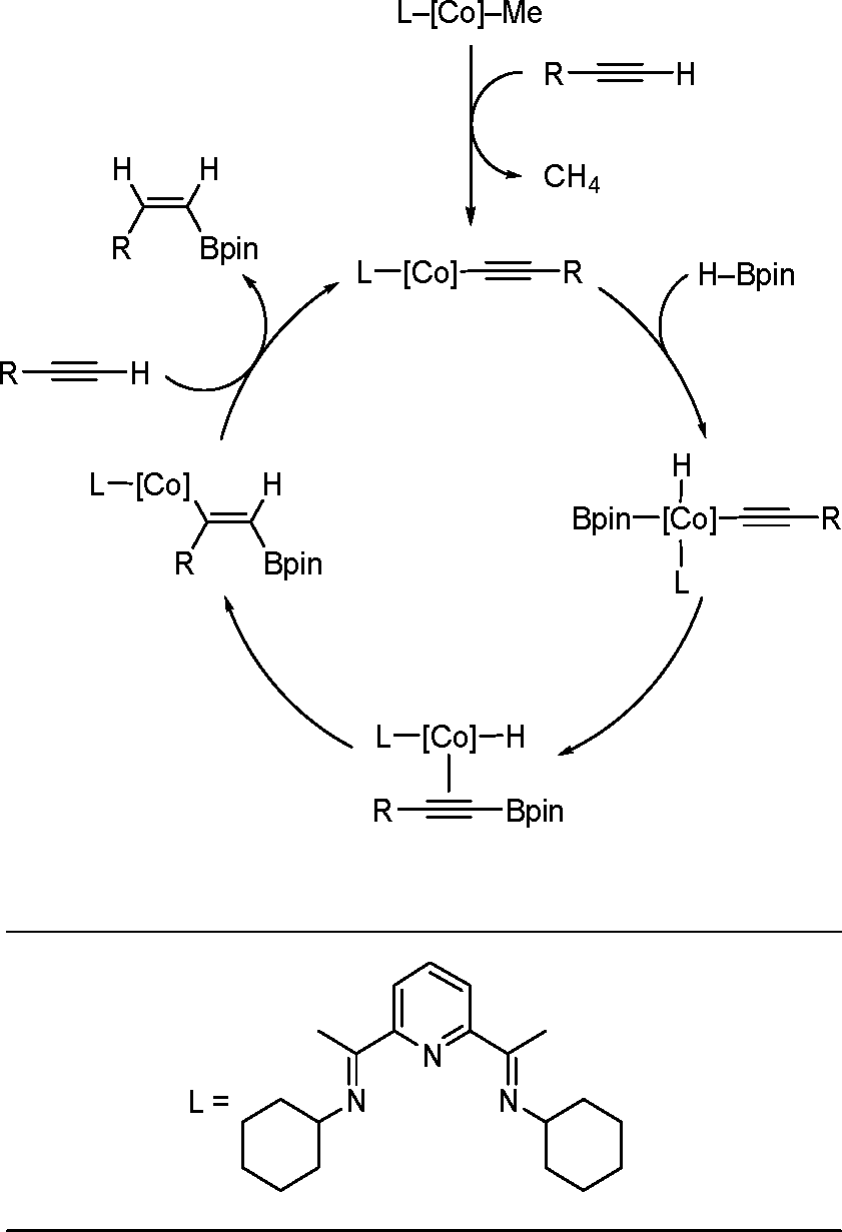
An NNN-Pincer-Cobalt-Catalyzed Hydroboration of Terminal Alkynes

The process starts with the reaction of a cobalt–methyl complex with an alkyne to form a cobalt acetylide species (see Scheme 42). Following the oxidative addition of HBpin to the cobalt center, reductive elimination occurs to give a cobalt–alkynylboronate complex. Subsequently, *syn*-hydrocobaltation generates a (*Z*)-cobalt–vinyl intermediate, which reacts with alkyne to release the corresponding product and to regenerate the starting complex. In principle, the mechanism can give a single product. The observed (*Z*)-selectivity is proposed to occur through the stereodefined *syn*-hydrometalation of the alkynylboronate–a mechanism distinct from those mediated by precious metal catalysts.

In a related work, (Z)-selective terminal alkyne hydroboration was also observed when [(PNP-pincer)Fe(H)_2_(H_2_)] precatalysts were used.^122^

**Scheme 43:**
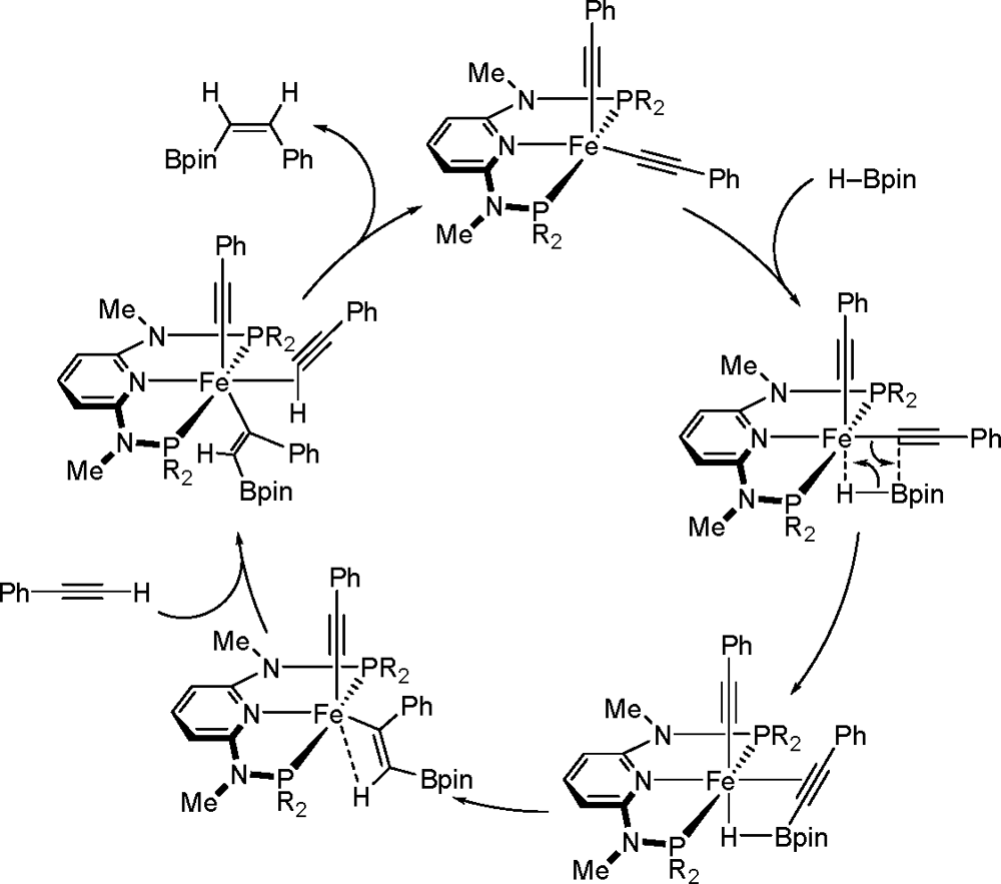
Catalytic Cycle for the Hydroboration of Terminal Alkynes Catalyzed by a PNP-Pincer–Iron Complex

Kirchner and co-workers proposed another reaction mechanism for the Z-selective hydroboration of terminal alkynes.^123^ The pincer complex [Fe(PNP)(C≡CPh)_2_] was used as an active catalyst in the hydroboration reaction. In this case, the hydroboration reaction did not involve vinylidene intermediate formation but rather proceeded via a σ-bond metathesis/hydrometalation reaction sequence (Scheme 43).

In the first step, the borane closely binds to the catalyst, giving an intermediate with two alkynyl ligands in the *cis* position to each other. The H atom is attached to the iron center acting as an electron donor, while boron is attached to a neighboring acetylide α-carbon acting as an electron acceptor. This initiates C–B bond formation. The B–H bond, which is still present, is cleaved and thus permits alkyne insertion into the iron–hydride bond to finally yield an iron–vinyl intermediate. The elimination of the final product and the recovery of the initial bis(acetylide) complex is accomplished via proton transfer from another alkyne. The mechanism was presented on the basis of experimental results (species and intermediates were trapped and fully characterized spectroscopically) and based on DFT calculations.

**Scheme 44:**
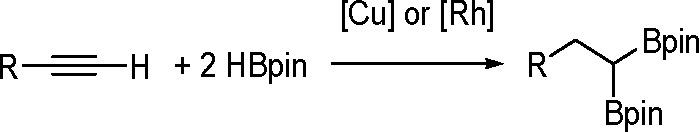
Sequential Hydroboration of Alkynes Catalyzed by Rh or Cu Complexes

The catalytic sequential hydroboration of terminal alkynes is a synthetically useful approach to the generation of 1,1-diboronates. However, sequential and regioselective hydroborations of alkenylboronate intermediates are rare, and most reactions generate a mixture of regioisomers. There are examples of Rh and Cu complexes used as catalysts for the sequential hydroboration of alkynes with HBpin to produce double hydroboration products (Scheme 44).

**Scheme 45:**
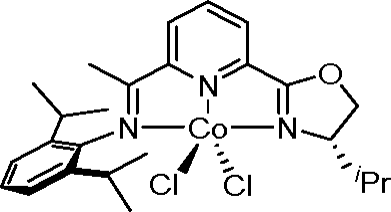
NNN-Pincer–Cobalt Complex as an Effective Catalyst for the Hydroboration of Alkynes

An iminopyridine oxazoline-ligated cobalt complex is one of the most effective catalysts (Scheme 45).

This catalyst offers high conversion and regioselectivity, broad functional group tolerance, and transformation under mild reaction conditions.^124^

Although alkenylboronate esters are traditionally prepared by the hydroboration of alkynes, this method can be complicated by regioselectivity issues and gives a mixture of isomers. In some cases, the addition of a second equivalent of borane to the activated alkenylboronates once again leads to a mixture of saturated bisboron-containing alkanes. The dehydrogenative borylation reaction is an alternative method for synthesizing alkenylboronates.^125^ Today, many different TM complexes (i.e., Rh, Pd, Ir, Ru, Fe, Co, Ti) are known to be effective catalysts of this process. This aspect has been thoroughly summarized in ref (85).

**Scheme 46:**
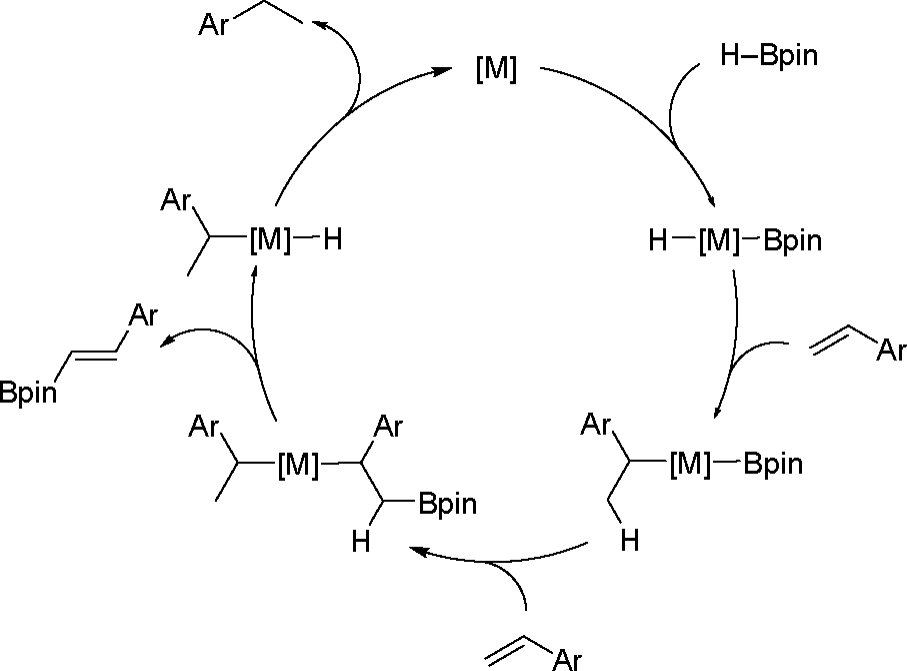
General Mechanism for Dehydrogenative Borylation

**Scheme 47:**
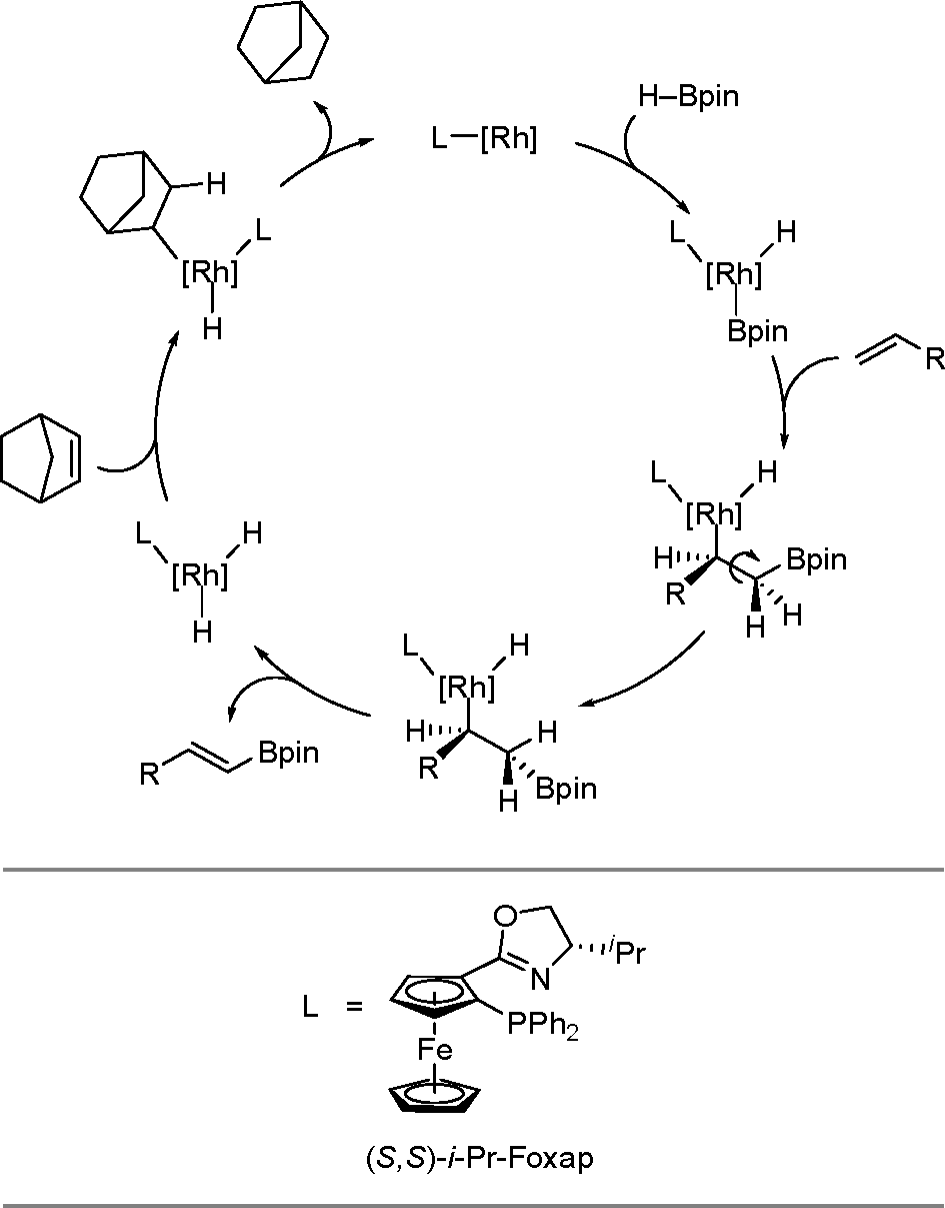
Plausible Catalytic Cycle for the Rhodium-Catalyzed Dehydrogenative Borylation of Alkenes in the Presence of Norbornene

The key to the success of this reaction is to use a metal system capable of alkene insertion into an M–B bond (followed by β-hydride elimination) while avoiding metal hydride and dihydrido species that are capable of forming hydroboration and hydrogenation products, respectively (see Scheme 46). This catalytic reaction provides an attractive method for generating valuable alkenylboronate esters. The group of Miura and Murakami^126^ reported the first general dehydrogenative borylation of aryl and aliphatic alkenes using [Rh(cod)_2_][BF_4_]/*i*-Pr-Foxap as a catalyst (Scheme 47).

Norbornene was used as the H_2_ acceptor as it was surmised that its strained structure would be reduced in preference to the alkene substrate. Oxidative addition of B–H onto rhodium(I) affords a boryl(hydrido)rhodium species which undergoes alkene insertion to give an alkyl–rhodium intermediate. This intermediate subsequently rotates around the C–C bond axis to form a conformer, which gives the E-isomer of the vinylbornate ester after *syn* β-H elimination. The dihydride rhodium species reacts with norbornene to regenerate the rhodium(I) catalyst, together with norbornane.

**Scheme 48:**
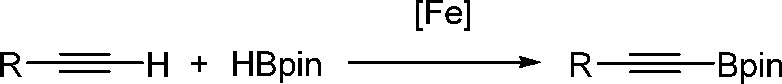
Dehydrogenative Borylation of Terminal Alkynes Catalyzed by an Iron Complex

Recently, a catalytic system based on an iron complex (Fe(OTf)_2_ with DABCO) that selectively promotes the dehydrogenative borylation of both aromatic and aliphatic terminal alkynes has been reported (see Scheme 48) to generate alkynylboronate derivatives.^127^

This method is applicable to a variety of terminal alkynes. Very recently, iridium complexes with diarylamido/bis(phosphine) PNP ligands have also been studied using DFT calculations as catalysts for the dehydrogenative borylation of terminal alkynes.^124^

#### 1.5.2 Hydroboration of Carbonyl Compounds

Hydroboration of carbonyl compounds is an efficient strategy for accessing primary and secondary alcohols. It is especially useful for the preparation of a variety of chiral alcohols that are widely used in organic synthesis, materials science, and pharmaceutical chemistry.^129,130^ The hydroboration of carbonyl compounds using both homogeneous and heterogeneous catalyst systems has been addressed in excellent reviews. Kinjo demonstrated TM-catalyzed hydroboration of unsaturated bonds, such as C=O and C=N, and investigated its mechanism.^131^ A recent review by Bose summarizes the developments in the hydroboration of C=O bonds via s- and p-block elements and TMs (including precious and nonprecious metals and lanthanides) as efficient catalysts, as well as heterogeneous catalyst systems.^132^

**Scheme 49:**
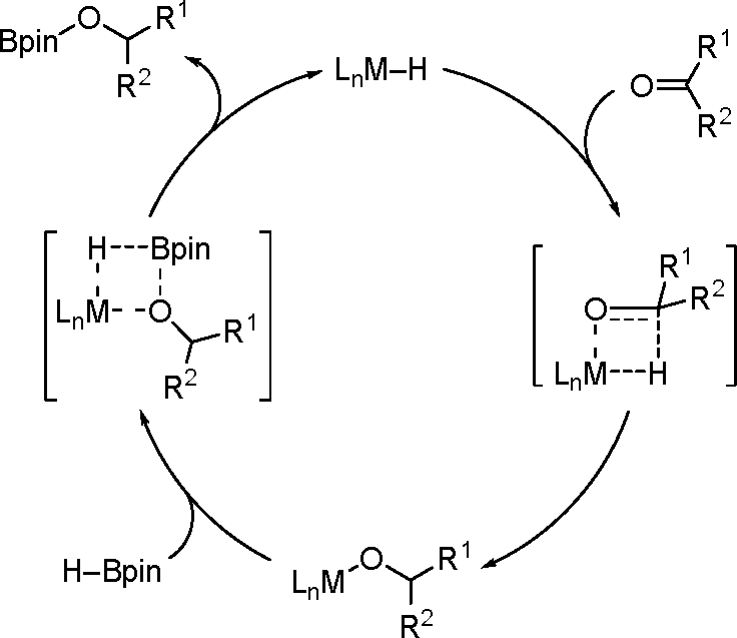
General Mechanism for the TM-Catalyzed Hydroboration of Carbonyl Compounds

In the majority of the proposed mechanisms, the transformation proceeds via a σ-bond metathesis in reactions catalyzed by TMs or main group metals (Scheme 49).

All the studies of this mechanism imply generation of M–H intermediate species, and this might be the key step in the hydroboration of aldehydes and ketones.

In 2015, Gunanathan group reported a ruthenium [{Ru(p-cymene)Cl_2_}_2_] complex as an efficient catalyst of the hydroboration of various aromatic and aliphatic aldehydes with electron-withdrawing and electron-donating groups.^133^ The proposed mechanism of this reaction type is different from the one described above.

**Scheme 50:**
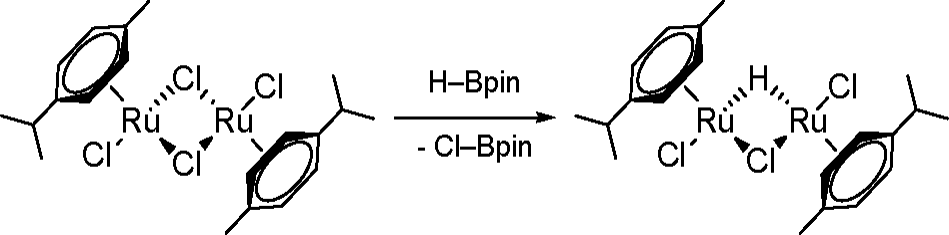
Formation of a Monohydrido-Bridged Dinuclear Ruthenium Complex

The reaction of the starting complex with HBpin gave a monohydrido-bridged dinuclear ruthenium complex, [{(η^6^-p-cymene)RuCl}_2_(μ-H-μ-Cl)] (Scheme 50). This complex was prepared and spectroscopically characterized.

**Scheme 51:**
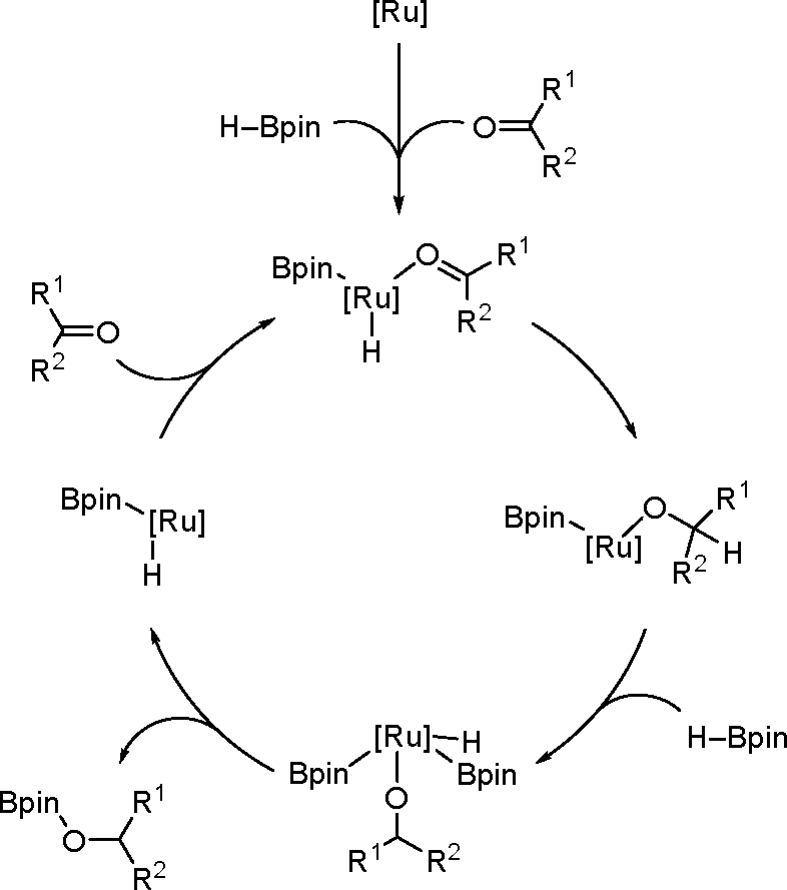
Catalytic Cycle for the Ruthenium-Catalyzed Hydroboration of Ketones

This complex leads to the formation of an active intermediate in a reaction with the next HBpin molecule in the presence of a carbonyl, according to Scheme 51.

In the first step, the hydride is transferred from the metal to the carbonyl group, followed by the oxidative addition of HBpin, which increases the oxidation state and the coordination number of ruthenium. In the final stage, the product is reductively eliminated, and the starting catalyst is reconstituted in the presence of the raw material.

#### 1.5.3 Double Hydroboration of Carbon–Nitrogen Triple Bonds

**Scheme 52:**
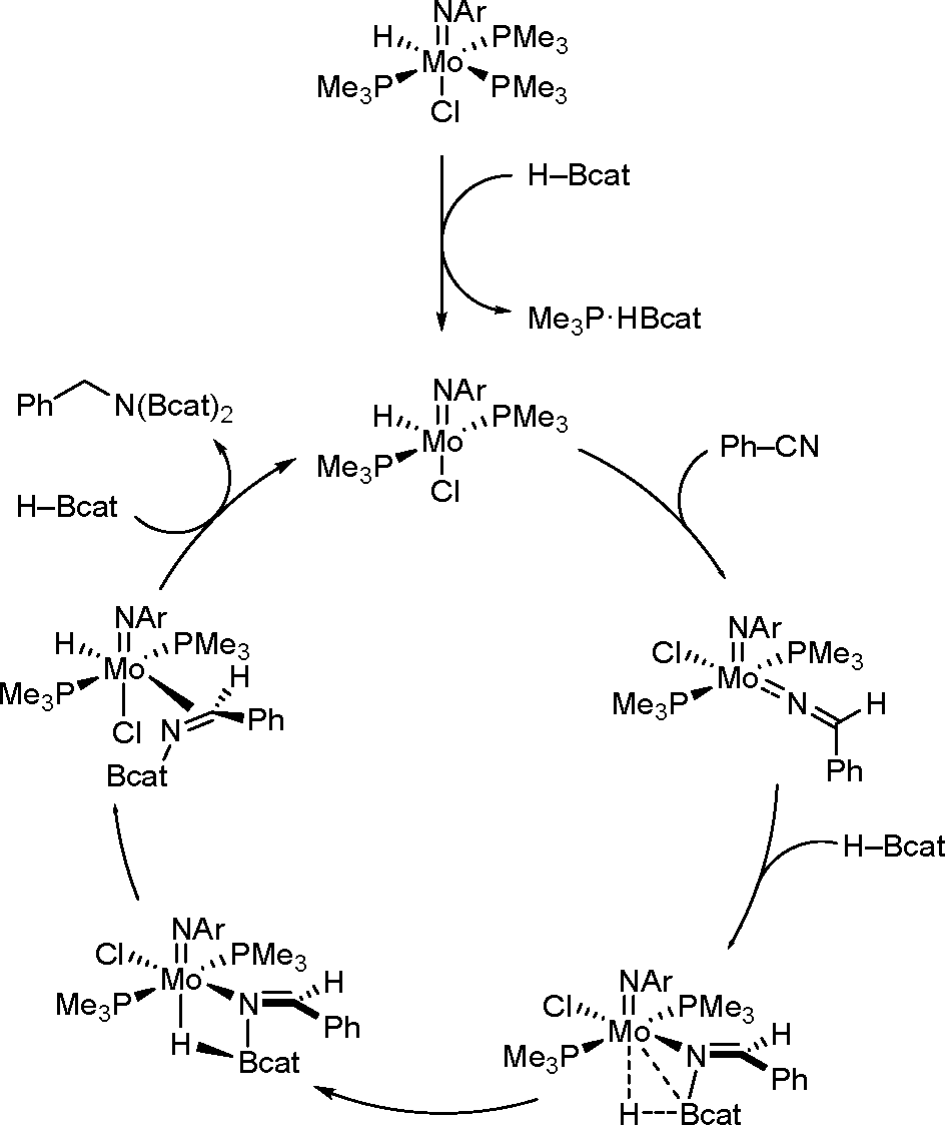
Reaction Pathway of the Double Hydroboration of Nitriles Catalyzed by an Imido–Hydrido Mo(IV) Complex

Borylamines have been reported to show unique reactivity as iminum ion generators. Hydroboration of the C≡N bond is also one of the effective methods for reducing organonitriles. However, the reaction does not occur under typical hydroborylation conditions because of the high dissociation energy of the strong C≡N bond. At present, only a few examples of the catalytic hydroboration of carbon–nitrogen triple bonds are known (presented in ref (134)). The imido–hydrido Mo(IV) complex was one of the first examples of an active double hydroboration catalyst, presented by Nikonov and Khalimon groups (see Scheme 52).^135^

As a result of the elimination of the phosphine molecule with HBcat, the Mo(IV) complex is formed, which initiates the catalytic cycle. The addition of benzonitrile followed by HBcat produces an amido-borane adduct is formed, which is finally converted to a borylimine complex. In the final stage, reductive elimination of diborylamine occurs and the active Mo(IV) complex is restored.

**Scheme 53:**
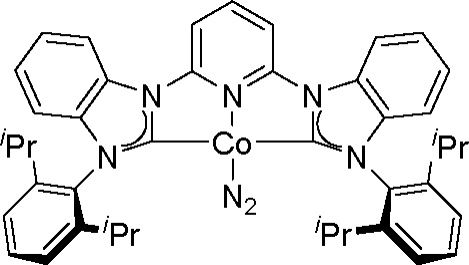
Cobalt(I) Complex as a Catalyst of the Double Hydroboration of Nitriles

In 2017, the Fout group reported the double hydroborylation of organonitriles catalyzed by a Co(I) complex^136^ (Scheme 53).

**Scheme 54:**
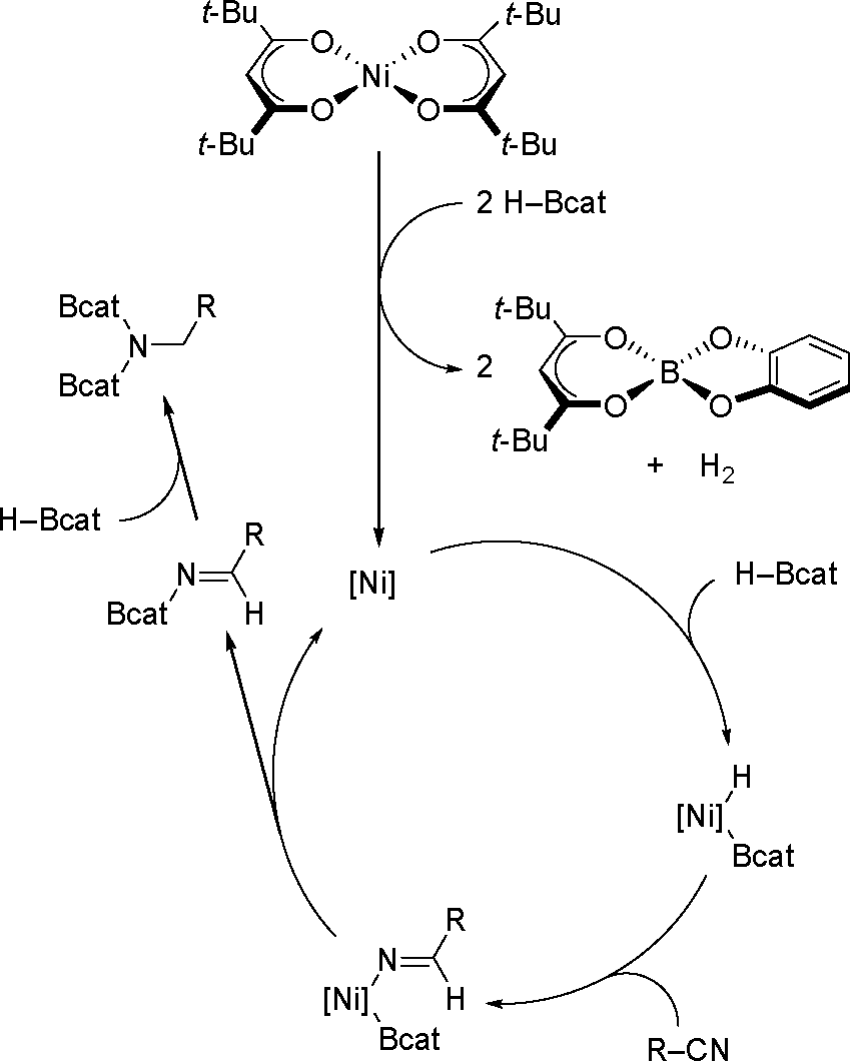
Proposed Mechanism for the Ni-Catalyzed Double Hydroboration of Nitriles

An interesting example of using a simple nickel salt (a nickel–acetylacetonate derivative) as an active double hydroboration catalyst has been presented by Nakajima and Shimada groups.^137^ (see Scheme 54).

Reduction of the starting complex by 2 equiv of HBcat produces an active Ni(0) species. The elimination product was cofirmed by NMR spectroscopy as well as by single crystal X-ray diffraction analysis. Oxidative addition of HBcat to the Ni(0) center gives a boryl–hydrido intermediate. The nitrile insertion into the Ni–H bond, followed by the subsequent reductive elimination of borylimine, regenerates the active catalyst. The resulting borylimine further reacts with HBcat to give *N*,*N*-diborylamine. The proposed mechanism of this reaction is a typical hydroboration reaction mechanism. The distinguishing feature of this reaction, however, is the use of a very cheap commercially available catalyst that can successfully replace precious metal complexes.

Several other effective catalytic systems based on other metals, such as Ru, Mg, Co, and Fe, are currently known,^134^ which demonstrates the great interest in the possibility of selective double hydroboration. A titanium(IV) complex [{Ph_2_P(BH_3_)-N}_2_C_6_H_4_Ti(NMe_2_)_2_] is one of the latest examples of this reaction type and it enables chemoselective hydroboration of organic nitriles to obtain *N*,*N*-diborylamines with a wide substrate range, containing both aliphatic and aromatic nitriles.^138^

### 1.6 General Mechanism for Hydrometalation

**Scheme 55:**
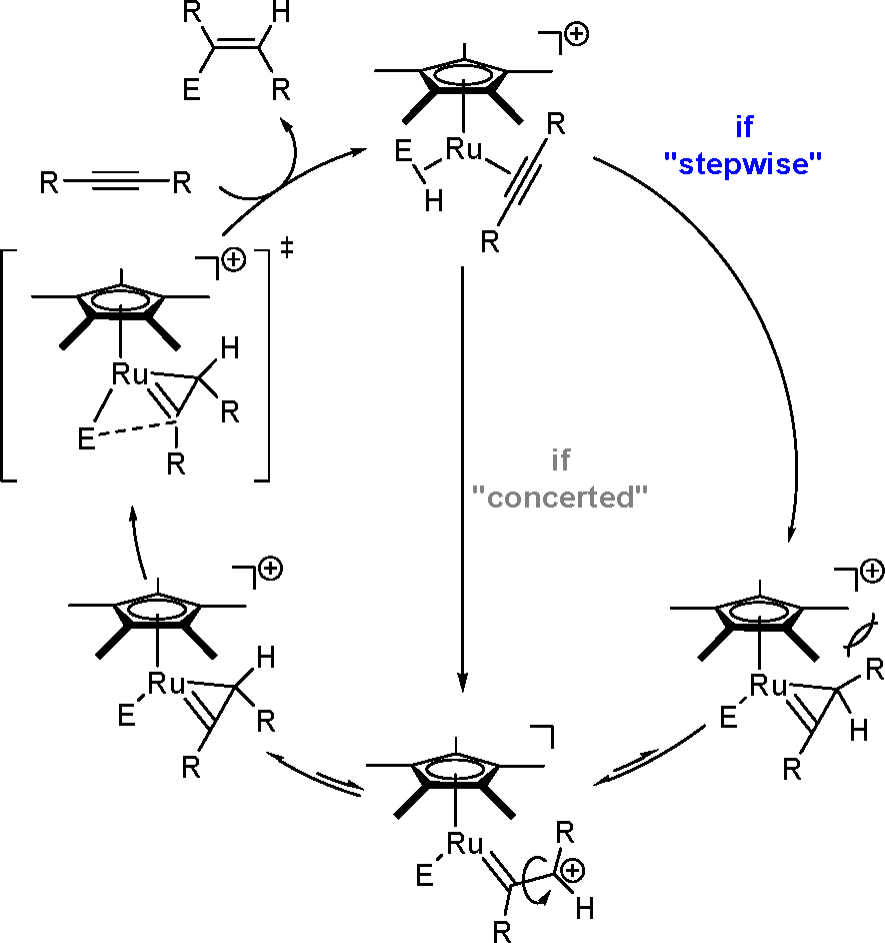
Putative Mechanism of the Hydrometallation of Alkynes (E = H, (RO)_2_B, R_3_Si, R_3_Ge, R_3_Sn)

On the basis of a comprehensive study by Fürstner of the catalysis of hydrometalation with ruthenium complexes, a general mechanism of the *trans* addition of E–H bonds to triple bonds has been presented^139^ (see Scheme 55). Thus, the mechanistic scheme above may provide excellent preliminary evidence for the general role of the TM–metalloid catalytic system in inorganometallic chemistry.

## 2 Addition of Metalloid–Metalloid Bonds to Carbon–Carbon Multiple Bonds

### 2.1 Mechanistic Aspects of Bis-Metalations

Since the early 1990s, the TM-catalyzed insertion of a bond between two elements of the *p*-block (Si–Si, Si–Sn, Sn–Sn, B–B, Si–B, etc.) into carbon–carbon multiple bonds (alkenes, alkynes, and allenes) has drawn significant attention because these additions provide promising methods for the introduction of metalloids into organic molecules in a regio- and stereoselective manner.^56,140−145^ Significant progress in this area in the past two decades has been made possible by the application of new effective catalysts, deeper understanding of the reaction mechanisms, and introduction of selective chiral ligands so that bis-metalation chemistry can be used for enantioselective synthesis.^146^

A variety of *p*-block element σ-bonds can be added to unsaturated organic molecules owing to TM catalysts, mostly group 10 TM complexes (commonly phosphine Pd(0) and Pt(0) complexes); however, other complexes containing rhodium or ruthenium could also be employed as bis-metalation catalysts. On the other hand there has been much interest in the promising catalysis of base (Fe, Co, Ni) and coinage (Cu, Au) metals for the bis-metalation of C–C multiple bonds.

**Scheme 56:**
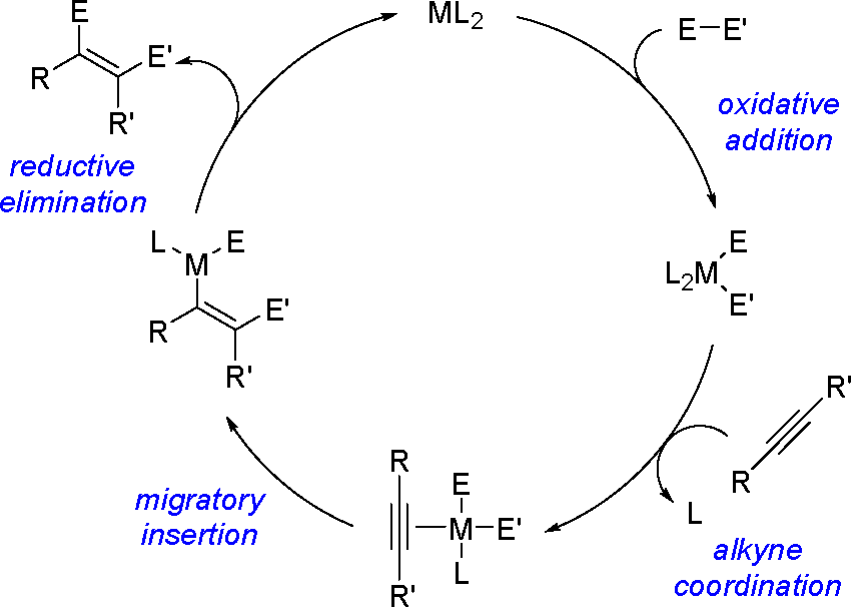
General Mechanism of Alkyne Bis-Metalation

It is widely accepted that inorganometallic complexes containing TM–metalloid (TM–E) bonds are the key intermediates of catalytic bis-metalation. The general mechanism of bis-metalation involves oxidative addition of the E–E σ-bond into the TM complex, which gives a bis-metalated TM complex, insertion of the C–C multiple bond into the TM–E bond and reductive elimination of the bis-metalated compound with regeneration of the catalyst (Scheme 56). The regioselectivity of the E–E addition is usually defined by the insertion of the alkyne (alkene), and the determining factors include the energy of the bonds broken versus that of the bonds formed, steric effects of the system, and electronic stabilization effects within the resulting intermediates. However, experimental studies suggest that the insertion is often the rate-limiting step in these reaction pathways.^146^

**Scheme 57:**
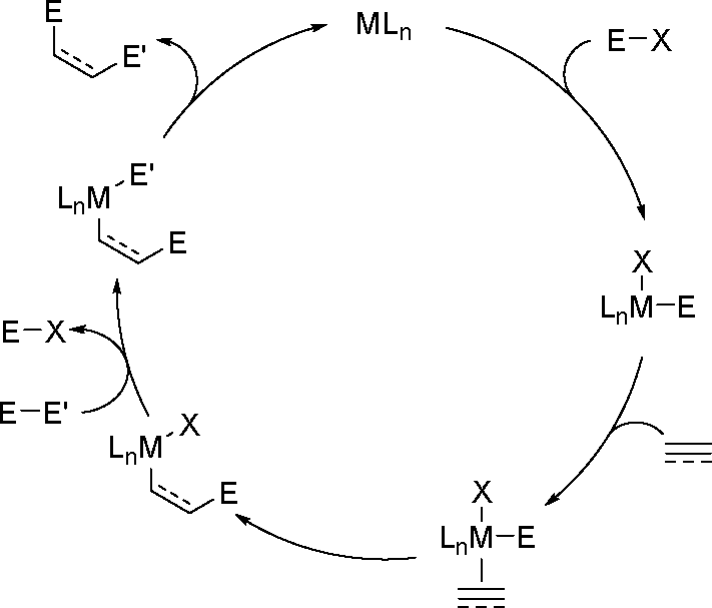
Alternative Mechanism of Three-Component Bis-Boration and Silaboration

Alternatively, bis-metalation may occur without oxidative addition of the E–E reagent. As already determined for a palladium-catalyzed allene bis-boration and silaboration (see below) these reactions can be initiated by the oxidative addition of a metalloid halide (E–X) to a TM complex. Allene insertion is followed by transmetalation with the E–E-bonded reagent. This step regenerates the metalloid halide and provides an intermediate for product release (Scheme 57). Thus, phosphine-free palladium complexes together with alkenyl or aryl iodides are very efficient catalysts for the 1,2-diboration of allenes. This Pd-catalyzed reaction proceeds via a mechanism involving the oxidative addition of an I–B bond to the palladium center instead of the oxidative addition of a B–B bond to a metal.^147^ A similar mechanistic pathway involving a palladium-catalyzed three-component assembling reaction of iodosilane, allene, and borylsilane has been proposed to account for the silaboration reaction.^148^

The mechanistic implications of the bis-metalation processes have been explored mostly for platinum and palladium complexes in alkyne bis-silylation, bis-boration, and silaboration (silylborylation) reactions. Therefore, we will focus mainly on alkyne bis-metalation reactions in this section, as proven by stoichiometric or computational studies.

### 2.2 Addition of Homoelement–Element Linkages to Unsaturated Substrates

**Scheme 58:**
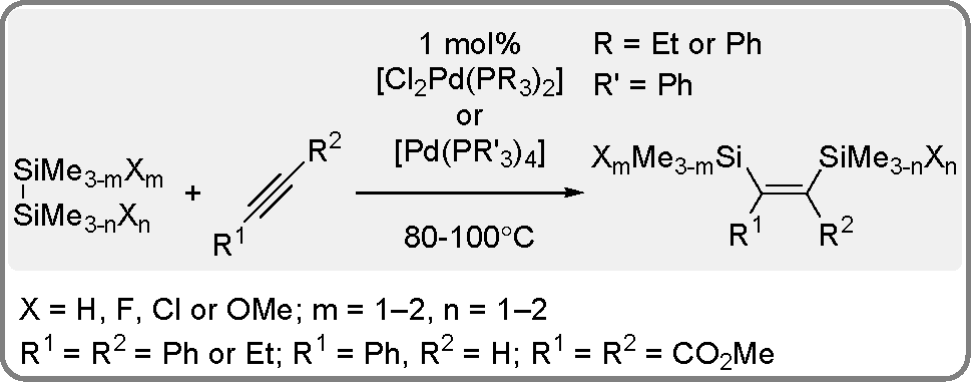
Bis-Silylation of Alkynes in the Presence of Palladium Catalysts

The catalytic addition of the Si–Si bond to alkynes (the first example of element–element additions to unsaturated compounds) has been developed since the 1970s.^149,150^ The addition of disilanes to terminal as well as internal alkynes catalyzed by TM complexes occurs with high *syn*-selectivity (Scheme 58). When the disilane reagents are nonsymmetric, the more electron-deficient silicon atom tends to be added to the internal position of alkynes.^151^ The majority of bis-silylation reactions are catalyzed by palladium complexes but also other types of catalysts based on platinum, rhodium, and nickel are able to catalyze the addition of disilanes to alkynes.

Since the Si–Si bond energy (74–80 kcal mol^–1^) is similar to that of the Si–H bond (88–92 kcal mol^–1^), it is reasonable to assume that the oxidative addition of a Si–Si bond would also be a facile process and would offer the potential for the formation of bis(silyl) TM complexes. An *ab initio* MO/MP4 study of the oxidative addition of SiH_4_ and H_3_Si–SiH_3_ to a [Pt(PPh_3_)_2_] complex showed that the addition of a Si–Si bond is more exothermic than that of a Si–H bond (46.4 and 25.8 kcal/mol, respectively), but the activation barrier is higher for bis-silylation (17.4 and 0.7 kcal/mol for Si–Si and Si–H, respectively).^152^ Therefore, the two reaction types can compete with the compounds that contain both types of bonds, with the actual result being dependent on the structure of the reagents and reaction conditions. It has been shown that the oxidative addition of H_3_Si–SiH_3_ to Pd(0) is less exothermic than that to Pt(0), although the former process has a slightly lower activation energy.^153^ Successful synthesis and isolation of bis(silyl)palladium,^154^ platinum,^155^ and nickel^156^ complexes via oxidative addition of the Si–Si bond have been reported.

**Scheme 59:**
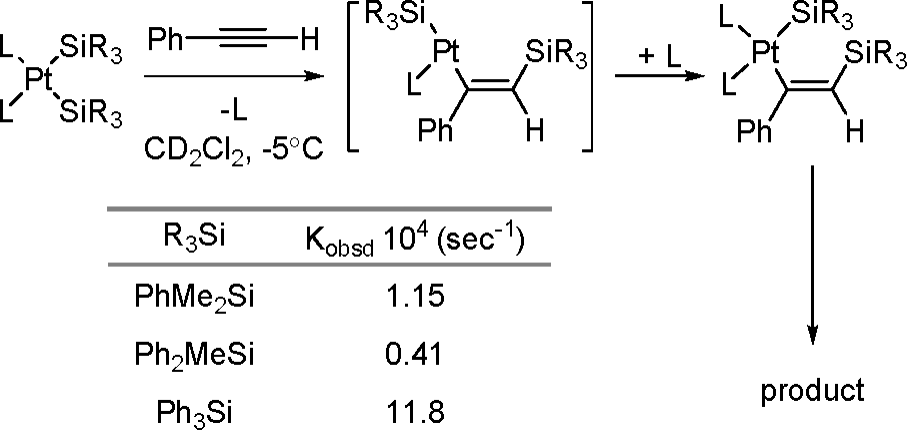
Insertion of Phenylacetylene into Pt–Si Bonds in Pt(II) Disilyl Complexes

The insertion of acetylenes into the Pt–Si bond has been studied by Ozawa and Hikida^157^ In the presence of excess phenylacetylene, pseudo-first-order rate constants (*k*_obsd_) were measured for the insertion of [Pt(PMe_2_Ph)_2_(SiR_3_)_2_] compounds into the Pt–Si bond (Scheme 59). However, the rate constants for the insertion of phenylacetylene into Pt–Si bonds in Pt(II)–disilyl complexes revealed a reactivity order that was inconsistent with the steric and electronic nature of the silyl substituents. A detailed kinetic investigation has shown that the first step is the dissociation of one of the phosphine (L) ligands to form an unsaturated complex which undergoes rate-determining migratory insertion via the prior coordination of acetylene to the vacant site.

The use of disilanes with electron-withdrawing substituents or strained cyclic disilanes is crucial for the formation of bis(silyl) TM complexes. However, there are two catalytic systems that allow bis-silylation with unreactive hexaalkyldisilanes. Ito et al. employed an efficient catalyst comprising Pd(OAc)_2_/1,1,3,3-tetramethylbutyl isocyanide,^158^ widely used for the intramolecular bis-silylation of disilanyl propargyl ethers. Tanaka and Yamashita discovered a catalytic system based on a combination of [Pd(dba)_2_] with a bicyclic phosphate: P(OCH_2_)_3_CEt (4-ethyl-2,6,7-trioxa-1-phospha-bicyclo[2.2.2]octane), applied in the synthesis of silicon-containing polymers and oligomers.^159^ More recently, the application of nonactivated hexamethyldisilane as a silylating agent using the [Pd(ITMe)_2_(SiMe_3_)_2_] complex as a catalyst (ITMe = 1,3,4,5-tetramethylimidazol-2-ylidene) has been demonstrated.^160^ A *cis*-[(ITMe)_2_Pd(SiMe_3_)_2_] complex as a result of the oxidative cleavage of Me_3_Si–SiMe_3_ with a [(ITMe)Pd(methallyl)Cl] precursor was isolated, which supported the catalytic studies.

**Scheme 60:**
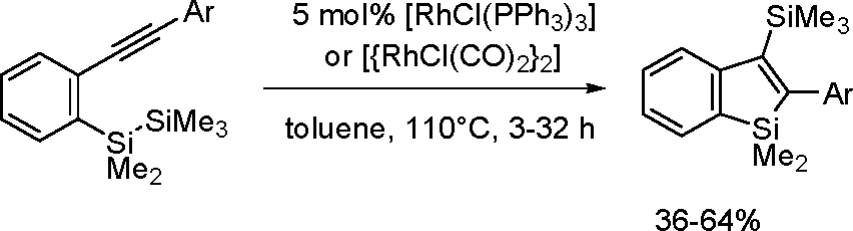
Rhodium(I)-Catalyzed Intramolecular Bis-Silylation of (2-Alkynylphenyl)-disilanes

Furthermore, the rhodium(I)-catalyzed intramolecular bis-silylation of disilanyl propargyl ethers or (2-alkynylphenyl)disilanes proceeded with a stereoselectivity opposite to that of the analogous palladium-catalyzed addition, and 4-silyl-2,5-dihydro-1,2-oxasilole or 3-silyl-1-benzosilole derivatives were obtained (Scheme 60).^161^

Alkene bis-silylation reactions seem to be less important because the use of bis(silyl)alkane products is limited. They are mainly carried out in the presence of platinum and palladium complexes. Me_3_Si–SiF_2_Ph has been found to undergo oxidative addition with an L_2_Pd(styrene) complex furnishing a *trans*-[Pd(SiMe_3_)(SiF_2_Ph)L_2_] complex (L = PMe_3_, PMe_2_Ph), which supports the classical oxidative addition/reductive elimination mechanism.^162^

**Scheme 61:**
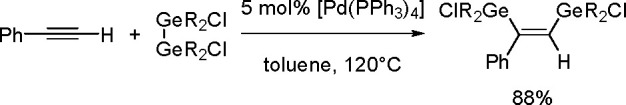
Bis-Germylation of Phenylacetylene with Dichlorotetraorganodigermane

In contrast to the insertion of alkynes into Si–Si and B–B bonds, the insertion of alkynes into Ge–Ge bonds has been investigated to a much lesser extent. The first additions of Ge–Ge bonds to triple bonds were reported by Ando and Tsumuraya who used a strained cyclic digermirane in the reaction with acetylene and dimethyl acetylene dicarboxylate in the presence of [Pd(PPh_3_)_4_], with moderate yields (20%–51%).^163^ Linear nonstrained dichlorotetramethyldigermane underwent effective double germylation with terminal alkynes in the presence of the [Pd(PPh_3_)_4_] or [Pd(dba)_2_]/P(OCH_2_)_3_CEt catalytic system (Scheme 61),^164^ whereas the more challenging hexamethyldigermane required a [Pt(acac)_2_] catalyst.^165^ However, a wide variety of aromatic and aliphatic terminal alkynes reacted quantitatively with nonactivated hexamethyldigermane in the presence of a heterogeneous catalyst (Au/TiO_2_) to form exclusively *cis*-1,2-digermylated alkenes with high yields (76–96%).^166^ As in the case of homogeneous catalysis, inorganometallic complexes containing Au–Ge bonds are postulated as key intermediates in this process.

**Scheme 62:**
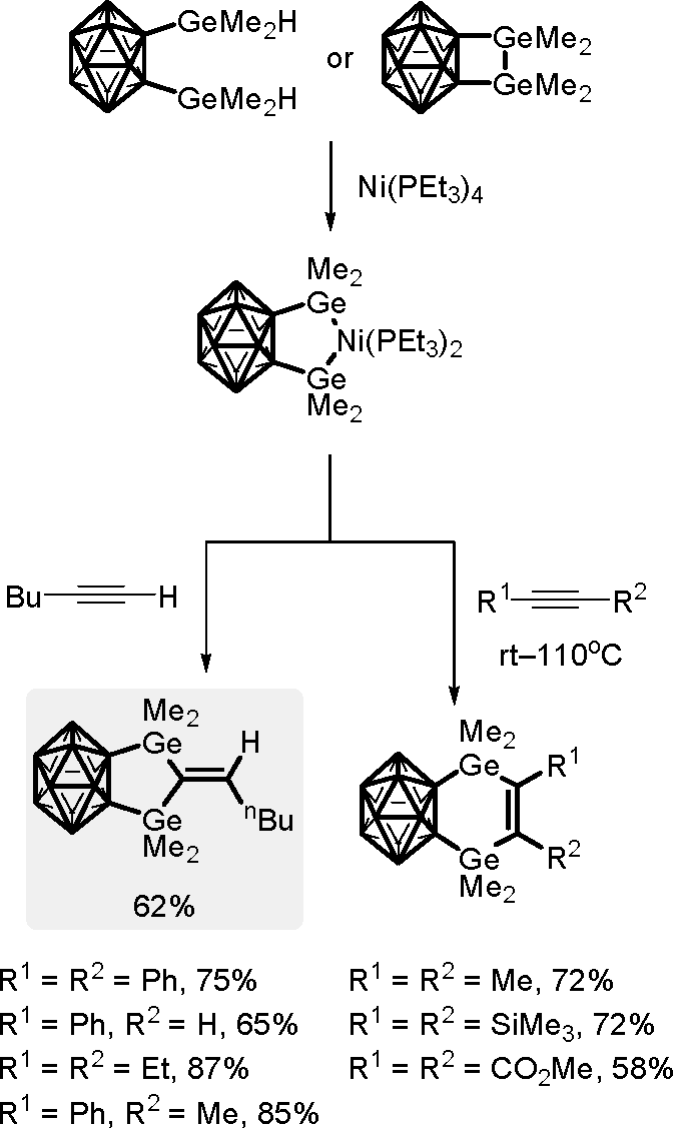
Stoichiometric Reaction of Alkynes with 1,2-Digermylcarborane and a Nickel(0) Complex

A stoichiometric study of alkyne bis-germylation has been performed using 1,2-digermylcarborane and a nickel(0) complex. A digermyl–nickel(II) complex, generated by the oxidative addition of 3,4-carboranylene-1,1,2,2-tetramethyl-1,2-digermacyclobutane to tetrakis(triethylphosphine)nickel(0) reacted with internal alkynes to give *cis*-addition products. In contrast, the stoichiometric reaction of the digermyl–nickel(II) complex with 1-hexyne provided vinylidenedigermacyclopentane as the major product (Scheme 62).^167^

**Scheme 63:**
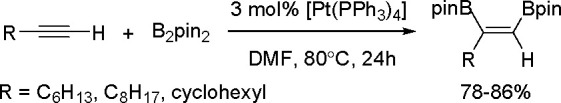
Bis-Boration of Terminal Alkynes with Bis(pinacolato)diboron

The catalytic addition of diboranes to internal and terminal alkynes in the presence of a platinum catalyst to form *cis*-diboration products was discovered by Miyaura and co-workers in 1993 (Scheme 63). In the original report, NMR data showed that the oxidative addition of B_2_pin_2_ to [Pt(PPh_3_)_4_] followed the generally accepted oxidative addition/reductive elimination mechanism.^168^

**Scheme 64:**
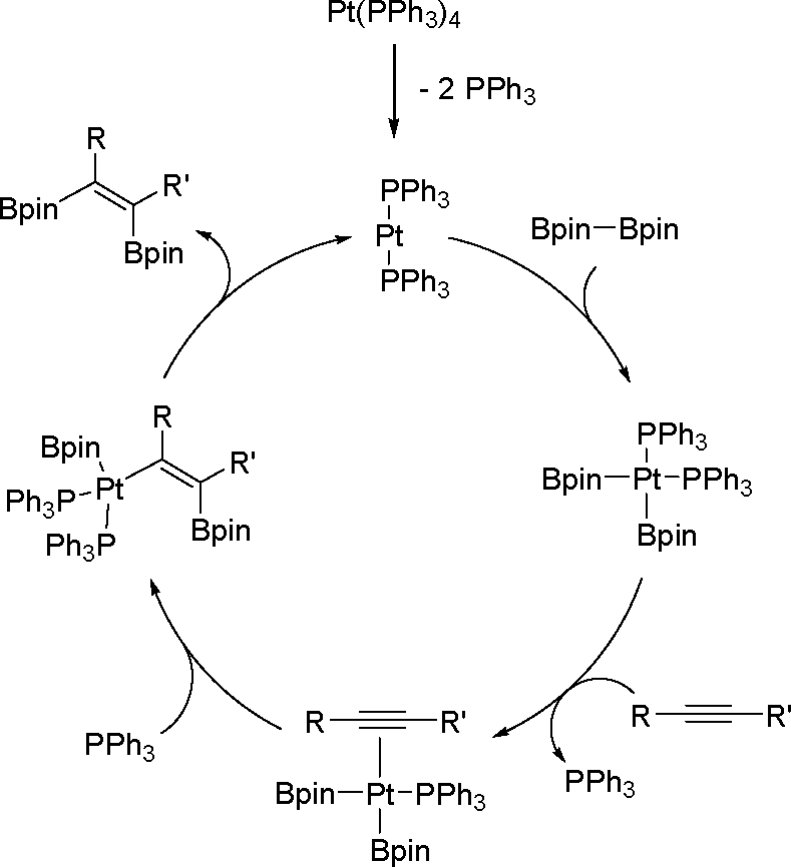
General Mechanism of the Platinum-Catalyzed Bis-Boration of Alkynes

In the following years, Pt(0) complexes were proved to be the most effective catalysts for diboration reactions, for example [Pt(PPh_3_)_2_(C_2_H_4_)_2_]^169^ and [Pt(dba)_2_]/[3,5-(CF_3_)_2_C_6_H_3_]_3_P.^170^ Platinum(0) monophosphine complexes containing PCy_3_ or PPh_2_(*o*-Tol) ligands generated in situ from [Pt(nbe)_3_] (nbe–norbornene) as well as isolated platinum(0) complex [Pt(PCy_3_)(η^2^-C_2_H_4_)_2_] provide high catalytic activity for terminal and internal alkyne diboration at room temperature.^171^ However, nanoporous gold^172^ and platinum nanoparticles^173^ were also found to be effective catalysts for the diboration of internal alkynes with B_2_pin_2_. Most Pt-catalyzed diboration reactions employ symmetrical diboranes, such as bis(pinacolato)diboron (B_2_pin_2_) and bis(catecholato)diboron (B_2_cat_2_), with the latter being more active. The application of a diborane with two different boryl groups, (pin)B–B(dan) (dan = naphthalene-1,8-diaminato), provides access to unsymmetrically substituted bis-boronated alkenes, with the B(dan) group occupying the terminal carbon position.^170^ Platinum-catalyzed diboration of alkynes is proposed to proceed via the oxidative addition of B–B bonds to Pt(0), insertion of C–C multiple bonds into Pt–B bonds and reductive elimination of C–B bonds with regeneration of the catalytically active Pt(0) species (Scheme 64).

**Scheme 65:**
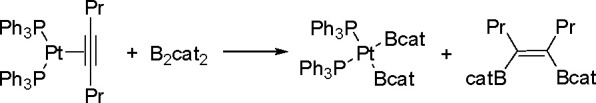
Synthesis of a Bis(boryl)–Platinum(II) Complex

Experimental evidence has been found for each step of the catalytic cycle.^174^ The groups of Marder^169^ and Smith^175^ reported the isolation and characterization of [Pt(PR_3_)_2_(B(OR)_2_)_2_] complexes, which proved to be highly active in the diboration of alkynes (Scheme 65). On the basis of studies with monodentate and chelating phosphine ligands, the authors concluded that phosphine dissociation was a key step in the diboration reaction mechanism.

The results obtained by the Braunschweig^176^ and Marder^177^ groups on the reversible oxidative addition/reductive elimination of diboranes in Pt(II) complexes show a more complicated reaction mechanism. The oxidative addition of B_2_cat_2_ to rhodium(I) and iridium(I) complexes^178,179^ as well as to cobalt(0) precursors,^180^ further supported by studies on alkene insertions into TM–boron bonds, suggested that diborations using other TM catalysts could occur through a similar reaction mechanism. However, it is noted that dehydrogenative borylation products of rhodium catalysis could also be detected as a result of a competitive β-hydride elimination process. Pt(0) complexes are generally more effective in alkyne diboration than Rh(I) and Pd(0) complexes since the increased d-electron energy of Pt(0) facilitates oxidative addition more than Pd(0) and Rh(I). However, [Pd(ITMe)_2_(PhC≡CPh)] complex (ITMe = 1,3,4,5-tetramethylimidazol-2-ylidene) has been found to be a highly reactive precatalyst in the diboration of sterically and electronically demanding alkynes under mild reaction conditions. DFT calculations suggest a reaction pathway similar to that proposed for platinum phosphine analogues. According to the results of the computational study, dissociation of one NHC ligand is a crucial part of the reaction mechanism. Destabilization of the [(BR_2_)_2_Pd(0)(ITMe)_2_] complex by the NHC ligand has been shown to facilitate oxidative addition of the B–B bond.^181^

**Scheme 66:**
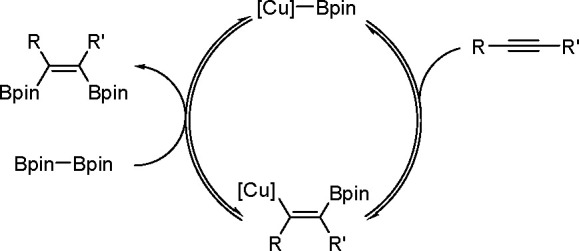
Insertion of Alkyne into the Cu–B Bond

Yoshida and co-workers disclosed general *syn*-diboration with bis(pinacolato)diboron using a Cu(OAc)_2_/PCy_3_ catalytic system,^182^ which was equally effective as platinum catalysts. The reaction proceeds through a boryl-substituted alkenyl–copper intermediate (formed by alkyne insertion into a Cu–B bond), which reacts with a diborane to give a bis-boration product, thereby regenerating the boryl–copper complex (Scheme 66).

**Scheme 67:**
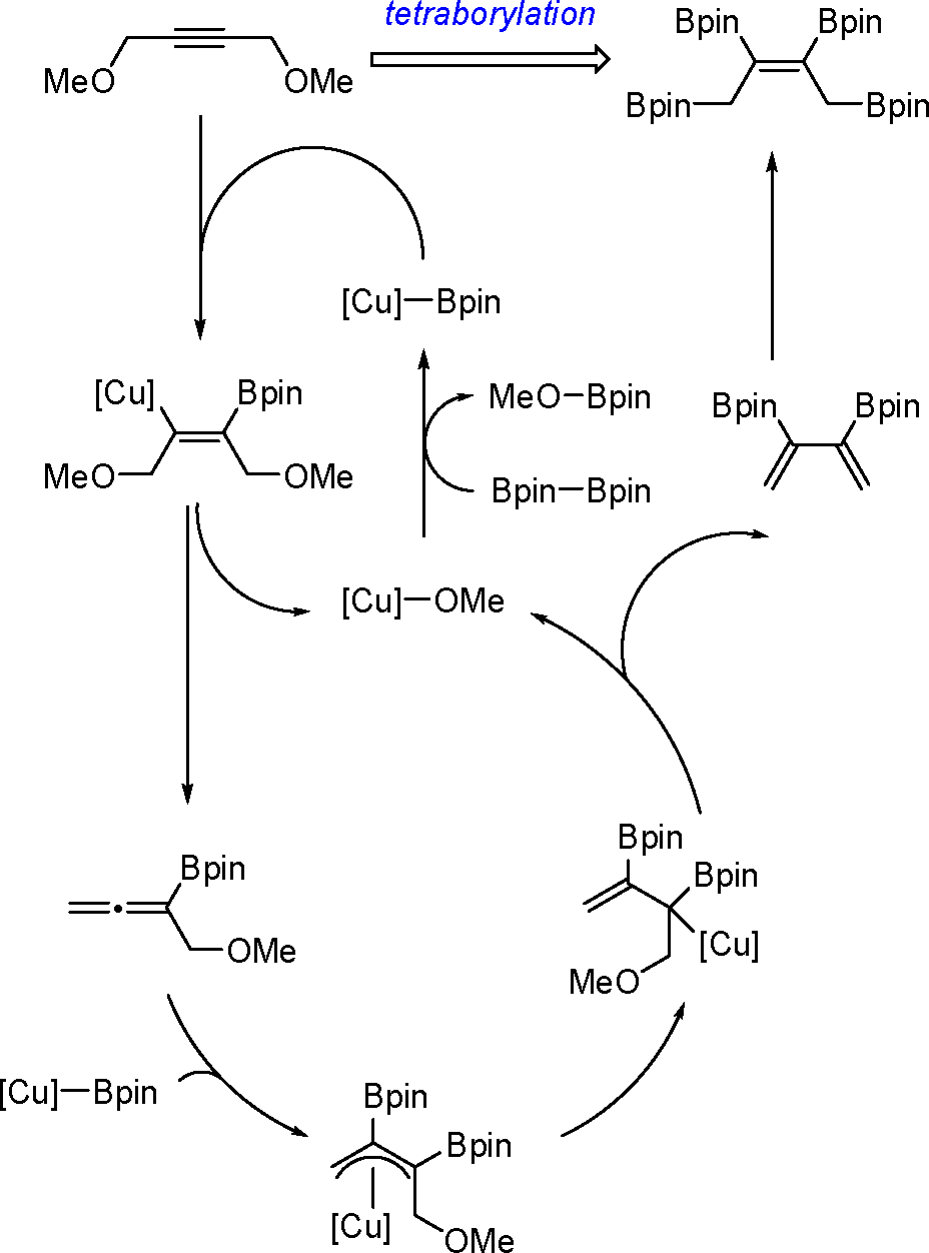
Mechanism of Copper-Catalyzed Alkyne Tetraboration

This catalytic system has been explored using 1,4-dimethoxy-2-butyne converted into a tetraborylated product, in which the MeO groups were also substituted by B(pin) groups.^182^ This unique tetraborylation reaction involves the formation of borylallene (by the elimination of copper(I) methoxide from the alkenyl–copper intermediate) and 2,3-diboryl-1,3-butadiene (borylallene insertion into a Cu–B complex) as key intermediates. In the final step, 2,3-diboryl-1,3-butadiene underwent copper(I)-catalyzed 1,4-diboration to give tetraborylethylene (Scheme 67).

**Scheme 68:**
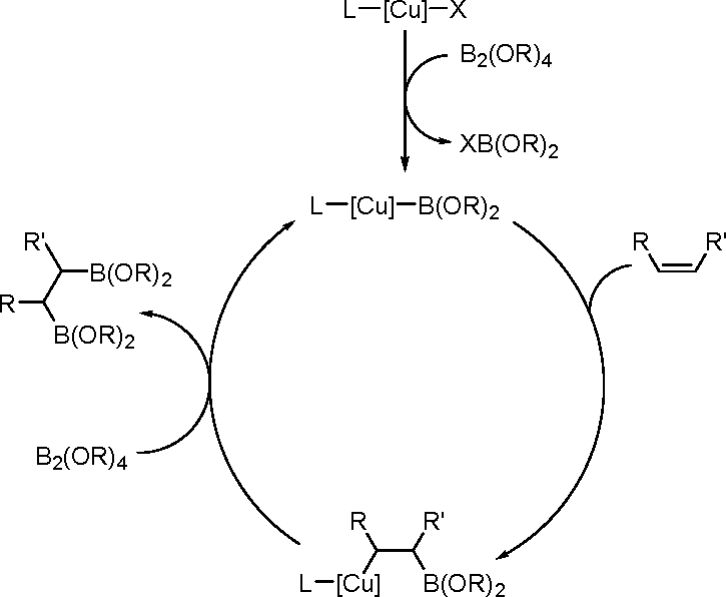
Mechanism of Copper-Catalyzed Alkene Bis-Boration

The high catalytic activity of copper(I) complexes in alkene diboration reactions prompted the search for a new mechanism based on a series of σ-bond metathesis steps, resulting in the overall transfer of both boryl groups to the substrate.^183^ Computational studies have shown that in contrast to the catalytic cycle proposed for platinum(0) complexes, the catalytic cycle of copper catalysts is believed to operate through the transmetalation pathway. The reaction occurred via the formation of a monoborylcopper(I) complex. Insertion of an alkene into the Cu–B bond formed an alkyl–copper(I) complex, which subsequently reacted with diborane to produce the bis-boration product with regeneration of the boryl–copper complex (Scheme 68). Detailed DFT studies by Marder, Lin, and co-workers showed the mechanism and confirmed differences in the reactivity of B_2_cat_2_ and B_2_pin_2_.^184^ Further support for the stepwise transfer of boryl groups was provided by Fernández and co-workers who generated mixed bis-borylalkanes using two different diboranes.^185^

Stoichiometric σ-bond metathesis reactions of nickel(II) and cobalt(II) *tert*-butoxide complexes with B_2_cat_2_ or B_2_pin_2_ to generate TM–boryl species and to support the transmetalation mechanism of diboration have been also demonstrated.^186^

**Scheme 69:**
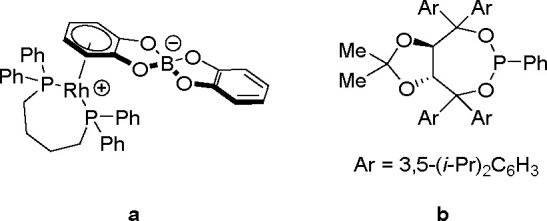
(a) Zwitterionic η^6^-(Arene)Rhodium Complex and (b) TADDOL Ligand Structure

The catalytic diboration of alkenes was first reported in 1995 and employed bis(catecholato)diboron in the presence of Rh or Au catalysts.^187^ Catalysis with the [RhCl(PPh_3_)_3_] rhodium complex was based on an earlier report on the oxidative addition of B_2_cat_2_ with Rh(I) to give bis(boryl)Rh complexes and on another report showing that bis(boryl)rhodium complexes reacted with alkenes to give alkene 1,2-diborylation.^188^ β-Hydrogen elimination, which generates vinyl boronates, often competes with the reaction pathway in the presence of rhodium catalysts. However, the formation of byproducts arising from β-hydride elimination can be significantly diminished by using a zwitterionic η^6^-(arene)rhodium catalyst with a diphenylphosphinomethane ligand (dppm) (Scheme 69a).^189^ The synthetic utility of rhodium-catalyzed diboration has been considerably expanded by the development of its enantioselective variant in the presence of an (S)-QUINAP/[(nbd)Rh(acac)] catalytic system ((S)-QUINAP is (*S*)-1-(2-diphenylphosphino-1-naphthyl)isoquinoline).^190^

**Scheme 70:**
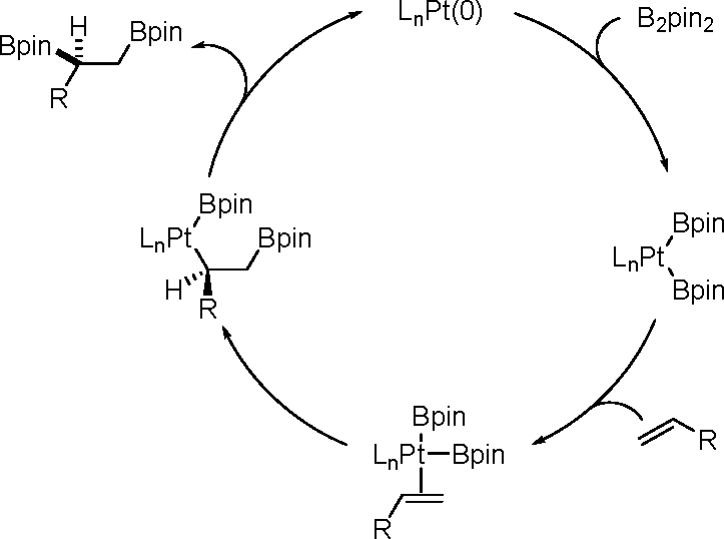
Mechanism of Enantioselective Pt(0)-Catalyzed Terminal Alkene Bis-Boration

Successful catalytic bis-boration of aliphatic alkenes with B_2_pin_2_^191^ and B_2_cat_2_^192^ using nonphosphine platinum(0) catalysts bearing π-ligands (dibenzylideneacetone (dba), norbornene (nbe) or cyclooctadiene (cod)) has been disclosed. An experimental and computational study of enantioselective [Pt(dba)_3_]-catalyzed terminal alkene bis-boration in the presence of chiral phosphonite ligands (TADDOL-derivatives, Scheme 69b) was published by Morken and co-workers^193^ A DFT study combined with other experimental data suggests olefin migratory insertion into a Pt–B bond to give an internal C–Pt intermediate as the rate-limiting and stereocontrolling step of the reaction (Scheme 70).

**Scheme 71:**
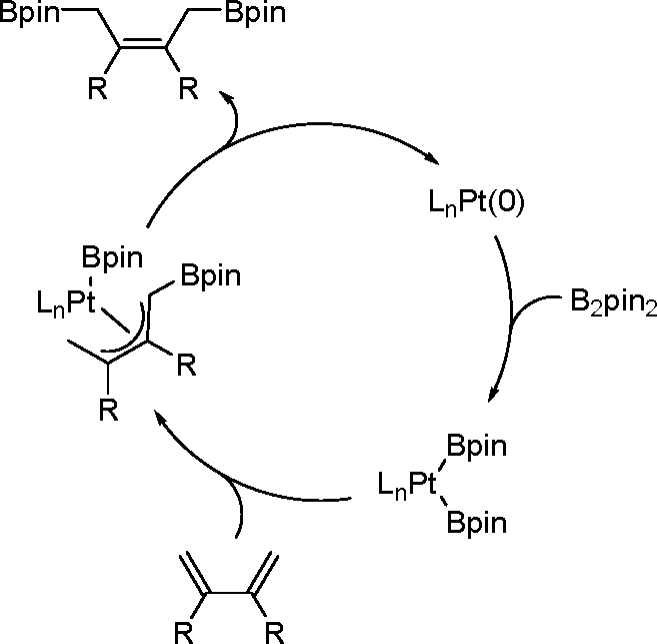
Mechanism of Pt(0)-Catalyzed Bis-Boration of Conjugated 1,3-Dienes

Catalytic bis-boration of conjugated 1,3-dienes proceeded in the presence of platinum(0) catalysts (Scheme 71). Depending on the structure of the substrate and catalysts, the reaction leads either to 1,4-diboration products–as in the presence of [Pt(PPh_3_)_4_]^194^–or to 1,2-diboration products when the reaction is mediated by [Pt(dba)_2_].^191^

**Scheme 72:**
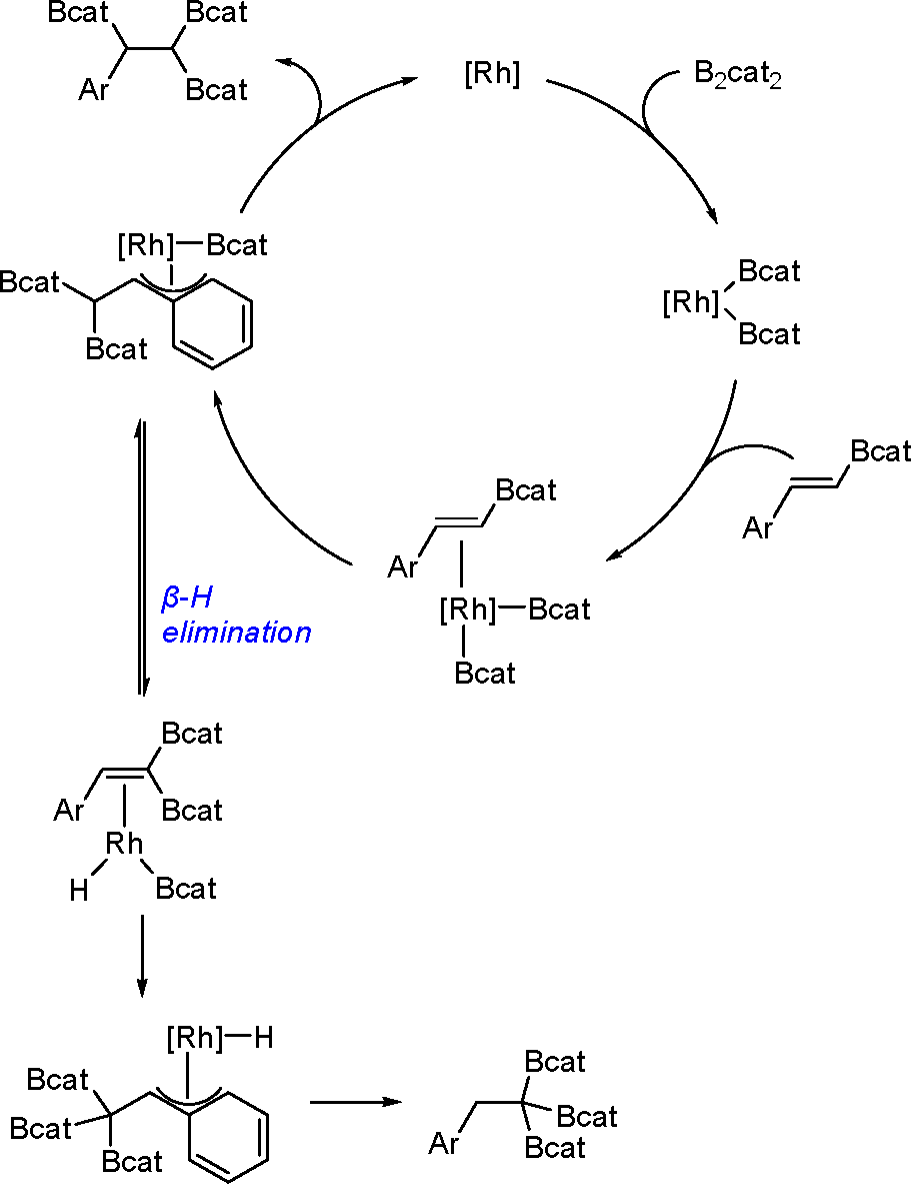
Mechanism of the Rh(I)-Catalyzed Bis-Boration of (*E*)-Styrylboronate Esters

The Rh(I)-catalyzed bis-boration of (*E*)-styrylboronate esters gave predominantly 1,1,1-triboryl-2-arylethane in the presence of Wilkinson’s catalyst, but isomeric 1,1,2-triboryl-2-arylethanes were formed with the [{Rh(coe)_2_(μ-Cl)}_2_] complex.^195^ The formation of untypical 1,1,1-triboryl-2-arylethanes involves the regiospecific insertion of vinylboronates into a Rh–B bond followed by β-hydride elimination and the second insertion of 2,2-vinyl bis(boronate) into the remaining Rh–B bond and C–H reductive elimination, leading to 2,2-diboration and a 2,1-hydrogen shift (Scheme 72).

**Scheme 73:**
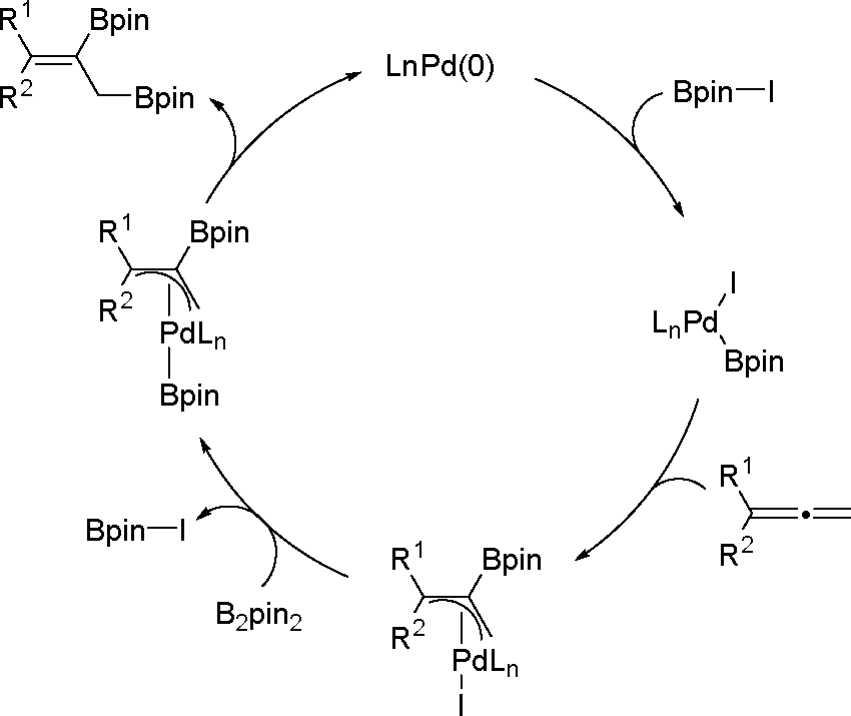
Mechanism of the Pd(0)-Catalyzed Three-Component Coupling of Iodoborane, Diborane, and Allene

A new mechanism has been discovered in the study of the palladium-catalyzed bis-boration of allenes in the presence of alkenyl- or aryl iodide as a cocatalyst. This three-component coupling of iodoborane, diborane, and allene proceeds through the oxidative addition of the boron–iodine bond instead of the boron–boron bond, and the terminal double bond of the allene is then inserted into the palladium–boron bond. The transmetalation with diborane results in the elimination of the diboration product and regeneration of the palladium–boryl–iodide complex (Scheme 73).^148^

**Scheme 74:**
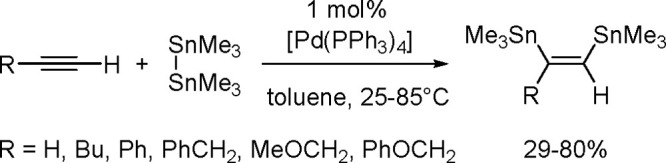
Bis-Stannation of Terminal Alkynes with Hexamethyldistannane

Bis-stannation (bis-stannylation) of functionalized terminal alkynes with hexamethyldistannane catalyzed by [Pd(PPh_3_)_4_] has been reported for the first time in good yields by Mitchell and co-workers (Scheme 74).^196^

**Scheme 75:**
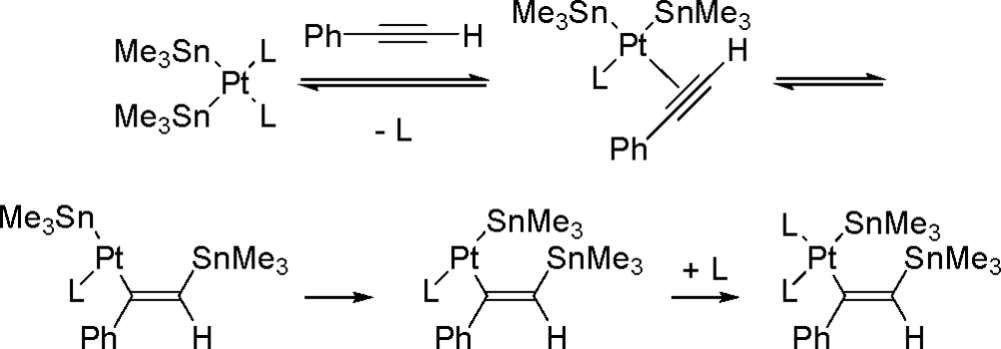
Insertion of Phenylacetylene into Pt–Sn Bonds

As for the other bis-metalations, it is believed that reactions of alkynes with distannanes proceed via *syn*-addition according to the oxidative addition/reductive elimination mechanism. A detailed experimental study of the insertion of phenylacetylene into Pt–Sn bonds was reported by Ozawa and co-workers (Scheme 75).^197^

**Scheme 76:**
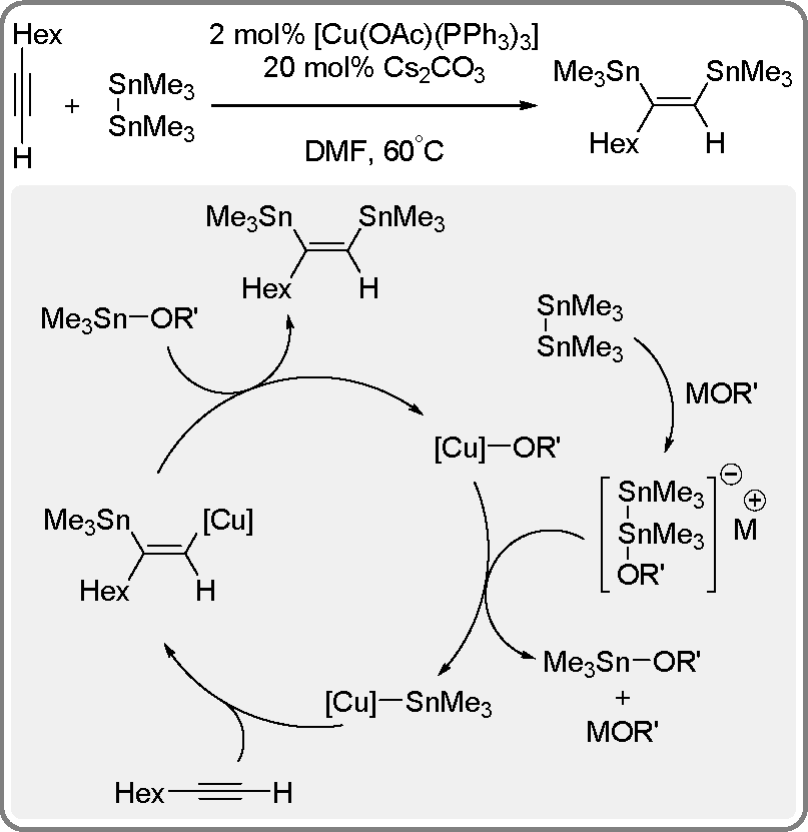
Mechanism of the Bis-Stannation of Alkynes with Hexamethyldistannane Using a Cu(I) Catalyst

Catalysts other than platinum and palladium complexes are rarely used in bis-stannylation reactions. However, similarly to bis-boration, addition of Sn–Sn bonds can be achieved with copper catalysts. Yoshida and co-workers reported catalytic bis-stannylation of alkynes with Me_3_Sn–SnMe_3_ using a copper catalyst, [Cu(OAc)(PPh_3_)_3_], in the presence of Cs_2_CO_3_. The proposed key intermediate in this process is an inorganometallic complex containing a Cu–Sn bond derived from an alkoxycopper complex and a base-activated distannane (Scheme 76).^198^

**Scheme 77:**
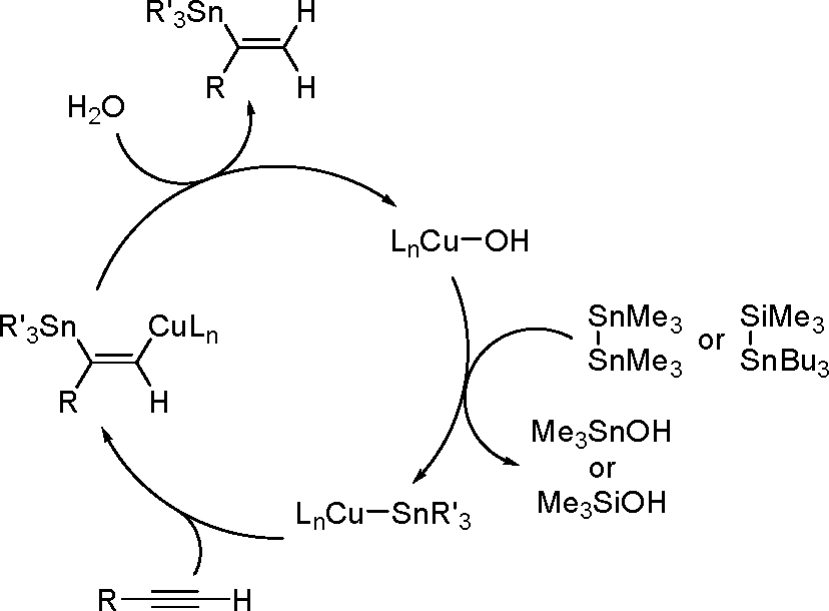
Mechanism of the Markovnikov Hydrostannation of Terminal Alkynes with Distannanes

Stannyl-substituted alkenylcopper species were also found to serve as key intermediates in the α-selective hydrostannylation of terminal alkynes using Me_3_Sn–SnMe_3_ or Me_3_Si–SnBu_3_ as a stannylating agent and water as a hydrogen source.^199^ Formation of the Cu–Sn complex proceeds through σ-bond metathesis between a hexamethyldistannane (or trimethylsilyltributylstannane) and Cu–OH complex that is formed in the reaction mixture in the presence of water. The resulting stannylcopper species is inserted into the carbon–carbon triple bond (stannylcupration), which induces the formation of β-stannylvinylcopper species. Finally, the latter is transformed into a hydrostannylation product through protonation with water followed by regeneration of copper hydroxyl intermediate (Scheme 77).

### 2.3 Addition of Heteroelement–Element Linkages to Unsaturated Substrates

**Scheme 78:**
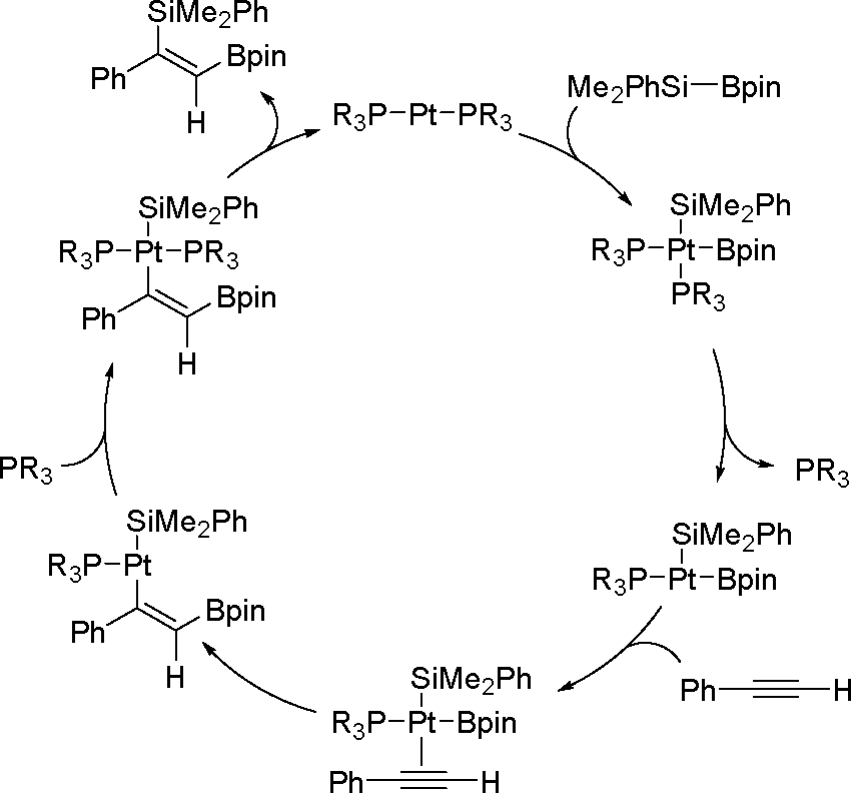
Mechanism of Pt(0)-Catalyzed Alkyne Silaboration

Silaboration (silylboration or silylborylation) of alkynes and silaborative carbocyclization of diynes and enynes have first been reported by Ito^200^ and Tanaka groups.^201^ As for homoelement–element linkages, the proposed catalytic cycle for silaboration using Pt group catalysts involves an initial oxidative addition resulting in the formation of a *cis*-[Pt(L)_2_(SiR_3_)(BR_2_)] complex. The alkyne then undergoes migratory insertion into the M–B bond to form the corresponding [Pt(L)_2_(SiR_3_)(CH=CHBR_2_)] species, followed by reductive elimination to form 1-silyl-2-borylalkene (Scheme 78).^202^

**Scheme 79:**
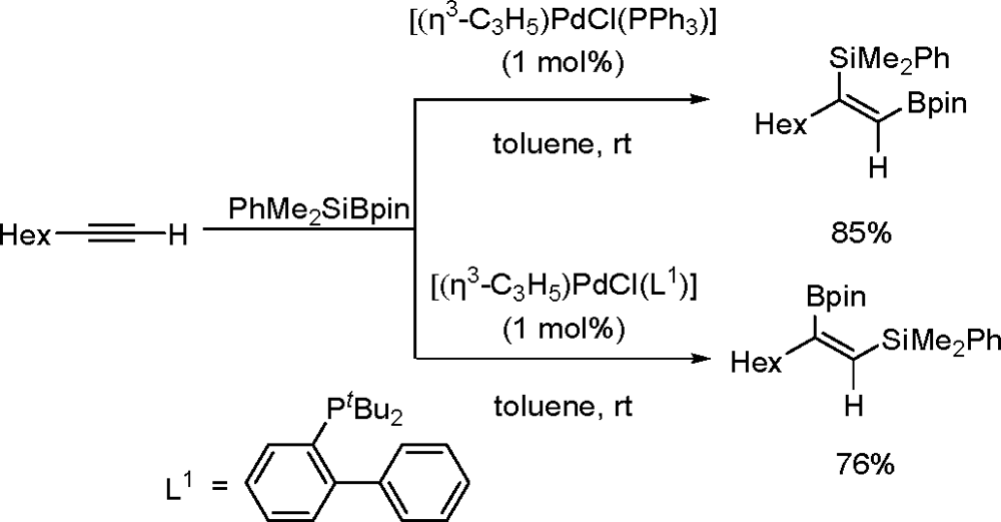
Regiodivergent Oct-1-yne Silaboration in the Presence of Palladium Complexes

The catalytic addition of Si–B bonds to alkynes is highly regio- and stereoselective since the boryl group is usually linked to the terminal carbon atom, whereas the silyl group is connected to the internal carbon atom. However, the application of palladium complexes and bulky phosphine ligands reverses the regioselectivity, yielding products that contain terminal silyl groups.^203^ Thus, the silaboration of alkynes with silylboronic esters proceeds with normal regioselectivity in the presence of [(η^3^-C_3_H_5_)Pd(PPh_3_)Cl] to give 1-boryl-2-silyl-1-alkenes, but 2-boryl-1-silyl-1-alkenes are formed selectively when a palladium catalyst bearing P(*t*-Bu)_2_(biphenyl-2-yl) is applied (Scheme 79). Quantum-chemical calculations suggest that ligand-controlled regioselective alkyne insertion into the Pd–B bond followed by reductive elimination is responsible for the observed switch in regioselectivity.^203^

Isolation of the oxidative addition products of Pt complexes is extremely rarely reported because of their low stability. However, Ozawa and co-workers have demonstrated that all the elementary processes presumed for the catalytic silaboration of phenylacetylene with PhMe_2_SiBpin can be examined stepwise using platinum complexes with phosphine ligands.^204^ Thus, silyl(boryl)platinum(II) complexes prepared by the oxidative addition of silylborane to platinum(0) complexes of the formula *cis*-[Pt(SiMe_2_Ph)(Bpin_2_)L_2_] (L = PMe_3_, PMe_2_Ph, PEt_3_) undergo selective insertion of phenylacetylene into the Pt–B bond to give *cis*-[Pt{C(Ph)=CH(Bpin)}(SiMe_2_Ph)L_2_]. The insertion complexes undergo C–Si reductive elimination to give (*Z*)-α-silyl-β-borylstyrene.

**Scheme 80:**
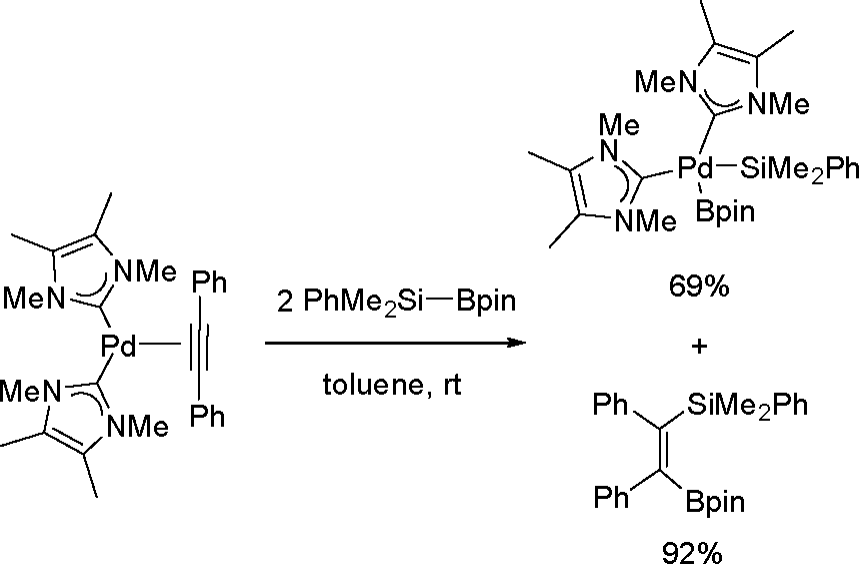
Synthesis of a (Silyl)(boryl)palladium(II) Complex

More recently, a *cis*-[Pd(ITMe)_2_(SiMe_2_Ph)(Bpin)] (ITMe = 1,3,4,5-tetramethylimidazol-2-ylidene) complex was synthesized by the oxidative addition of PhMe_2_SiBpin to a [Pd(ITMe)_2_(PhCCPh)] precursor. This was a very rare example of a (silyl)(boryl)palladium(II) complex isolated from the oxidative addition of a Si–B reagent to a Pd(0) center (Scheme 80).^205^ On the basis of stoichiometric studies, the authors proposed a catalytic cycle of alkyne silaboration in the presence of a (NHC)_2_–Pd(0) complex.^205^

Supported gold nanoparticles (Au/TiO_2_) have been found to catalyze the easily *syn*-selective silylboration of alkynes, giving vicinal boryl(silyl)alkenes.^206^ The regioselective silaboration could be used for aliphatic, aromatic, and propargyl-functionalized terminal alkynes and led to the preferential incorporation of the boryl moiety into the internal carbon. Similarly to homogeneous catalysis, oxidative addition of a silylborane and insertion of an alkyne into [Au]–B(pin) have been proposed to be the key steps of silylboration. The observed regioselectivity may be attributable to steric repulsion between [Au] and a substituent of the alkyne in the insertion step.

**Scheme 81:**
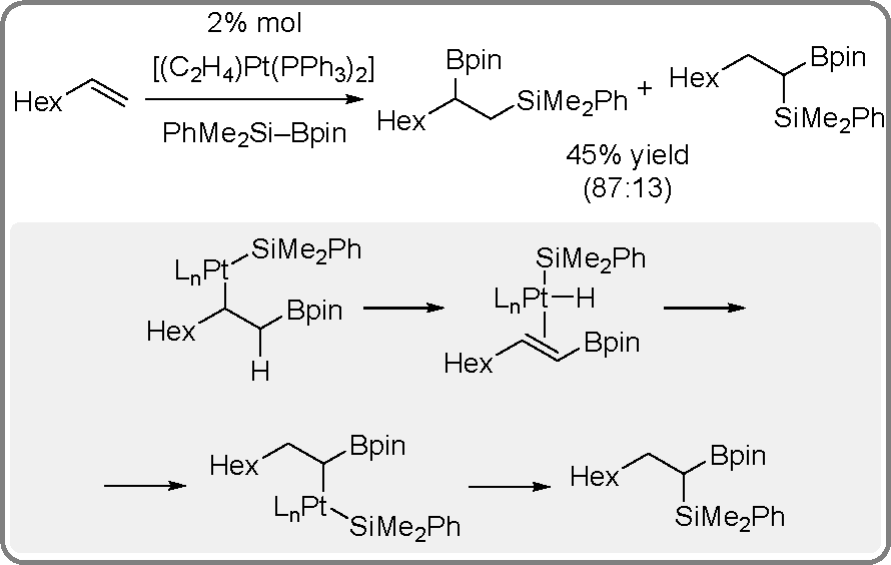
Platinum(0)-Catalyzed Addition of Silylboranes to Terminal Alkenes

In contrast to the regioselectivity observed in alkyne silaboration, the platinum(0)-catalyzed (e.g., [(PPh_3_)_2_Pt(C_2_H_4_)] or [Pt(PPh_3_)_4_]) addition of silylboranes across terminal C=C double bonds resulted in 1,2-difunctionalized alkanes with the silyl group attached to the terminal carbon atom (2-boryl-1-silylalkanes). However, minor amounts of 1,1-silaboration products could also be formed in this process as a result of a competitive *β-*hydrogen elimination/hydrometalation pathway (Scheme 81).^207^

**Scheme 82:**
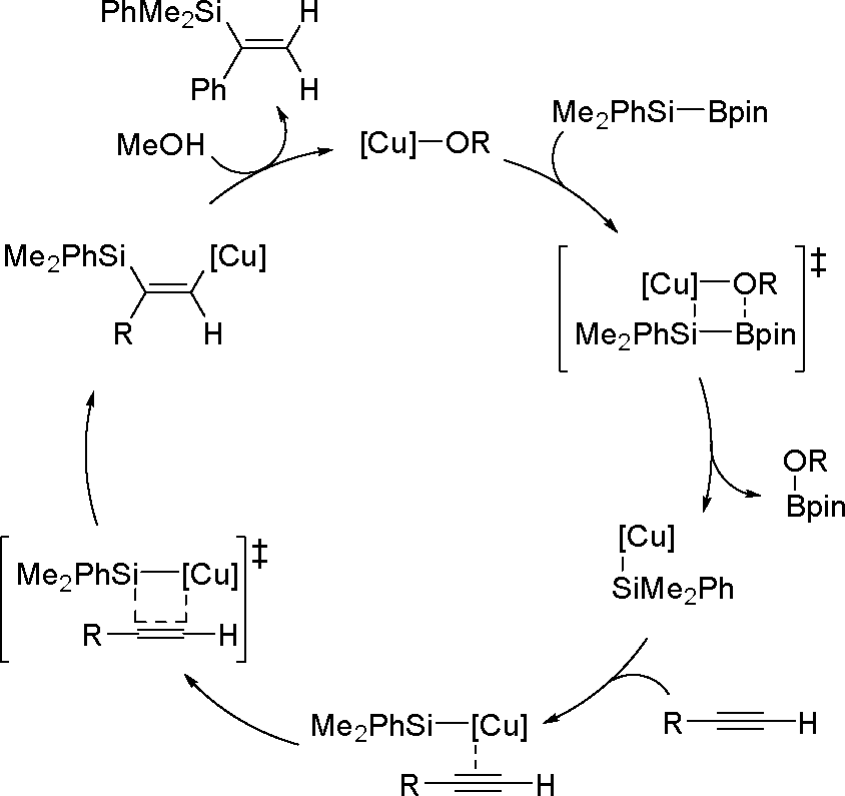
Mechanism of the Markovnikov Silylation of Alkynes Using Silylboranes

In addition to their involvement in classical bis-metalation, silylboranes can also act as silylating reagents. Several examples of the selective preparation of linear or branched vinylsilanes from alkynes, mainly in the presence of copper salts, have been reported in the literature.^208^ The mechanism of this process is similar to copper(I)-catalyzed Markovnikov borylation^117^ with diboranes (Scheme 41) and does not assume the oxidative addition of silylborane to copper, but the formation of the inorganometallic Cu–Si complex via the reaction of alkoxycopper with silylborane, into which an alkyne is inserted, is of key importance (Scheme 82).

**Scheme 83:**
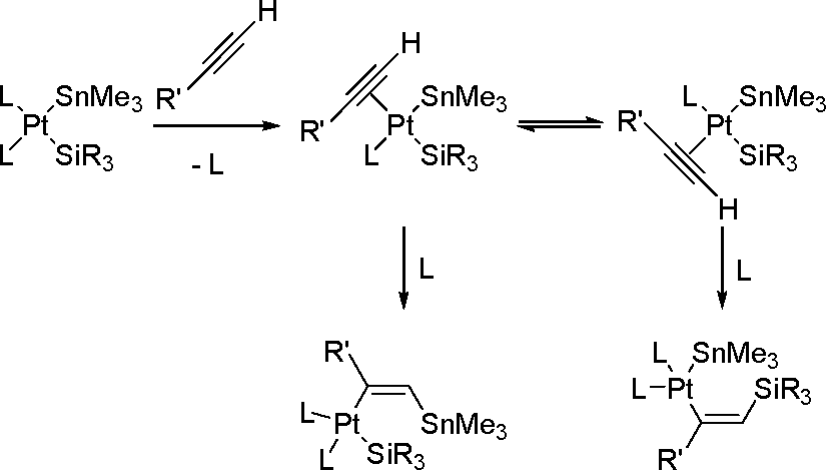
Competitive Alkyne Insertion into Pt–Si and Pt–Sn Bonds

Palladium-catalyzed addition of Si–Sn bonds to terminal alkynes proceeds in a highly regioselective manner with a stannyl group attached to the more substituted carbon atom.^209^ A detailed mechanistic study of alkyne insertion into *cis*-silyl(stannyl)platinum(II) complexes has been reported by Ozawa and co-workers^210^ Competitive insertion of alkyne into Pt–Si and Pt–Sn bonds has been considered, with the ratio of the two product types being dependent on the substituents on the silyl and phosphine ligands (*cis*-[Pt{C(R′)=CHSnMe_3_}(SiR_3_)L_2_] and *cis*-[Pt(SnMe_3_){C(R′)=CHSiR_3_}L_2_], respectively) (Scheme 83).

**Scheme 84:**
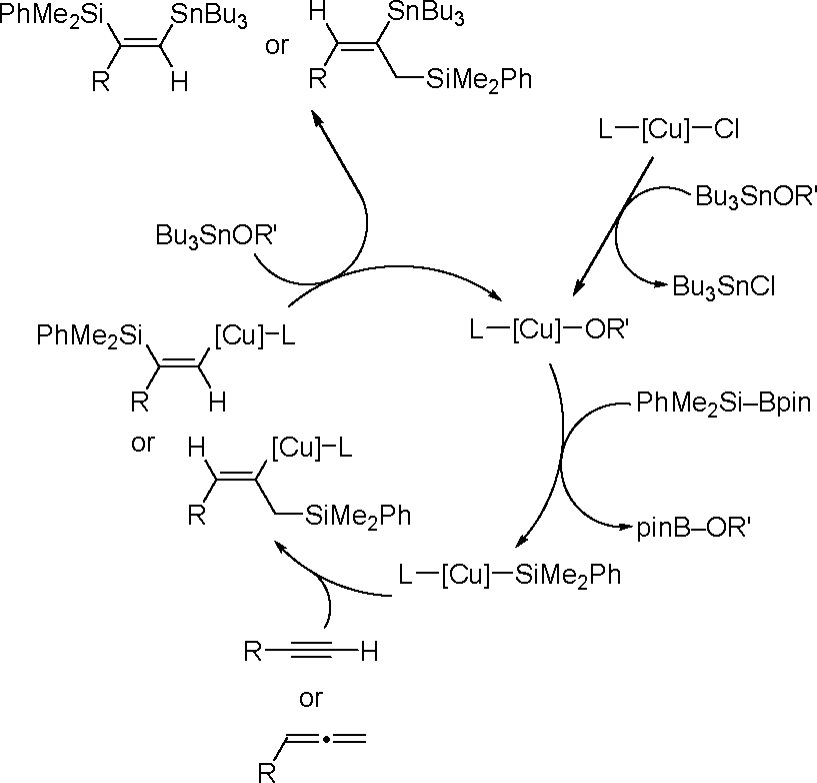
Mechanism of the Cu(I)-Catalyzed Three-Component Silastannation of Terminal Alkynes

A theoretical study of the alkyne silylstannylation mechanism with a palladium catalyst suggested that alkyne insertion into the Pd–Sn bond, with Sn attack at the terminal position, is favored over Pd–Si insertion.^211^ Inverse regioselectivity in the silylstannylation reaction can be achieved using a copper catalyst in the three-component variant of this process, with terminal alkynes, a silylborane (PhMe_2_SiBpin), and a tin alkoxide (*n*-Bu_3_SnO*t*-Bu) in the presence of the [Cu(Cl)P(*t*-Bu)_3_] catalyst. In this case, the silylcopper species, Cu–SiMe_2_Ph (formed via σ-bond metathesis between the copper alkoxide and silylborane), played a key role in the catalytic cycle to enable alkyne insertion into the Cu–Si bond and give a β-silylalkenylcopper intermediate, which in the reaction with the tin alkoxide produced the addition product and regenerated the copper alkoxide (Scheme 84).^212^

**Scheme 85:**
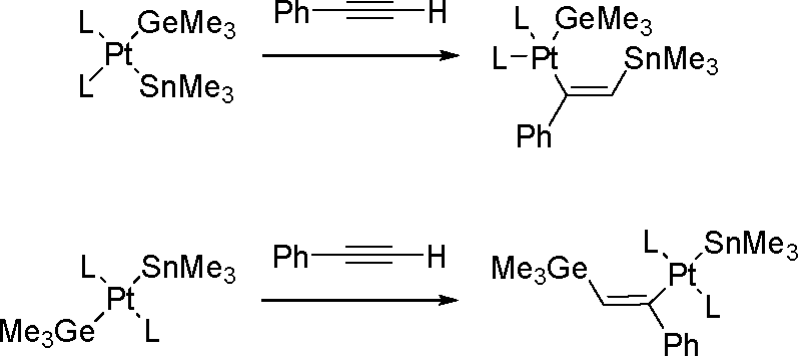
Competitive Insertion of Phenylacetylene into Pt–Sn and Pt–Ge Bonds

Studies of the addition of germastannanes to alkynes are limited to arylacetylenes^213^ and propargylic esters^214^ in the presence of the [Pd(dba)_2_/P(OCH_2_)_3_CEt] or the [Pd(PPh_3_)_4_] catalytic system, respectively. In contrast to other *bis*-metalation products, germylstannylation products are usually formed with poor stereo- and regioselectivity. A study of the insertion of phenylacetylene into a mixture of *cis*-*trans* isomers of [Pt(GeMe_3_)(SnMe_3_)(PMe_2_Ph)_2_], obtained by the oxidative addition of Me_3_Ge–SnMe_3_ with a Pt(0) complex, supported the classical bis-metalation mechanism.^215^ The isomeric germyl(stannyl)platinum complexes underwent competitive insertion of phenylacetylene into Pt–Sn and Pt–Ge bonds to give a mixture of *cis*-[Pt(GeMe_3_){C(Ph)=CH(SnMe_3_)}(PMe_2_Ph)_2_] and *cis*-[Pt{C(Ph)=CH(GeMe_3_)}(SnMe_3_)(PMe_2_Ph)_2_] complexes in the 8:2 ratio (Scheme 85).^215^

**Scheme 86:**
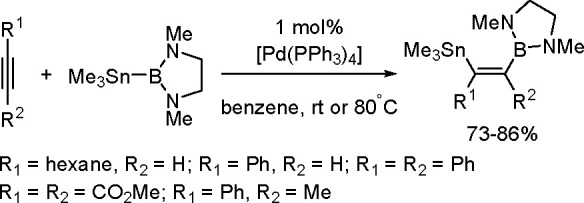
Pd(0)-Catalyzed Borastannation of Terminal and Internal Alkynes

The borastannation (borylstannylation) of terminal and internal alkynes was discovered by Tanaka and co-workers in 1996. Sterically hindered borylstannanes are usually employed, for example, 1,3-dimethyl-2-(trimethylstannyl)-2-bora-1,3-diazacyclopentane (Me_3_SnB[NMe{CH_2_CH_2_}NMe]) or 1,3-di-*tert*-butyl-2-(trimethylstannyl)-2-bora-1,3-diaza-cyclopentane (Me_3_SnB[N*t*-Bu{CH_2_CH_2_}N*t*-Bu]), in the presence of the [Pd(PPh_3_)_4_] catalyst to yield *cis*-1-boryl-2-stannyl-1-alkenes as predominant products (Scheme 86).^216,217^ The results are in agreement with the accepted mechanism, with the alkyne being inserted into the Pd–B bond. Application of Tanaka’s methodology to 1,3-enynes led to the formation of *cis*-1-boryl-2-stannyl-1,3-dienes in a regio- and stereoselective manner.^218^

Regio- and stereoselective installation of boryl and stannyl moieties into the carbon–carbon triple bonds of various alkynes has also been achieved using three-component coupling with a bis(pinacolato)diborane and a tin alkoxide, Bu_3_SnOMe, with the aid of a Cu(OAc)_2_/PCy_3_ catalytic system, and vicinal boryl(stannyl)alkenes were obtained in a straightforward manner.^219^ In this variant of *cis*-borylstannylation, a stannyl alkoxide acted as the electrophile for capturing catalytically generated organocopper species. The stannyl group was found to be exclusively attached to the α-position of an aryl substituent in the reaction with alkyl(aryl)alkynes, whereas the reaction of terminal alkynes led to products with a stannyl moiety at the internal carbon.

A copper complex, [(IMes)CuCl] (IMes = 1,3-bis(2,4,6-trimethylphenyl)imidazol-2-ylidene), has been found to catalyze the borylstannylation of alkenes and allenes through three-component coupling with a diborane and a tin alkoxide, yielding structurally diverse *vic*-boryl(stannyl)alkanes (or alkenylstannanes with boryl moieties in the case of allenes) in a regio- and stereoselective manner.^220^ Copper-catalyzed asymmetric three-component borylstannation of aryl-substituted alkenes with B_2_pin_2_ and Bu_3_SnOMe, leading to the enantioselective formation of α-aryl-β-borylstannane compounds, has also been reported with chiral sulfinylphosphine ligands.^221^

**Scheme 87:**
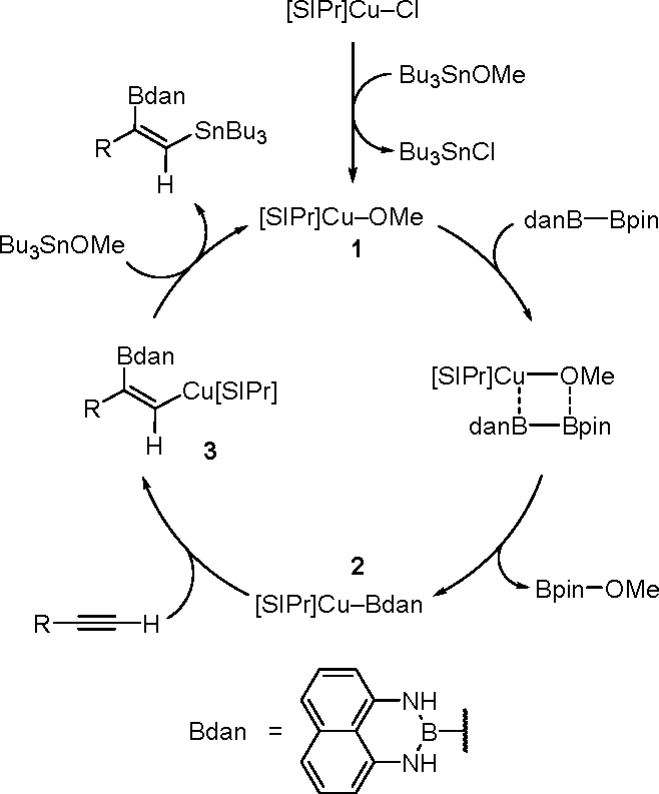
Cu(I)-Catalyzed Three-Component Borylstannation of Terminal Alkynes

A *N*-heterocyclic carbene copper complex [(SIPr)CuCl] (SIPr = 1,3-bis(2,6-diisopropylphenyl)imidazolidin-2-ylidene) is used as a catalyst in the borylstannylation of terminal alkynes with a (pin)B–B(dan) unsymmetrical diborane of inverse regioselectivity to produce compounds with a stannyl moiety at the terminal carbon.^222^ The reaction is initiated by the formation of a catalytically active Cu-OMe complex (**1**), which subsequently reacts with diborane to form a boryl complex (**2**). Insertion of an alkyne into a Cu–B bond generates complex (**3**), which is then captured by tin methoxide to furnish a *cis*-boryl(stannyl)alkene (Scheme 87). A rational explanation of the formation of Cu–B(dan) vs Cu–B(pin) is based on assumption of a selective interaction between the Lewis acidic B(pin) moiety of unsymmetrical diborane and the methoxy moiety of copper methoxide species, which may lead to selective introduction of the boryl group into the triple bond of the alkynes. The regiochemical outcome of the borylstannylation and positioning of the B(dan) moiety at the internal carbon of the terminal alkynes follows from the orientation of the Cu–B species in the borylcupration step.

**Scheme 88:**
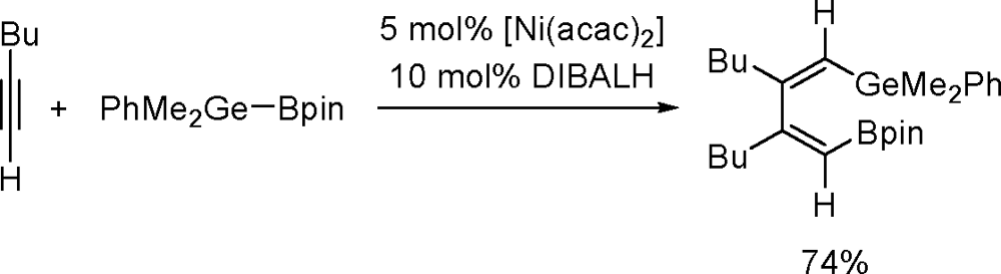
Germaborative Dimerization of Hex-1-yne in the Presence of a Ni(II) Catalyst

The products obtained in the germaboration (germylborylation) of alkynes are highly dependent on the metal catalyst used. Classical *cis*-addition products can be obtained in the presence of the [Pt(PPh_3_)_2_(C_2_H_4_)] catalyst; however, with the [Ni(acac)_2_]/DIBALH catalytic system, the reaction of germylboranes with alkynes led to 1-germyl-4-borylbuta-1,3-diene derivatives in a regio- and stereoselective manner (Scheme 88).^223^

## 3 Activation (and Functionalization) of C–H Bonds with Hydrometalloids and Bismetalloids

### 3.1 Dehydrogenative Borylation of C–H Bonds

**Scheme 89:**
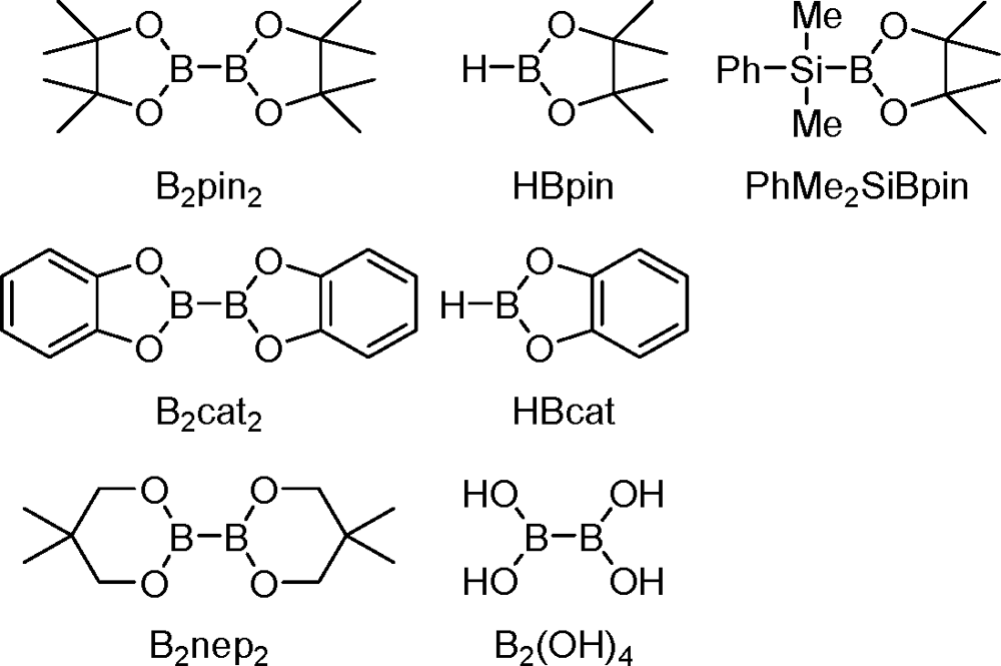
Commonly Used Borylating Agents

Direct borylation of C–H bonds is currently one of the best developed types of metal-catalyzed C–H bond functionalization. The reaction is catalyzed by metal salts and complexes of Ir, Zn, Pd, Co, Cu, Fe, and Ag. Procedures for the borylation of C–H bonds in alkanes, alkenes, arenes, and alkynes that ensure high yields and high selectivities have been described. The reaction permits the synthesis of several useful building blocks of wide applicability in organic synthesis. The state of knowledge of the reaction has been presented in many reviews.^100,224−230^Scheme 89 illustrates the borylating agents that are most frequently used in C–H and C–X borylation.

#### 3.1.1 Dehydrogenative Borylation of C(sp)–H Bonds

**Scheme 90:**
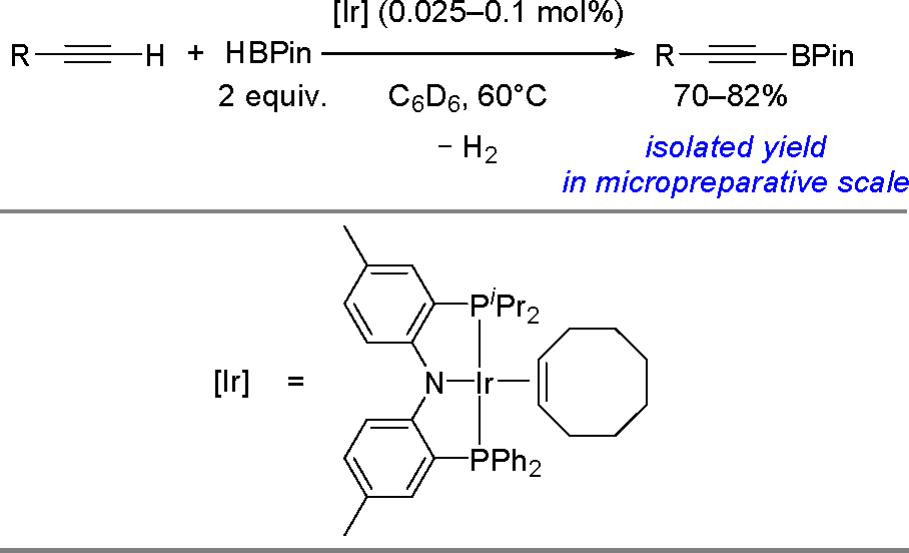
Dehydrogenative Borylation of Acetylenes with Pinacolborane

Dehydrogenative borylation of C(sp)–H bonds in terminal alkynes greatly expands the access to alkynylboronates that act as versatile reagents in organic synthesis.^231^ This has recently been briefly summarized.^232^ The first efficient examples of the reactions were discovered by Ozerov and co-workers who reported the activity of iridium complexes featuring a silyl–amido–quinoline tridentate SiNN-pincer ligand in the selective and efficient reaction of a variety of terminal alkynes with pinacolborane to form alkynylboronates.^233^ The most promising results were obtained using iridium–diarylamido/bis(phosphine) pincer complexes (Scheme 90).^234^ Selective and high-yield borylation of a variety of alkynes was possible in the presence of small amounts of catalysts.

**Scheme 91:**
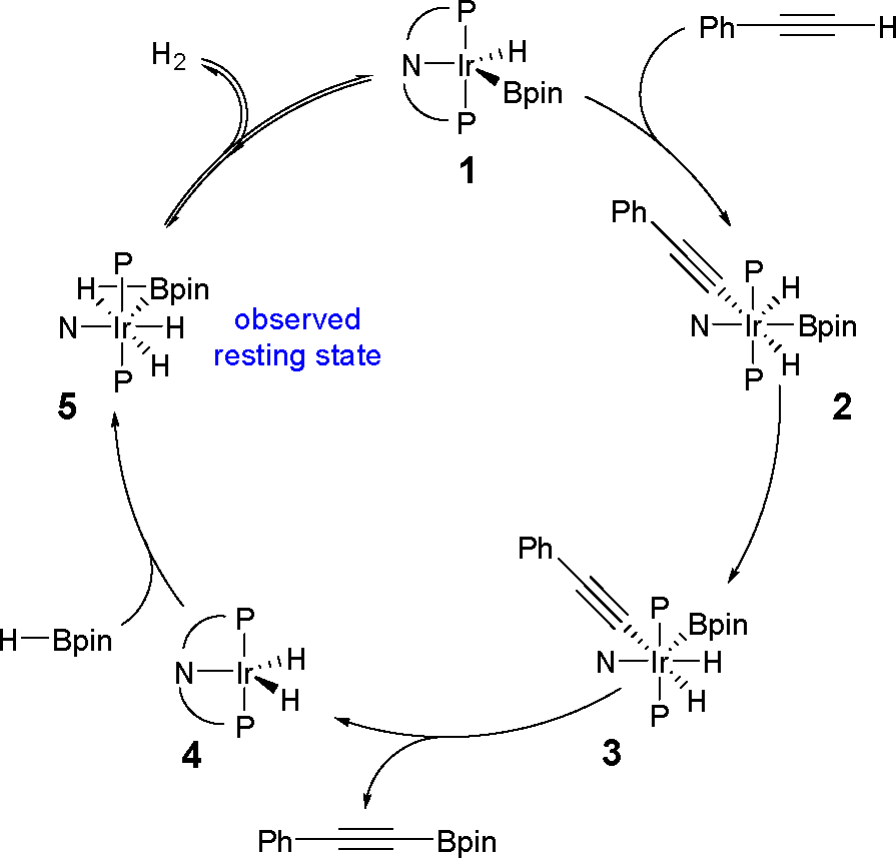
Mechanism of C(sp)–H Borylation in the Presence of an Ir-PNP-Pincer Complex (P= P(*i*-Pr)_2_)^128^ For clarity, the structure of the diarylamidobis(phosphine) pincer ligand is not shown in complexes **2**, **3** and **5**.

On the basis of experimental studies, including detailed kinetic studies, as well as computational analysis in the presence of optimized PNP–pincer iridium complexes, the mechanism of the process was suggested (Scheme 91).^128^

According to the proposed mechanism, the iridium(III) monoboryl monohydride complex (**1**) was assumed to be the active form of the catalyst. Complex **1** is in equilibrium with the dihydrido(σ-pinacolborane)iridium(III) complex (**5**), which is the resting state of the catalyst. Oxidative addition of phenylacetylene to **1** leads to the formation of **2**. Subsequently, to displace the alkynyl group next to the boron molecule and allow the reductive elimination of the C–B bond, rotation of the B–H unit and formation of complex **3** is needed. In DFT calculations, this process has been found to be the rate-limiting step. Subsequently, reductive elimination of alkynylboronate from **3** produces dihydrido–iridium complex **4**. The catalytic cycle is completed by the oxidative addition of HBpin and reductive elimination of dihydrogen.

#### 3.1.2 Dehydrogenative Borylation of Olefinic C(sp^2^)–H Bonds

Vinylboronate esters are useful intermediates in organic synthesis. Catalytic methods for their formation include hydroboration of alkynes, palladium-catalyzed borylation of alkenyl halides, hydrogenation of 1-borylalkynes, cross-metathesis of terminal olefins with vinylboronate, and diboration of alkynes with diboron compounds. Borylation of olefins has recently been briefly reviewed.^125^ Dehydrogenative borylation of olefins with HBR_2_ is discussed in Section 1.5.1. Herein, dehydrogenative borylation of olefins with diboron derivatives is described.

**Scheme 92:**
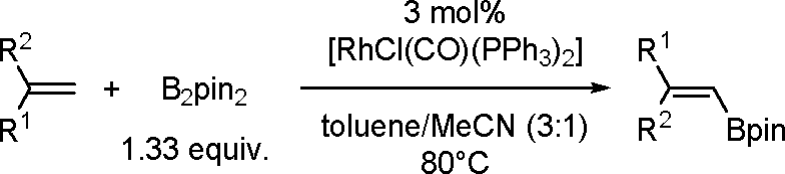
Dehydrogenative Borylation of Olefin with Bis(pinacolato)diboron

Borylation of vinylarenes and 1,1-disubstituted alkenes with bis(pinacolato)diboron (B_2_pin_2_) in the presence of a rhodium catalyst, leading to corresponding vinylboronate esters, has first been reported by Marder (Scheme 92).^235^ The method does not suffer from significant formation of hydroboration and/or hydrogenation byproducts, seen when H-BR_2_ is used as the borylating agent.

**Scheme 93:**
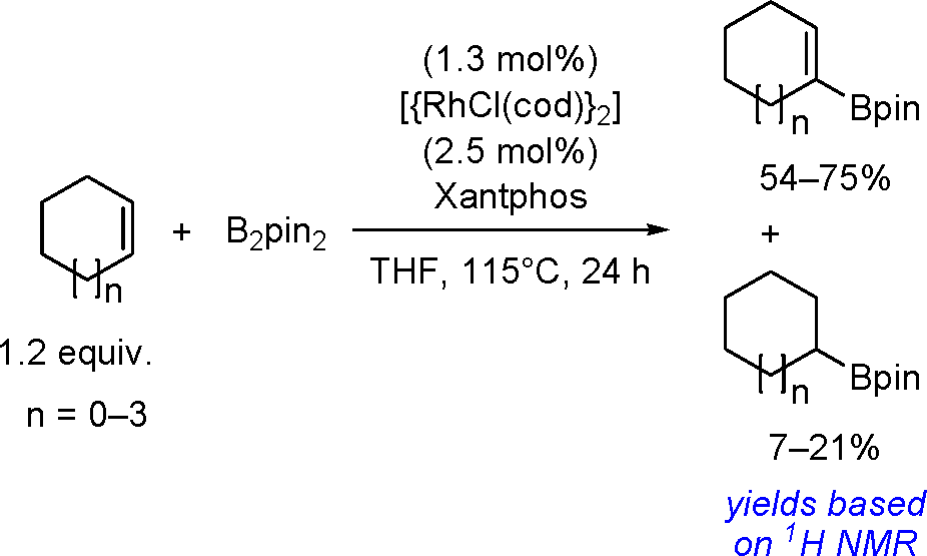
Undirected Dehydrogenative Borylation of Cycloolefins with B_2_pin_2_

The use of double excess B_2_pin_2_ and a higher catalyst concentration (5 mol %) afforded a 1,1-disubstituted derivative that could not be synthesized by alkyne hydroboration. In a separate paper, the same authors reported the conditions resulting in higher yields and selectivities. The reaction time could be shortened to 60 min by using microwave reactors.^236^ The rhodium complexes also show activity in the borylation of cycloolefins, but in this case, saturated products also form in the reaction (Scheme 93).^237^

Allylsilanes and some vinyl substrates such as methyl acrylate or vinyl methyl ketone undergo borylation with B_2_pin_2_ in the presence of [{IrCl(cod)}_2_].^238,239^ A similar [{Ir(OMe)(cod)}_2_] complex and 4,4′-di-*tert*-butyl-2,2′-bipyridine (dtbpy) have been used in the borylation of cyclic vinyl ethers.^240^ Optimized conditions for the iridium-catalyzed borylation of α,β-unsaturated esters with B_2_pin_2_ involve [{IrCl(cod)}_2_] or [{Ir(OMe)(cod)}_2_] and AsPh_3_ as the ligand of choice. Such reaction conditions give borylated esters with isolated yields of 29–56%.^241^

**Scheme 94:**
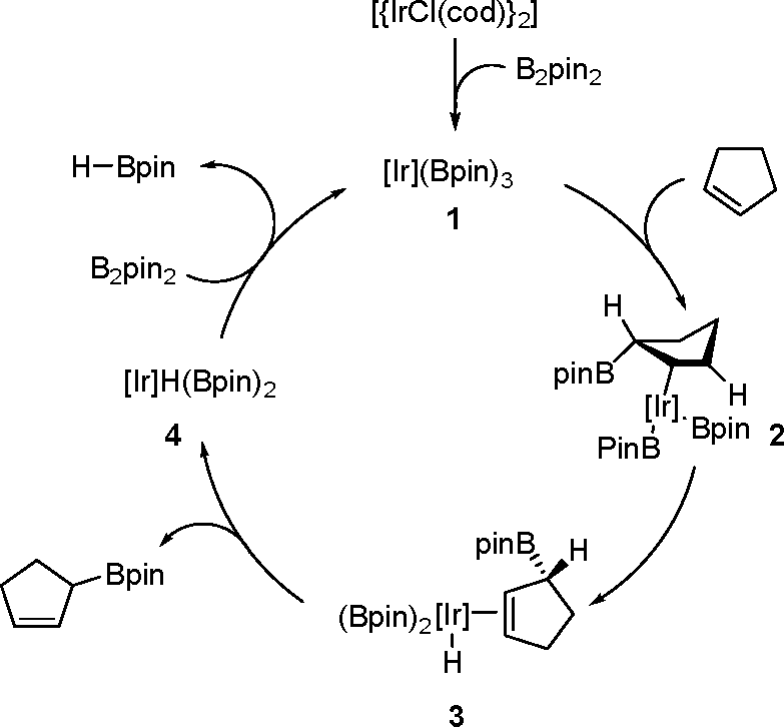
Mechanism of the Ir-Catalyzed Dehydrogenative Borylation of Cyclic Olefins. Adapted with Permission from Ref (239). Copyright 2009 American Chemical Society

Although the literature does not provide the mechanism of olefin borylation supported by unquestionable evidence, the results strongly indicate that the mechanism involves olefin insertion into the M–B bond, followed by product formation and activation of the C–H bond via β–H elimination. The mechanism for the iridium-based catalytic system has been proposed by Szabo on the basis of earlier literature data^230^ and results of labeling and kinetic studies (Scheme 94).^239^

According to this mechanism, the starting iridium(I) complex [{IrCl(cod)}_2_] reacts with an excess of B_2_pin_2_, providing an active triboronate catalyst (**1**). Association of the cycloalkene and its migratory insertion into the iridium–boron bond produces **2**, which subsequently undergoes β-H elimination to give **3**. Subsequently, the allylic product dissociates, and the iridium–hydride complex (**4**) reacts with the diboronate, thus regenerating the active catalyst and producing pinacolborane. The formation of the allyl derivative instead of the expected vinylboronate is a consequence of the stereochemistry of the β-H-elimination of hydrogen.^239^ Dehydrogenative borylation of acyclic olefins proceeds via an analogous mechanism.^239^ Similar mechanisms have been proposed for rhodium complexes^235^ and palladium–pincer complexes.^242^ However, it should be noted that an alternative mechanism has been proposed in the literature, which assumes oxidative addition of the olefinic C–H bond to the metal center.^241^

**Scheme 95:**
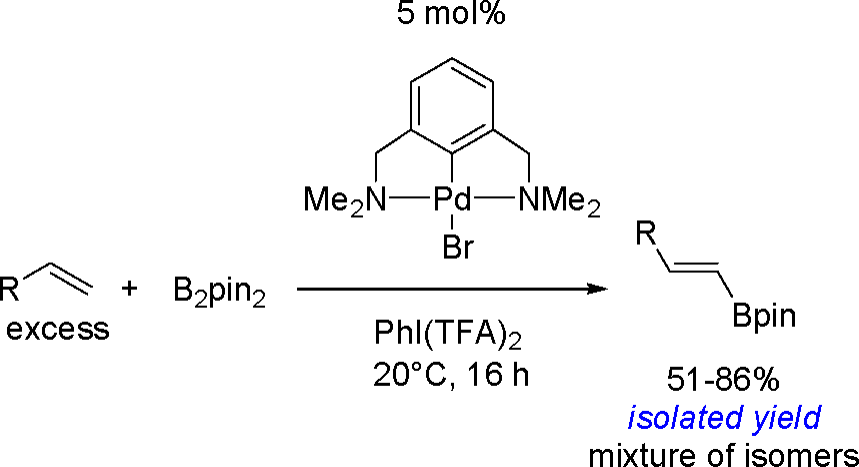
Dehydrogenative Borylation of Olefins with Bis(pinacolato)diboron Catalyzed by a Palladium NCN-Pincer Complex

Efficient palladium-catalyzed olefin borylation has first been described by Szabo and co-workers.^243^ Cycloolefins, allyltrimethylsilane, and vinylboronic acid pinacol ester undergo efficient borylation with B_2_pin_2_ in the presence of a palladium pincer complex and [bis(trifluoroacetoxy)iodo]benzene as oxidant (Scheme 95). Cycloolefin borylation leads to a mixture of the target product and an allyl isomer, while the ratio of isomers depends on the size of the cycloolefin ring.

Borylation of allyl derivatives of the general formula R^1^R^2^CHCH=CH_2_ in a catalytic system containing selectfluor as an oxidant enables efficient synthesis of allylboronates.^244^

The proposed reaction mechanism involves the formation of a palladium(IV) complex which undergoes transmetalation with diboronate, olefin insertion into the palladium–boron bond, and finally reductive elimination.

Another monoborylpalladium complex with an anionic PSiP-pincer ligand was used by Iwasawa.^242^ The reaction leads to the selective synthesis of 1,1-diboryl- or monoborylalkenes depending on the amount of diborane used without sacrificial hydroboration. Contrary to the system described by Szabo, the reaction proceeds in oxidant-free conditions through a monoborylpalladium complex bearing an anionic PSiP-pincer ligand as the key intermediate.

Finally, *N*-heterocyclic carbene copper complexes [(NHC)Cu(O*t*-Bu)], where NHC = IPr = (1,3-bis(2,6-diisopropylphenyl)imidazol-2-ylidene) or NHC = 1,3-bis(2,4,6-trimethylphenyl)-3,4,5,6-tetrahydropyrimidin-2-ylidene) were found to be catalytically active in the borylation of styrenes.^245^ The method provides access to trisubstituted vinylboronates. The protocol involves addition of a ketone which is to react with the hydride complex and enables reformation of the boronate complex, the proposed key intermediate. The type of catalyst used, the ketone, and the pattern of substituents at the double bond affect the reaction yield.

A copper salt (CuSCN, 10 mol %), with a specific phosphine (CyJohnPhos or Xantphos (22 mol %)) and TEMPO as a mild oxidant, has been reported to catalyze the borylation of terminal aryl and aliphatic alkenes with B_2_pin_2_.^246^ This approach enables direct functionalization of both aryl- and alkyl-substituted terminal alkenes. Mechanistic studies indicate a radical pathway of the reaction.

#### 3.1.3 Dehydrogenative Borylation of Arene C(sp^2^)–H Bonds

**Scheme 96:**
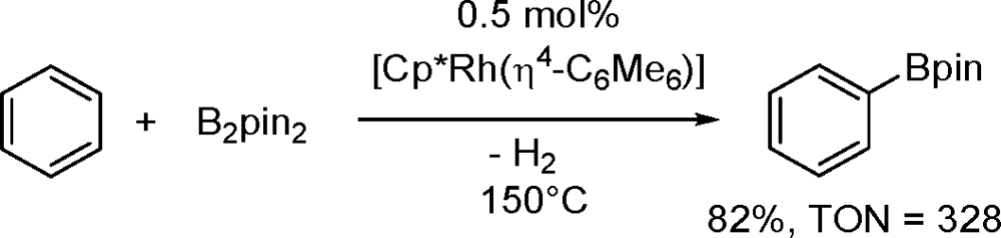
Rhodium-Catalyzed Undirected Dehydrogenative Borylation of Arene with B_2_pin_2_

Borylation of the arene C(sp^2^)–H bond—a method that has been greatly developed over the past two decades—provides a rapid and versatile route to synthetically useful (hetero)arylboronic esters. A series of catalytic systems are available that permit effective undirected, directed, and intramolecular borylation of arenes. The complexes of many TMs (iridium, rhodium, ruthenium, palladium, platinum, manganese, iron, cobalt, and nickel) and rare-earth metals have been demonstrated to exhibit catalytic activity in this process. The research on the process has been described in excellent review papers.^224,230,247^ The success story of arene borylation begins with the observation that photochemically activated [FeCp(CO)_2_Bcat] (cat = catecholate) reacts with benzene to form PhBcat.^248^ The first thermally activated catalytic borylation was reaction of benzene solvent with B_2_pin_2_ in the presence of [Cp*Rh(η^4^-C_6_Me_6_)], leading to the formation of PhBpin with TON > 300 (Scheme 96).^249^

**Scheme 97:**
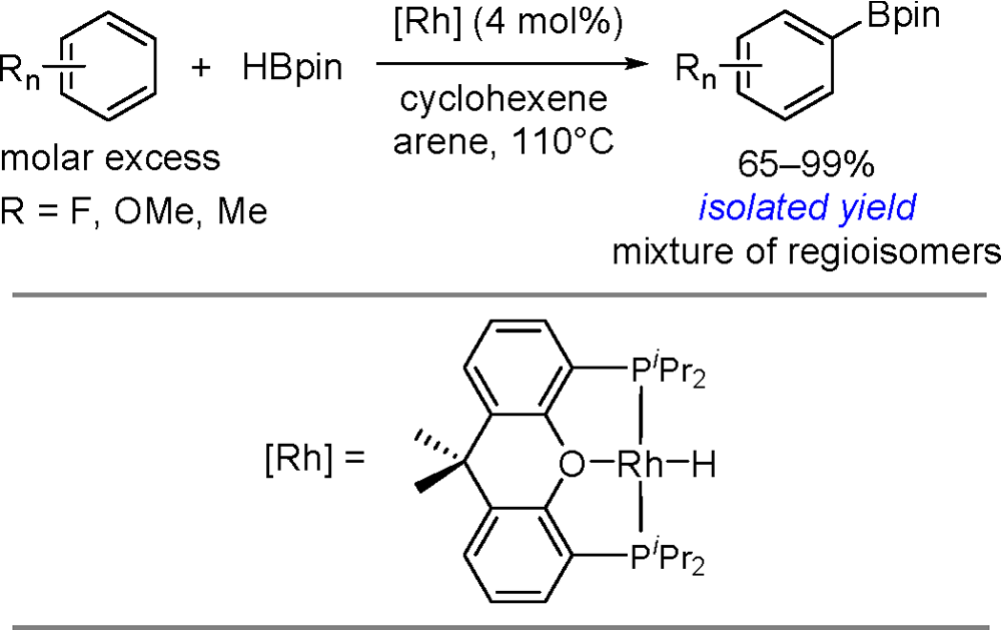
Arene Borylation with HBpin Catalyzed by a Rhodium POP-Pincer Monohydrido Complex

Currently, rhodium catalysts have been less used as catalysts of the C–H bond borylation of arenes. Since 2006, rhodium complexes have been used in the *ortho*-borylation of arylphosphines,^250^ SCF_3_-functionalized arenes,^251^ pyridylarenes,^252^ and *N*-(2-pyridyl)-2-pyridone.^253^ Sawamura reported silica-supported bridgehead phosphine–Rh systems for the C–H borylation of a large variety of arenes assisted by nitrogen-based directing groups.^254^ A POP-pincer rhodium hydride [RhHL] (Scheme 97) was found to catalyze the borylation of methyl-, methoxy-, and fluoro-substituted arenes. This complex serves as a model for studying the stoichiometricsteps of arene borylation with HBpin and permits isolation of the key intermediates.^255^

**Scheme 98:**
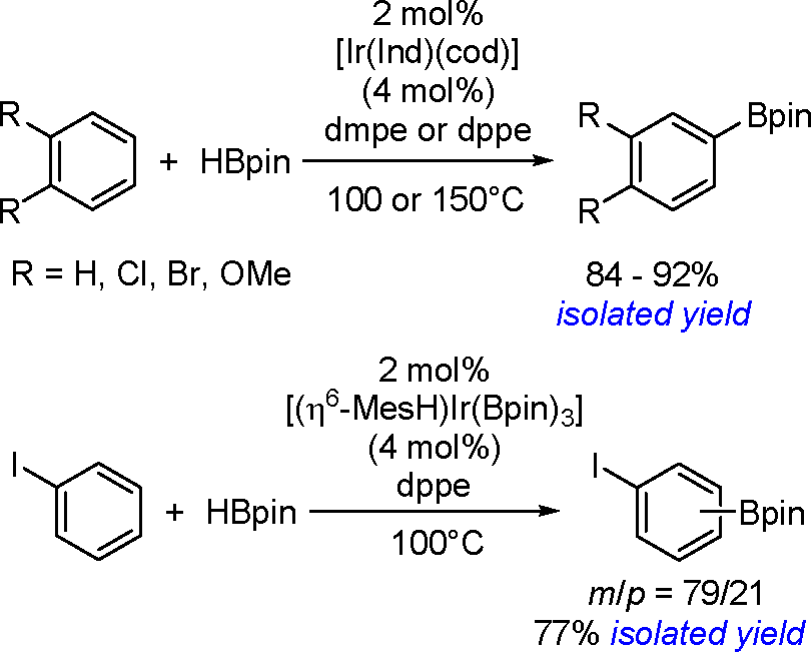
Iridium-Catalyzed Undirected Dehydrogenative Borylation of Arenes with HBpin

Presently, the most successful group of arene borylation catalysts are those based on iridium. The catalytic activity of iridium complexes was shown by Smith who reported indirect borylation of benzene^256^ and aryl derivatives^257^ with pinacolborane in the presence of [Cp*Ir(PMe_3_)(H)(Ph)]. Initial results were soon improved using a system consisting of [(Ind)Ir(cod)] and a bulkier and more stable bidentate phosphine, that is, 1,2-bis(diphenylphosphino)ethane (dppe) or 1,2-bis(dimethylphosphino)ethane (dmpe) (Scheme 98).^258^ The system enables borylation of benzene and selected mono- and disubstituted aryl compounds with pinacolborane (HBpin), leading to the highly efficient and selective formation of corresponding arylboronates.

**Scheme 99:**
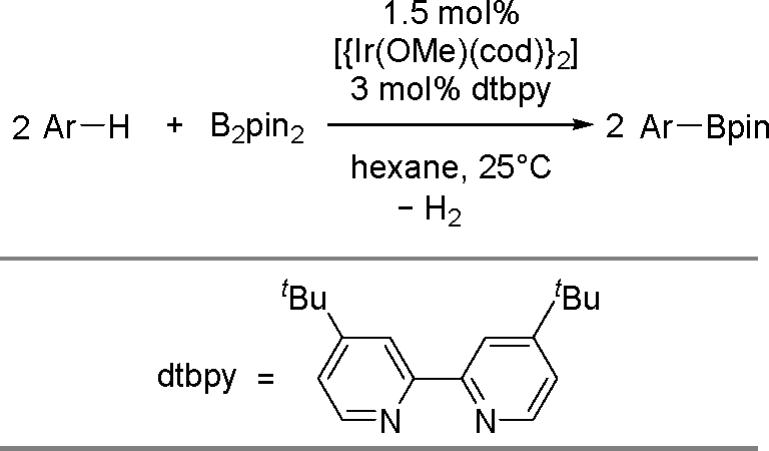
Undirected Dehydrogenative Borylation of Arenes with Pinacolborane

Subsequently, Ishiyama and Miyaura and Hartwig described an analogous system of [{Ir(OMe)(cod)}_2_] and dtbpy (dtbpy = 4,4′-di(*tert*-butyl)-2,2′-bipyridine) for the borylation of benzene and aryl derivatives with B_2_pin_2_ at room temperature.^259^ Currently, [{Ir(OMe)(cod)}_2_] is the most common catalyst for the borylation of arenes with B_2_pin_2_ in combination with dtbpy developed by Ishiyama, Miyaura, and Hartwig.^260,261^ This complex ensures high yields of arylboronates already at room temperature and tolerates the presence of several functional groups in the reagents (Scheme 99).

Recently, a combination of [{Ir(OMe)(cod)}_2_] and Me_4_phen (3,4,7,8-tetramethyl-1,10-phenanthroline) has been shown to exhibit increased reactivity in the borylation of alkanes and some arenes.^262^ The proper choice of the chelating ligand may be crucial for obtaining high yields and site selectivity of C–H borylation.^263^ Krska, Maleczka, Smith, and co-workers published a tutorial on the practical applications of iridium-based arene borylation.^262^

**Scheme 100:**
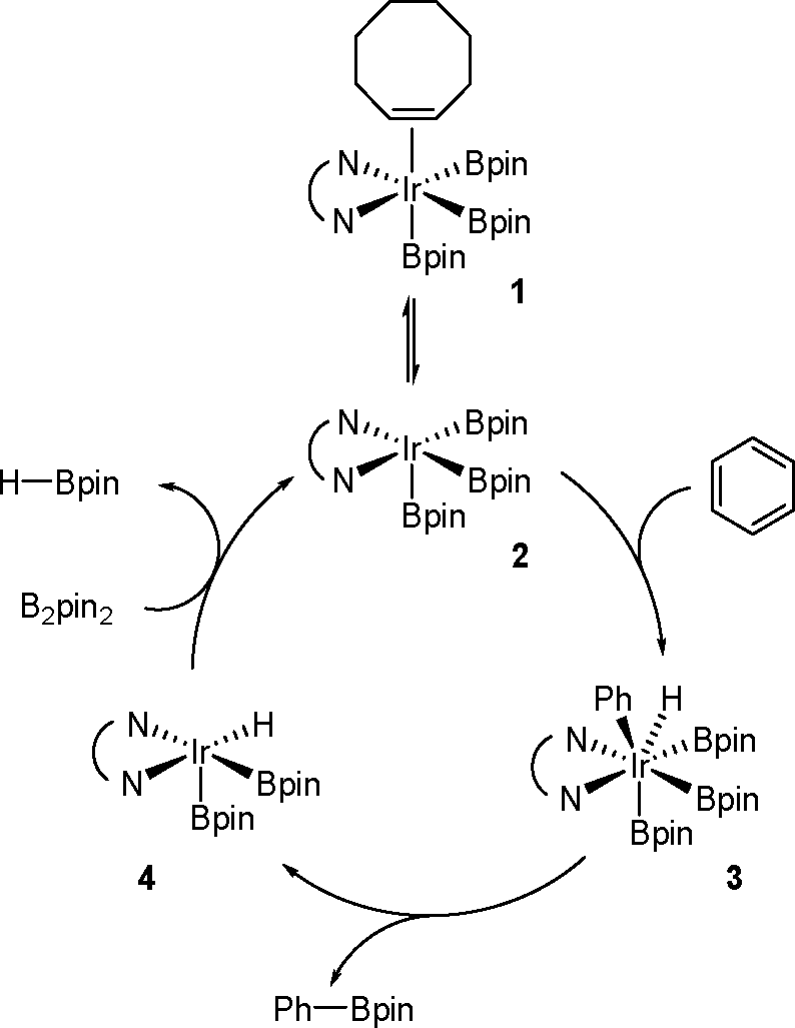
Mechanism of the Ir-Catalyzed Dehydrogenative Borylation of Arene C–H Bonds with B_2_pin_2_. Adapted with Permission from Ref (264). Copyright 2005 American Chemical Society

The mechanism of the process with the involvement of iridium catalysts has been proposed by Hartwig (Scheme 100).^264^ The mechanism assumes that the iridium(III)–tri(boronate) complex, [Ir(Bpin)_3_(bpy)], (**2**) is the active catalyst. The C–H bond is activated as a result of oxidative addition to the iridium(III) complex, with the formation of a seven-coordinate iridium(V) complex (**3**), which then undergoes reductive elimination to form **4**.

Bis(pinacolato)diboron (when present in excess) readily oxidatively adds to **4**, and the subsequent reductive elimination of HBpin regenerates complex **2**. Results of DFT calculations confirmed the proposed mechanism, in particular the potential oxidative addition of the C_aryl_–H bond to the iridium(III) complex with formation of an iridium(V) complex (**3**), and the pathway from the [{IrCl(cod)}_2_] precursor to the active complex (**2**).^265^ Complex **1**, which is an olefin-stabilized catalyst in the process, has been synthesized and characterized by spectroscopic and X-ray diffraction methods.^259^ Complex **1** has also been used in kinetic studies whose results, together with those of DFT calculations, have proved that the reaction rate is determined by the oxidative addition of arene and the reductive elimination of arylborane. The heteroarene borylation reaction proceeds according to a similar mechanism. However, heteroarenes could be associated with a catalyst and their binding strength determines the resting state.^266^

The selectivity of undirected arene C–H borylation is determined by steric effects; that is, borylation occurs at the least sterically crowded position. In contrast, the regioselectivity of heteroarene borylation is controlled mainly by electronic effects.^228^ Regioselectivity of C–H functionalization remains a challenge in synthetic chemistry. A number of procedures for the selective functionalization of arenes in the *ortho*-position have been described. The use of directing groups in C–H borylation has recently been summarized.^225,267^ Meta and para C–H borylation remains more challenging.^268,269^ Palladium complexes have been used in the directing group-assisted *ortho*-borylation of acetanilides, *N*-arylanilides, 2-(thiomethyl)phenyl, and 2-pyridyl ferrocenyl rings.

Ruthenium catalysts have been used in the directing group-assisted *ortho*-selective borylation of arenes. N-Donor groups, that is, pirydyl,^270,271^ pyrazolyl,^272^ and imine groups,^273^ were most frequently used. In addition, the Ru-catalyzed directed ortho-borylation of arylphosphines,^274^ arylphosphinites and phosphites,^275^ and arylimides^276^ was described. Moreover, Murata and co-workers reported undirected borylation of arenes with pinacolborane in the presence of a ruthenium complex prepared *in situ* from [{RuCl_2_(p-cymene)}_2_] and TpMe_2_K (TpMe_2_ = hydrotris(3,5-dimethylpyrazolyl)borate).^277^

**Scheme 101:**
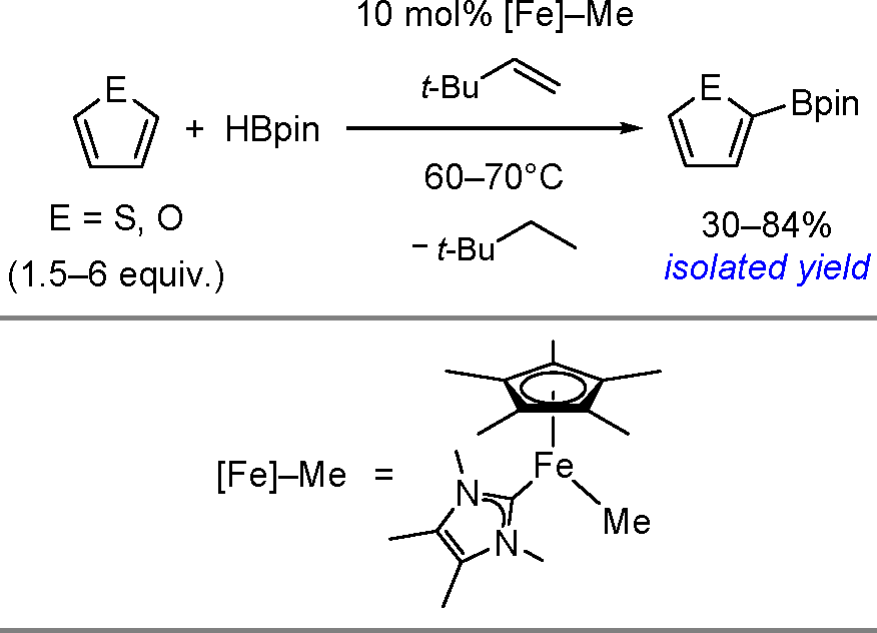
Iron-Catalyzed Dehydrogenative Borylation of Heteroarenes in the Presence of Pinacolborane, with 3,3-Dimethylbut-1-ene Acting as a Hydrogen Acceptor

The first iron-catalyzed C–H borylation of heteroarenes with HBpin was reported in 2010 (Scheme 101).^278^ A half-sandwich iron *N*-heterocyclic carbene (NHC) complex was found to be catalytically active in the C–H bond borylation of furans and thiophenes with pinacolborane.

[FeMe_2_(dmpe)_2_] used under UV irradiation (350 nm) was the first iron-based catalyst of arene borylation.^279^ Variously substituted arenes can undergo undirected borylation at ambient temperatures with isolated yields of 25–73%. The [Fe(H)(Bpin)(dmpe)_2_] hydrido(boryl)iron complex has been isolated and shown to be a competent catalyst of the process. FeBr_3_ has been found to catalyze the directed *ortho*-borylation of pyridylarenes with a 9-borabicyclo[3.3.1]nonane dimer (9-BBN dimer).^280^ Borylated products were obtained in 50%–74% yield with good functional group tolerance. A plausible mechanism operating via the Lewis acid–base interaction of 2-phenylpyridines 1 and 9-BBN and iron-catalyzed C–H bond activation has been proposed.

Mankad, Keith and co-workers have elucidated the mechanism of the photochemical C–H borylation in the presence of heterobimetallic Cu–Fe catalysts.^281^ A Ni-based catalytic system ([Ni(cod)_2_]/[ICyH]^+^Cl^–^/NaO(*t*-Bu)) (ICy = 1,3-dicyclohexylimidazol-2-ylidene) catalyzes the borylation of arenes and indoles with HBpin at 100 °C.^282^ Preliminary investigation of the reaction mechanism suggests that heterogeneous nickel species are likely to be the catalytically active forms.

**Scheme 102:**
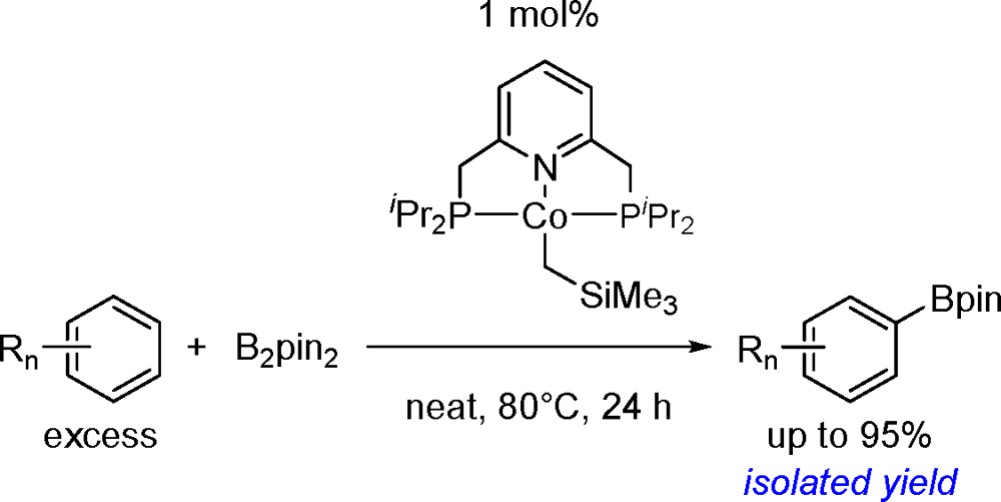
Undirected Arene Borylation with B_2_pin_2_ Catalyzed by a Cobalt–PNP-Pincer Complex

PNP-pincer cobalt(I) complexes developed by Milstein have been found to be active in the catalytic borylation of aryl C–H bonds. Chirik group reported the high catalytic activity of these complexes in the borylation of variously substituted arenes and heteroarenes with HBpin or B_2_pin_2_ (Scheme 102).^283^

**Scheme 103:**
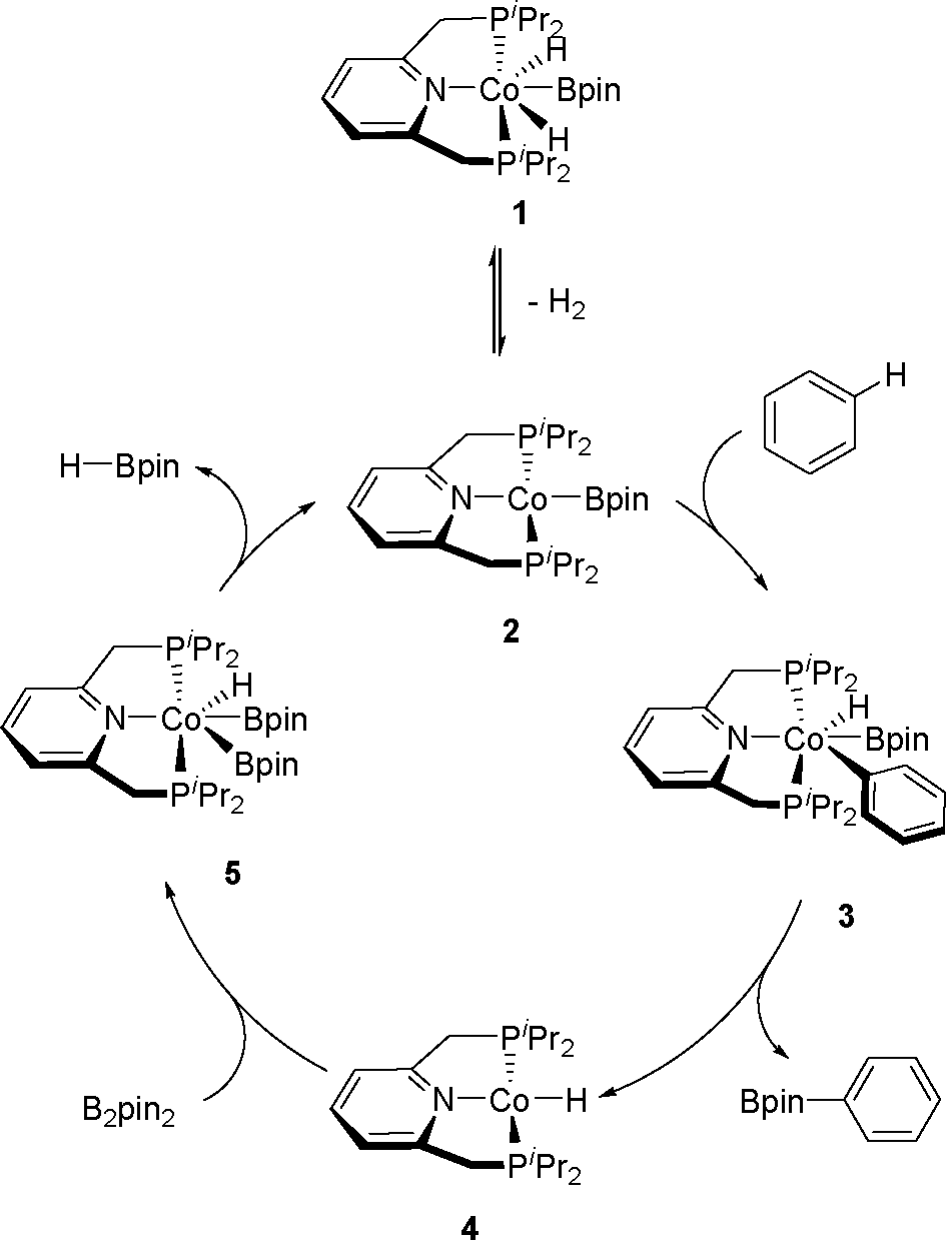
Mechanism of the Co-Catalyzed Dehydrogenative Borylation of Arenes with B_2_pin_2_^286^

As with other undirected functionalization reactions, regioselectivity depends on the arene substitution pattern. In subsequent papers,^284−286^ the catalytic studies were supplemented with mechanistic research on the isolation of catalytic intermediates, stoichiometric experiments, and kinetic measurements. On the basis of the results, a mechanism of arene borylation in the presence of a cobalt bis(phosphine)pyridine pincer complex was proposed. Borylation of the benzene C–H bond with B_2_pin_2_ catalyzed by cobalt–pincer complexes was also included in DFT calculations.^286^ The suggested mechanism (Scheme 103) involved three distinct steps: oxidative addition of C_6_H_6_, reductive elimination of PhBpin, and regeneration of the catalytically active Co(I)–boronate complex. The *trans*-[(PNP)CoH_2_(Bpin)] complex was experimentally observed as the resting state in the borylation of five-membered ring heteroarenes. The active catalyst of this complex can be generated by reductive dihydrogen elimination, preceded by the rearrangement of the complex into a *cis*-isomer. A boronate complex is proposed as the active catalyst (**2**). Oxidative addition of the benzene C–H bond to **2** forms a six-coordinate cobalt(III) complex (**3**) which subsequently undergoes reductive elimination of PhBpin (**4**). Finally, the sequence of the oxidative addition of B_2_pin_2_ and the reductive elimination of HBpin results in the regeneration of the active species (**2**).^286^

#### 3.1.4 Dehydrogenative Borylation of C(sp^3^)–H Bonds

**Scheme 104:**
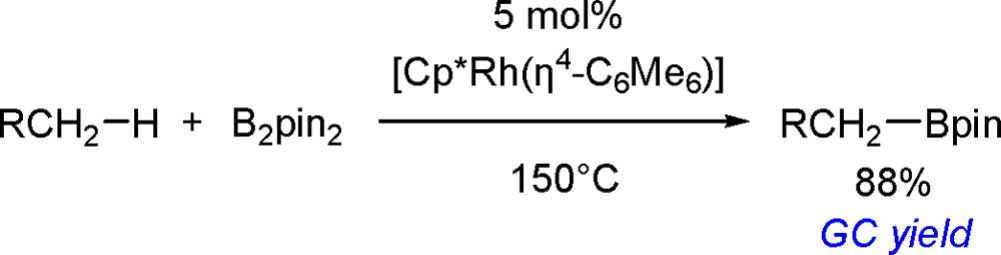
Dehydrogenative Borylation of Linear Alkanes

Activation and functionalization of the C(sp^3^)–H bond, especially a site-selective process, remains a challenge in catalysis.^287^ In 1999, the Hartwig group reported the catalytic functionalization of alkanes at primary C–H bonds in the presence of a rhenium complex [Cp*Re(CO)_3_] under photochemical activation^288^ and the first regiospecific catalytic borylation of linear alkanes under thermal conditions (Scheme 104).^249^

**Scheme 105:**
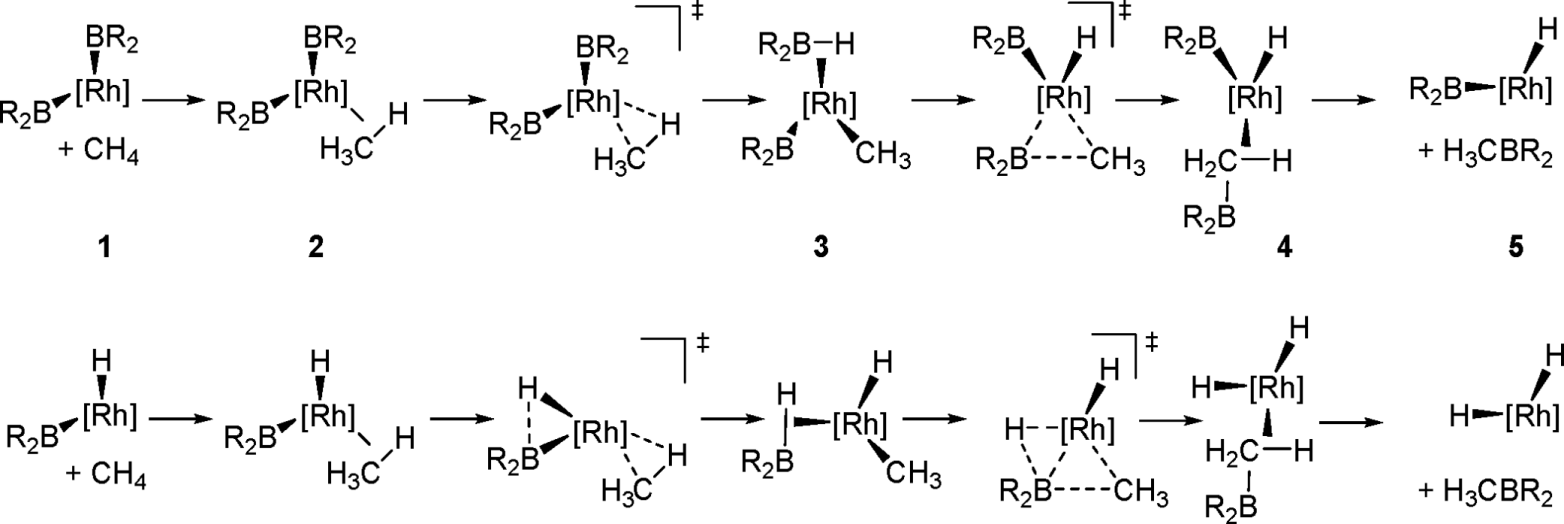
Mechanism of C–H Activation and C–B Formation according to the Results of DFT Calculations for [CpRhH(BR_2_)] (a) and [CpRh(BR_2_)_2_] (b) Complexes (BR_2_ = 1,3-Dioxaborolane). Reprinted with Permission from Ref (289). Copyright 2005 American Chemical Society

Selective borylation of the least congested terminal methyl groups of linear and branched alkanes, alkyl ethers, linear and cyclic tertiary amines, and partially fluorinated alkanes with bis(pinacolato)diboron or pinacolborane has been achieved in the presence of [Cp*Rh(C_2_H_4_)_2_] and [Cp*Rh(η^4^-C_6_Me_6_)]. Other rhodium complexes, such as [Cp*Rh(η^4^-C_6_Me_6_)], [Cp*Rh(H)_2_(SiEt_3_)_2_], and [Cp*Rh(C_2_H_3_SiMe_3_)_2_], also showed catalytic activity in the borylation of octane with B_2_pin_2_. In contrast, the tested iridium complexes, [Cp*IrH_4_] and [Cp*Ir(C_2_H_4_)_2_], showed much lower catalytic activity. [Cp*Rh(η^4^-C_6_Me_6_)] gave the highest yield of octane borylation of 88% (Scheme 109). According to the proposed reaction mechanism, the catalytically active species [Cp*Rh(H)_*x*_(Bpin)_4–*x*_] was generated by the dissociation of hexamethylbenzene and the addition of a combination of HBpin and B_2_pin_2_. It was possible to isolate [Cp*Rh(H)_2_(Bpin)_2_] from the reaction of the starting [Cp*Rh(η^4^-C_6_Me_6_)] with HBpin under photochemical conditions. Moreover, [Cp*Rh(H)(Bpin)_3_] was isolated from the reaction of [Cp*Rh(H)_2_(Bpin)_2_] with HBpin. Both complexes reacted with octane to yield octylBpin. The yields and reaction rates imply that [Cp*Rh(H)_2_(Bpin)_2_] and [Cp*Rh(H)(Bpin)_3_] are chemically and kinetically competent to be intermediates in the borylation of alkanes. Energetic profiles of methane borylation with diboron bispinacolate have been calculated by DFT. In line with the DFT calculations, the dissociation of HBpin from complexes **2** and **3** is faster than the dissociation of H_2_ or B_2_pin_2_ from these complexes. On the basis of the calculation results, the mechanism of activation of the methane C–H bond in the presence of boryl–rhodium complexes as well as the mechanism of the reductive elimination of methylborane were proposed (Scheme 105).^289^

The reaction of a diborate complex (**1**) with methane occurs via the coordination of an alkane to the boryl complex to form a σ-alkane complex (**2**), followed by its transformation into a σ-pinacolborane complex (**3**). Subsequently, the methyl group undergoes bond formation with the second boryl group to form MeBpin. An analogous reaction of methane with pinacolborane proceeds via a similar mechanism involving formation of the σ-alkane complex and breaking of the C(sp^3^)–H bond with the simultaneous formation of the σ-borane complex. Product formation proceeds through a multicenter transition state. DFT calculations suggest that the processes of C–H bond cleavage and B–C bond formation run via a metal-assisted σ-bond metathesis mechanism. The calculations have shown a specific role of boron in the activation of C–H bonds in alkanes and arenes. According to Hartwig and Hall,^289^ the presence of an unoccupied p orbital in boron and the interaction of the boryl ligand with the hydride located *cis* to it is crucial for the activation of the C–H bond in alkenes. The thermodynamic driving force of the reaction is the formation of the B–C bond, while the unoccupied orbital in boron reduces the energy barrier for C–H bond activation and formation of the borylated product.

**Scheme 106:**
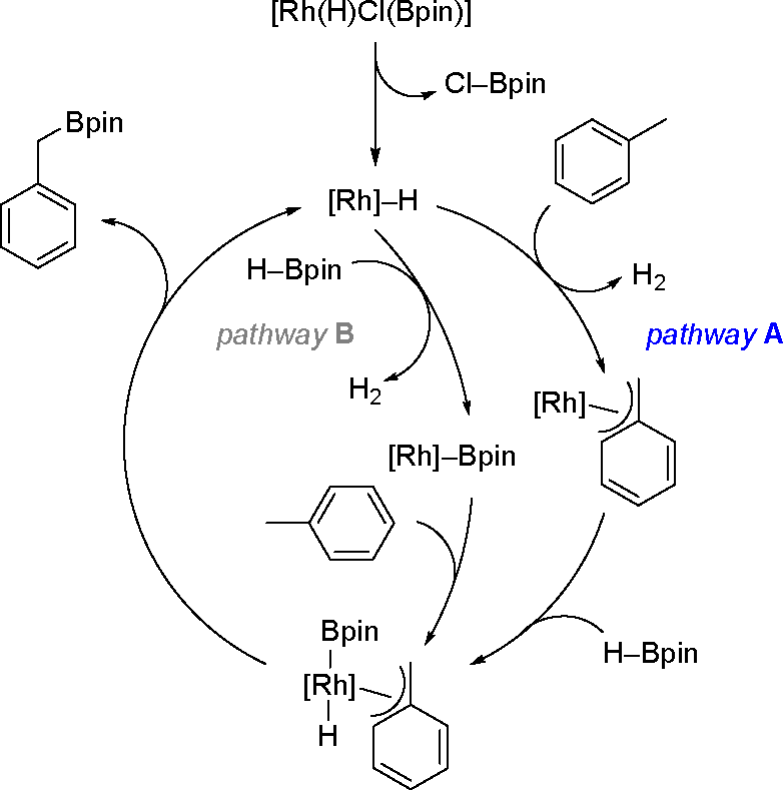
Mechanism for the Rh-Catalyzed Borylation of the Benzylic C–H Bond with HBpin^290^

Efficient borylation of the benzylic C–H bond has first been reported by Marder and co-workers.^290^ In the presence of [RhCl(P*i*-Pr_3_)_2_(N_2_)], toluene, *p*-xylene, and mesitylene have been shown to react with HBpin. Benzylic monoboration was accompanied by diboration and aromatic borylation. Highly selective borylation at the benzylic position compared with the aryl position was observed in the reaction with *para*-xylene. On the basis of the investigation of stoichiometric reactions, a mechanism of the process was proposed, assuming the [Rh(H){P(*i-*Pr)_3_}_2_] rhodium hydride as the active catalyst. Scheme 106 presents two proposed mechanistic pathways.

**Scheme 107:**
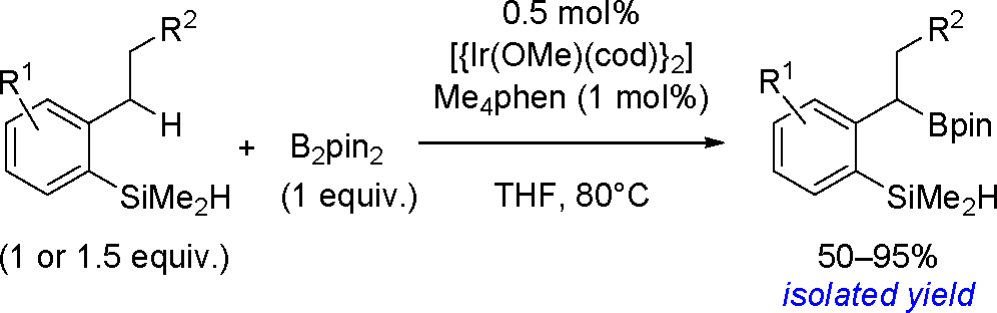
Silyl-Directed Dehydrogenative Borylation of Secondary Benzylic C–H Bonds

DFT calculations by Lin and Marder for a slightly simplified model indicate that the activation of the benzylic C–H bond is the rate-limiting step and that it occurs more favorably in the coordination sphere of [L_2_Rh(H)] (pathway **A**, Scheme 106).^291^ Secondary benzylic C–H bonds were selectively borylated in the presence of a hydrosilyl directing group and an iridium complex (Scheme 107).^292^

**Scheme 108:**
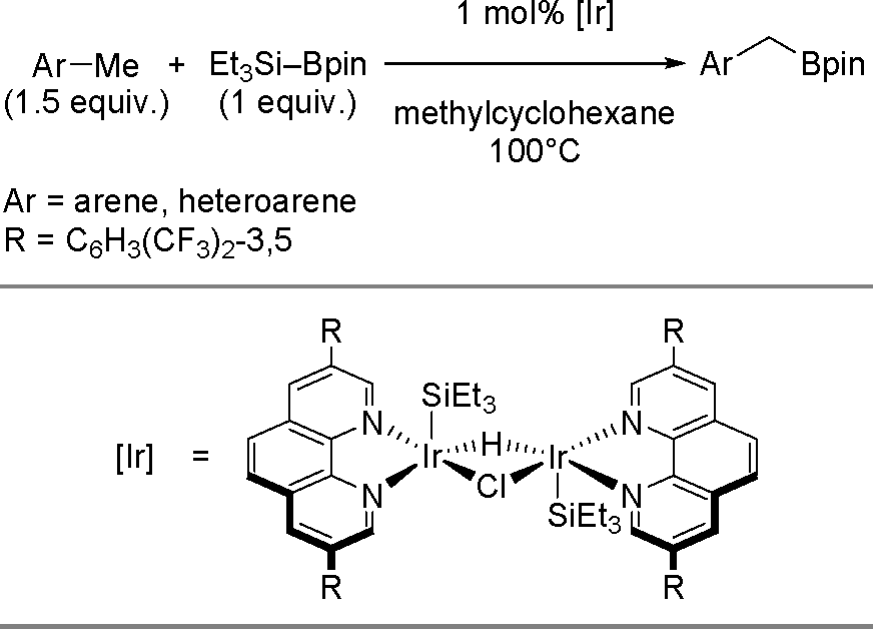
Undirected Dehydrogenative Borylation of Primary Benzylic C–H Bonds

Hartwig and co-workers reported selective borylation of the benzylic C–H bonds of methylarenes with Et_3_SiBpin as the borylating agent in the presence of an iridium complex bearing an optimized phenanthroline ligand (Scheme 108).^293^ The reaction suffered from competitive arene C–H borylation. However, optimization of the phenanthroline ligand achieved high chemoselectivity because of the high sensitivity of aryl C–H borylation to the electronic properties of the catalyst. Reactions of toluene and *meta*-substituted methylarenes resulted in the formation of benzylic boronates with moderate yields and good to excellent selectivity.

**Scheme 109:**
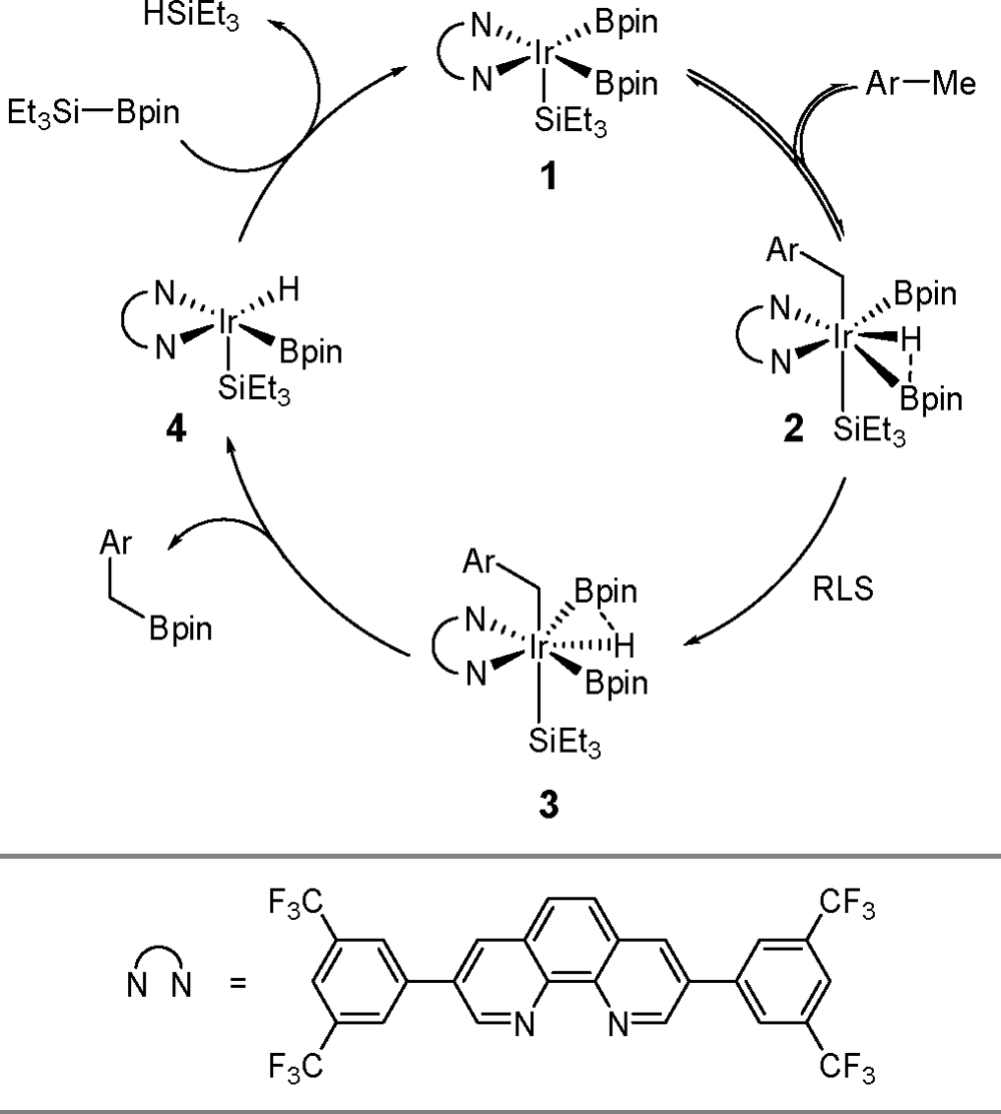
Mechanism of the Ir-Catalyzed Borylation of Benzylic C(sp^3^)–H Bonds with Et_3_SiBpin. Adapted with Permission from Ref (293). Copyright 2015 American Chemical Society

**Scheme 110:**
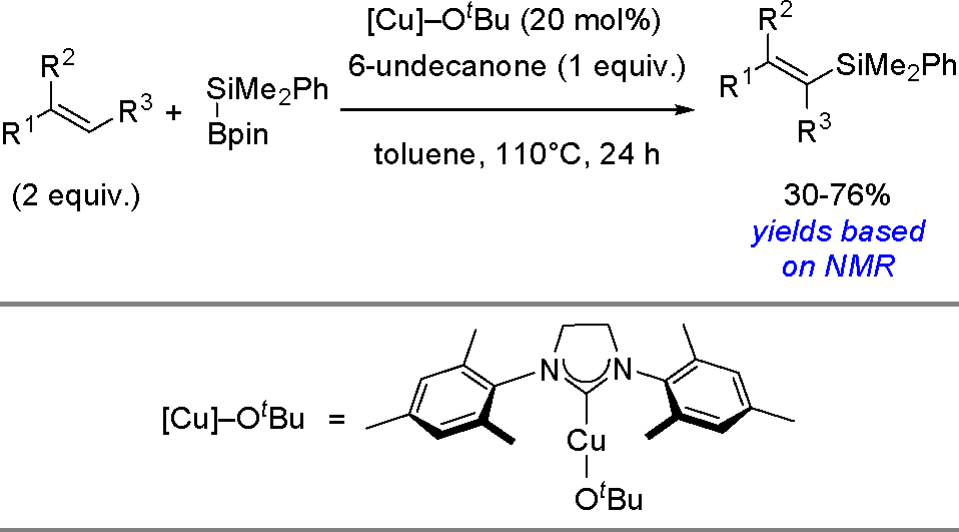
Dehydrogenative Silylation of Olefinic C(sp^2^)–H Bonds with PhMe_2_SiBpin Catalyzed by the NHC Copper Complex

The resting state of the [LIr(Bpin)_2_(SiEt_3_)] catalyst (**1**) has been identified, isolated, and characterized (Scheme 110) and has been found to be kinetically competent to act as an intermediate in the reaction. The iridium–diborylsilyl complex has been demonstrated to be more electron-deficient than the trisboryl complexes used for C–H borylation. It has been shown that a reduction in the electron density in the metal leads to a decrease in the borylation rate of the aryl C–H bond that is much greater than the decrease in the borylation rate at the benzyl position, which enhances reaction selectivity. On the basis of the isolation of the resting state of the catalyst, analysis of stoichiometric reactions, kinetic studies, and DFT calculations, a reaction mechanism has been proposed involving reversible oxidative addition of the benzylic C–H bond to [LIr(Bpin)_2_(SiEt_3_)], rate-limiting isomerization, and reductive elimination of the benzylic boronate ester (Scheme 109). The reaction of **4** with Et_3_SiBpin regenerates the catalyst and HSiEt_3_ forms.

Mono- and polyborylation of benzylic C–H bond(s) with B_2_pin_2_ has also been reported in the presence of cobalt–diamine complexes by the Chirik group.^294^

### 3.2 Dehydrogenative Silylation of C–H Bonds

Direct silylation of C–H bonds is a well-explored class of metal-catalyzed C–H bond functionalizations. Efficient procedures have been developed for the silylation of C–H bonds in alkanes, alkenes, arenes, and alkynes. The reaction permits the synthesis of useful building blocks with a wide range of applications in organic synthesis. The current knowledge of the reaction has been presented in several review papers.^227,295−300^

#### 3.2.1 Dehydrogenative Silylation of C(sp)–H Bonds

Alkynylsilanes are important structural motifs in organic chemistry. The silyl group not only acts as a protecting group for the reactive C(sp)–H bond but also modifies the steric and electronic properties of the triple bond. Alkynylsilanes are convenient reagents for the formation of carbon–carbon and carbon–heteroatom bonds.^301^ The first selective C(sp)–H silylation of terminal alkynes with hydrosilanes was reported by Voronkov in 1980, using a combination of H_2_PtCl_6_·6H_2_O and LiI (100 equiv) as a catalytic system.^302,303^ The literature provides a few other examples of the dehydrogenative silylation of acetylenes with trisubstituted silanes (HSiR_3_) catalyzed by TM complexes. Although the mechanisms of ethynylsilane formation have been proposed (e.g., ref (304)), no detailed mechanistic studies have been carried out so far.

#### 3.2.2 Dehydrogenative Silylation of Olefinic C(sp^2^)–H Bonds

Dehydrogenative silylation with HSiR_3_ as the silylating agent was discussed in Section 1.1.1. Metal-catalyzed silylation of olefinic C(sp^2^)–H bonds with disilanes is in the early stage of development, and as yet no mechanism has been proposed. Recently, Mankand has described dehydrogenative silylation in the presence of a *N*-heterocyclic carbene–copper complex and commercially available PhMe_2_SiBpin as the silylating agent (Scheme 110).^245^

**Scheme 111:**
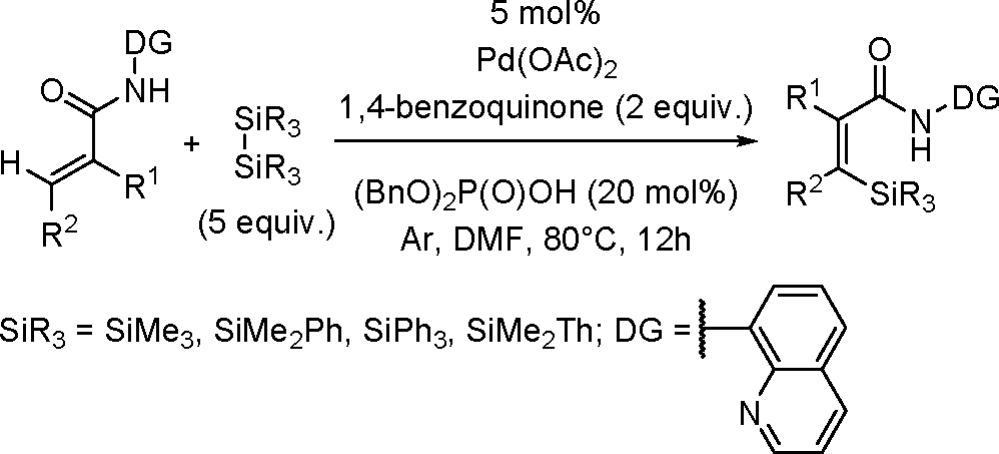
Directed Dehydrogenative Silylation of Olefins

Another publication described the activity of a palladium-based catalyst in the selective synthesis of (*Z*)-vinylsilanes via olefinic C–H silylation with disilanes (Scheme 111).^305^

#### 3.2.3 Dehydrogenative Silylation of Arene C(sp^2^)–H Bonds

The silylation of aryl C–H bonds is a suitable method for the synthesis of arylsilanes that are valuable synthetic intermediates. The known examples of the reaction can be divided into three classes: (i) intramolecular, (ii) directed intermolecular, and (iii) undirected intermolecular silylations of aryl C–H bonds.

**Scheme 112:**
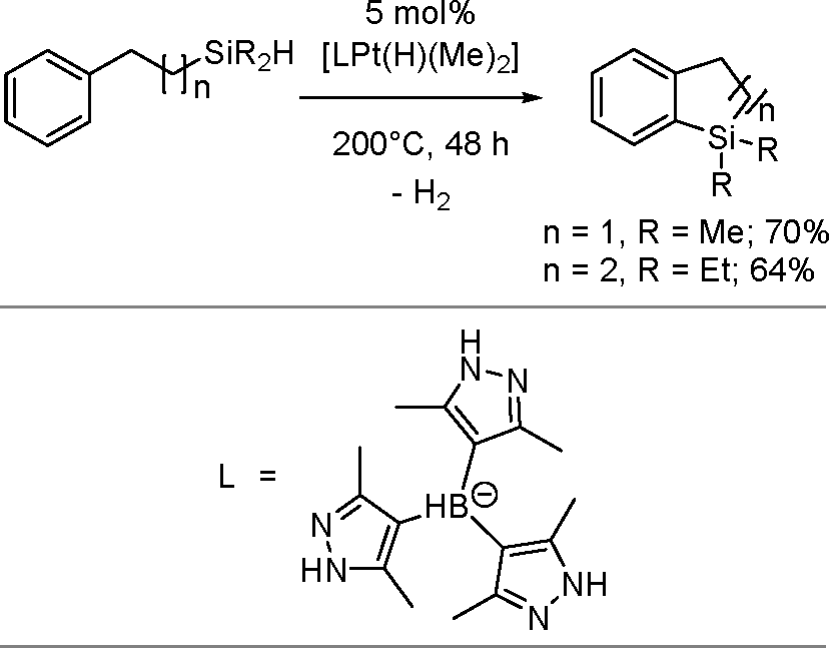
Intramolecular Silylation of Arenes

Intramolecular arene silylation leads to the formation of silacyclic compounds and it is catalyzed mainly by Rh and Ir complexes but also by Pt and Ru complexes. In 2005, Hartwig group reported the first examples of the intramolecular dehydrogenative coupling of silanes, leading to the efficient formation of both five- and six-membered organosilicon products in the presence of a platinum complex (Scheme 112).^306^

**Scheme 113:**
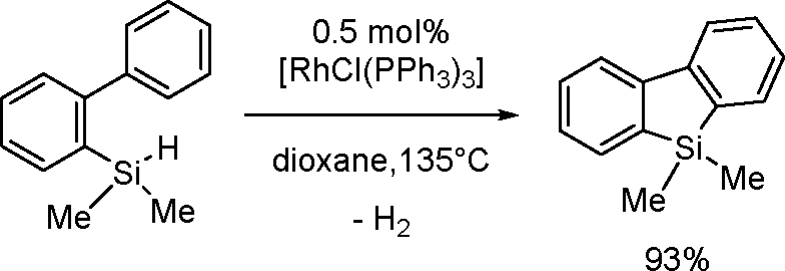
Intramolecular Silylation of 2-(Silyl)-substituted Biarene in the Presence of Wilkinson’s Catalyst

Kuninobu, Takai, and co-workers demonstrated the synthesis of silafluorene derivatives (Scheme 113) via the intramolecular silylation of [1,1′-biphenyl]-2-yldimethylsilanes in the presence of Wilkinson’s catalyst.^307^ The presence of electron-withdrawing substituents in the aromatic ring achieved a highly efficient reaction without the need for a hydrogen acceptor in the reaction system.

**Scheme 114:**
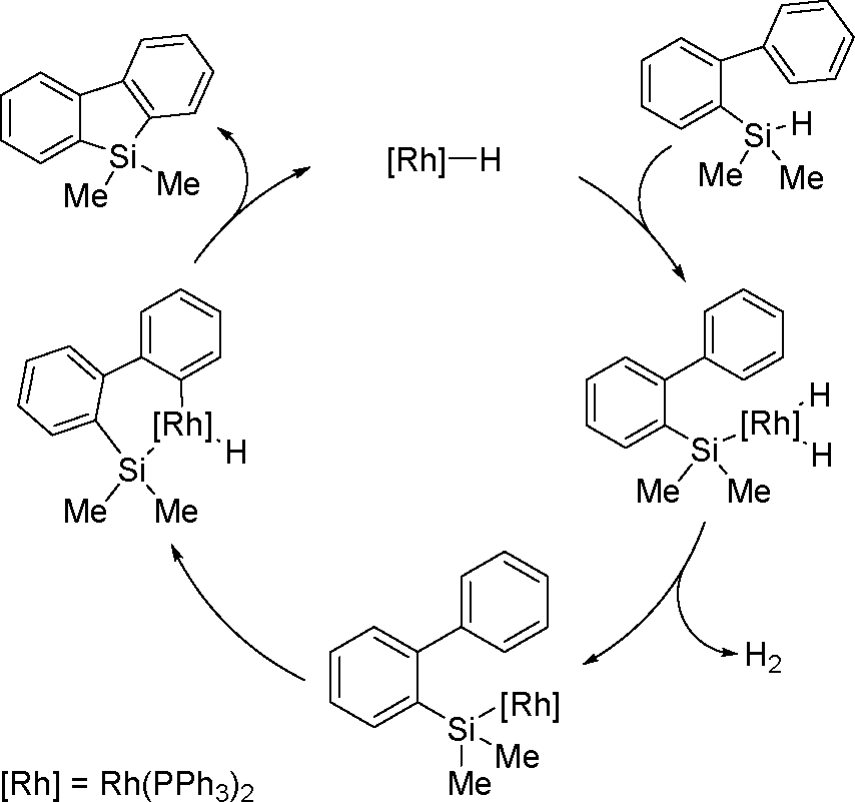
Mechanism of the Rh-Catalyzed Intramolecular Dehydrogenative Silylation of Arene C–H Bonds^308^

The reaction tolerates halides, methoxy and trifluoromethyl groups, and thiophene rings. Further research on this reaction permitted optimization of the catalytic system ([{RhCl(cod)}_2_]/6 PPh_3_) and use of milder reaction conditions.^308^ In the presence of an analogous iridium complex, a much lower yield was obtained. On the basis of a combined experimental and computational mechanistic study, Murai and Takai proposed the reaction mechanism (Scheme 114). The authors demonstrated that intramolecular dehydrogenative silylation can proceed efficiently, even without hydrogen acceptors.^308^ The reaction in the presence of a hydrogen acceptor (3,3-dimethyl-1-butene) was proved by calculations to increase the free enthalpy of the process.

The mechanism is essentially the same as that proposed by Hartwig and Cheng for undirected intermolecular silylation.^309^

**Scheme 115:**
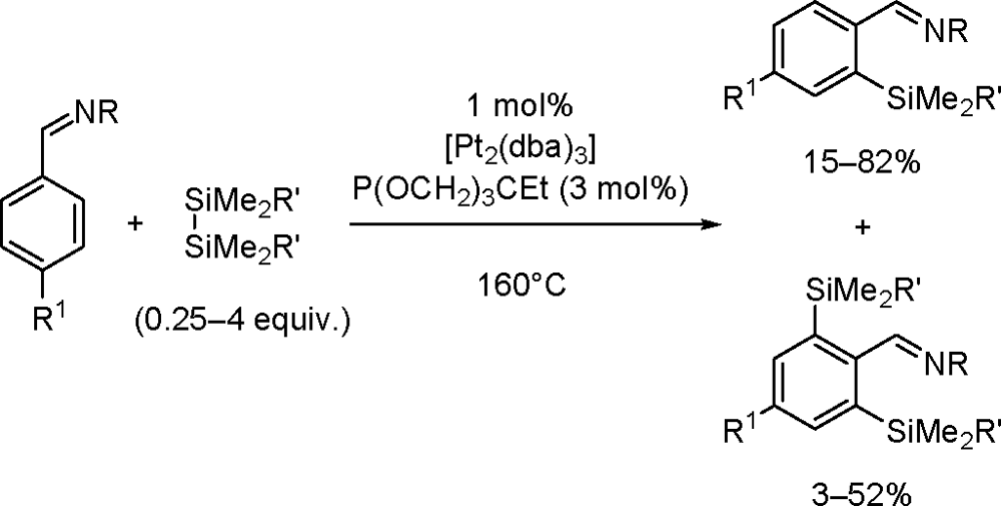
Directed Intermolecular Silylation of Arenes with Disilanes

Directed intermolecular silylation of the arene C(sp^2^)–H bond is assisted by a directing group and occurs with high regioselectivity, generally in the *ortho* position to the directing group. The first directed silylation of aryl C–H bonds catalyzed by [Pt_2_(dba)_3_] accompanied by P(OCH_2_)_3_CEt has been reported by Tanaka who described the *ortho*-silylation of benzylideneamines (Scheme 115).^310^

**Scheme 116:**
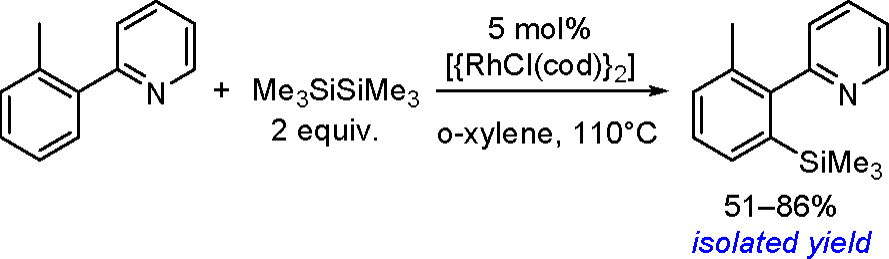
Rhodium-Catalyzed Directed Intermolecular Silylation of Arenes with Disilanes

A great contribution has been made by Murai who described the ruthenium-catalyzed directed silylation of five-membered ring heteroarenes with vinylsilanes, followed by silylation of the aryl C–H bond with tertiary silanes (HSiR_3_), applicable to a great variety of directing groups.^311,312^ Tobisu and Chatani designed the Rh-catalyzed, pyridine-directed *ortho*-silylation of aryl C–H bonds with hexamethyldisilane using a pyridyl directing group (Scheme 116).^313^

**Scheme 117:**
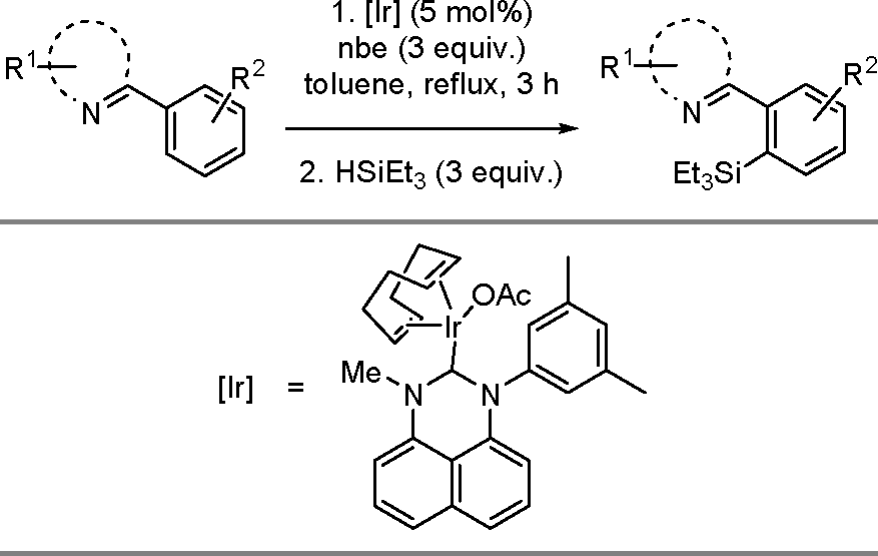
Iridium-Catalyzed Directed Intermolecular Silylation of Arenes with Tertiary Silanes

Further progress was made by Suginome,^314^ Murata,^315^ as well as Kuninobu and Kanai.^316^ Efficient silylation of heteroarenes was reported by Mashima and co-workers.^317^ The reaction occurred in the presence of an iridium complex, [Ir(NHC)(OAc)(cod)], bearing a hemilabile carbene ligand. By using a 3-fold molar excess of triethylsilane and norbornene as a hydrogen acceptor (Scheme 117), a mono-*ortho*-silylation product was obtained in good to excellent yields. In addition, excellent research has been conducted to determine the mechanism of the Ir-based directed silylation of 2-phenylpyridine in the presence of hemilabile ligands.

**Scheme 118:**
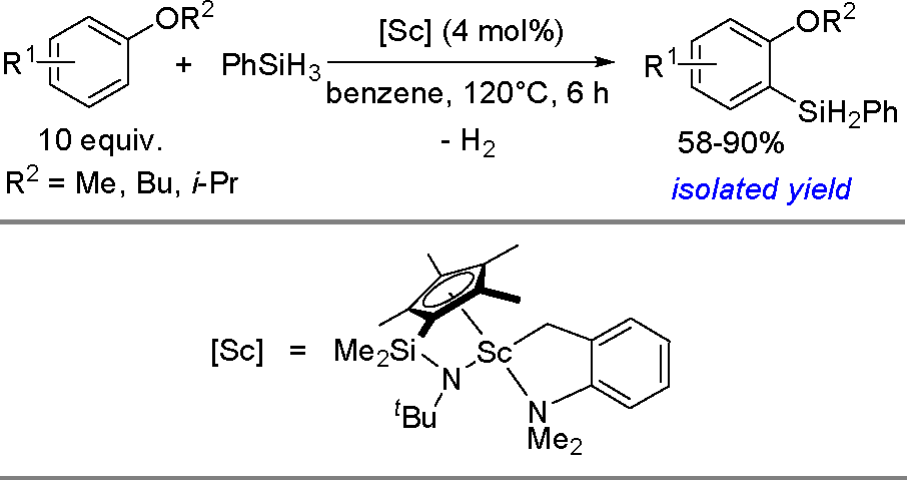
Alkoxy-Directed *ortho*-Silylation of Arenes in the Presence of a Scandium Complex

Half-sandwich scandium σ-alkyl complexes have been reported to catalyze the *ortho*-selective C–H silylation of alkoxy-substituted benzene derivatives with a primary silane (H_3_SiPh) without the need for a hydrogen acceptor (Scheme 118).^318^

**Scheme 119:**
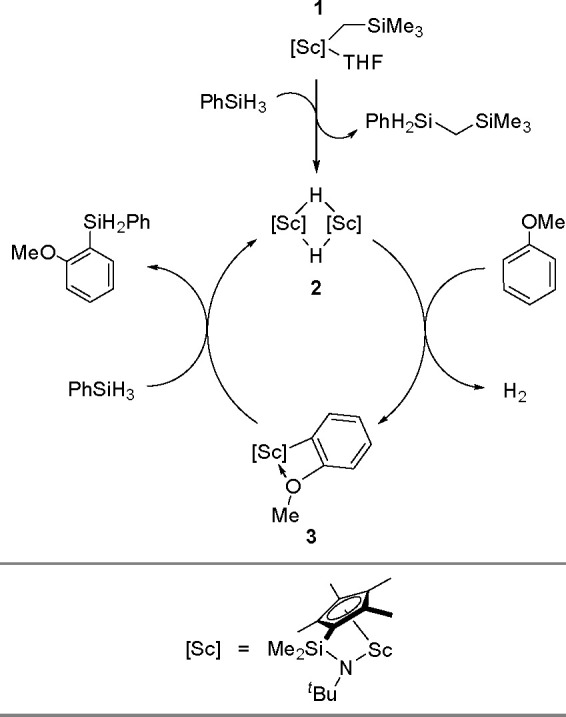
Mechanism of the Sc-Catalyzed Directed Dehydrogenative Silylation of Arene C–H Bonds with PhSiH_3_^318^

An efficient course of the reaction is observed with PhSiH_3_ as the silylating agent. The reaction tolerates functional groups such as Cl, Br, I, SMe, and NMe_2_ in the aromatic ring. The proposed mechanism of the Sc-catalyzed directed silylation of anisole is shown in Scheme 119.^318^

The precatalyst (**1**) undergoes σ-bond metathesis with H_3_SiPh to generate a dimeric Sc(II) complex (**2**) and PhSiH_2_CH_2_SiMe_3_. Complex **2** then undergoes σ-bond metathesis with the arene substrate to form **3**. Complex **3** undergoes σ-bond metathesis with H_3_SiPh to generate the silylarene product and regenerate the Sc dimer (**2**). The key intermediates (**2** and **3**) were synthesized independently, characterized by X-ray diffraction, and tested for their kinetic relevance. Noncatalytic reactions of **2** with anisole yielded **3**, accompanied by the release of hydrogen. The reaction of **3** with H_3_SiPh at 40 °C in the absence of an arene afforded complex **2** in a quantitative yield. Additional experiments suggested that the rate-limiting step was substrate coordination to the metal center and not the cleavage of the C–H bond.

**Scheme 120:**
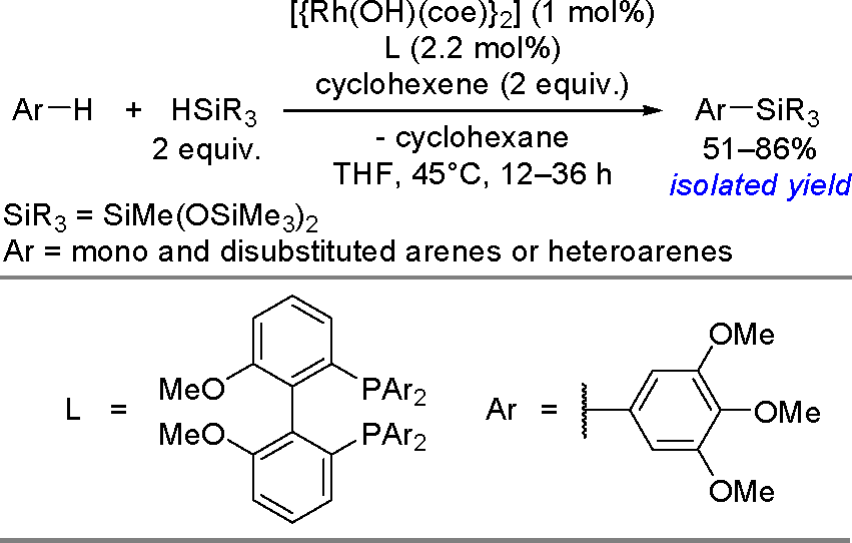
Undirected Dehydrogenative Silylation of Arenes with Tertiary Silanes

Undirected intermolecular silylation of the aryl C(sp^2^)–H bond started with the seminal work of Curtis who reported the silylation of benzene (used as a solvent) in the presence of Vaska’s complex, [IrCl(CO)(PPh_3_)_2_].^319^ Significant progress was made by Ishiyama and Miyaura who described effective silylation of arenes using *t*-BuF_2_SiSiF_2_*t*-Bu in the presence of [{Ir(OMe)(cod)}_2_]/dtbpy (dtbpy = 4,4′-di-(*tert*-butyl)-2,2′-bipyridine)^320,321^ and arene silylation with 1-hydrosilatrane in the presence of [{Ir(OMe)(cod)}_2_]/dmphen (dmphen = 2,9-dimethyl-1,10-phenanthroline).^322^ Hartwig, who reported the activity of [Tp^Me2^PtRR’H] (Tp^Me2^ = hydridotris(3,5-dimethylpyrazolyl)borate; R,R′ = H, Me, or Ph) in the silylation of benzene with triethylsilane,^296^ and Murata, who described the activity of PtCl_2_/Tp^Me2^K in the C–H silylation of arenes with HSiMe(OSiMe_3_)_2_,^323^ greatly contributed to the field. Unfortunately, all these methods suffered from the need for the high excess of the arene reacting partner and harsh reaction conditions. Currently, the most commonly used rhodium-based catalytic system for arene C–H silylation is the one proposed by Hartwig (Scheme 120).^324^ The system enables efficient silylation of the arene as the limiting reagent under mild conditions. Moreover, the reaction exhibits high sterically derived regioselectivity and tolerates several functional groups, such as halides, alkyl, alkoxy, CF_3_, and secondary amine and amide.

**Scheme 121:**
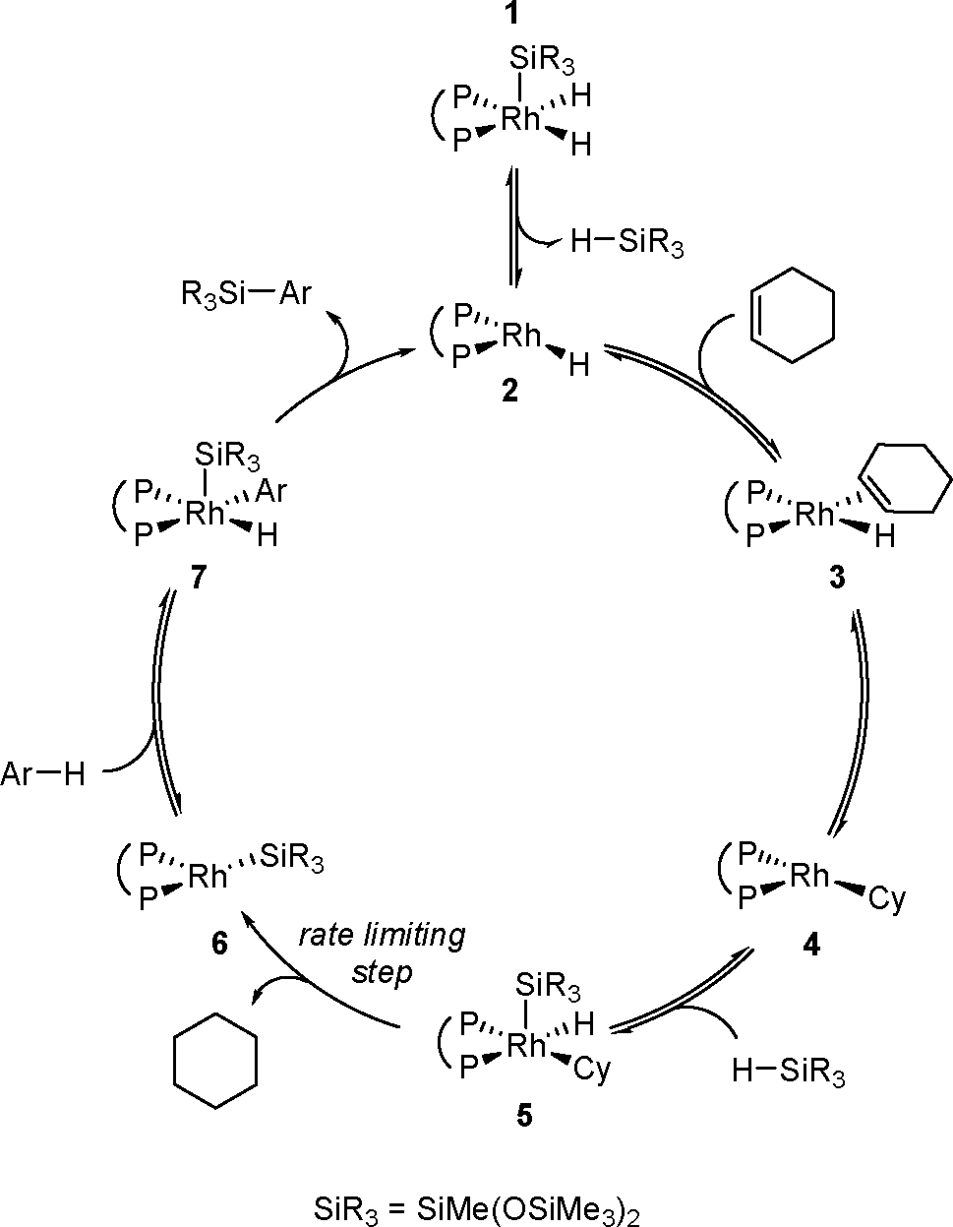
Mechanism of the Rh-Catalyzed Silylation of Aryl C–H Bonds in the Presence of HSiR_3_, with Cyclooctene as a Hydrogen Acceptor. Adapted with Permission from Ref (309). Copyright 2014 American Chemical Society

The mechanism of the process was proposed on the basis of kinetic studies, deuterium-labeling experiments, and stoichiometric reactions of isolated complexes.^309^ The proposed mechanism of the Rh-catalyzed intermolecular silylation of aryl C–H bonds shown in Scheme 121 comprises two processes: hydrogenation of the hydrogen acceptor (cyclohexene) and arene C–H silylation. The presence of the hydrogen acceptor is necessary for the reaction to proceed. The identified rate-limiting step of the mechanism is the reductive elimination of cyclohexane from an alkylrhodium(III)–hydride complex (**5**). Moreover, it has been found that the oxidative addition step is reversible. The phosphine-ligated rhodium(III)–silyldihydride complex (**1**), postulated to be the resting state of the catalyst, has been synthesized and characterized by X-ray crystallography.

**Scheme 122:**
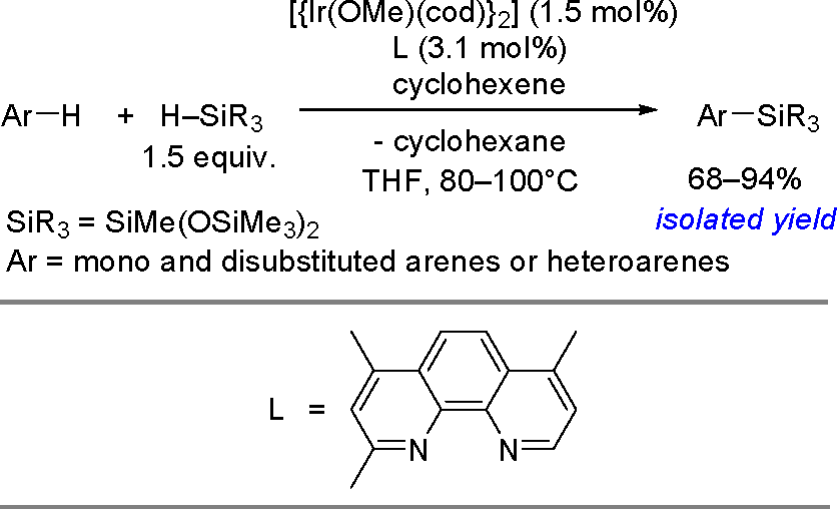
Iridium-Catalyzed Undirected Silylation of Arenes in the Presence of HSiMe(OSiMe_3_)_2_ with Cyclohexene Acting as a Hydrogen Acceptor

The [{Ir(cod)(OMe)}_2_] iridium complex, with an excess phenanthroline-based ligand enables efficient undirected silylation of arenes with HSiMe(OSiMe_3_)_2_ (Scheme 122).^325^ The ligand structure has been found to strongly influence catalytic activity. The highest yields were obtained using 2,4,7-trimethyl-1,10-phenanthroline.

**Scheme 123:**
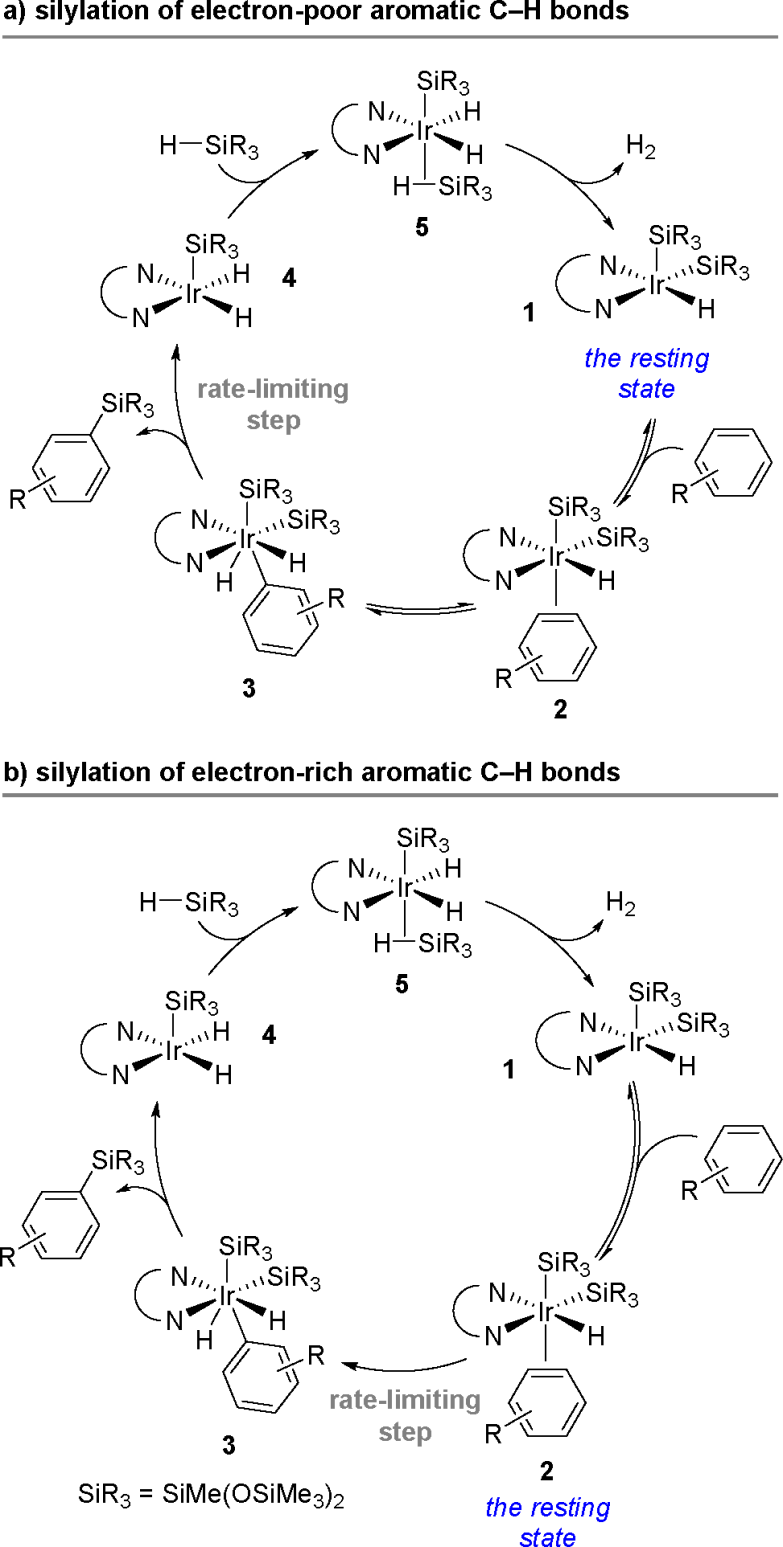
Catalytic Cycles for the Silylation of Electron-Poor (a) and Electron-Rich (b) Aromatic C–H Bonds. Adapted with Permission from Ref (327). Copyright 2020 American Chemical Society

The reaction occurs with arene as the limiting reagent and is highly regioselective. The catalyst enables efficient silylation of the C–H bond of both electron-rich and electron-poor arenes and heteroarenes. The use of 2,9-dimethylphenanthroline provides good yields (66%–94%) in the silylation of more challenging electron-rich arenes.^326^ Through a combination of experimental and computational studies, the catalytic cycle for the iridium-catalyzed silylation of aromatic C–H bonds was proposed (Scheme 123).^327^ The mechanisms involve sequences of oxidative addition of aromatic C–H bonds and reductive elimination of arylsilanes. The results indicate that the reactions involving electron-rich and electron-poor C–H bonds may be described by the same mechanistic scheme, but they differ in the rate-limiting step and the resting state of the catalyst.

**Scheme 124:**
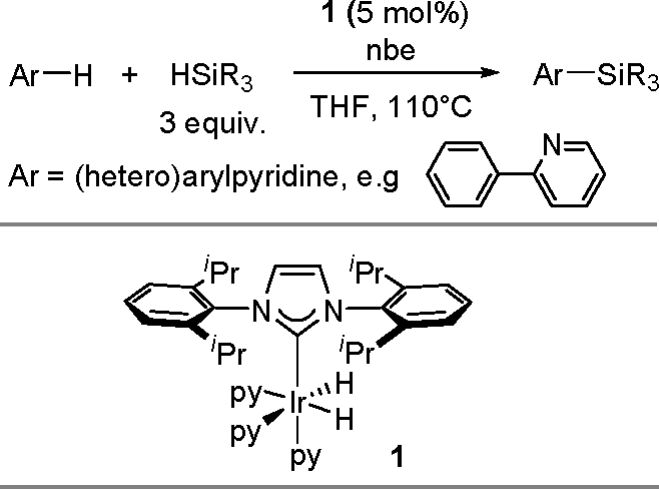
Iridium-Catalyzed Dehydrogenative Silylation of Arenes with Tertiary Silanes

Another attractive iridium-based catalytic system is [Ir(H)_2_(IPr)(py)_3_][BF_4_], containing the *N*-heterocyclic carbene ligand, described by Iglesias, Oro, and co-workers (Scheme 124).^328^ The reaction enables both directed and undirected silylation of C–H bonds with the arene as the limiting reagent. However, most of the research on the reaction mechanism concerns the silylation of arylpyridines. A variety of hydrosilanes, including Et_3_SiH, Ph_2_MeSiH, PhMe_2_SiH, Ph_3_SiH, and (EtO)_3_SiH, can be used here as reacting partners.

**Scheme 125:**
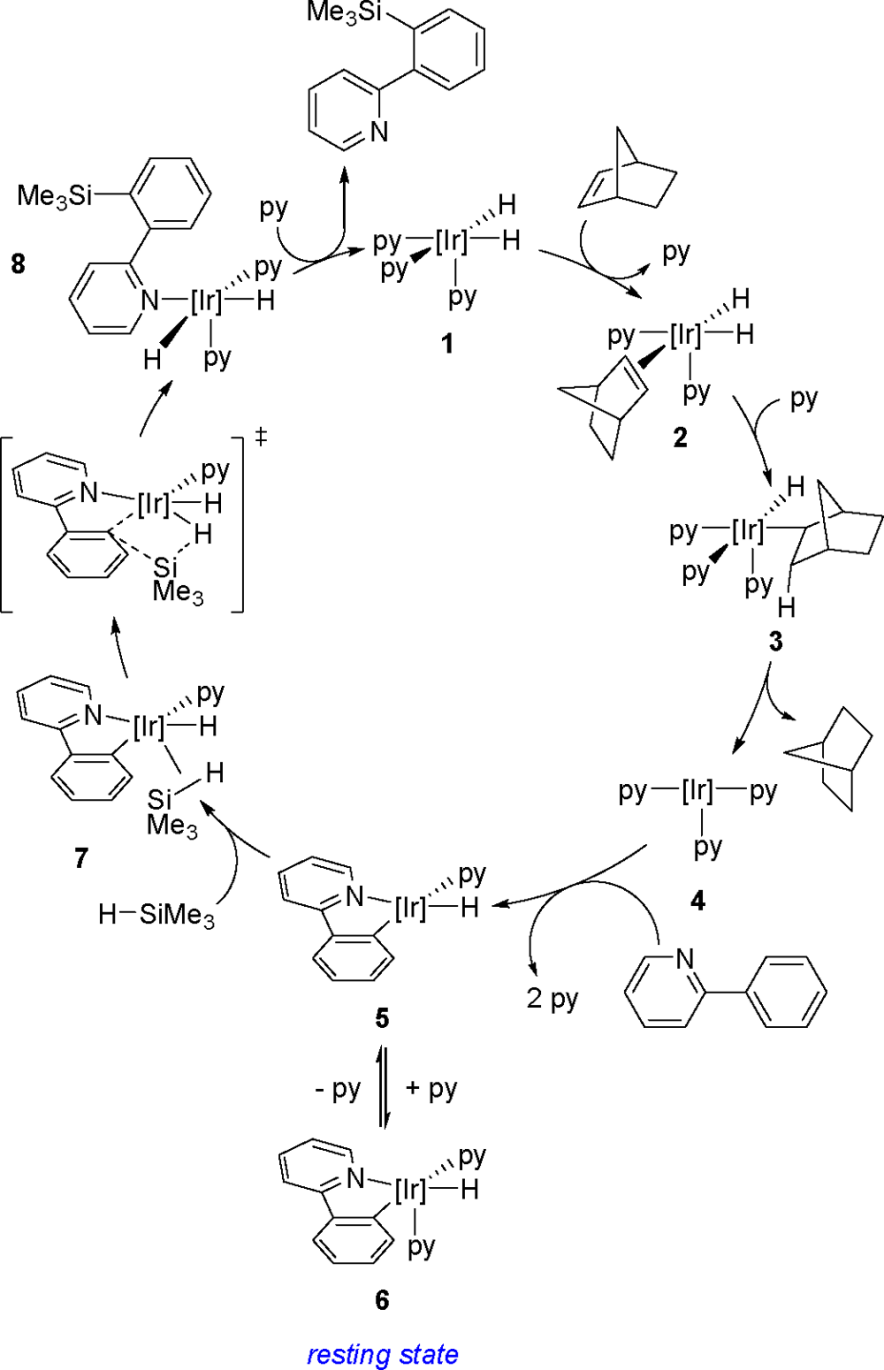
Mechanism for the Ir-Catalyzed Directed Dehydrogenative Silylation of Arene C–H Bonds with Norbornene as a Hydrogen Acceptor

DFT calculations have been conducted for a system consisting of an iridium–carbene complex (**1**) (Scheme 124), 2-phenylpyridine, HSiMe_3_ as a model hydrosilane, and norbornene as the hydrogen acceptor. Scheme 125 sums up the results of the calculations which indicate that complex **6** is the resting state of the catalyst. Transfer of the silyl group from the coordinated silane to phenylpyridine has been proposed to be the rate-limiting step, and it proceeds via σ-metathesis. The energy profile calculated for a system without a hydrogen acceptor shows a higher energy barrier.

The cyclometalated complexes corresponding to the calculated resting state of the catalyst have been synthesized, characterized, and demonstrated to be catalytically competent in arene C–H silylation.^328^

#### 3.2.4 Dehydrogenative Silylation of C(sp^3^)–H Bonds

Silylation of C(sp^3^)–H bonds was originally observed as a side reaction in the studies of other processes.^329−332^ The reaction has recently been described in a comprehensive review.^333^ As for arene silylation, the silylation of alkyl C(sp^3^)–H bonds can be divided into three classes: (i) intramolecular, (ii) directed intermolecular, and (iii) undirected intermolecular silylation of C(sp^3^)–H bonds. Complexes of Ru, Rh, Pd, Ir, and Pt have been shown to be active in the functionalization of C(sp^3^)–H bonds.

Presently, only the intramolecular mode of alkyl C–H silylation has gained importance in organic synthesis. The catalytic activity of Pt, Ir, Rh, and Ru complexes in the intramolecular silylation of C(sp^3^)–H bonds has been described. Most of the published examples concern reactions of the activated C–H bond, that is, the benzylic C(sp^3^)–H bond or the one located α to a heteroatom (N, O, Ge, Si). Moreover, in most examples, the presence of a directing group is needed. As dihydrogen is formed in the reaction, an olefin is used as a hydrogen acceptor (usually norbornene) in many reaction systems. Cyclizations can be classified as (i) reactions of an isolated silane substrate and (ii) cyclizations of an *in situ* generated silane substrate. A silyl group can be incorporated by the silylation of an O–H or N–H bond or by the hydrosilylation of an unsaturated carbon–carbon or carbon–heteroatom bond.

**Scheme 126:**
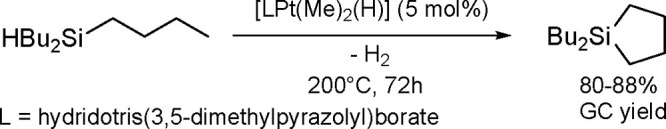
Intramolecular Cyclization of Alkylsilanes

The first example of the intramolecular silylation of a C(sp^3^)–H bond has been reported by Hartwig. In the presence of a platinum complex, tributylsilane was selectively converted into a 5-membered cyclic organosilane (Scheme 126).^306^

**Scheme 127:**
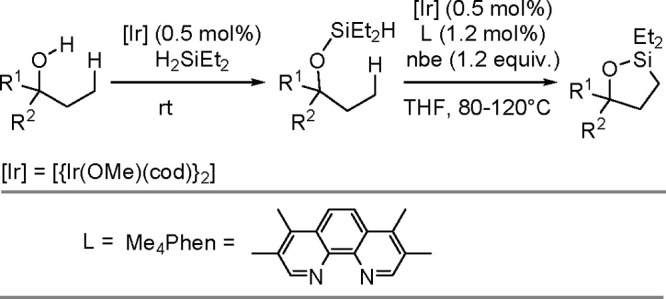
Sequential OH Silylation and Intramolecular Dehydrogenative Silylation of C(sp^3^)–H Bonds

Silylation of the inactivated primary alkyl C–H bond by the *in situ* installation of a silyl group and the subsequent selective intramolecular activation of the primary C–H bond have been proposed by the Hartwig group (Scheme 127).^334^

**Scheme 128:**
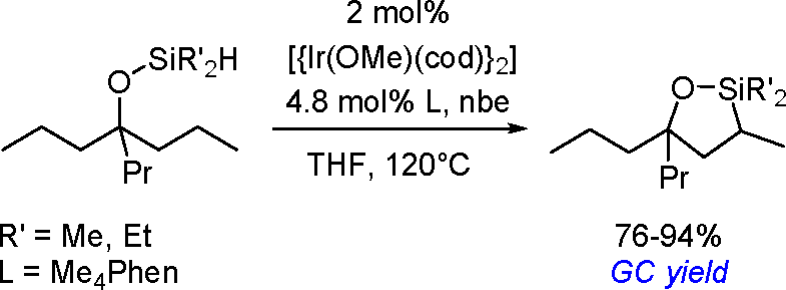
Intramolecular Dehydrogenative Silylation of Secondary C(sp^3^)–H Bonds

Five-membered cyclic silyl ethers can be obtained through the activation of secondary C–H bonds in the presence of iridium complexes (Scheme 128).^335^

**Scheme 129:**
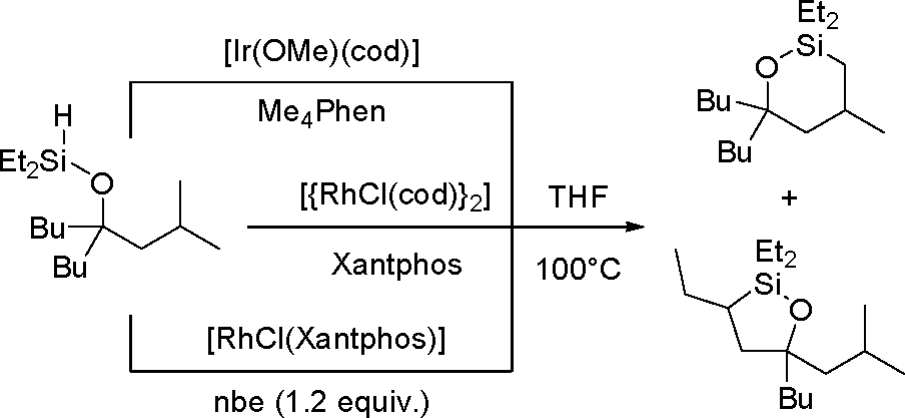
Intramolecular Silylation of C(sp^3^)–H Bonds at the γ and δ Positions

For the reagents with available C–H bonds at the γ and δ positions, a mixture of products is formed (Scheme 129).^336^

**Scheme 130:**
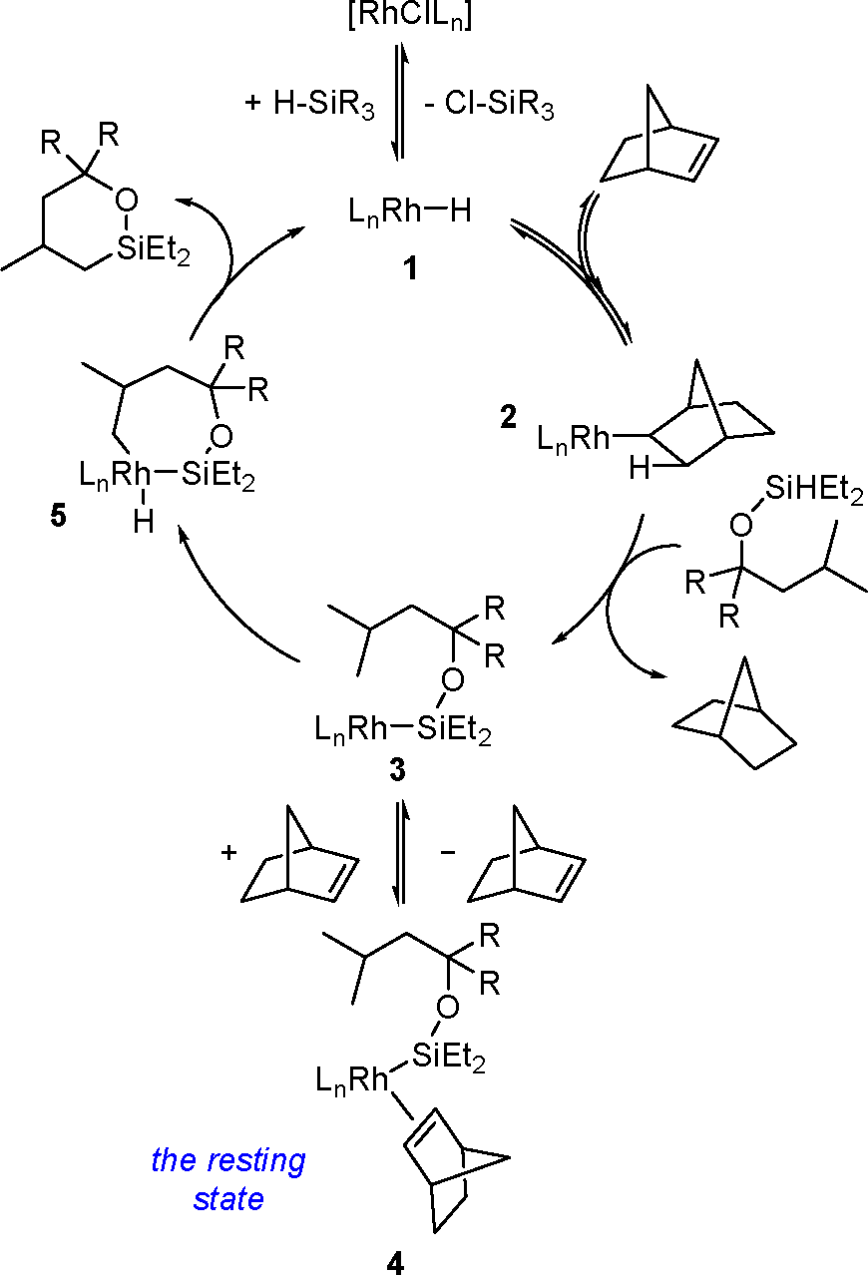
Mechanism of the Rh-Catalyzed Intramolecular Silylation of Alkyl C–H Bonds, with Norbornene as a Hydrogen Acceptor. Adapted with Permission from Ref (336). Copyright 2018 American Chemical Society

In the presence of the [Ir(OMe)(cod)] iridium complex with Me_4_Phen, a very high yield (94%) of a mixture of isomers was obtained, with significant prevalence of a five-membered product formed as a result of secondary C(γ)–H activation, which suggests that the size of the formed ring has a greater effect on selectivity than the degree of substitution at the C–H bond. The reaction is also effective in the presence of [{RhCl(cod)}_2_] used along with an appropriate diphosphine. Selectivity and yield of the reaction depend significantly on the diphosphine used; the highest yield and selectivity of the C(δ)–H silylation product were obtained for [RhCl(Xantphos)]. In contrast, [RhCl(Segphos)] afforded the same products with prevalent C(γ)–H silylation. Mechanistic studies identified [(Xantphos)Rh(SiR_3_)(nbe)] as the resting state of the catalyst. An analogue of the proposed resting state with a modified SiR_3_ group was synthesized and characterized by X-ray diffraction studies. The rate-limiting step of the process was demonstrated to be the oxidative addition of the C(δ)–H bond to Rh. The proposed mechanism of the reaction is illustrated in Scheme 130.^336^

The active catalyst has been proposed to form via the oxidative addition of a silane to [{RhCl(cod)}_2_] and the subsequent reductive elimination of chlorosilane. Rhodium(I) hydride (**1**), formed in the first step of the catalytic cycle, undergoes migratory insertion of norbornene to form **2**. Subsequent oxidative addition of a silyl hydride and reductive elimination of norbornane produces the 14-electron Rh(I) silyl complex (**3**). Association of norbornene to complex **3** produces the resting state (**4**), which arrests the catalytic cycle. Oxidative addition of the C–H bond to the rhodium center in **3** forms the metallacycle (**5**). Finally, reductive elimination of a six-membered cyclic silyl ether leads to the regeneration of the Rh(I) hydride complex (**1**). Kinetic studies show that C–H bond cleavage is irreversible and rate limiting. Computational studies have demonstrated that the selectivity for the silylation of a C(δ)–H bond over a C(γ)–H bond in the presence of the Rh-Xantphos catalyst results from a higher barrier of reductive elimination from the six-membered metalacyclic complex than from the seven-membered complex.

**Scheme 131:**
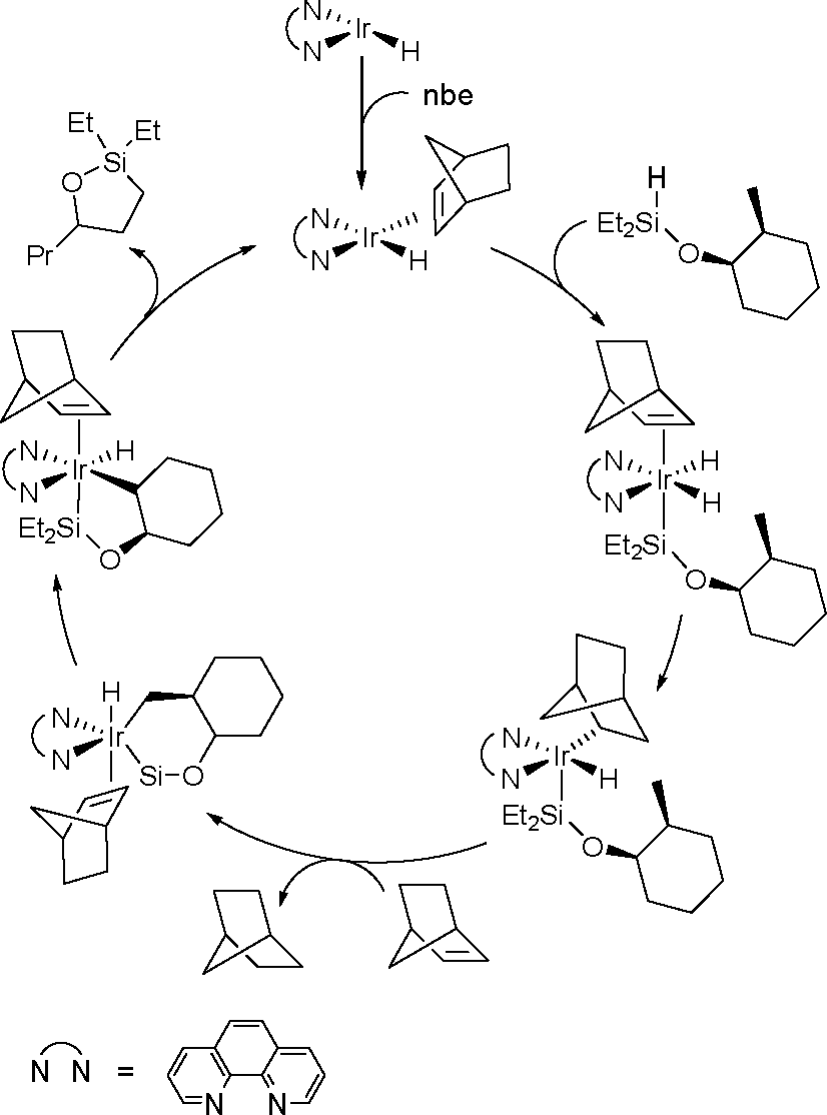
Intramolecular Silylation of Primary C(sp^3^)–H Bonds in 2-(Methyl)cyclohexanol Proposed by Sunoj^337^

Sunoj examined the Ir-catalyzed γ-functionalization of a primary C(sp^3^)–H bond in 2-methyl cyclohexanol using DFT calculations.^337^ The [IrH(nbe)(phen)] complex was identified as the active catalyst. The proposed mechanism assumed a catalytic cycle with activities of Ir(I) and Ir(III) complexes (Scheme 131).

**Scheme 132:**
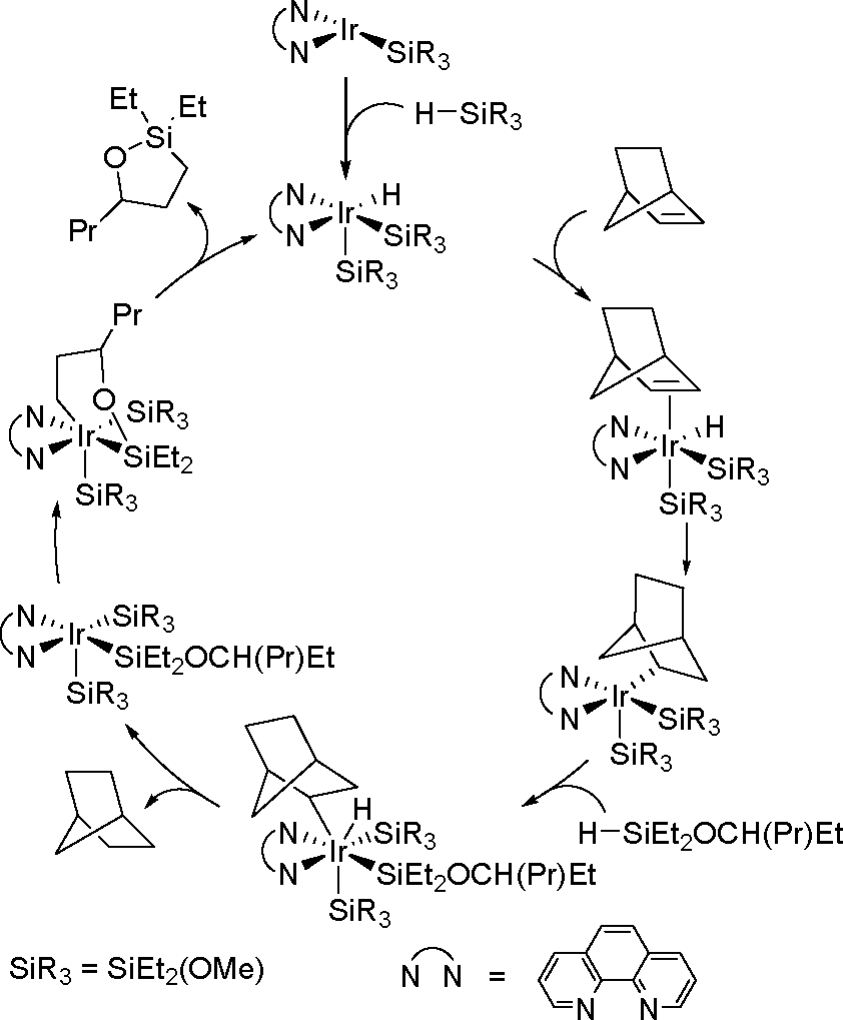
Intramolecular Silylation of Primary C(sp^3^)–H Bonds in 2-(Methyl)cyclohexanol Proposed by Li^338^

In contrast, the calculations performed by Li^338^ for the reaction presented in Scheme 132 indicate that the Ir(III)/Ir(V) mechanism is more feasible than the one proposed by Sunoj.^337^

Formation of [Ir(Me_4_phen){Si(OR)Et_2_}] as the catalyst precursor was postulated to be more probable than the formation of [IrH(Me_4_phen)(nbe)] as proposed by Sunoj. The intramolecular oxidative addition of the C(sp^3^)–H bond was proposed to occur in the [(Me_4_Phen)Ir{SiEt_2_(OR)}_3_] iridium(III) complex. This step was calculated to be rate limiting.

**Scheme 133:**
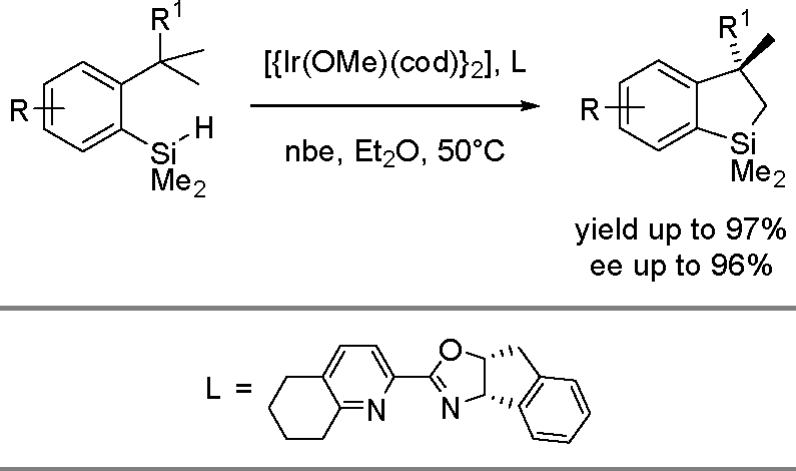
Asymmetric Intramolecular Silylation of Inactivated C(sp^3^)–H Bonds

The first example of the asymmetric intramolecular silylation of inactivated C(sp^3^)–H bonds was reported by Murai, Takai, and co-workers.^339^ Hartwig developed a highly enantioselective intramolecular silylation of inactivated C(sp^3^)–H bonds under relatively mild reaction conditions (Scheme 133).^340^ It was found that in the presence of a combination of [{Ir(OMe)(cod)}_2_], a chiral pyridyl oxazoline ligand (L) and norbornene (nbe) as the hydrogen acceptor, dimethylarylsilanes (**1**) underwent selective silylation at one of the two prochiral methyl groups to afford **2** in high yields and with excellent enantioselectivity.

**Scheme 134:**
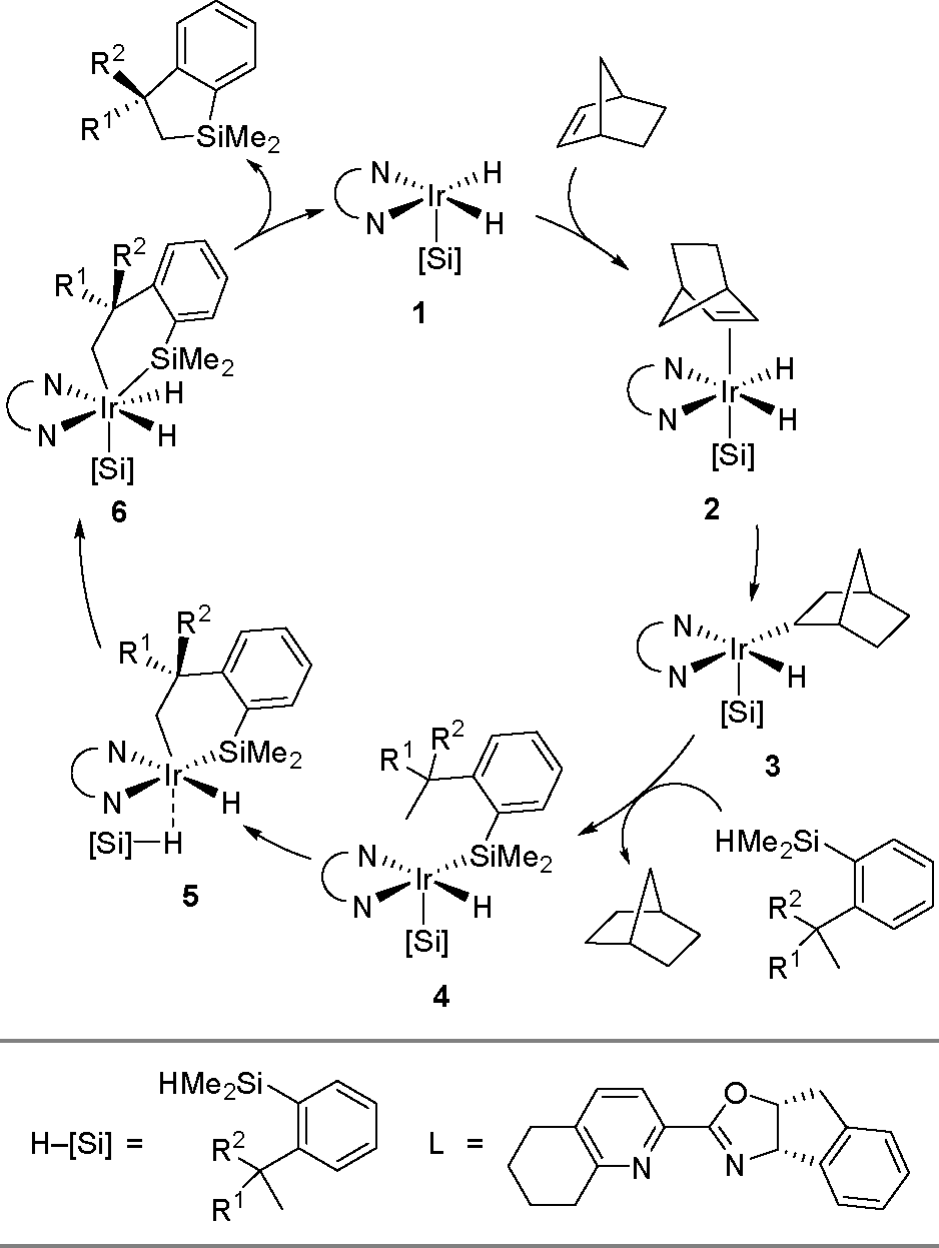
Mechanism of the Ir-Catalyzed Intramolecular Silylation of Alkyl C–H Bonds, with Norbornene as the Hydrogen Acceptor^341^

Kinetic studies suggest that C–H bond cleavage is the turnover-limiting step of the catalytic cycle.^335^ In 2019, Huang and co-workers presented results of density functional theory calculations for the mechanism of the iridium(I)-catalyzed intramolecular silylation of inactivated C(sp^3^)–H bonds, and a reasonable reaction mechanism was proposed with the origin of enantioselectivity explained (Scheme 133).^341^ Dimethylarylsilane was selected as the model substrate in these calculations. Formation of the [Ir(OMe)L(nbe)] complex from [{Ir(OMe)(cod)}_2_], L and norbornene as the hydrogen acceptor was calculated to be the most exergonic. The subsequent reactions of the complex with two silane molecules produced the [IrLH_2_Si] iridium(III) complex (**1**), proposed to be the active catalyst of the reaction. According to the mechanism (Scheme 134), the coordination of nbe to complex **1** produces intermediate **2**. Subsequently, **2** undergoes migratory insertion of a C=C double bond of norbornene into the Ir–H bond. The resultant intermediate (**3**) has been found to interact with silane to give an iridium(III) disilyl hydride species (**4**) and release norbornane. Further steps of the mechanism occur through the Ir(III)/Ir(V) catalytic cycle and involve the oxidative addition of the C(sp^3^)–H bond to the metal center and subsequent reductive elimination. Oxidative addition may lead to the formation of two enantiomeric complexes, S-**5** or R-**5**, and consequently to an enantiomeric product. The calculated energy profile shows that C(sp^3^)–H oxidative addition constitutes the rate-limiting and enantioselectivity-determining step.

On the basis of the optimized geometric structures of the respective transition states, the origin of enantioselectivity was suggested as a combination of steric and electronic effects.

**Scheme 135:**
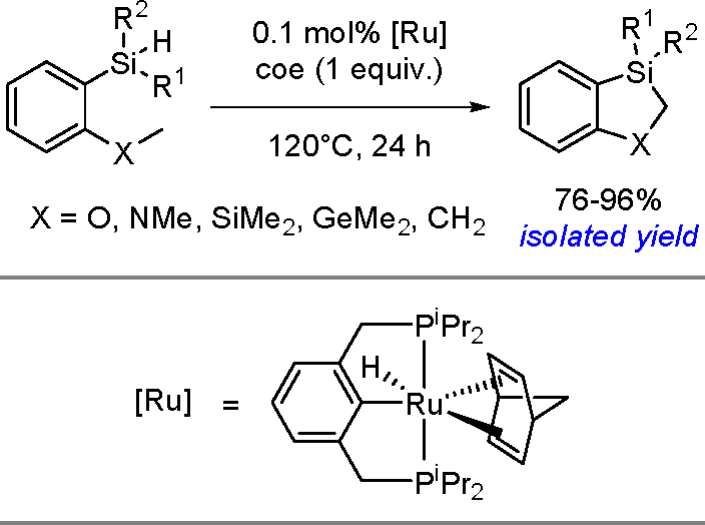
Intramolecular Silylation of Primary C(sp^3^)–H Bonds Catalyzed by a Ruthenium–Pincer Complex

Finally, Huang reported that a ruthenium–pincer complex was capable of the silylation of activated and inactivated primary C(sp^3^)–H bonds to form sila(hetero)cyclic products (Scheme 135).^342^

**Scheme 136:**
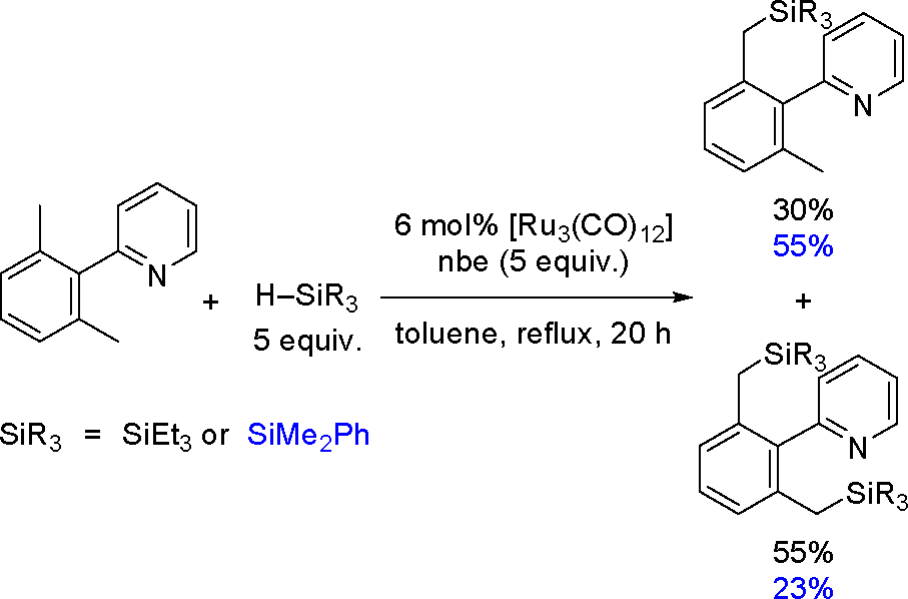
Directed Intermolecular Silylation with Tertiary Silanes

Directed intermolecular silylation of alkyl C–H bonds has been demonstrated for substrates with activated C–H bonds, such as those located in the benzylic position or α to a heteroatom (e.g., N, O or Ge). Only a few examples of the directed silylation of inactivated C–H bonds are known. The first intermolecular silylation of benzylic C–H bonds has been reported by Kakiuchi and co-workers (Scheme 136)^343^ who described several examples using a pyridyl or a pyrazolyl directing group.

**Scheme 137:**
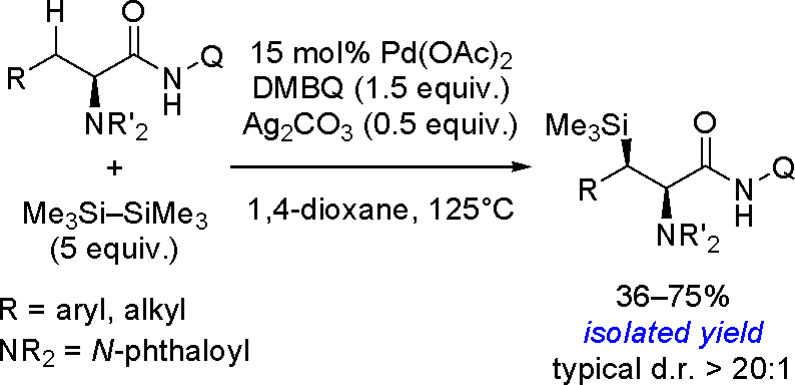
Directed Intramolecular Silylation of Secondary C(sp^3^)-H Bonds with Disilane

Mita and Sato have reported analogous silylation to occur in the presence of the iridium complex [{Ir(cod)Cl}_2_] and pyrimidine, pyrazine, and oxazole directing groups. The Ir-catalyzed reaction does not require a hydrogen acceptor to proceed efficiently.^344^ Stereoselective silylation of inactivated primary or secondary C(sp^3^)–H bonds of α-amino acids in the β position to oxygen has been reported by Shi.^345^ The reaction runs in the presence of palladium complexes and uses hexamethyldisilane as the silylating agent (Scheme 137). A directing-group-assisted approach was used, as proposed for carboxylic acid derivatives by Kuninobu and Kanai.^316^

**Scheme 138:**
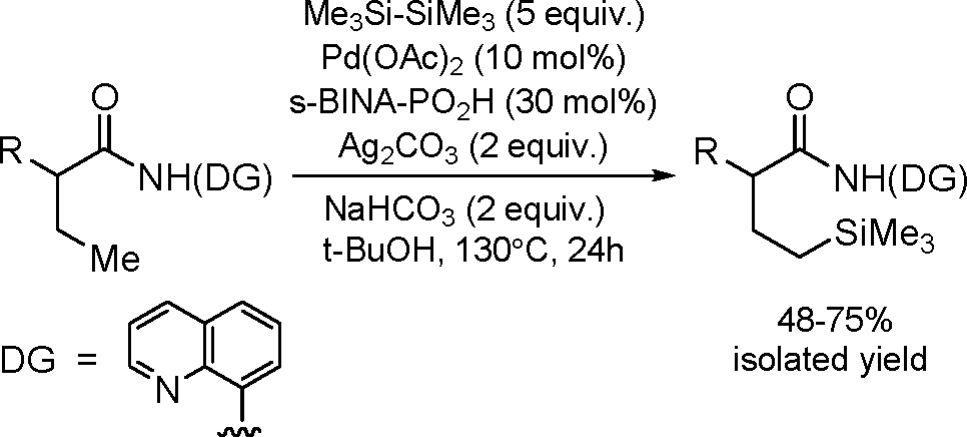
Directed Intramolecular Silylation with Disilanes

The yields were 31%–71% and 36%–75% in the silylation of primary and secondary C–H bonds, respectively. When the mechanism of this process was investigated, palladacyclic intermediates were isolated, shown to form silylation products in a stoichiometric reaction with disilane and being catalytically active intermediates in primary and secondary C(sp^3^)–H silylation. The palladium-catalyzed silylation of γ C(sp^3^)–H bonds in a series of aliphatic carboxamides with hexamethyldisilane has been described by Sunoj and Maiti (Scheme 138).^346^

**Scheme 139:**
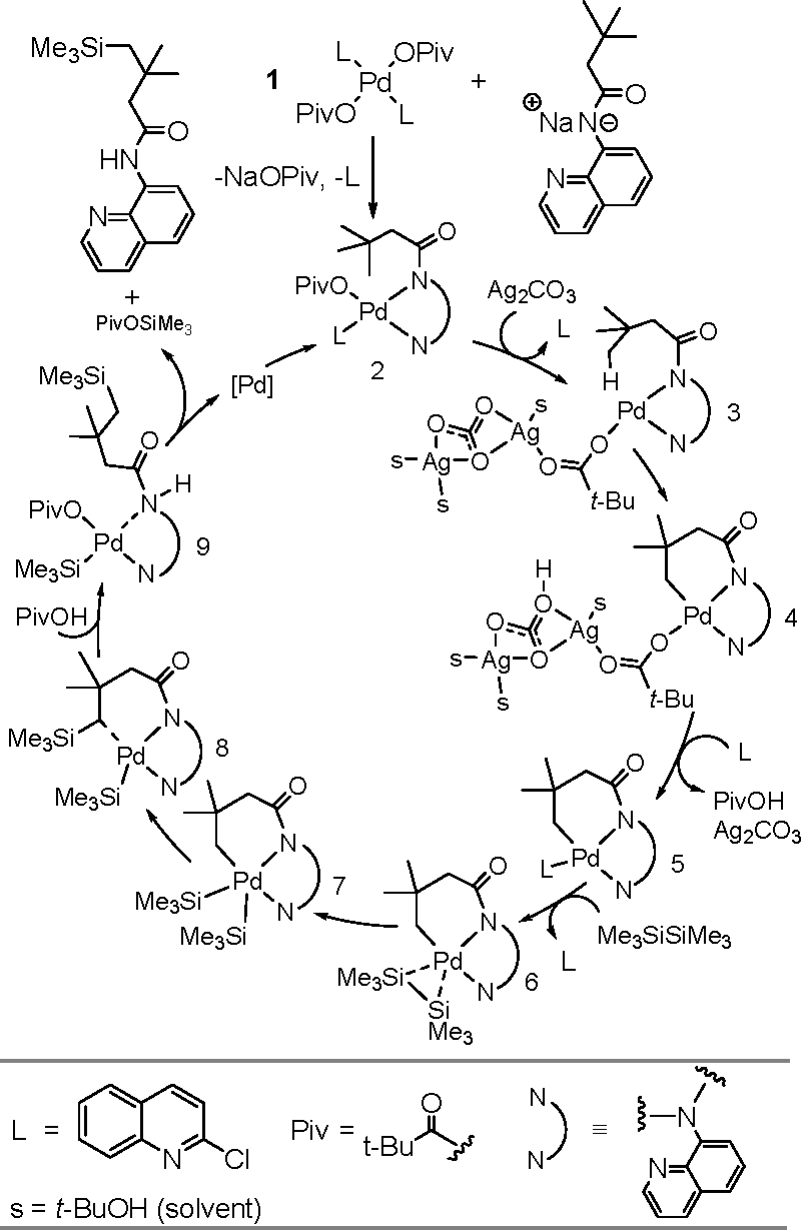
Mechanism of the Pd-Catalyzed γ-Silylation (or Germylation) of Alkyl C–H Bonds. Adapted with Permission from Ref (346). Copyright 2017 American Chemical Society

A reaction mechanism has been proposed on the basis of preliminary kinetic studies and DFT calculations (Scheme 139). [Pd(OPiv)_2_L_2_] (L = 2-chloroquinoline) (**1**) has been found to be energetically the most favorable active species. Association of the substrate as a sodium amidate to complex (**1**) forms a catalyst–substrate chelate complex (**2**), proposed to be the starting point of the catalytic cycle. Activation of the C–H bond occurs via concerted metalation-deprotonation to form palladacycle (**4**). Calculations show that the carbonate ligand is involved in C–H activation. Subsequently, 2-chloroquinoline replaces the labile pivalic acid and silver carbonate (formed by combining silver pivalate and silver bicarbonate) to give **5**. A model of **5** has been successfully synthesized and characterized by X-ray diffraction studies. In the next step, disilane displaces ligand L to form intermediate **6**, which subsequently undergoes oxidative addition to form a palladium(IV) complex (**7**). This step was calculated to be rate-limiting. The subsequent stage is the formation of a C–Si bond as a result of reductive elimination. The PivOH formed in the C–H activation step then interacts with intermediate **8** by proton transfer to the amidate nitrogen to form a product–catalyst complex (**9**). Subsequently, product dissociation, reductive elimination of silyl pivalate, and oxidation of the formed palladium(0) complex occur. Silver carbonate is used as an oxidant. The C(sp^3^)–H bond activation step is proposed to be reversible.

DFT calculations were also performed for the analogous process of germylation.^346^ The potential energy profiles for the silylation and germylation reactions are slightly different. For both processes, the highest-energy points are the transition states for the oxidative addition of Si–Si (or Ge–Ge) bonds to the palladium(II) complex and for reductive elimination. Analysis of the energy profiles shows that the oxidative addition of disilane is the rate-limiting step of silylation, whereas reductive elimination is the rate-limiting step of germylation.

**Scheme 140:**
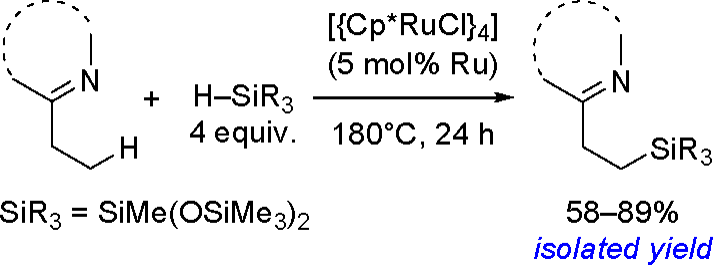
Directed Intramolecular Silylation with HSiMe(OSiMe_3_)_2_

The silylation of the C(sp^3^)–H bond in 2-alkyloxazolines and 2-alkylpyridines occurs in the presence of the [{Cp*RuCl}_4_] ruthenium tetramer (Scheme 140).^347^ The reaction is site-selective and proceeds at the inactivated primary C–H bond in the γ-position to nitrogen. The reaction is effective only in the presence of HSiMe(OSiMe_3_)_2_. It requires high temperature to run efficiently and is accompanied by efficient silane dehydrocoupling.

Labeling studies and DFT calculations show that the reductive elimination of the C–Si bond limits the reaction rate.

**Scheme 141:**
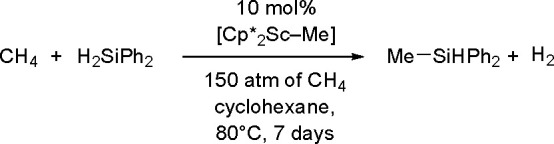
Dehydrogenative Silylation of Methane

Undirected silylation of the C(sp^3^)–H bond is the least developed silylation of C–H bonds. In 2003, Tilley reported the intermolecular silylation of methane in the presence of the [Cp*_2_ScMe] scandium complex.^348,349^ On the basis of DFT calculations, the reaction was proposed to involve Si–Me bond formation via σ-bond metathesis between the Sc–Me and Si–H bonds (Scheme 141).

The reaction has not gained practical importance. However, reports on the reaction opened up new opportunities for catalysis as the productive functionalization of methane by σ-bond metathesis had not been described.

**Scheme 142:**
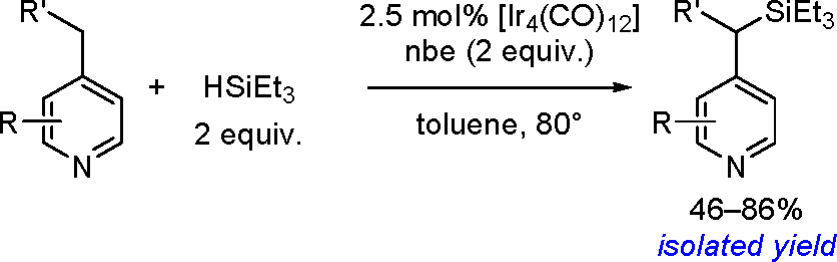
Undirected Silylation at the Benzylic Position of 4-Alkylpyridines

Fukumoto has revealed the silylation of C(sp^3^)–H bonds at the benzylic position in 4-alkylpyridines^350^ and 2-alkylpyridines^351^ with hydrosilanes (Scheme 142).

**Scheme 143:**
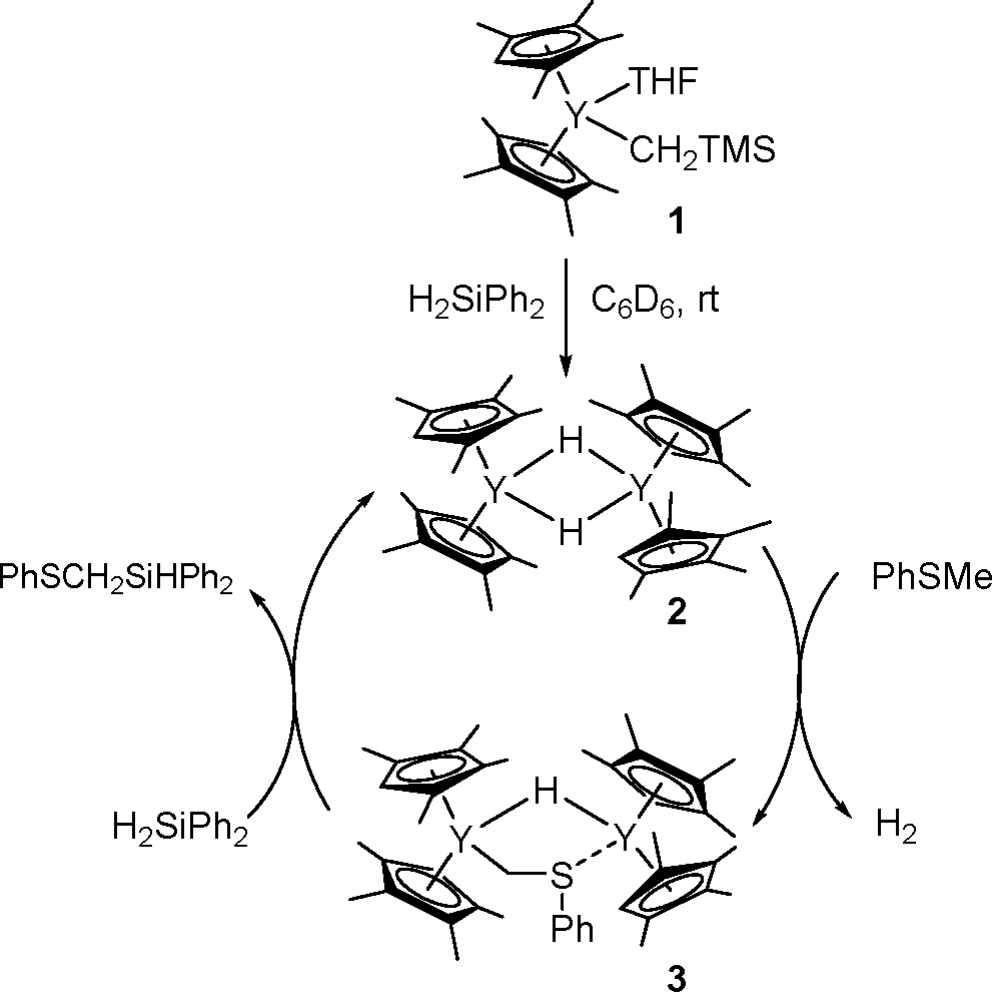
Mechanism of the Y-Catalyzed Intramolecular Silylation of Alkyl C–H Bonds. Adapted with Permission from Ref (352). Copyright 2018 American Chemical Society

The reaction takes place in the presence of [Ir_4_(CO)_12_]. The yield is strongly improved when 3,5-dimethylpyridine is added. The proposed mechanism includes an electrophilic silicon species as the key intermediate. Product formation is proposed to occur by the silylation of a *N*-silylated enamine with electrophilic silicon species and subsequent elimination of the silyl group. However, several details of the mechanism remain unclear. Finally, Hou and co-workers reported the regioselective α-C–H silylation of methyl sulfides with hydrosilanes catalyzed by yttrium complexes.^352^ The catalytic cycle has been proposed on the basis of stoichiometric reactions and isolation of key intermediates (Scheme 143).

### 3.3 Dehydrogenative Germylation of C–H Bonds

Direct germylation of organic molecules is still at the early stage of development. The interest in the germylation of arene C(sp^2^)–H and C(sp^3^)–H bonds has grown in the past decade.

#### 3.3.1 Dehydrogenative Germylation of Arene C(sp^2^)–H Bonds

**Scheme 144:**
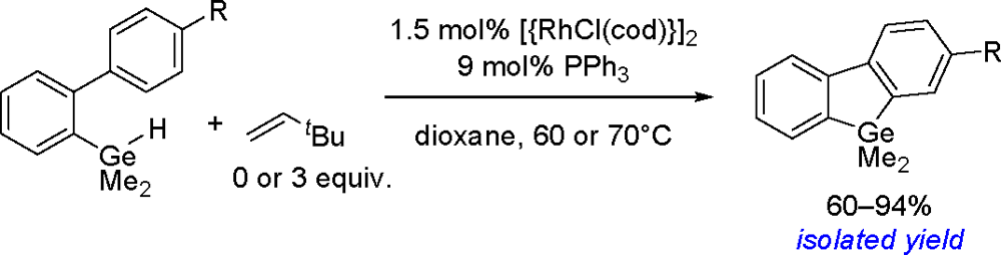
Intramolecular Dehydrogenative Germylation of Arene C–H Bond

Murai and Takai have reported rhodium-catalyzed intramolecular germylation leading to 9-germafluorene and germylene-bridged π-conjugated systems (Scheme 144).^308,353^

**Scheme 145:**
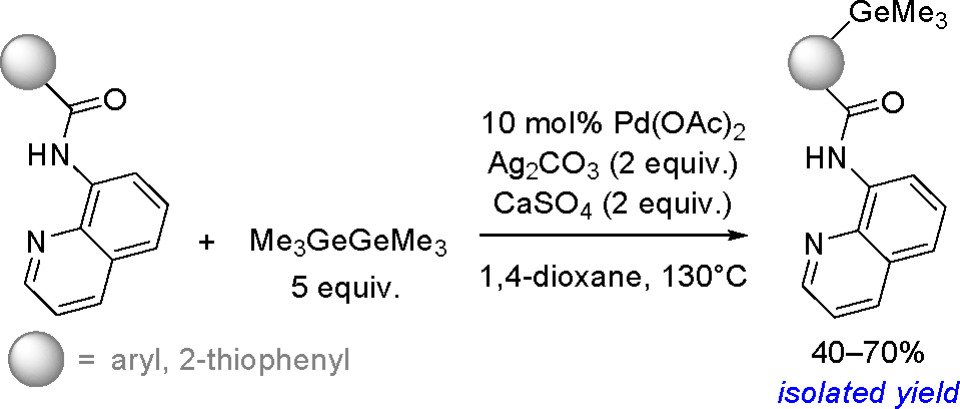
Directed Germylation of Arenes with Digermanes

Directed germylation of arenes has been reported by Kuninobu and Kanai. Benzamides and carboxamides undergo germylation with Me_3_GeGeMe_3_ in the presence of a palladium catalyst (Scheme 145).^316^

**Scheme 146:**
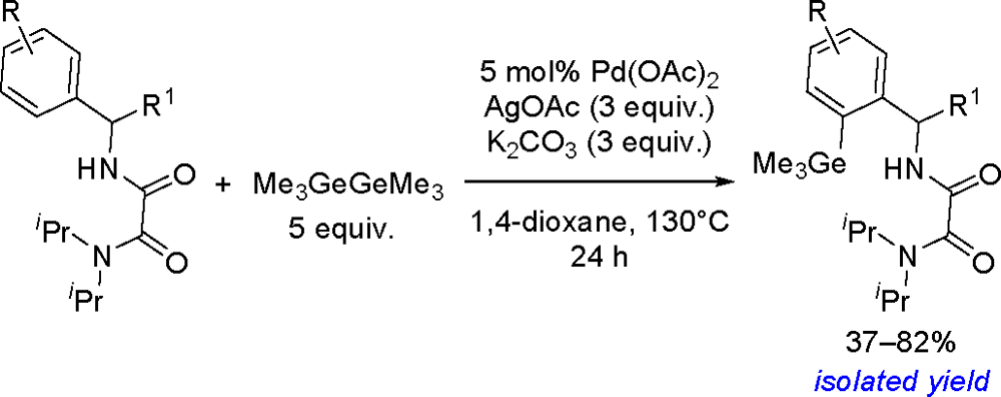
Directed Germylation of Arenes with Digermanes

An analogous palladium-catalyzed directed germylation of modified benzylamines has been reported by Wen and Zhao (Scheme 146).^354^

Finally, Maiti and co-workers demonstrated the directed *meta*-selective germylation of the arene C–H bond.^355^ The procedure of arene germylation with HGeEt_3_ in the presence of a palladium phosphine dimer via formation and functionalization of a tetrafluorothianthrenium salt has been reported by Schoenebeck.^356^ The reaction proceeds in mild conditions and enables germylation of nonactivated and directing group-free arenes. The transformation tolerates a variety of functional groups (i.e., phenoxy, carbonyl, amide, nitro, nitrile, and chloride).

#### 3.3.2 Dehydrogenative Germylation of C(sp^3^)–H Bonds

**Scheme 147:**
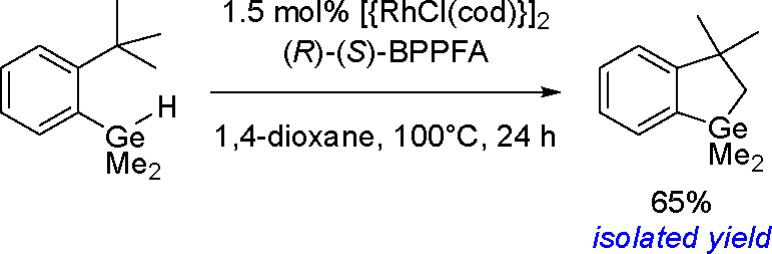
Rhodium-Catalyzed Intramolecular Germylation

Murai and Takai have described the rhodium-catalyzed intramolecular germylation of the primary C(sp^3^)–H bond leading to the synthesis of 2,3-dihydrobenzo[*b*]germole (Scheme 147).^339^

**Scheme 148:**
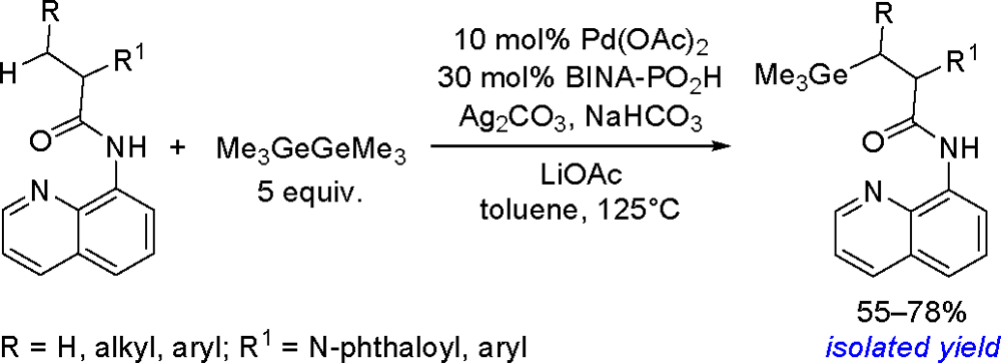
Directed Germylation of Primary and Secondary Alkyl C(sp^3^)–H Bonds

Palladium-catalyzed germylation of primary and secondary alkyl C(sp^3^)–H bonds enables the synthesis of β-germyl-α-amino amides and β-germyl-modified α-amino acids after deprotection of the amine group (Scheme 148).^357^

**Scheme 149:**
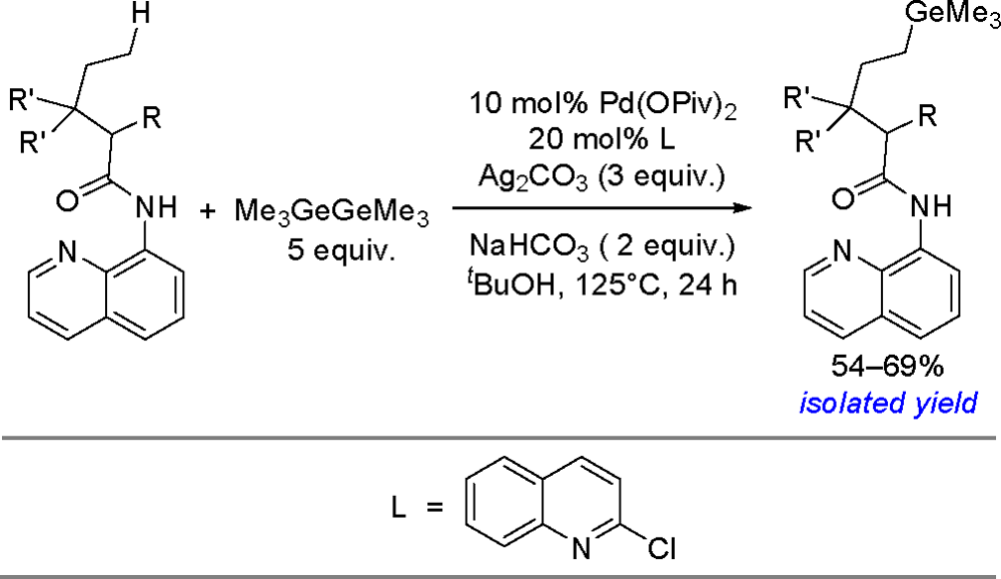
Directed Dehydrogenative Germylation of γ-C(sp^3^)–H Bonds

Directed γ-C(sp^3^)–H germylation of aliphatic carboxamides has been successfully performed by Maiti (Scheme 149).^346^

## 4 Activation (and Functionalization) of C–X Bonds with Hydrometalloids and Bismetalloids

This section covers the activation of the C–X bond, where X is a halide or sulfonate, and its functionalization leading to the formation of the carbon–metalloid bond. The activation and functionalization of C–C, C–O, C–N, and C–S bonds in cross-coupling processes is beyond the scope of this Review.

### 4.1 Borylation of C–X Bonds

The reaction offers an alternative to the classical method of C–B bond synthesis, including the treatment of trialkyl borates with magnesium or lithium alkyl reagents, followed by hydrolysis or transesterification. Unlike the catalytic C–H borylation reaction, this reaction does not suffer from a lack of site selectivity. This section also discusses the activation and functionalization of the C–F bond.

#### 4.1.1 Borylation of Olefinic C(sp^2^)–X Bonds

**Scheme 150:**
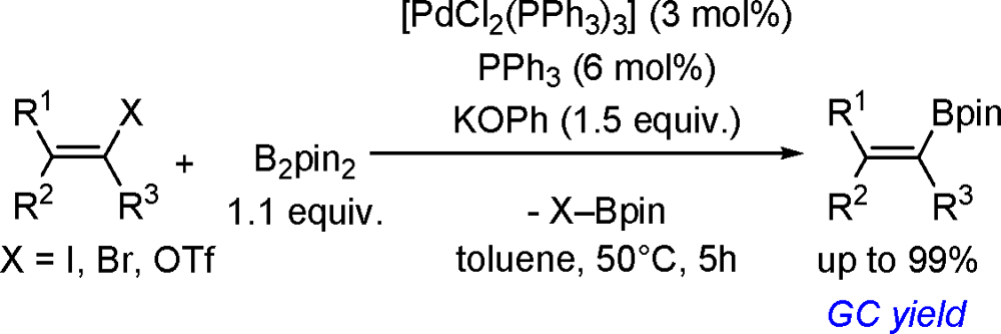
Borylation of Olefins

Miyaura has shown that 1-alkenyl iodides, bromides, and triflates can be borylated with B_2_pin_2_ in the presence of a palladium complex and potassium phenoxide (Scheme 150).^358,359^ The reaction is highly efficient and proceeds with complete retention of the double bond configurations.

**Scheme 151:**
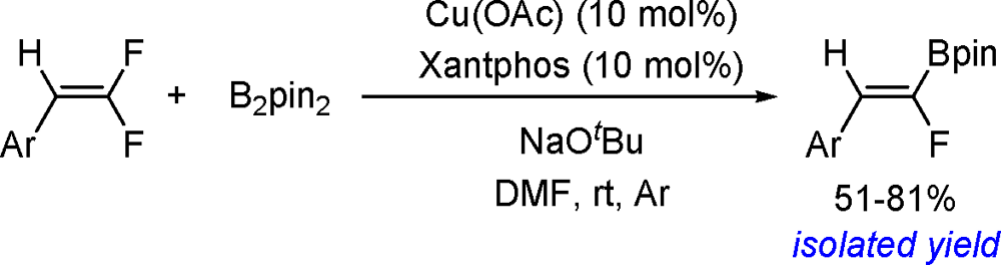
Borylation of Fluoroolefins

Buchwald has reported that vinyl chloride can be successfully converted into the corresponding pinacol boronate ester by borylation with HBpin in the presence of [PdCl_2_(CH_3_CN)_2_] and SPhos.^360^ The first example of olefin defluoroborylation has been described by Braun.^361^ The [RhH(PEt_3_)_3_] rhodium complex was shown to catalyze the C–F bond borylation of hexafluoropropene with HBpin at room temperature in quantitative yields. The sequence of catalytic transformations involves consecutive hydroboration, hydrogenation, and/or dehydrogenative borylation. Cao and co-workers reported the Cu-catalyzed stereoselective monoborylation of gem-difluoroalkenes with B_2_pin_2_ (Scheme 151).^362^ In the presence of CuOAc, NaO*t*-Bu, and Xantphos, a variety of (Z)-fluorinated alkenylboronic acid pinacol esters were obtained with moderate to good yields at room temperature. Involvement of the phosphine-coordinated copper-boryl complex in the reaction mechanism was proposed.

**Scheme 152:**
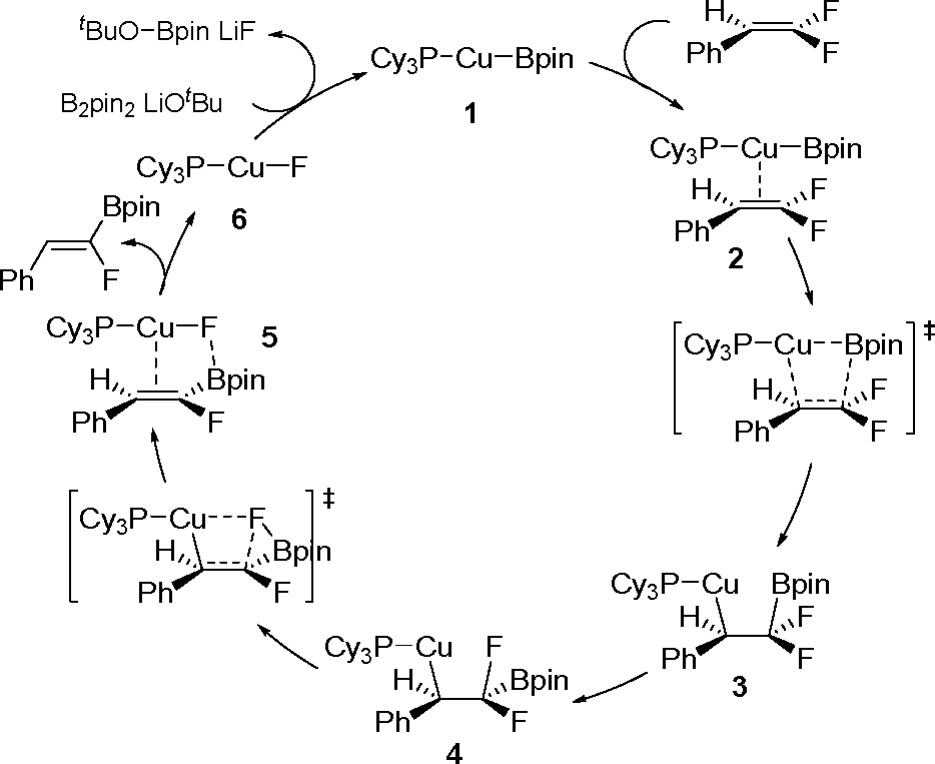
Mechanism of the Cu-Catalyzed Defluorinative Borylation of gem-Difluoroalkenes

[CuCl(PCy_3_)_2_], another copper complex, catalyzes regioselective monodefluoroborylation of polyfluoroalkenes.^363^ The reaction takes place under mild conditions in the presence of a base. The choice of the boron source has been found to be important for the efficient transformation of gem-(difluorovinyl)arenes. While B_2_pin_2_ was suitable for substrates with an electron-deficient aryl group, B_2_nep_2_ performed better with reagents bearing an electron-rich aryl group. By examining an analogous stereoselective borylation of gem-difluoroalkenes, Ito^364^ and Gao and Wang^365^ independently proposed a reaction mechanism based on DFT calculations (Scheme 152). The identified active catalyst of the process is the [Cu(Bpin)(PCy_3_)] complex (**1**). The olefin associates to the complex and undergoes regioselective insertion into the Cu–B bond, resulting in the formation of complex **3** in which boron is bound to the gem-difluoromethylene unit. Subsequently, after rotation of the C–C bond, syn-planar β-F elimination occurs, and **5** is formed. The interaction of the fluorine atom with the empty p orbital of boron contributes to the weakening of the C–F bond. Reductive elimination of vinylboronate and subsequent transmetalation result in the regeneration of complex **1**. The possibility of inserting olefins into the Cu–B bond was previously demonstrated experimentally by Sadighi^366^ and as a result of DFT calculations by Lin and Marder groups.^367^

#### 4.1.2 Borylation of Aryl C(sp^2^)–X Bonds

**Scheme 153:**
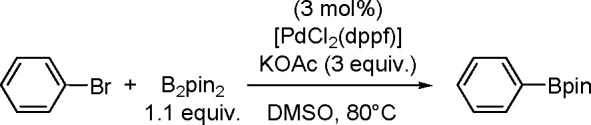
Borylation of Bromoarenes with B_2_pin_2_

The first borylation of aryl halides was reported in 1995 by the Miyaura group (Scheme 153).^368^ Cross-coupling of aryl iodides or bromides with a pinacol ester of diboronic acid (B_2_pin_2_) in the presence of [PdCl_2_(dppf)] and KOAc as a base gave bromoarenes with high selectivities and yields. The use of KOAc is crucial for the selective synthesis of the arylboronic acid pinacol ester, as competing Suzuki–Miyaura cross-coupling may occur in the presence of stronger bases. The process is known as Miyaura borylation.

Borylation of chloroarenes has been found to proceed in the presence of [Pd(dba)_2_]/PCy_3_ and KOAc (1.5 equiv). This catalyst also permitted the borylation of aryl bromides and triflates and tolerated the presence of ester, ketone, and nitrile functionalities.^369^ Procedures for the effective borylation of (hetero)aryl halides (electron-rich or -poor) and procedures for the borylation of sterically crowded aryl halides have been described.^370−372^ This important reaction feature has been achieved using bulky ligands. Besides, the activation and functionalization of C–X bonds (X = I, Br, Cl, OTf, OTs, OMs) is possible. Another important property of the reaction is functional group compatibility. The reaction can run effectively with a broad spectrum of functional groups. The metals Mn, Fe, Co, Ni, Cu, and Zn are known to show catalytic activity in Miyaura borylation. Examples of photoinduced and photocatalyzed reactions have been reported.^373−375^ Advances in the TM-catalyzed borylation of aryl halides have been reviewed (e.g., refs (100, 376, and 377)).

**Scheme 154:**
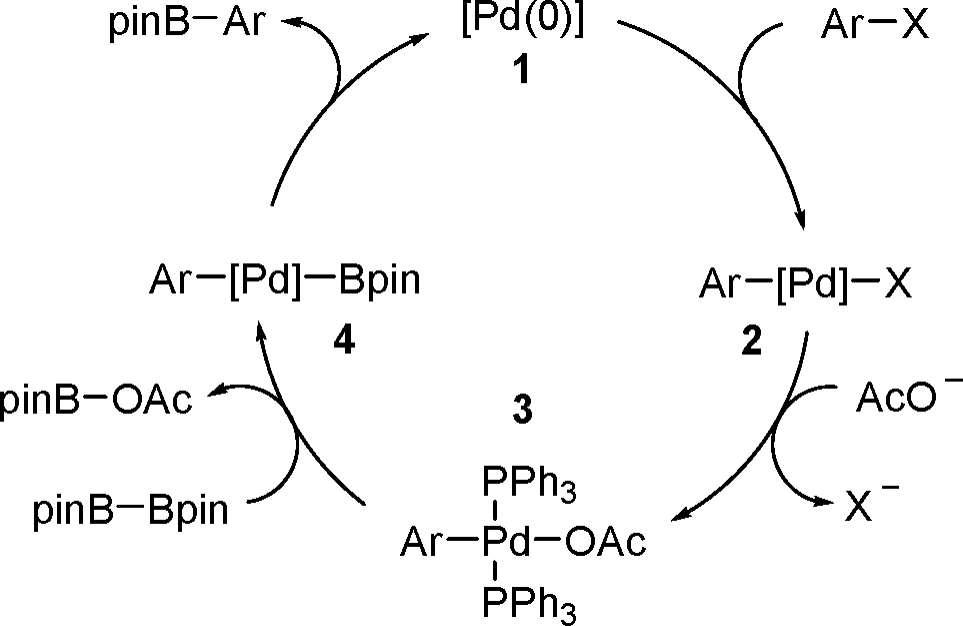
Mechanism of Aryl Halide Borylation with B_2_pin_2_ Proposed by the Miyaura Group^357^

The reaction mechanism has been proposed in Miyaura’s seminal paper^368^ on the basis of the isolation and characterization of the *trans*-[Pd(OAc)Ph(PPh_3_)_2_] intermediate and a study of its stoichiometric reactivity with bis(pinacolato)diboron (Scheme 154).

The transmetalation step in a simplified reaction model has been investigated by DFT.^378^ The results showed the importance of the type of base used for the energy profile of the process. When a hydroxide or a fluoride is used as an anionic ligand in the palladium complex, the formation of strong X–B bonds weakens the Pd–X and B–B bonds and accelerates both the transmetalation and the cleavage of B–B bonds. According to DFT calculations, corroborated by experimental results, the energy barrier for the transmetalation of palladium chloride is high. Moreover, the calculations imply that the associative mechanism of transmetalation takes place more easily than the dissociative mechanism, while the reductive elimination of an arylboronic ester from the bisphosphinic complex occurs with no significant energy barrier.

**Scheme 155:**
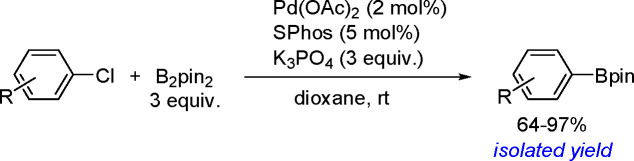
Borylation of Aryl Chlorides with B_2_pin_2_

A recently reported convenient reaction procedure involves the use of lipophilic bases such as potassium 2-(ethyl)hexanoate; the reaction proceeds under low palladium catalyst loading (0.5 mol %) in mild conditions.^379^ A preliminary mechanistic study showed an inhibitory effect exerted by the carboxylate anion on the catalytic cycle.^379^ On the basis of the results, a modification of the reaction mechanism was proposed to include the formation of an inactive diacetate anionic complex. Moreover, a dissociative mechanism of phosphine substitution by a borylating agent prior to transmetalation was suggested. Buchwald has shown that catalysts composed of Pd and bulky biarylmonophosphine such as XPhos or SPhos are highly stable and active for the borylation of aryl chlorides (Scheme 155).^370^

**Scheme 156:**
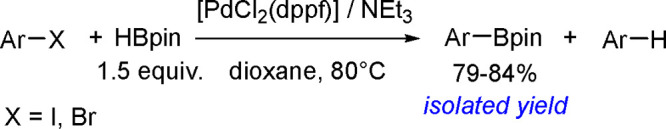
Arene Borylation with Pinacolborane

In 1997, the borylation of aryl halides using HBpin was described by Masuda and co-workers (Scheme 156).^380^ The reaction occurs in the presence of [PdCl_2_(dppf)] (dppf = 1,1′-bis(diphenylphosphino)ferrocene) as a precatalyst, requires the presence of triethylamine, and affords arylboronic esters in yields higher than 80%. Compared with Miyaura borylation, the reaction shows higher atom efficiency and uses a cheaper and more available borylating agent. The drawback of the process is side hydrodehalogenation which can partly be eliminated with the right choice of the base. The reaction was briefly reviewed in 2012.^381^

Similar yields have been reported for aryl halides containing either electron-donating or electron-accepting substituents. A combination of [Pd(dba)_2_] and bis(2-di-*tert*-butylphosphinophenyl) ether was found to be active in the efficient borylation of electron-rich and electron-deficient aryl bromides and electron-rich aryl chlorides with pinacolborane.^382^ The system permitted efficient borylation of bulky aryl bromides. Buchwald has shown that [PdCl_2_(CH_3_CN)_2_] and bulky SPhos used as the supporting ligands achieve effective borylation of several aryl bromides and chlorides.^360^

**Scheme 157:**
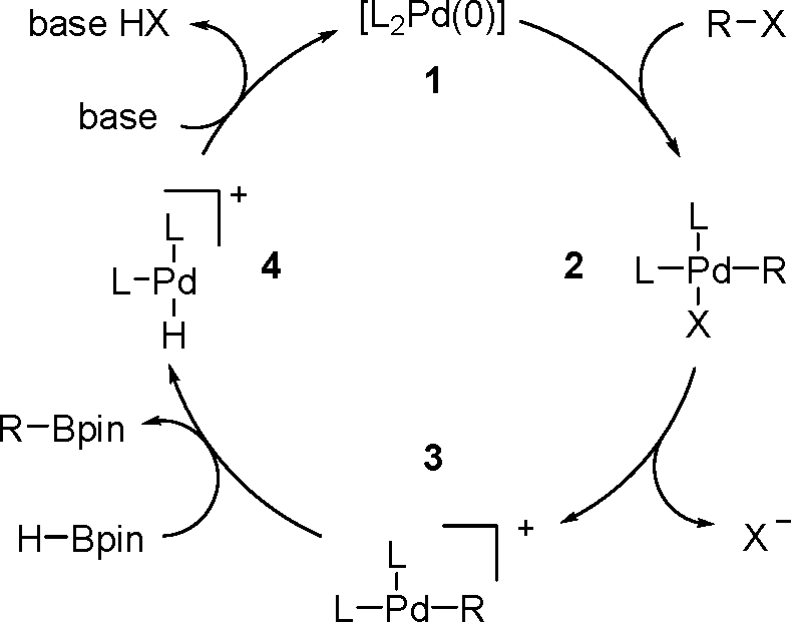
Mechanism of Masuda Borylation^384^

On the basis of DFT computations, the mechanism of the process proposed by Masuda^383^ was corrected by Marder and Lin.^384^ The results of the calculations indicated that the formation of an ammonium/boride ion pair, [Et_3_NH]^+^[Bpin]^−^, is energetically unfavored. The same authors examined the mechanism involving the participation of neutral and cationic palladium complexes using DFT methods. The calculations showed that the cationic pathway was favored over the neutral one. The proposed mechanism (Scheme 157) involves the oxidative addition of an aryl halide to a palladium(0) complex (**1**) leading to compound (**2**). Subsequently, as a result of the NEt_3_-assisted dissociation of the iodide anion, a cationic, coordinatively unsaturated 14-electron complex (**3**), *cis*-[Pd(Ph)(NMe_3_)(η^2^-dhpp)]^+^ (dhpp = PH_2_CH_2_CH_2_CH_2_PH_2_), is formed. Cation **3** reacts with HBpin via σ-bond metathesis to generate an arylboronic ester and cationic palladium hydride (**4**). Finally, base-assisted deprotonation of **4** leads to the regeneration of the active complex (**1**).

**Scheme 158:**
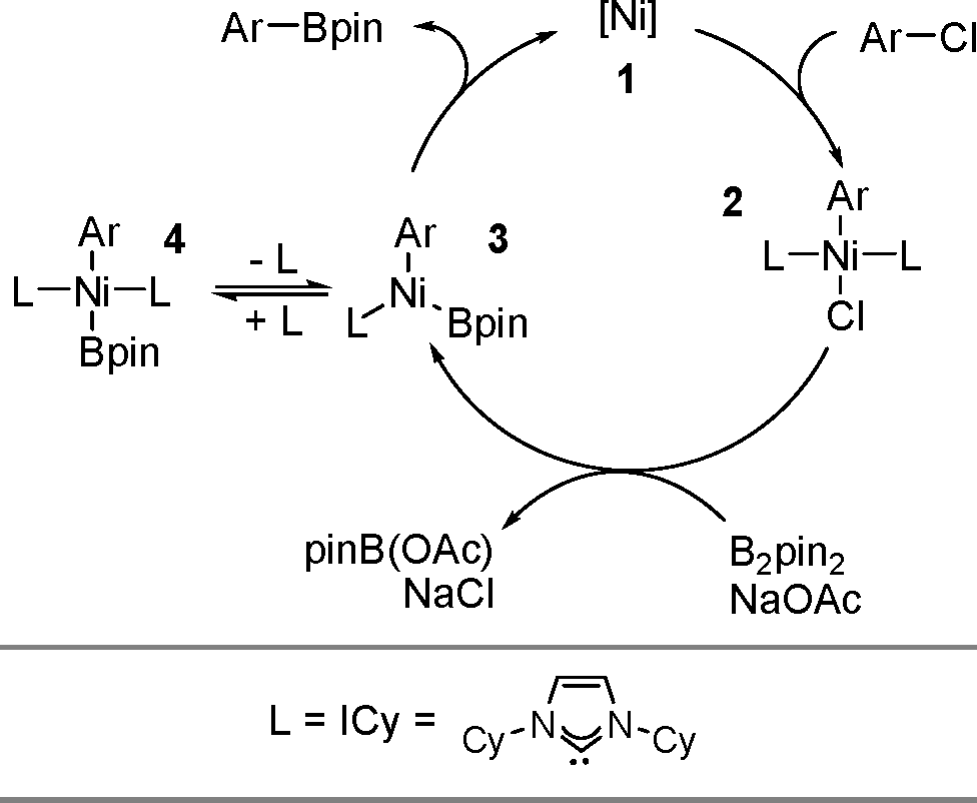
Mechanism of the Ni-Catalyzed Borylation of Aryl Chlorides^387^

Complexes of the first-row transition metals may be an attractive alternative to palladium complexes because of their lower cost and toxicity. Of the 3d group metals, nickel is the most important in catalysis. The [NiCl_2_(PMe_3_)_2_] complex has been found to be active in the borylation of aryl chlorides with bis(pinacolato)diboron in the presence of a metal 2,2,2-trifluoroethoxide as a base.^385^ A variety of functional groups are tolerated. The reaction achieves effective conversion of derivatives with strong steric hindrance. Molander has extended these methods to include the borylation of aryl and heteroaryl halides and pseudohalides using tetrahydroxydiboron.^386^ The reactions are catalyzed by a combination of [NiCl_2_(dppp)], PPh_3_, and diisopropylethylamine. An important feature of the reaction is its functional group compatibility and applicability to several heterocyclic systems. Recently, Marder and Radius have reported the activation and borylation of aryl chlorides using *N*-heterocyclic carbene (NHC) ligated nickel(0) complexes [Ni_2_(ICy)_4_(μ-(η^2^:η^2^)-cod)] (ICy = 1,3-dicyclohexylimidazol-2-ylidene) or [Ni_2_(IPr)_4_(μ-(η^2^:η^2^)-cod)], with NaOAc as the base and B_2_pin_2_ as the boron source.^387^ On the basis of the examination of stoichiometric reactions and the isolation of complexes of the *trans*-[Ni(ICy)_2_(Cl)(Ar)] type, formed in the reaction of [Ni_2_(ICy)_4_(μ-(η^2^:η^2^)-cod)] with different aryl chlorides at room temperature, a reaction mechanism has been proposed (Scheme 158).

The proposed mechanism involves the oxidative addition of an aryl chloride to [Ni(ICy)_2_] (**1**), transmetalation and reductive elimination. The diboron compound undergoes transmetalation, possibly in the form of an adduct, with an acetate anion (Na[B_2_pin_2_(OAc)]). The final reductive elimination step occurs from a three-coordinate species (**3**) to produce the borylated product and to regenerate [Ni(ICy)_2_]. The deduced rate-limiting step is the formation of the boryl complex. The mechanism requires further in-depth experimental and computational research. In 2020, Guo, Radius, and Marder described the visible light-induced Ni-catalyzed radical borylation of chloroarenes with B_2_pin_2_.^375^

**Scheme 159:**
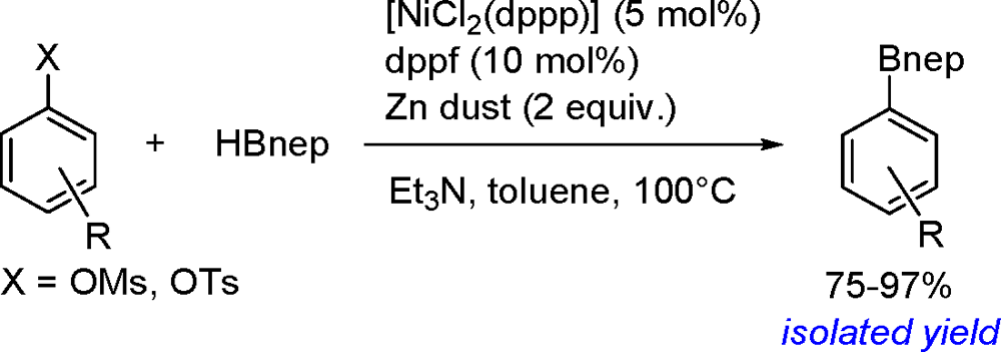
Borylation of Aryl Sulfonates with Neopentylglycolborane Pinacolborane

A mixed-ligand system consisting of [NiCl_2_(dppp)]/dppf permits the borylation of aryl halides with pinacolborane and neopentylglycolborane as borylating agents.^388,389^ This catalytic system combined with Zn strongly increases the reaction rate and yield of aryl neopentylglycolboronates. Diverse electron-rich and electron-deficient aryl iodides, bromides, and chlorides have been efficiently borylated.^390^ Aryl mesylates and tosylates undergo borylation in the presence of a nickel-based catalytic system, using two different phosphine ligands and a zinc additive (Scheme 159).^391^

**Scheme 160:**
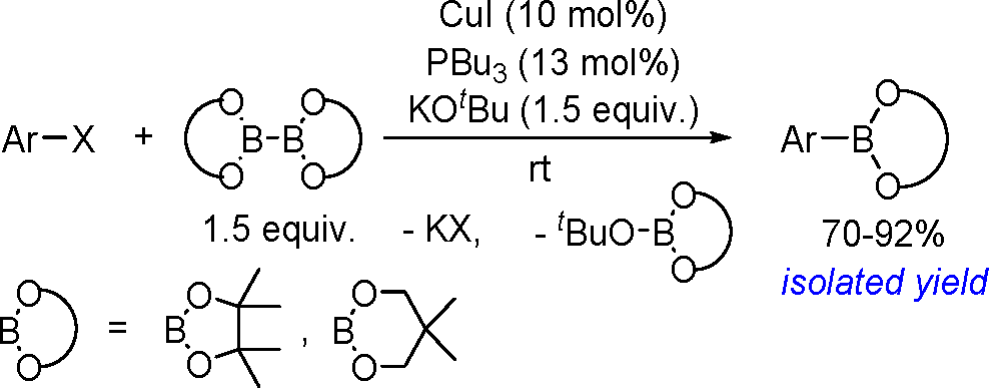
Copper-Catalyzed Borylation of Aryl Bromides

Copper complexes were found to efficiently catalyze the borylation of aryl iodides and electron-rich aryl bromides under mild conditions in the presence of CuI, PBu_3_, or KO*t*-Bu as a base (Scheme 160).^392^

The catalytic cycle of copper-catalyzed borylation proposed on the basis of experimental observations and DFT calculations involves the formation and the key role of Cu–B intermediate. DFT calculations suggest its formation by σ-bond metathesis.^392^

**Scheme 161:**
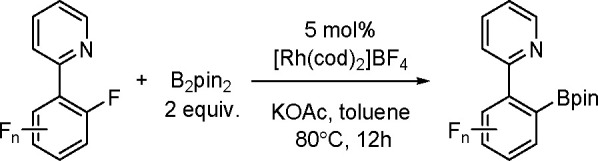
Directed *ortho*-Defluoroborylation of Polyfluoroarenes

Further research established procedures for the efficient borylation of aryl chlorides with different electronic and steric properties.^393^ NHC copper complexes afford isolation yields of 37%–80% in the presence of KO*t*-Bu. The reaction shows a broad functional group tolerance. In the presence of copper iodide and NaH, aryl iodides undergo borylation with pinacolborane (HBpin) as a borylating agent. The reaction is effective already at room temperature and leads to the corresponding aryloboronates in yields of 61%–83%.^394^ Cobalt^395^ and iron complexes^396,397^ have also been found to be active in aryl halide borylation. As in palladium catalysis the formation of a metal-boryl complex is of key importance for catalysis with Ni, Cu, Fe, and Co complexes. Recently, the borylation of aryl iodides with B_2_pin_2_ has been reported to proceed in the presence of the rhodium complex [{RhCl(cod)}_2_] and KOAc as a base.^398^ In 2015, Zhang described the directing-group-assisted *ortho*-selective defluoroborylation of polyfluoroarenes with B_2_pin_2_ (Scheme 161).^399^ Several di-, tri-, tetra-, and pentasubstituted fluoroarenes were transformed with different isolation yields, typically exceeding 70%.

On the basis of preliminary studies of the reaction mechanism, a Rh(III)/Rh(V) catalytic cycle was suggested as involved in the reaction. Nonactivated fluoroarene was borylated with B_2_nep_2_ in the presence of a nickel catalyst.^400^ On the basis of results of preliminary studies, a mechanism was proposed, involving the oxidative addition of an aromatic C–F bond to a Ni(0) complex followed by boryl transfer and C–B bond formation via reductive elimination. The nickel complex was reported to catalyze fluoroarenes with B_2_pin_2_ when used in the presence of a copper(I) salt.^401^ However, examination of this reaction suggested that the Ni(I) complex was involved in the activation of the C–F bond. Marder and Radius used the [Ni(IMes)_2_] *N*-heterocyclic carbene nickel complex in the borylation of multifluorinated arenes with B_2_pin_2_.^402^ Several partially fluorinated arenes were converted into their corresponding boronate esters. It was demonstrated that the formation of monoborylation and diborylation products as the main product can be controlled by the fluoroarene to B_2_pin_2_ ratio.

Copper complexes have been found to catalyze the C–F borylation of mono-, di-, and trifluoroarenes.^403^ Mechanistic studies suggest that the reaction occurs via an SRN1 mechanism^404^ involving a single-electron transfer process. In the presence of lithium bis(silyl)amide (LiHMDS) as a base, both palladium complexes ([Pd_2_dba_3_]) and iron salts (FeCl_2_) exhibit catalytic activity in the borylation of an aryl fluoride with B_2_pin_2_.^405^ The proposed mechanism comprised oxidative addition, base-assisted transmetalation, and reductive elimination of aryl boronate. LiHMDS was proposed to play a vital role in the activation of the B–B bond and in the transmetalation step.

Guo, Radius, Marder, and co-workers described the selective photocatalytic C–F borylation of polyfluoroaromatics with B_2_pin_2_ in the presence of an NHC nickel catalyst.^406^ The protocol employed a rhodium–biphenyl complex as a triplet sensitizer. Finally, Shi reported the copper-catalyzed multiborylation of *gem-*difluoroalkenes.^407^

#### 4.1.3 Borylation of C(sp^3^)–X Bonds

**Scheme 162:**
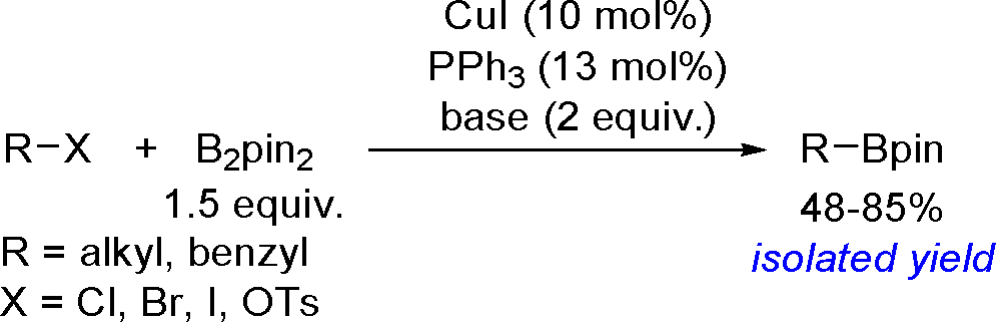
Copper-Catalyzed Borylation of Inactivated Alkyl Halides

The reaction is an alternative synthetic method for the Csp^3^–B bond. The most important information on this reaction has been published in a recent review.^376^ Catalytic activity in this reaction has been observed for the salts or complexes of several TMs, including Co, Cu, Fe, Mn, Ni, and Pd; most papers concern copper-based catalysis. Advances in Cu-catalyzed C(sp^3^)–B bond formation have recently been reviewed.^408^ The first reported example of the reaction was the borylation of activated allyl chlorides with bis(pinacolato)diboron, and stoichiometric amounts of a copper(I) salt had to be used.^409^ Inactivated alkyl halides were found to undergo borylation in the presence of 10 mol % coper(I) iodide, PPh_3_, and a base (Scheme 162).^410^ The reaction provides primary and secondary alkylboronic esters with diverse structures and a variety of functional groups.

**Scheme 163:**
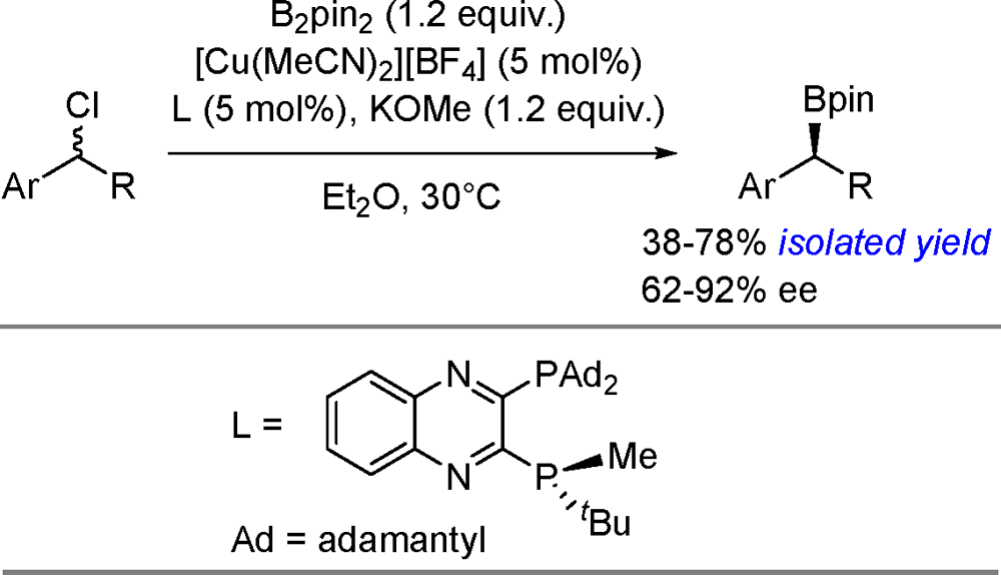
Enantioconvergent Borylation of Racemic Benzyl Chlorides

Subsequent results expanded the scope of the reaction. In the presence of copper(I)/Xantphos, primary and secondary alkyl chlorides, bromides, and iodides undergo efficient borylation with B_2_pin_2_.^411^ A radical mechanism was postulated on the basis of a reaction with cyclopropylmethyl bromide, which resulted in the formation of ring-opening products. Recently, Ito has reported the first copper(I)-catalyzed enantioconvergent borylation of racemic benzyl chlorides (Scheme 163).^412^ Benzyl boronates were obtained with high enantioselectivity (up to 92% ee) and isolation yields, depending on the substrate and reaching 78%.

**Scheme 164:**
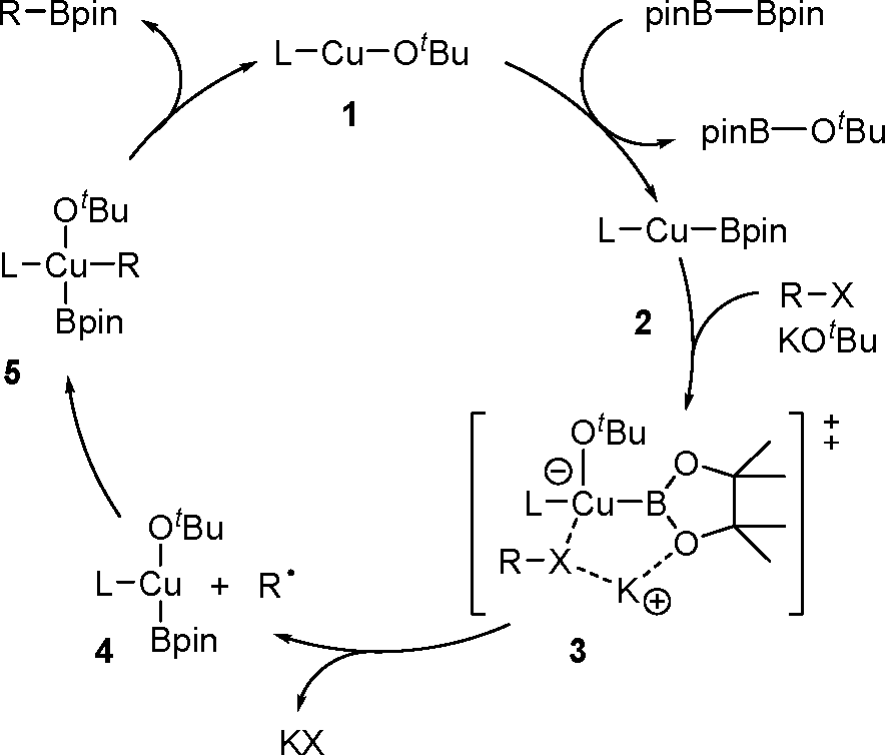
Mechanism of Alkyl Halide Borylation in the Presence of a Copper Complex

Although the detailed mechanism of the process is not known and further experimental and computational research is necessary, Ito has proposed a reasonable reaction mechanism (Scheme 164).^376^

According to the mechanism, a copper(I) boryl complex is generated when a copper(I) alkoxide (**1**) reacts with a diboron compound. The proposed oxidative addition takes place in the presence of a base and leads, in the first stage, to a copper(II) complex (**4**) and an alkyl radical as a result of single-electron transfer between the alkyl halide and copper(I). The association of the radical in the second stage generates a copper(III) complex (**5**). Subsequent reductive elimination gives the borylation product and leads to the regeneration of copper(I) alkoxide (**1**).

**Scheme 165:**
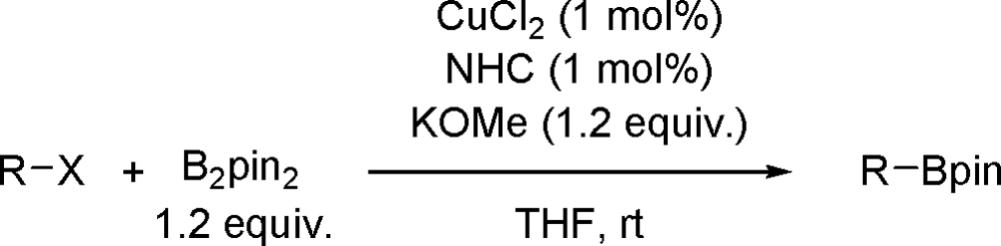
Copper-Catalyzed Borylation of Alkyl Halides

Copper(II) salts in the presence of NHC (IMes, IPr) were proved to be catalytically active in the borylation of primary, secondary, and tertiary alkyl halides with B_2_pin_2_ (Scheme 165).^413^

The procedure enables the borylation of a wide range of primary, secondary, and some tertiary alkyl halides to give corresponding alkyl boronates in good yields. Allyl and benzyl bromides can be converted into the desired products in moderate to good yields. The reaction tolerates a range of functional groups, such as ethers, ketals, esters, alcohols, and nitriles. Preliminary mechanistic studies suggest that the reaction mechanism involves the formation of an alkyl radical. The advances and perspectives of Cu-catalyzed C(sp^3^)–B bond formation have recently been discussed.^408^

Allyl chlorides undergo borylation with bis(pinacolato)diboron in the presence of PdCl_2_ or [Pd_2_(dba)_3_]).^414^ [Pd_2_(dba)_3_] used with P(*t*-Bu)_2_Me·HBF_4_ is active in the borylation of primary alkyl bromides.^415^ The process enables the synthesis of primary alkyl boronate esters with various skeletons and functional groups.

**Scheme 166:**
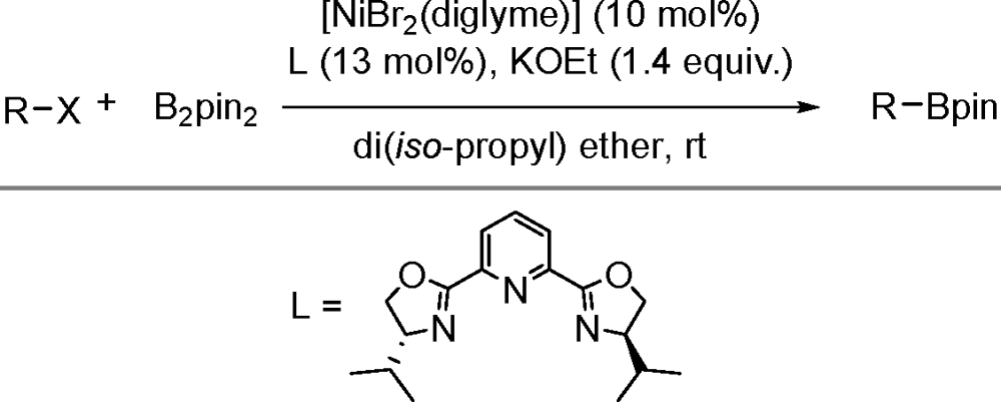
Nickel-Catalyzed Borylation of Alkyl Halides

Fu group has reported borylations of inactivated primary, secondary, and tertiary alkyl halides (iodides, bromides, and chlorides) in the presence of nickel complexes (Scheme 166).^416^ The method enables highly efficient and regiospecific synthesis of a large variety of alkyl boronates.

**Scheme 167:**
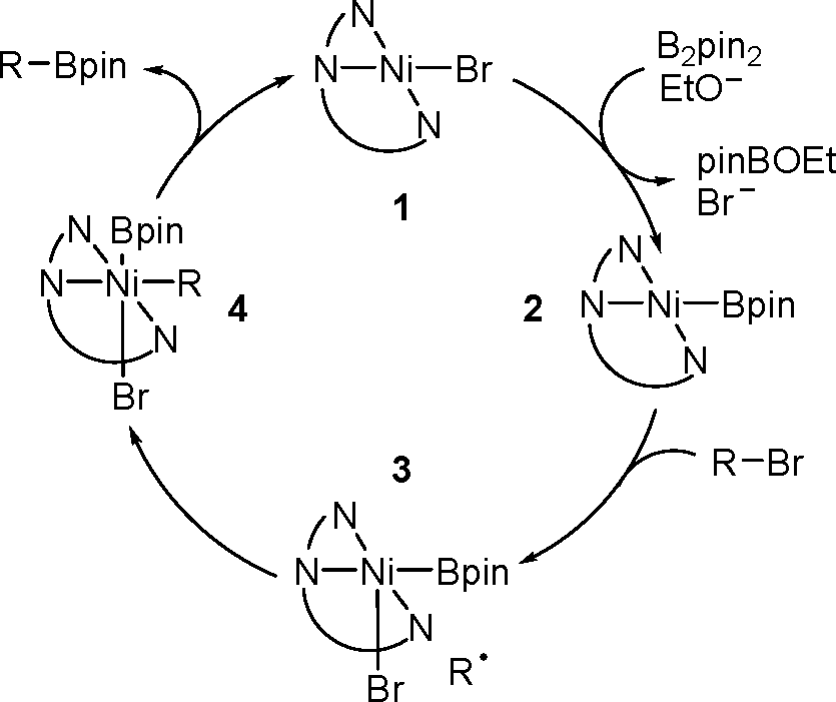
Mechanism of the Nickel-Catalyzed Borylation of Alkyl Halides

The observed reactivity trend for primary, secondary, and tertiary halides (more substituted electrophiles are more reactive) and stereochemical probes^417^ suggest the involvement of an organic radical in the mechanism (Scheme 167).^416^

The proposed mechanism is supported by the results of DFT calculations.^418^ The calculated energy diagram indicates that the transfer of a halogen atom to the metal is the rate-limiting step. According to the calculation results, the barrier to atom transfer increases with a decreasing substitution degree of the alkyl bromides, which explains the experimentally observed reactivity trend.^416^ Involvement of organic radicals in the reaction mechanism of alkyl halide borylations catalyzed by Mn,^419^ Fe,^420^ or Co^421^ has also been postulated.

### 4.2 Silylation of C–X Bonds

Catalytic silylation of the C–X bond is a versatile method for synthesizing silyl-substituted organic derivatives. Disilanes, tertiary silanes (HSiR_3_), and silylboranes (PhMe_2_Si-Bpin) can be used as silylating agents. The reaction is typically used in the synthesis of silylarenes characterized by high site selectivity.

#### 4.2.1 Silylation of Olefinic C(sp^2^)–X Bonds

**Scheme 168:**
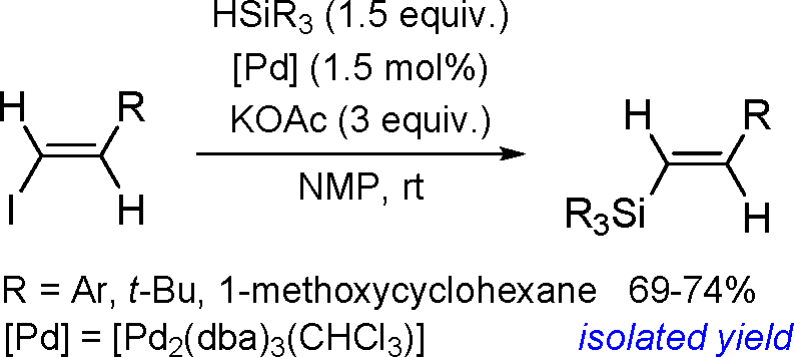
Palladium-Catalyzed Silylation of Vinyl Iodides with Tertiary Silanes

In 1978, Nagai demonstrated the synthesis of vinylsilanes and bis(silyl)ethenes via the coupling of vinyl chloride or *trans*-1,2-dichloroethylene with disilane in the presence of [Pd(PPh_3_)_4_].^422^ The palladium(0)-catalyzed silylation of vinyl iodides with HSiR_3_ has been reported by the Masuda group.^423^ The reaction permits the synthesis of alkenylsilanes with yields of up to approximately 70%. Reaction selectivity depends on the nature of reagents (both tertiary silanes and olefins), and for some systems, alkylsilane is observed as a byproduct (Scheme 168).

**Scheme 169:**
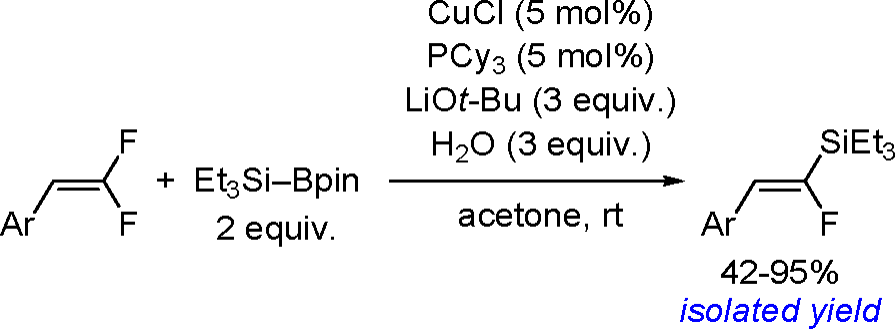
Copper-Catalyzed Defluorosilylation of *gem*-Difluoroalkenes with Silaboronate

*gem*-Difluoroalkenes undergo stereoselective defluorosilylation with Et_3_SiBpin in the presence of copper(I) chloride and PCy_3_ (Scheme 169).^365^ The reaction leads exclusively to Z-monofluoroalkenylsilanes.

#### 4.2.2 Silylation of Aryl C(sp^2^)–X Bonds

**Scheme 170:**
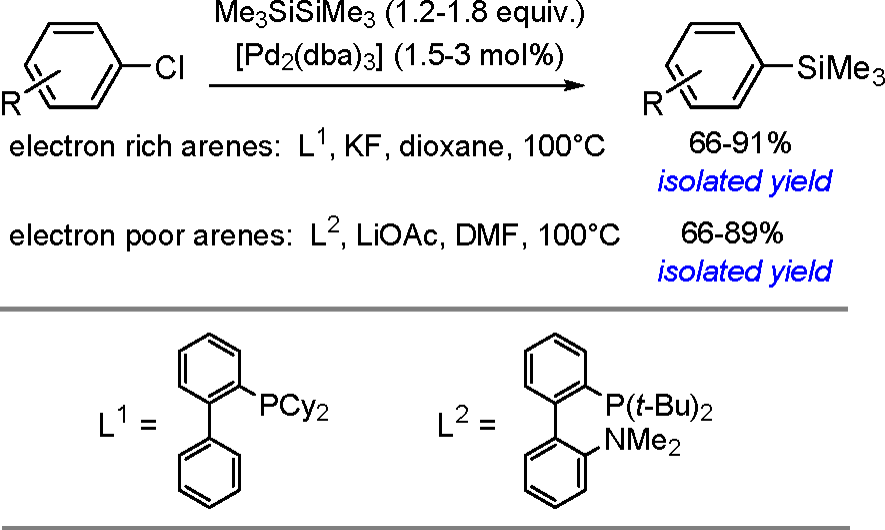
Palladium-Catalyzed Silylation of Aryl Chlorides with Disilane

Silylation of aryl halides with disilanes was reported already in 1975 by Matsumoto and co-workers who described the catalytic activity of [Pd(PPh_3_)_4_] in the silylation of aryl bromides and certain chlorides with hexamethyldisilane.^424^ Eaborn extended the scope of the reaction by demonstrating the germylation and stannylation of aryl bromides with digermane and distannane, respectively.^425^ Tsuji^426^ and Buchwald (Scheme 170)^427^ reported catalytic systems active in the silylation of aryl chlorides so that the reaction provided access to a wide variety of arylsilanes from commercially available aryl chlorides. The method produced the desired silylarenes in good yields and tolerated a variety of functional groups.

**Scheme 171:**
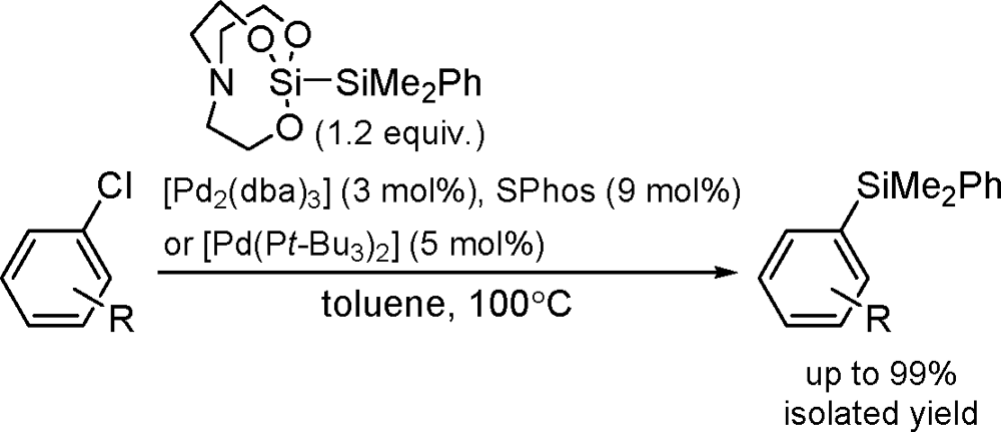
Silylsilatrane as a Silylating Agent

The Denmark group has described the palladium-catalyzed coupling of a variety of aryl bromides with 1,2-diethoxy-1,1,2,2-tetramethyldisilane (EtO)Me_2_Si-SiMe_2_(OEt) for the synthesis of dimethylalkoxyarylsilanes that are active reagents in Hiyama–Denmark coupling.^428^ An analogous process using aryl chlorides has also been described.^429^ The palladium-catalyzed silylation of aryl chlorides with silylsilatranes proceeds under external base-free conditions (Scheme 171).^430^ Experimental and computational studies show that smooth transmetalation from a silylsilatrane to arylpalladium chloride is facilitated by a strong interaction between the Lewis acidic silicon and the chloride.

**Scheme 172:**
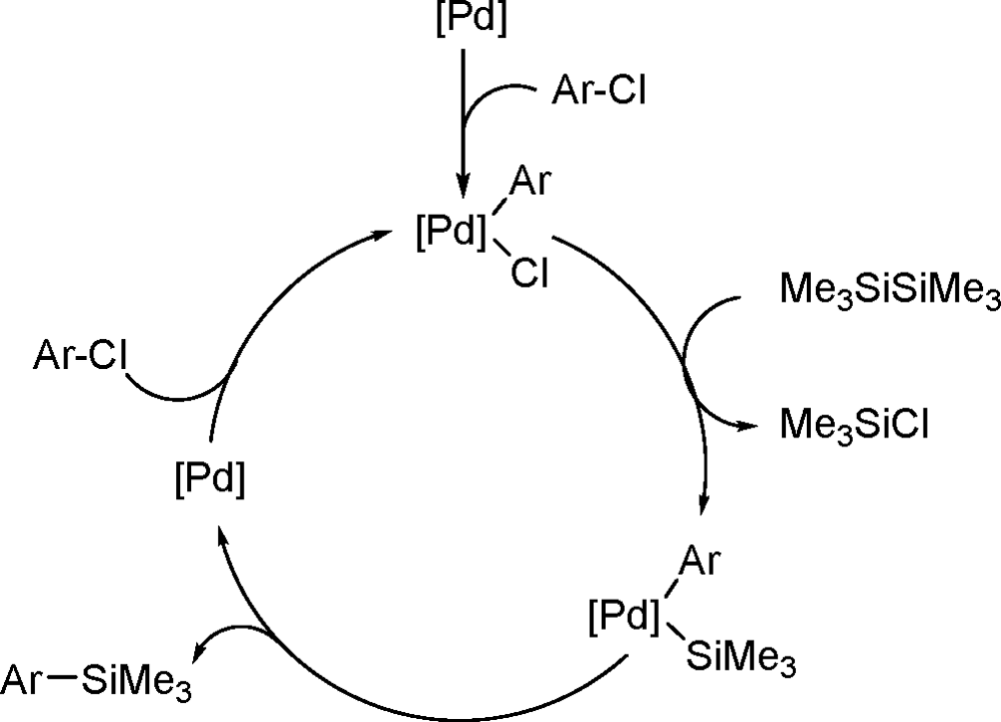
Mechanism of the Silylation of Aryl Halides with Disilanes^431^

The reaction mechanism has been presented first by Matsumoto et al. (Scheme 172);^431^ however, it should be modified to take into account the beneficial effect of a base, in particular KOAc.

**Scheme 173:**
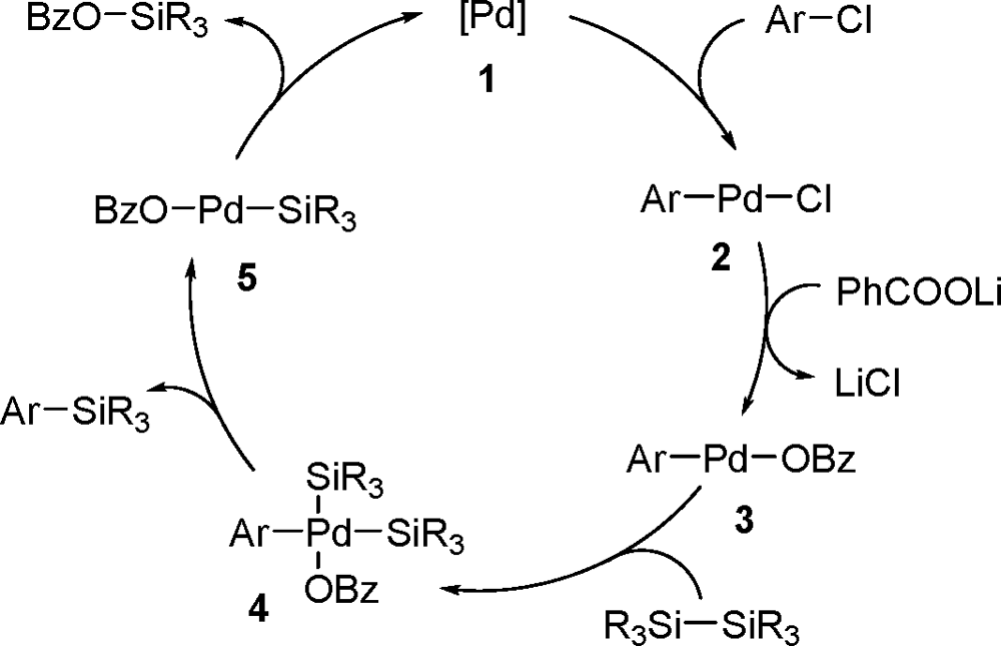
Mechanism of the Pd-Catalyzed Silylation of Aryl Chlorides with Disilanes

Shimokawa proposed possible involvement of palladium(IV) complexes in the reaction mechanism (Scheme 173).^429^

However, it may be the case that disilane undergoes transmetalation with a Pd(II) carboxylate to give silyl benzoate and an Ar–Pd(II)–SiMe_2_(O*t-*Bu) species, which would then form the arylsilane via reductive elimination and regenerate the Pd(0) complex.^429^

**Scheme 174:**
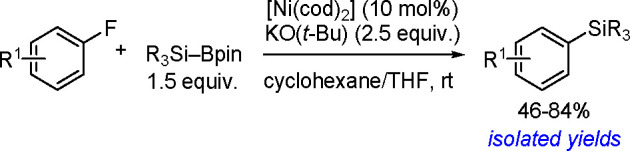
Silylation of Aryl Fluorides with Silaborane

In 1998, the Murai group reported the functional group-directed defluorosilylation of aryl fluorides, fluoroacetophenones and (fluorophenyl)oxazolines with hexamethyldisilane in the presence of [Rh(cod)_2_][BF_4_].^432^ Recently, Shibata presented an efficient Ni-catalyzed defluorosilylation of fluoroarenes with the R_3_SiBpin silylborane, providing arylsilanes in good to high yields. In the presence of [Ni(cod)], KO*t*-Bu and without an additional ligand, the activation and functionalization of the C–F bond occurs at room temperature (Scheme 174).^433^

Benzyl and alkyl fluorides are silylated at room temperature in the absence of a nickel complex. Results of experimental studies (free radical clock, effect of radical scavengers) suggest that the mechanism of defluorosilylation does not involve free radical intermediates. The reaction mechanism has been proposed, but further studies are needed for corroboration.

**Scheme 175:**
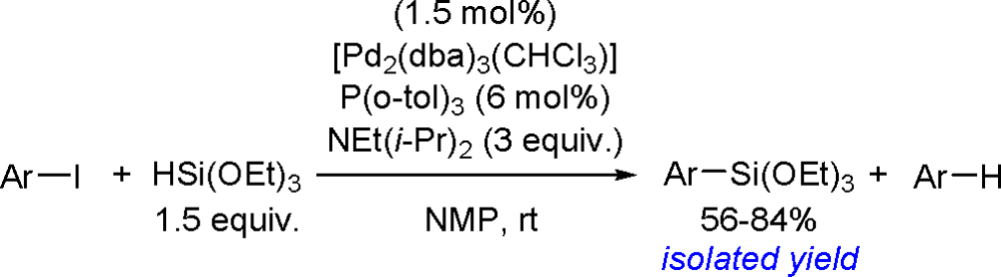
Silylation of Aryl Iodides with HSi(OEt)_3_

Silylation with tertiary silanes (HSiR_3_) carries the risk of the competing hydrodehalogenation reaction. However, a few catalytic systems for this reaction have been described, showing high selectivity for the formation of arylsilanes. The advantages, drawbacks, and the scope of the reaction have been reviewed.^300^ In 1997, Masuda’s group described the first example of the palladium-catalyzed silylation of para-substituted aryl iodides with triethoxysilane leading to arylsilanes (Scheme 175).^434^ The use of [Pd_2_(dba)_3_(CHCl_3_)], P(*o*-tol)_3_, and NEt(*i-*Pr)_2_ permitted high silylation yields for electron-rich (hetero)aryl iodides. The presence of electron-accepting substituents in the aryl ring has significantly increased the yield.

**Scheme 176:**
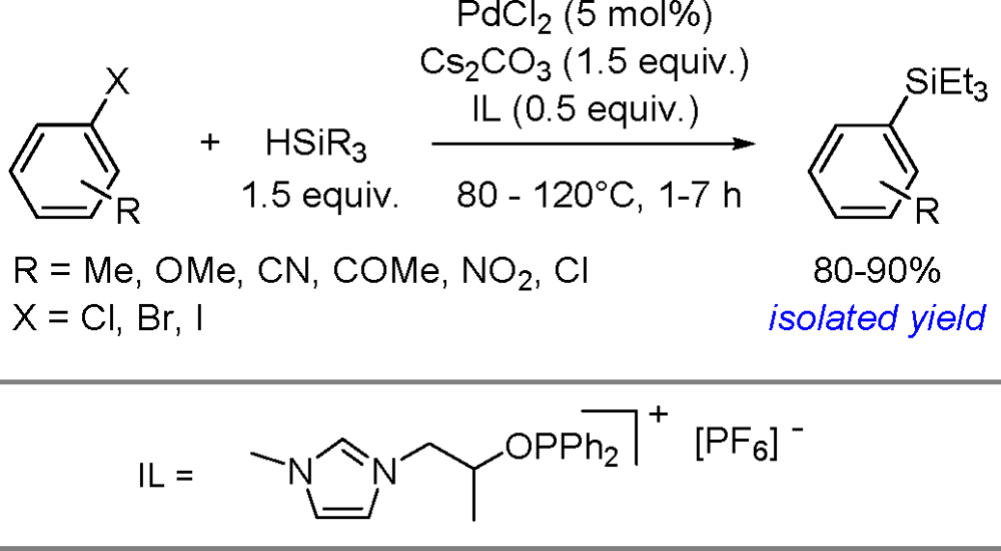
Palladium-Catalyzed Silylation of Aryl Halides with HSiEt_3_

Different substituted tertiary silanes, for example, tri(furan-2-yl)silane, tri(thiophen-2-yl)silane, Ph_3_SiH, and Ph_2_MeSiH, have been successfully used as silylating agents in the presence of [Pd_2_(dba)_3_(CHCl_3_)]/P(*o-*tol)_3_/NEt(*i-*Pr)_2_ in mild conditions.^435^ Efficient silylation of aryl chlorides (as well as iodides and bromides) with triethylsilane has been observed in a system containing PdCl_2_, imidazolium-based phosphinite ionic liquid, and cesium carbonate as a base (Scheme 176).^436^ Silylation in this system occurs with a minor contribution of hydrodehalogenation.

Presently, the reaction achieves effective silylation of aryl iodides, bromides, and chlorides with a variety of tertiary and some secondary silanes. The reaction proceeds more readily for electron-rich arenes and at less sterically crowded positions (e.g., para and meta positions in the aromatic ring). The reaction also permits the syntheses of optically active tertiary silanes via the enantioselective arylation of secondary silanes.^437^

**Scheme 177:**
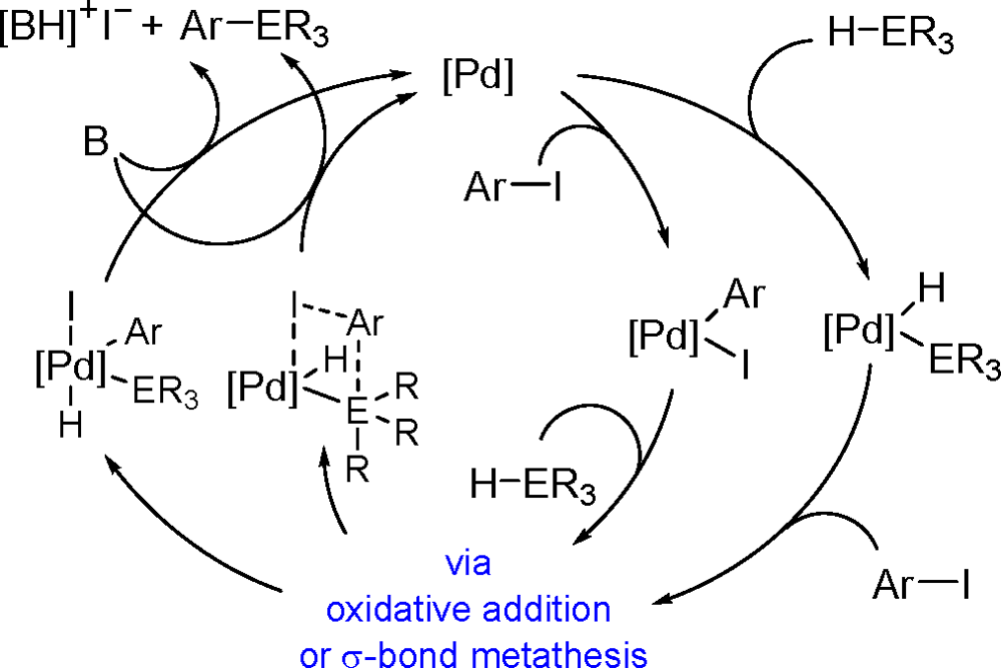
Current Mechanistic Understanding of the Silylation of Aryl Halides with Tertiary Silanes (HSiR_3_)

No mechanism supported by convincing experimental evidence and quantum-chemical calculations has been proposed so far for the silylation of aryl halides with tertiary silanes. The present knowledge of the reaction mechanism is illustrated in Scheme 177.

Results of DFT calculations^438^ suggest that the preferred mechanism depends on the reaction conditions, composition of the reaction system, and stereoelectronic properties of the catalyst. The suggested first step is the oxidative addition of an aryl iodide to a Pd(0) complex. Subsequently, a silylation product or a hydrodehalogenation product may form by σ-bond metathesis. A cycle involving palladium(IV) complex formation and its further conversion by reductive elimination cannot be ruled out. Further research is needed to clarify the ambiguities of the reaction mechanism.

**Scheme 178:**
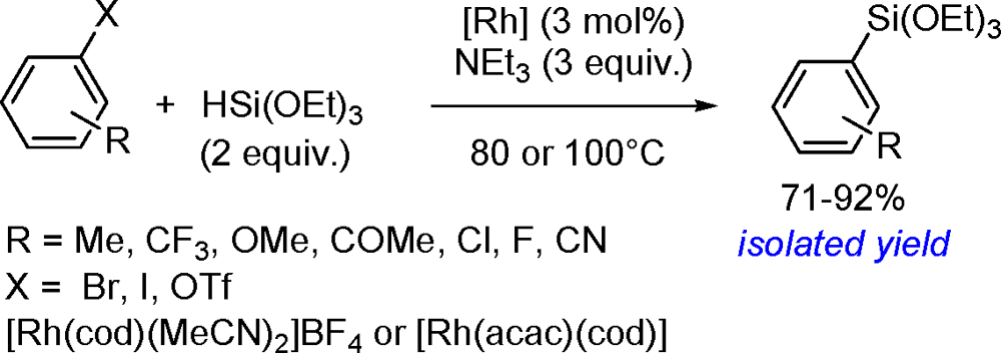
Rhodium-Catalyzed Silylation of Aryl Halides with HSi(OEt)_3_

In addition to palladium-based systems, the reaction is catalyzed by rhodium complexes. In each case, competition between silylation and reduction of the aromatic ring has been observed. The first selective silylation of aryl iodides and bromides with triethoxysilane (EtO)_3_SiH in the presence of catalytic amounts of [Rh(cod)(MeCN)_2_][BF_4_] and NEt_3_ has been described by Murata and Masuda.^439^ The Rh-catalyzed silylation process leads to the general and efficient silylation of aryl halides with triethoxysilane in the presence of a rhodium catalyst (Scheme 178).^440^ Aryl iodide, bromide, and trifluoromethylsulfonate are efficiently silylated with HSi(OEt)_3_ in the presence of [Rh(cod)(MeCN)_2_][BF_4_] or [Rh(acac)(cod)]. The substrate scope includes electron-rich and -deficient aryl halides with different substitution patterns. Rhodium catalysts ensure efficient silylation at sterically crowded positions (e.g., in *ortho*-substituted aryl halides).

A variety of aryltriethylsilanes have been synthesized in the presence of a rhodium-based catalytic system consisting of [Rh(cod)_2_][BF_4_] or [RhCl(CO)(PPh_3_)_2_], K_3_PO_4_ and *N*-methylpyrrolidinone (NMP) as a solvent.^441^ The catalytic system is active already at room temperature and is highly tolerant to various sensitive functional groups on the substrates.

**Scheme 179:**
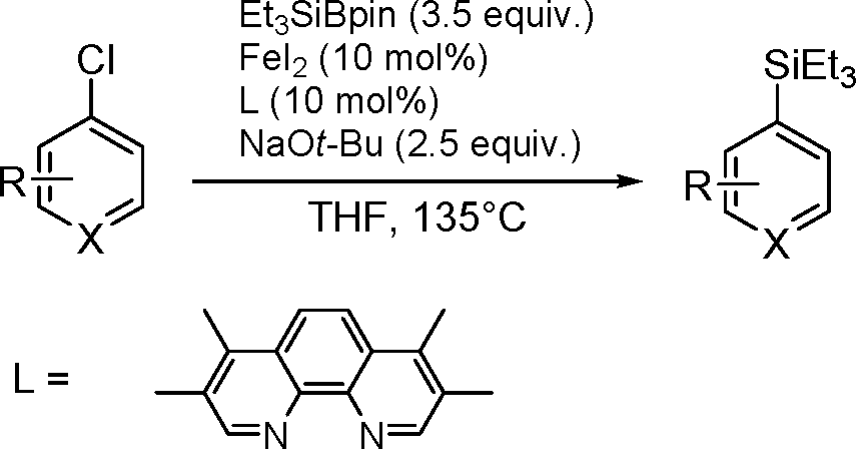
Iron-Catalyzed Silylation of Aryl Halides with HSi(OEt)_3_

Recently, an iron-catalyzed method for the silylation of (hetero)aromatic chlorides has been reported (Scheme 179). The protocol offers high efficiency, a broad substrate scope, and good functional group compatibility.^442^

#### 4.2.3 Silylation of C(sp^3^)–X Bonds

**Scheme 180:**
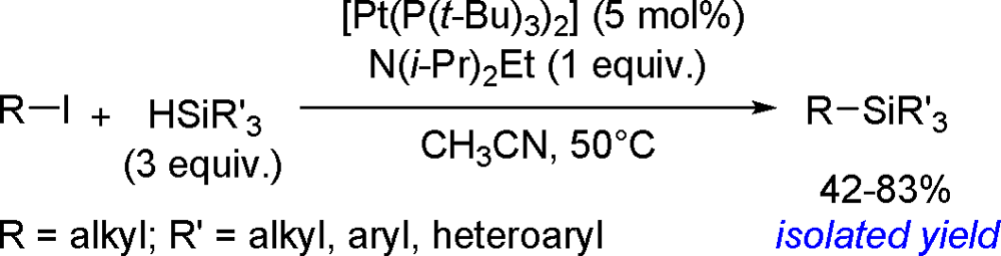
Platinum-Catalyzed Silylation of Alkyl Iodides with Tertiary Silanes

Palladium, platinum, iron, cobalt, nickel, and copper salts or complexes have been proved to have catalytic activity in the silylation of alkyl halides or sulfonates. Whereas palladium and platinum complexes are able to catalyze silylation with disilanes (Pd) or tertiary silanes (Pt), the first-row transition metals are active in the presence of silylborane or silyl-derivatives of organozinc and organomagnesium compounds. Copper-catalyzed C(sp^3^)–Si bond-forming reactions have recently been reviewed.^408^ As early as in 1983, Nagai reported that benzyl chlorides were silylated with Cl_2_MeSiSiMeCl_2_ in the presence of [Pd(PPh_3_)_4_]. The corresponding benzyldichloromethylsilanes were obtained in high GLC yields (up to 98%).^443^ Nonactivated alkyl iodides react in the presence of [Pt(P(*t*-Bu)_3_)_2_]/N(*i*-Pr)_2_Et with tertiary silanes to give alkylsilanes in moderate to good isolation yields (Scheme 180).^444^

More recent procedures for C–Si bond formation use nucleophilic silylating agents, for example, Me_2_PhSiBpin (Suginome’s reagent). The methods based on silylborane reagents have recently gained importance in organic synthesis.^202,445^ Primary and secondary benzyl halides undergo silylation with Me_2_PhSiBpin in the presence of only 2 mol % of the [Pd(PPh_3_)_4_]/Ag_2_O palladium catalyst at room temperature.^446^ The reaction permits synthesis of differently substituted benzylphenyldimethylsilanes with yields of 49%–95%.

**Scheme 181:**
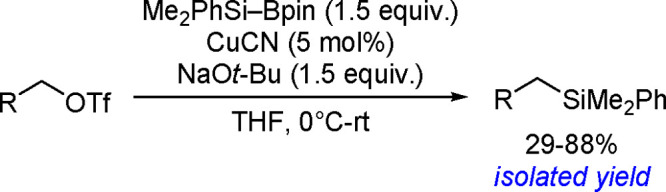
Copper-Catalyzed Silylation of Alkyl Triflates with Me_2_PhSiBpin

Primary alkyl triflates undergo silylation with Me_2_PhSiBpin in the presence of copper(I) cyanide (Scheme 181).^447^

**Scheme 182:**
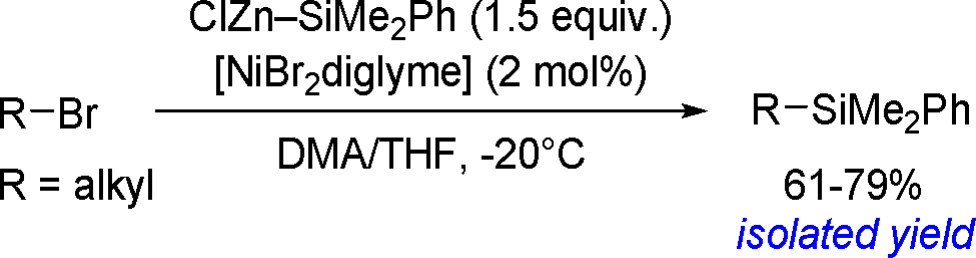
Silylation of Inactivated Secondary and Tertiary Alkyl Halides

The first metal-catalyzed cross-coupling reactions of inactivated secondary and tertiary alkyl electrophiles to form C–Si bonds were carried out using a nickel-based catalyst and ClZn–SiMe_2_Ph as a silylating agent (Scheme 182).^448^ The reaction proceeded efficiently under very mild conditions and tolerated a variety of functional groups.

**Scheme 183:**
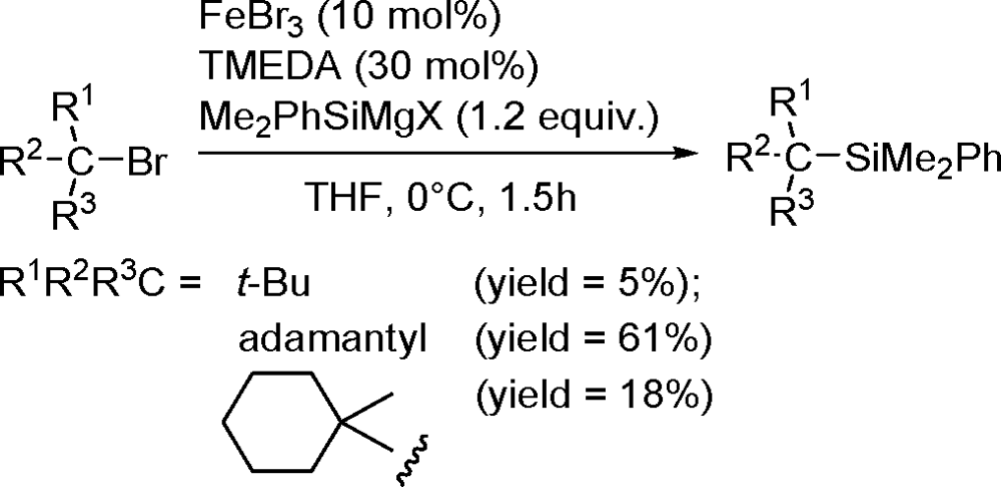
Fe-Catalyzed Silylation of Tertiary Alkyl Bromides with a Silyl Grignard Reagent

Iron and cobalt salts are active in the silylation of primary, secondary, and tertiary alkyl bromides with silyl Grignard reagents of the R_3_SiMgX type (Scheme 183).^449^

### 4.3 Germylation of C–X Bonds

**Scheme 184:**
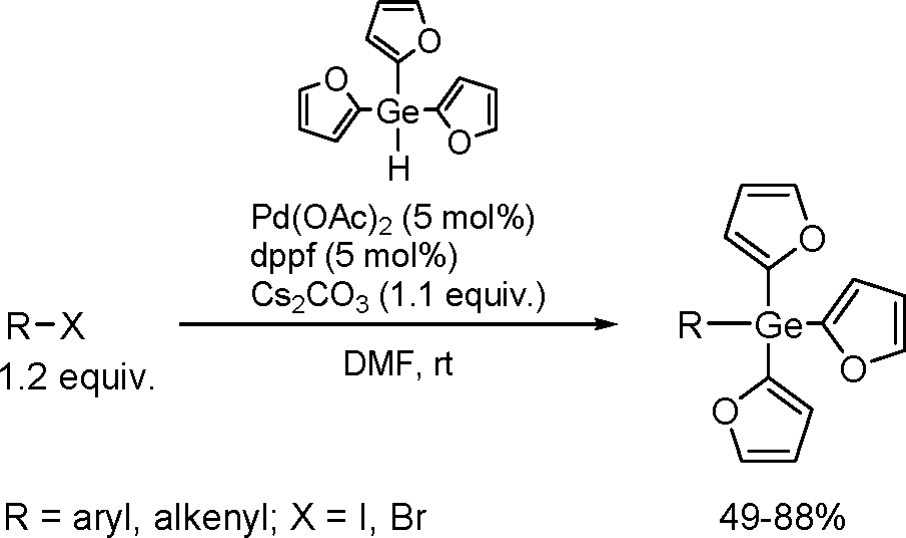
Germylation of Aryl Halides with Tri(2-furyl)germanes

Germylation of olefins was attempted first by Tanaka and co-workers.^450^ Palladium-catalyzed germylation of (*E*)-(2-bromovinyl)benzene and (*E*)-1,2-dichloroethene with ClMe_2_GeGeMe_2_Cl gave yields close to 30%. The first germylation of aryl bromides with Et_3_GeGeEt_3_ was reported by Eaborn.^425^ PhGeEt_3_ and 4-Me-C_6_H_4_GeEt_3_ were synthesized with yields of 28% and 35%, respectively. Tanaka used similar conditions and employed ClMe_2_GeGeMe_2_Cl; however, the reaction was nonselective, and the yields did not improve.^450^ In 2018, Yoshino and Matsunaga described the germylation of aryl bromides and aryl triflates using commercially available hexamethyldigermane.^451^ In optimized reaction conditions (typically, Pd(OAc)_2_, 2-(diphenylphosphanyl)phenol, Cs_2_CO_3_, Et_4_NBr, toluene, 120 °C, 24 h), moderate to good yields (up to 87%) of the corresponding aryltrimethylgermanes were obtained. Tri(furan-2-yl)germane readily reacts with aryl iodide, α- and β-bromostyrene, 1- and 2-bromopropene and 2-bromopyridine in the presence of palladium(II) acetate, 1,1′-bis(diphenylphosphino)ferrocene (dppf) and cesium carbonate (Scheme 184).^452^ Aryltri(2-furyl)germane (formed directly before the reaction) can be used as a reacting partner in palladium-catalyzed coupling with aryl iodide or bromide.

Monoarylation of tertiary germanes, diarylation of secondary germanes and stepwise arylation of secondary and primary germanes with aryl iodides were tested in the presence of [Pd{P(*t*-Bu)_3_}_2_] and a base in THF under mild conditions.^453^ Depending on the reacting partners, the yields varied in the range of 20%–63%. However, the reaction suffered from the lack of selectivity.

**Scheme 185:**
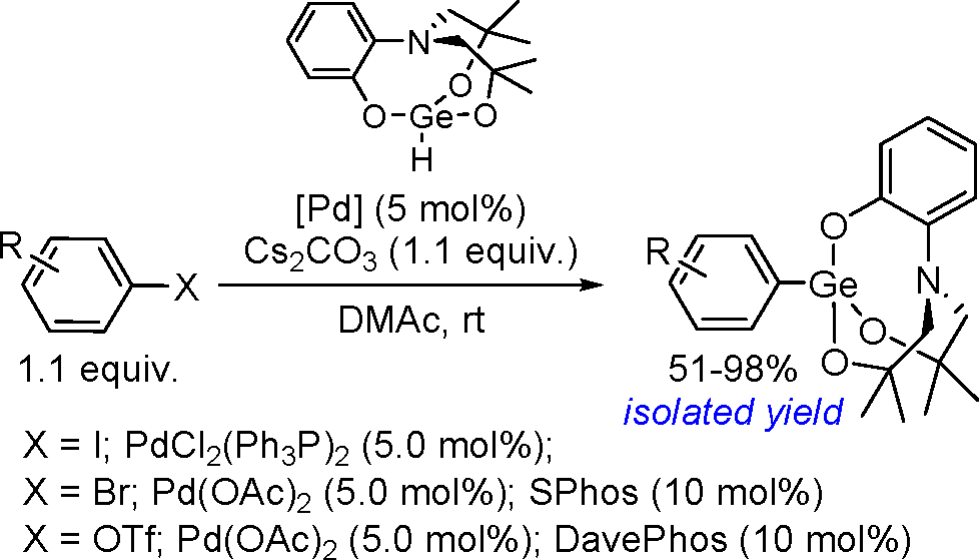
Germatranes as Germylating Agents

Pd-catalyzed germatranization was shown to be an efficient method for the formation of arylgermatranes (Scheme 185).^454^

Arylgermatranes can be subsequently used for palladium-catalyzed cross-coupling with aryl halides.

**Scheme 186:**
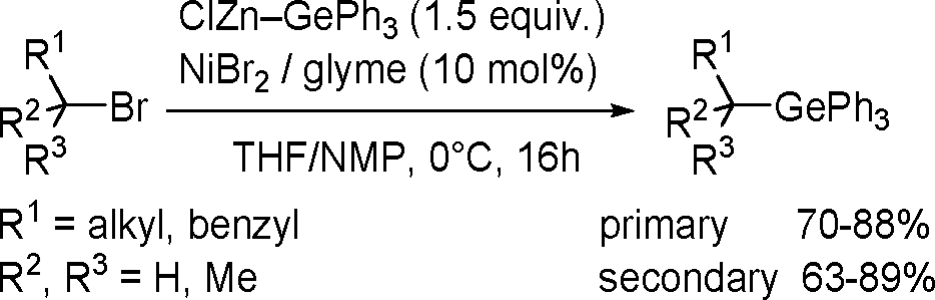
Nickel-Catalyzed Germylation of Primary and Secondary Alkyl Bromides

Recently, Oestreich and co-workers have reported methods for the synthesis of C(sp^3^)–Ge bonds from alkyl electrophiles and germanium nucleophiles.^455^ A germanium Grignard reagent was synthesized and shown to be active in reactions with primary and secondary alkyl bromides. The corresponding germanium zinc reagent (Ph_3_GeZnCl) undergoes a nickel-catalyzed reaction with primary, secondary, and, less successfully, tertiary alkyl bromides (Scheme 186).

Silylation of tertiary alkyl bromides proceeds less efficiently and is accompanied by C–H bond activation and borylation. Mechanistic studies, in particular the ring opening observed in a radical-probe experiment using (bromomethyl)cyclopropane, the loss of the stereochemical information in the C(sp^3^)–Ge coupling of an enantioenriched secondary alkyl bromide, and the observed order of reactivity of alkyl bromides (tertiary > secondary > primary) strongly support the involvement of alkyl radical intermediates in the reaction mechanism.

### 4.4 Stannylation of C–X Bonds

**Scheme 187:**
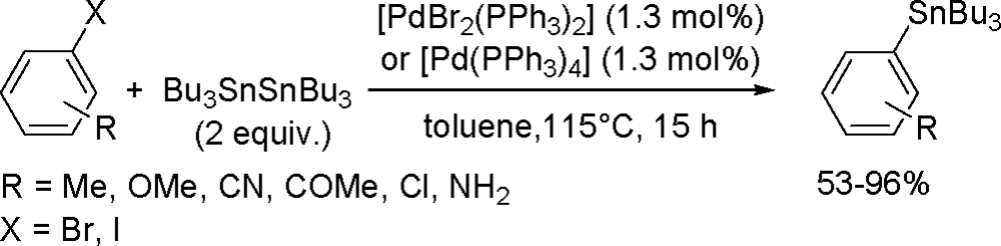
Stannylation of Aryl Halides with Distannanes

The first examples of the stannylation of aryl halides with distannanes have been reported by Eaborn.^425^ In the presence of [Pd(PPh_3_)_4_] at 120 °C in toluene, 1-bromo-4-methoxybenzene reacts with hexamethyldistannane or hexabutyldistannane to give corresponding arylstannanes. The reaction was accompanied by the competitive formation of biaryls. As a result of further studies, the activity of aryl iodides and bromides containing several functional groups—such as OMe, Cl, CN, COCH_3,_ NO_2_, and bromide and chloride allyl and benzyl derivatives—in the reaction was proved (Scheme 187).^456^ The achievements in the palladium-catalyzed synthesis of stannanes from corresponding organic halides by 2000 were summarized by Marshall.^457^

After 2005, the search for new efficient stannylation procedures has been reported rarely. Hanson showed high catalytic activity of [PdCl_2_(PhCN)_2_]/PCy_3_ and [Pd_2_dba_3_]/PCy_3_ in the stannylation of 5- and 6-bromoindoles with Sn_2_Bu_6_.^458^ Moderate to high yields of stannylation products in reactions of allyl chlorides,^459^ heteroaryl bromides, or iodides have been described.^460,461^ A variety of variously substituted aryl halides have been stannylated with Sn_2_Bu_6_ at 120 °C in the presence of Pd(OAc)_2_/PCy_3_.^462^

**Scheme 188:**
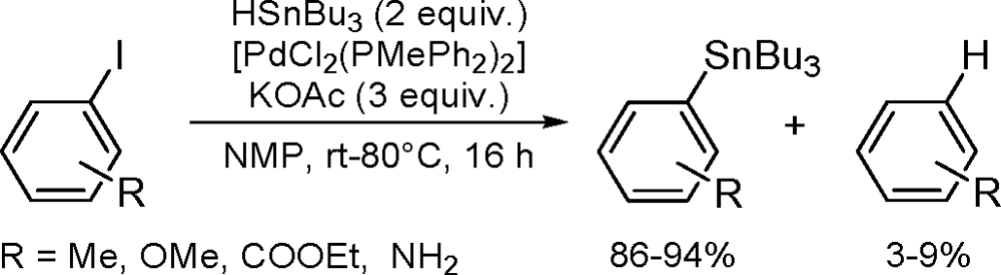
Stannylation of Aryl Halides with Tributylstannane

Masuda has demonstrated that HSnBu_3_ can be used as a stannylating agent (Scheme 188).^463^ In the presence of a catalytic amount of [PdCl_2_(PMePh_2_)_2_] and KOAc, aryl iodides undergo coupling with tributyltin hydride to afford the corresponding arylstannanes.

**Scheme 189:**
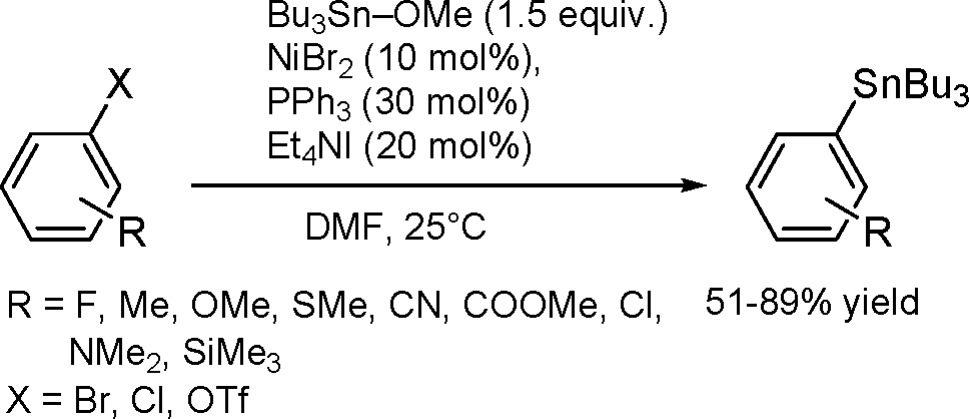
Stannylation of Aryl Halides with Tributylmethoxystannane

It has been established that Sn_2_Bu_6_ is the actual tin source, formed by the dehydrocoupling of HSnBu_3_ under the reaction conditions used. Komeyama and co-workers have reported the nickel-catalyzed stannylation of aryl and alkenyl halides using Bu_3_SnOMe as a stannylating agent to afford aryl- and vinylstannanes, respectively (Scheme 189).^464^

### 4.5 Arsination, Stibination, and Telluration of C–X Bonds

**Scheme 190:**
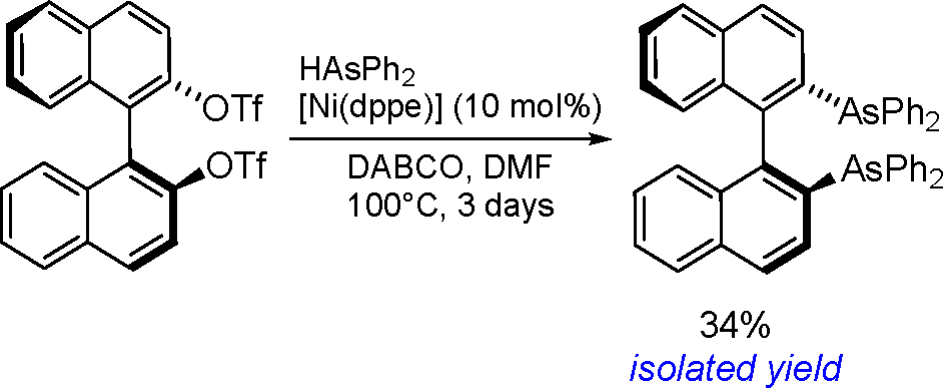
Nickel-Catalyzed Arsination of Aryl Triflates

In 1997, Shibasaki described the synthesis of 2,2′-bis(diphenylarsino)-1,1′-binaphthyl (BINA), the arsine analogue of BINAP (Scheme 190).^465^

**Scheme 191:**
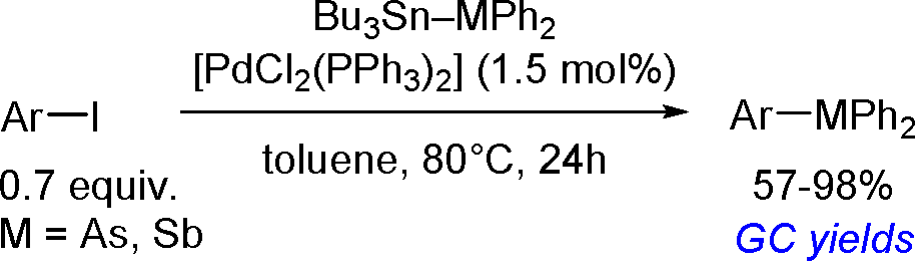
Palladium-Catalyzed Arsination or Stibination of Aryl Iodides

Rossi and Martín have developed a method leading to functionalized triarylarsines and triarylstibines by the palladium-catalyzed coupling of aryl iodides with reagents containing Sn–As or Sn–Sb (Scheme 191) bonds.^466^

The scope of the reaction has been extended to *ortho*-substituted aryl iodides containing a variety of functional groups (Br, Cl, NH_2_, COOMe), perfluoroalkyl iodides^467^ and naphthyl triflates.^468^ The method has been used for the synthesis of a chiral bis(arsine) ligand.^448^ Mechanistic studies by DFT calculations show that the reaction proceeds according to the typical Stille mechanism, involving oxidative addition, transmetalation, and reductive elimination.^469^ The transmetalation step has been found to be rate-limiting. The lack of reactivity of aryl chlorides in the analogous Stille coupling is a consequence of the high energy barrier for the oxidative addition of aryl halides. The syntheses and applications of organostannanes bonded to group 14, 15, and 16 elements have been reviewed.^470^

**Scheme 192:**
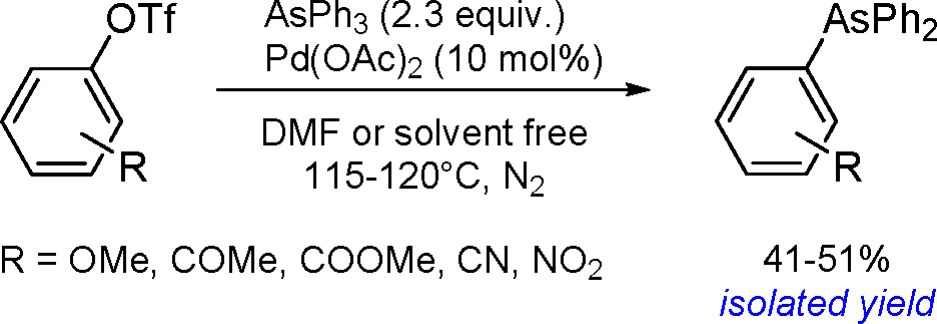
Arsination of Aryl Triflates with AsPh_3_

Another method for the synthesis of aryl arsines uses the palladium-catalyzed arsination of aryl triflates with commercially available, air-stable and inexpensive triphenylarsine as the arsinating agent (Scheme 192).^471^ The reaction is highly functional group-compatible, but it permits only moderate yields of functionalized aryl arsines.

**Scheme 193:**
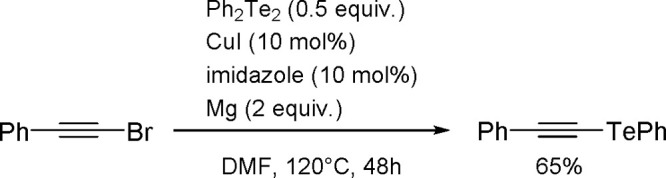
Telluration of Alkynyl Bromides with Ditellurides

Sharma and Braga have reported a single example of the copper-catalyzed synthesis of alkynyltelluride from alkynyl bromides and diaryl ditellurides (Scheme 193).^472^

**Scheme 194:**
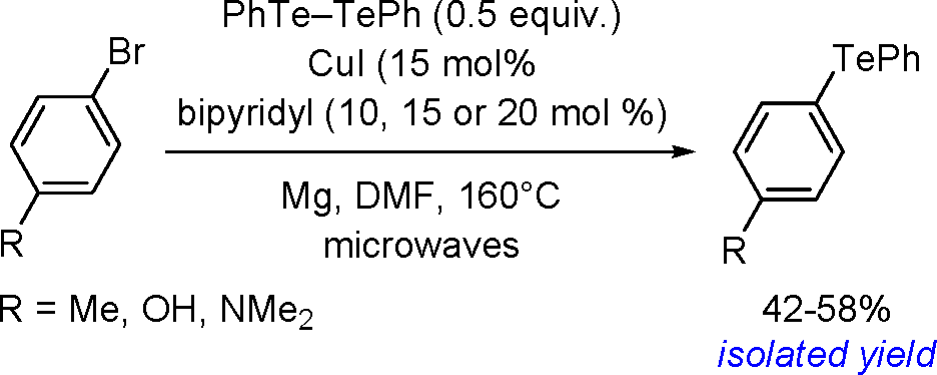
Telluration of Aryl Bromides with Ditellurides

CuI used with bipyridyl has been found to show catalytic activity in the coupling of aryl iodides with diaryl ditellurides (Scheme 194).^473^ A highly efficient reaction has also been observed for 3-bromopyridine and 1-bromonaphthalene.

**Scheme 195:**
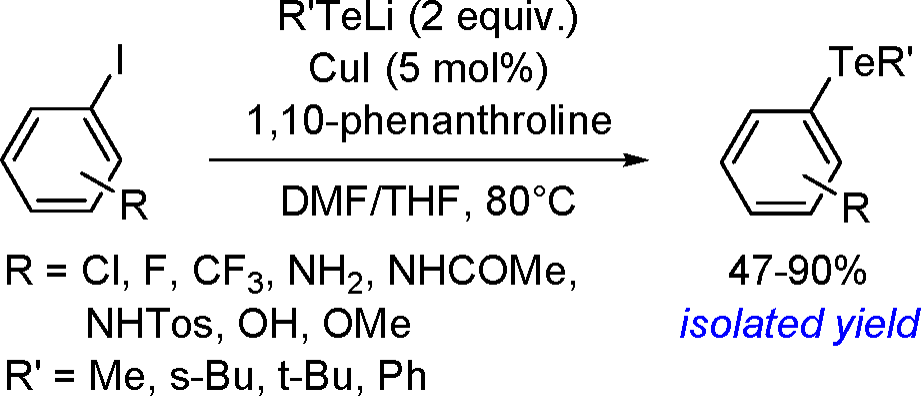
Copper-Catalyzed Telluration of Aryl Bromides with Lithium Tellurides

Synthesis of arylmethyltellurides is possible through the copper-catalyzed coupling of aryl iodides with lithium tellurolates (RTeLi) (Scheme 195).^474^ The tellurating agent is prepared directly before the reaction. The scope of the coupling includes a variety of substituted aryl iodides.

## 5 Dehydrocoupling of Hydrometalloids

While TM-catalyzed carbon–carbon bond-forming reactions are an extremely important part of modern organic synthesis, the catalytic formation of main group element–element bonds represents a comparatively less studied area.^475−477^ In this section, we focus on the dehydrocoupling reactions of hydrometalloids, but we do not discuss the heterodehydrocoupling reactions of hydrometalloids with compounds containing nonmetal–hydrogen bonds. Macromolecular compounds with metalloid–metalloid bonds show a wide range of applications (e.g., in high-performance elastomers, emissive and preceramic materials, thin films’ precursors),^478^ which stimulates the development of their synthesis methods, of the which catalytic dehydrocoupling of hydrometalloids is the most benign and atom-economical process.

Catalytic formation of metalloid–metalloid bonds via the dehydrogenative coupling of hydrometalloids was first reported by Sneddon and Corcoran in 1984 for the coupling of polyhedral boron cages using PtBr_2_ as a catalyst.^479^ The development of methods for coupling boronate esters was much more important as diborane esters are valuable reagents in catalytic borylation and bis-boration processes. Catalytic dehydrocoupling of pinacolborane and catecholborane has been discovered using palladium(II) and platinum(II) complexes with bidentate phosphine ligands as well as heterogeneous systems involving palladium, rhodium, and platinum on alumina, of which the last one seems to be most effective (with TONs of up to 11 600).^480,481^ In addition, the rhodium(I)-mediated homocoupling of amine-borane (H_3_B·NMe_3_), which gives a diborane coordinated to a Rh(I) center,^482^ and the rhodium(I)-catalyzed homocoupling of guanidine bases containing B–H bonds have been reported.^483^ The dehydrocoupling of the bulky H_2_B(dur) aryldihydroborane (dur = 2,3,5,6-tetramethylphenyl) with equimolar amounts of [Pt(PCy_3_)_2_] leads to electron-precise boron networks with bridging diborene and diborane(3) ligands and a platinum complex with both borylene and borane ligands.^484^ More recently, the dehydrocoupling of the electron-poor 3,5-(CF_3_)_2_C_6_H_3_BH_2_ aryldihydroborane mediated by a [(Cp*Ru)_2_(μ-H)_4_] diruthenium–tetrahydrido complex has been reported, which yields an anionic ruthenium complex bearing a tetraarylated chain of four boron atoms.^485^ Longer chains of boron molecules are practically nonexistent. Computational studies of B_*n*_H_*n*+2_ homologues have shown that in the absence of electronic or steric perturbations, the stability of linear B–B chains quickly diminishes relative to cluster structures as *n* increases.^486^

**Scheme 196:**
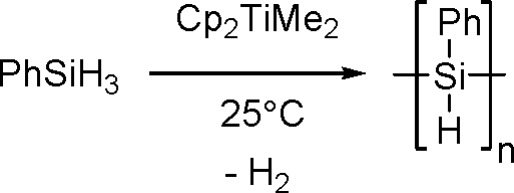
Dehydrocoupling of Primary Silanes in the Presence of Cp_2_TiMe_2_

The dehydrocoupling of organosilanes is an efficient method for producing polysilanes known as polymers with interesting electronic and optical properties resulting from the strong delocalization of σ electrons along the main silicon chain. Development of the dehydrocoupling of primary silanes to produce Si–Si bonds began with the discovery by Harrod and co-workers that the [Cp_2_TiMe_2_] complex initiated the polycondensation of PhSiH_3_ with the evolution of molecular hydrogen to produce phenylsilane oligomers (with DPs of around 10–20) (Scheme 196).^487^

**Scheme 197:**
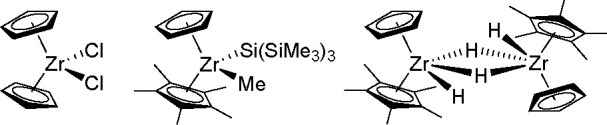
Zirconium Metallocene Catalysts for the Dehydrocoupling Reactions of Silanes

Tilley and Corey as well as Harrod and Tanaka groups developed other active catalytic systems (Scheme 197) leading to polysilanes, mainly group 4 metallocenes of the R_2_MX_2_ type (where M is Zr or Ti, R is Cp or Cp*, and X is usually halide or alkyl) for mechanistic and synthetic studies (e.g., Cp_2_ZrCl_2_/2*n*-BuLi, Cp_2_Zr[Si(SiMe_3_)_3_]Me, [CpCp*ZrH_2_]_2_, Cp_2_ZrMe_2_, Cp*_2_NdCH(SiMe_3_)_2_).^488,489^

The condensation of secondary silanes usually requires more rigorous conditions, and as yet no universal catalyst has been reported to efficiently couple tertiary silanes, with the exception of structurally complex substrates (e.g., Si–H-functionalized dithienophosphole in the presence of Karstedt’s catalyst).^490^

**Scheme 198:**
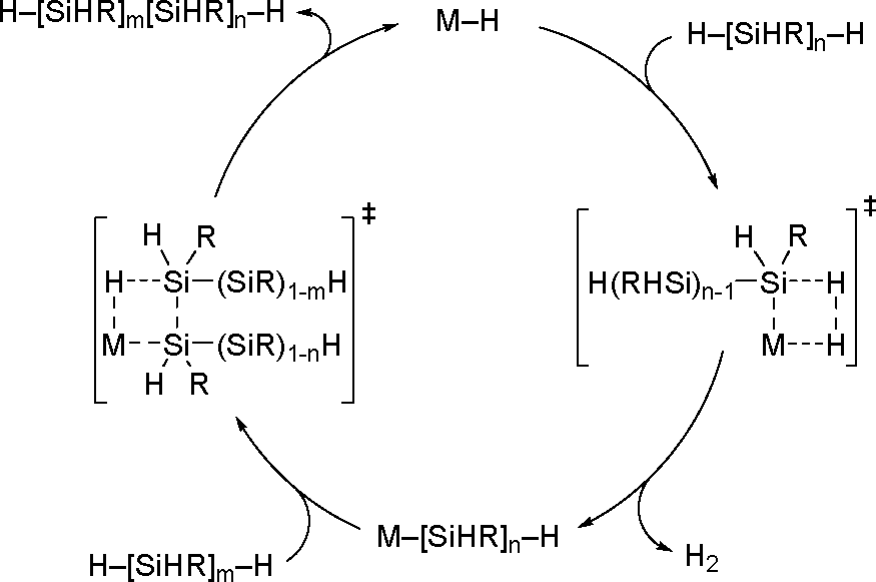
Mechanism of the Early TM-Catalyzed Dehydrocoupling of Silanes

Detailed mechanistic studies of the early TM-catalyzed dehydrocoupling of silanes have revealed σ-bond metathesis via four-membered metallacyclic transition states. In most cases, the active catalyst is considered to be a coordinatively unsaturated metal hydride. The coupling is essentially concerted 2σ + 2σ cycloaddition and passes through two 4-center transition states, resulting in chain elongation by step growth (Scheme 198).^491^

Platinum group metal complexes have also been applied as catalysts in the dehydrocoupling of silanes, with activities comparable to those of early metal complexes; however, these reactions usually show low selectivity and produce a mixture of short-chain linear and cyclic oligomers. However, there are some exceptions of increased selectivity. [Pt(cod)_2_] has been found to be a good catalyst for the dehydropolymerization of secondary aliphatic silanes (with yields of 76–92%) and silafluorene to produce linear polymers.^492^ Rosenberg and co-workers reported Wilkinson’s catalyst used in the dehydrogenative coupling of primary and secondary silanes and confirmed the presence of rhodium–hydride species as active catalysts.^493,494^ For example, Ph_2_SiH_2_ was reported to dehydrocouple at room temperature with a [RhCl(PPh_3_)_3_] catalyst to afford selectively Ph_2_HSiSiHPh_2_ in a yield of 78%. An aminopyridinato–palladium complex successfully catalyzed the polymerization of MeH_2_SiSiH_2_Me toward linear poly(methylsilane).^495^ In most cases, the postulated mechanism involves transition metal–silylene complexes as the key intermediates.

**Scheme 199:**
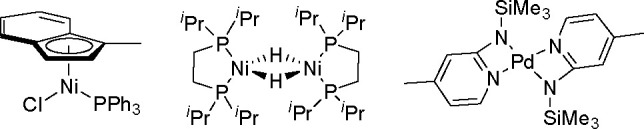
Late TM Catalysts for the Dehydrocoupling of Silanes

The dehydrocoupling of phenylsilane and phenylmethylsilane in the presence of the dimeric hydride-bridged nickel–phosphine complex [(dippe)Ni(μ-H)]_2_ (dippe = 1,2-bis(diisopropylphosphino)-ethane) has been reported, leading to linear oligosilanes (PhSiH)_n_ and (PhSiMe)_*n*_ (*n* = 10–16) (Scheme 199). The proposed catalytically active species is a hydrido–silyl–nickel(II) complex formed by dimer cleavage in a reaction with a silane.^496^ On the other hand, the dehydrogenative polymerization of hexylsilane or phenylsilane catalyzed by the [Ni(dmpe)_2_] complex (dmpe = 1,2-bis(dimethylphosphino)ethane) yielded cyclic silanes as predominant products.^497^ The [Ni(dmpe)_2_] complex has also been applied as a catalyst for the dehydrogenative polymerization of silafluorenes.^498^ The nickel(0) complex [Ni_2_(iPr_2_Im)_4_(cod)] (iPr_2_Im - 1,3-(diisopropyl)imidazol-2-ylidene) has been found an effective catalyst for the acceptorless dehydrogenative coupling of Ph_2_SiH_2_ to the corresponding disilane Ph_2_HSi–SiHPh_2_ and trisilane Ph_2_HSi–Si(Ph)_2_–SiHPh_2_, whereas the coupling of PhSiH_3_ gives a mixture of cyclic and linear oligomers (with 5 < *n* < 17). Nickel silyl complexes, that is, *cis*-[Ni(iPr_2_Im)_2_(SiHPh_2_)_2_] and [{(iPr_2_Im)Ni(μ^2^-(HSiPh_2_)}_2_], have been identified in the resulting mixture and they are likely to be the intermediates in the catalytic cycle.^499^

Another application of a nickel-based precatalyst for phenylsilane dehydropolymerization involves an indenyl–phosphine–nickel complex, [(1-MeInd)Ni(PPh_3_)Cl], known as an alkene polymerization catalyst.^500^ Generally, the mechanism of late TM-catalyzed dehydrogenative polymerization has not yet been fully clarified, and three possible options for Si–Si bond formation are postulated: via repetitive σ-bond metathesis, via disproportionation through a metal–silylene complex, or via oxidative addition/reductive elimination.

**Scheme 200:**
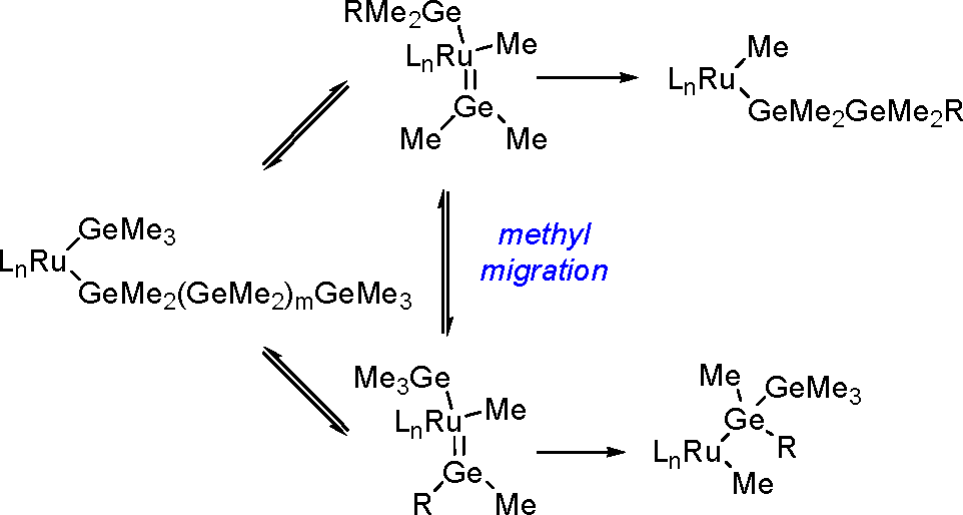
Mechanism of the Demethanative Coupling of HGeMe_3_

The activation of third- and fourth-row main group E–H bonds (E = Ge, As, Sn, Sb, Te) has been less studied since the resulting E–E bonds have generally reduced stability relative to that of the lighter *p*-block elements. Harrod and co-workers have reported that phenylgermane PhGeH_3_ reacts in the presence of [Cp_2_TiMe_2_] catalysts by analogy with phenylsilane to afford complex germanium-based polymers as a result of the further intermolecular dehydrocoupling and cross-linking of (PhGeH)_n_ oligomers.^501^ Similar results have been achieved by Tanaka and Choi using [Cp_2_ZrCl_2_] or [Cp*CpZrCl_2_] complexes activated by *n*-BuLi.^502^ On the other hand, the [Ru(PMe_3_)_4_(GeMe_3_)_2_]-catalyzed coupling of tertiary germane HGeMe_3_ led to highly branched polygermanes, as reported by Berry and co-workers^503^ This unprecedented demethanative coupling proceeded through sequential α-methyl- and germyl-to-germylene migration steps to form ruthenium–germylene intermediates (Scheme 200).

**Scheme 201:**
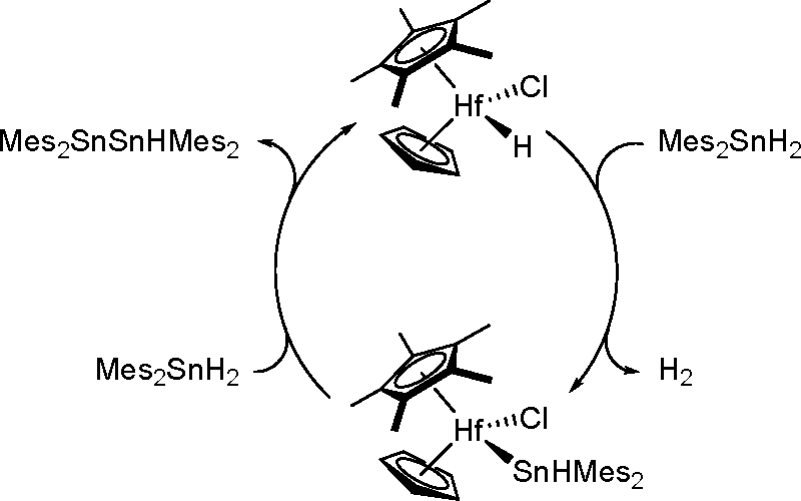
Mechanism of the Dehydrocoupling of Diarylstannanes in the Presence of a Hafnium Catalyst

The dehydrocoupling of dialkylstannanes proceeds with catalysts based on zirconium, lanthanides, rhodium, or palladium.^504^ Tilley and co-workers reported the catalytic dehydrocoupling of secondary stannanes mediated by zirconocenes (e.g., Me_2_C(C_5_H_4_)_2_Zr[Si(SiMe_3_)_3_]Me) to yield a mixture of high-molecular weight polystannanes and cyclic oligomers.^505^ The dehydrogenative dimerization of Mes_2_SnH_2_ (Mes = mesityl) has been successfully performed with the [CpCp*Hf(H)Cl] complex.^506^ Isolation of the hafnium–hydrostannyl complex [CpCp*Hf(SnHMes_2_)Cl] as an intermediate supported the concept of the key role of inorganometallic species in catalysis (Scheme 201). The dehydrocoupling appears to occur by elimination of a SnMes_2_ molecule from [CpCp*Hf(SnHMes_2_)Cl], with Sn–Sn bond formation proceeding via stannylene insertion into the Sn–H bond.

**Scheme 202:**
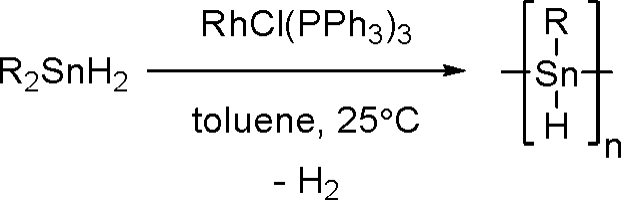
Dehydrocoupling of Dialkylstannanes in the Presence of Wilkinson’s Catalyst

Wilkinson’s catalyst has been reported to yield linear polystannanes using secondary stannanes such as *n*-Bu_2_SnH_2_ as substrates at room temperature (Scheme 202),^507,508^ whereas the dichlorobis(triphenylphosphine)palladium(II) complex promoted the formation of cyclic oligomers.^509^

**Scheme 203:**
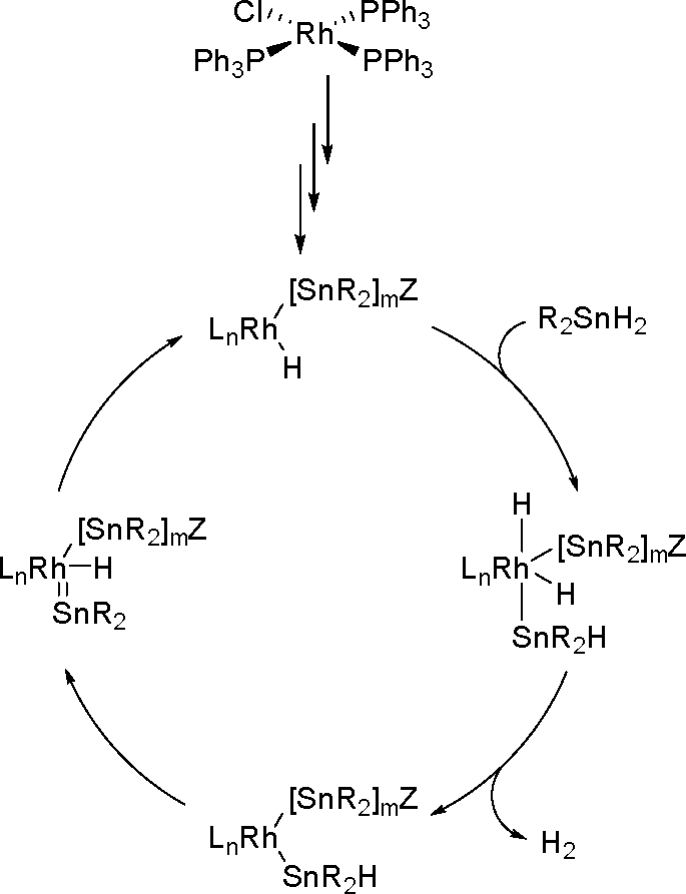
Mechanism of Dialkylstannane Dehydrocoupling in the Presence of Wilkinson’s Catalyst

The proposed mechanism for the late TM-catalyzed dehydrocoupling of secondary stannanes involves an oxidative-addition and reductive-elimination sequence followed by α-hydrogen elimination to furnish a stannylene ligand before the subsequent insertion into a growing stannane chain (Scheme 203).

**Scheme 204:**
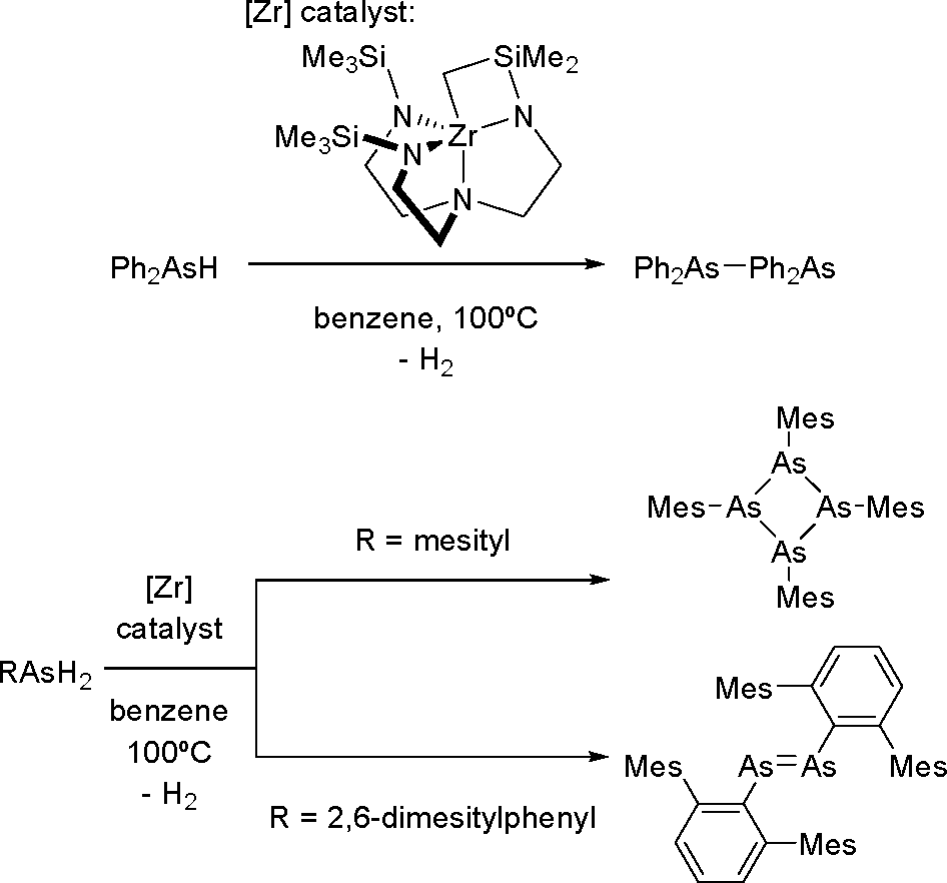
Dehydrocoupling of Hydroarsines in the Presence of a Zirconium Catalyst

Dehydrocoupling of secondary arsine leads to the formation of dimeric species with As–As bonds in the presence of triamidoamine-supported zirconium complexes, (N_3_N)ZrX (where N_3_N is N(CH_2_CH_2_NSiMe_3_)_3_^3–^ and X is a monoanionic ligand). Mechanistic studies show that As–As bond formation proceeds via σ-bond metathesis steps similarly to the previously reported dehydrocoupling of phosphines by the same catalysts. The reaction of primary arsines bearing bulky ligands (MesAsH_2_ or dmpAsH_2_ (dmp = 2,6-dimesitylphenyl)) under similar conditions resulted in the formation of a *trans*-bent diarsene dimer (dmp)As = As(dmp) (through the elimination of the arsinidene molecule (dmpAs:) from the arsenido–zirconium intermediate (N_3_N)ZrAsH(dmp)) or the cyclic Mes_4_As_4_ tetramer, depending on the level of steric crowding caused by the ligand (Scheme 204).^510^

**Scheme 205:**
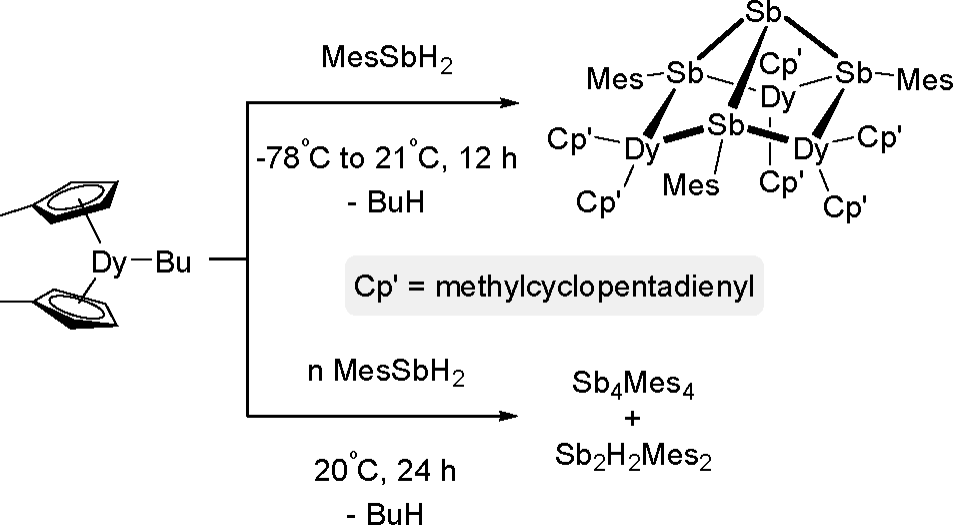
*Cross*-Dehydrocoupling of a Dysprosium Metallocene Complex with MesSbH_2_

Similar reactivity has been observed for stibine dehydrocoupling. The reaction of the MesSbH_2_ primary stibine with group 4 metallocene catalysts (e.g., [Cp_2_Zr(H)Cl], [CpCp*Hf(H)Cl]) proceeds effectively to generate the Sb_4_Mes_4_ cyclic tetrastibetane.^511^ It has been confirmed that the reaction occurs through α-hydrogen migration with stibinidene (:SbR) elimination from the [CpCp*HfCl{Sb(H)Mes}] intermediate, formed in a stoichiometric reaction between [CpCp*Hf(H)Cl] and MesSbH_2_. MesSbH_2_ dehydrocoupling activated by *n*-BuLi in the presence of the [Cp’_3_Y] (Cp’ = methylcyclopentadienyl) yttrium metalocene complex occurs to give a 1,2-distibane (Sb_2_H_2_Mes_2_) and tetrastibetane (Sb_4_Mes_4_). [Cp’_3_Dy] used as a precatalyst furnished a more complex product of the *cross*-dehydrocoupling of the former with MesSbH_2_.^512^ Such dysprosium compounds that contain the unusual [Sb_4_Mes_3_]^3–^ Zintl-like ligand (e.g., [(η^5^-Cp′_2_Dy)_3_{μ-(SbMes)_3_Sb}]) have been found as promising single-molecule magnets (Scheme 205).

**Scheme 206:**
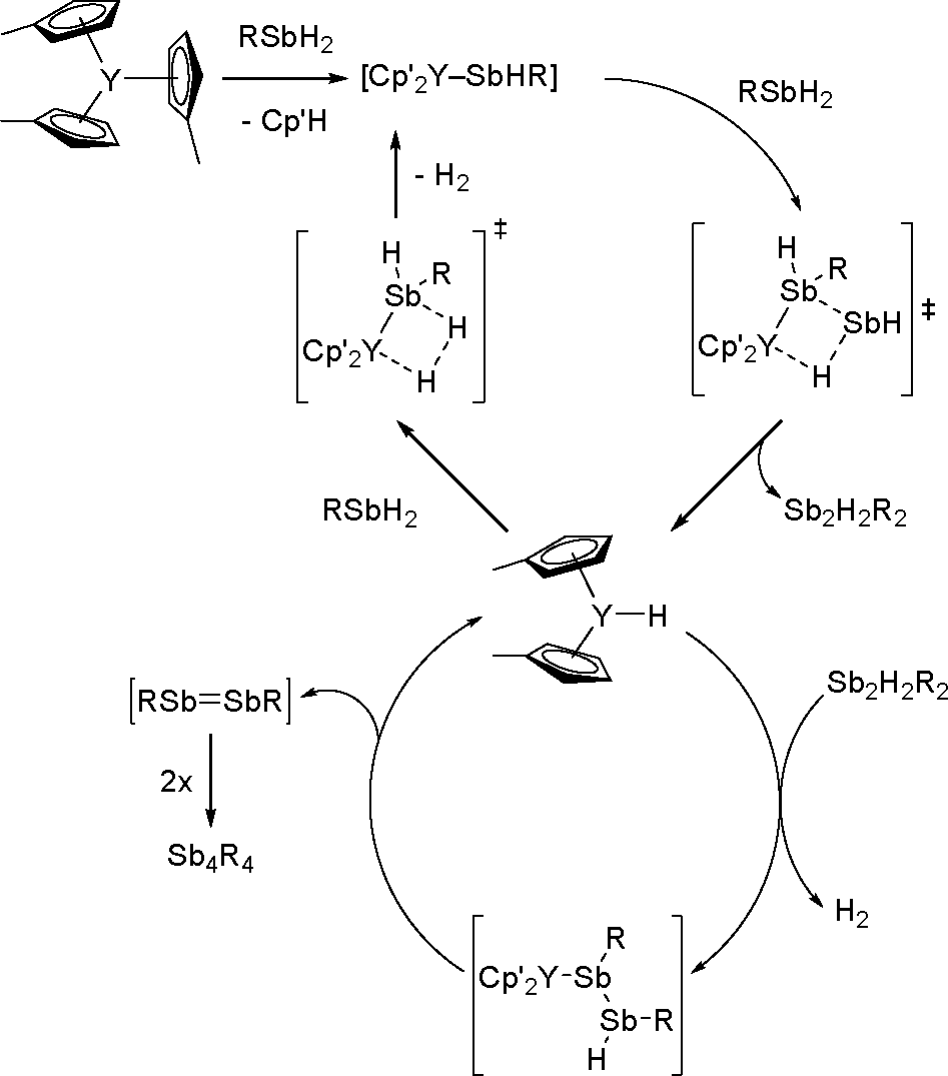
Dehydrocoupling of Stibines in the Presence of an Yttrium Catalyst

The [Cp′_2_YSb(H)Mes] stibinide complex has been proposed as an active catalyst, but the formation of Sb_2_H_2_Mes_2_ suggests that the dehydrocoupling does not occur via stibinidene (MesSb:) elimination. In the case of yttrium catalysis, the reaction mechanism involves the formation of a cyclic, four-membered transition state in which a distibine (Sb_2_H_2_Mes_2_) and a [Cp′_2_YH] yttrium–hydride intermediate are generated (Scheme 206).^512^ To explain the formation of Sb_4_Mes_4_ from Sb_2_H_2_Mes_2_, the formation of the MesSb = SbMes distibene via β-hydride elimination of the distibinide intermediate, [Cp′_2_Y{RSb–Sb(H)R}], has been proposed.

Heterodehydrocoupling reactions require that bonds between different metalloids are formed selectively over competitive homodehydrocoupling, so these are generally more challenging processes. It is worth emphasizing that among this group of processes, the dehydrocoupling of hydroboranes or hydrosilanes with P–H and N–H bonds is of practical importance, and it has been described in other reviews.^513−515^

**Scheme 207:**
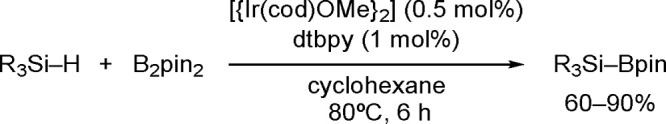
Iridium-Catalyzed *Cross*-Dehydrocoupling of Hydrosilanes with Diboranes

The synthesis of silylboranes through the catalytic borylation of Si–H bonds has been reported by Boebel and Hartwig.^516^ The reaction of trialkylhydrosilanes with B_2_pin_2_ catalyzed by the combination of [Ir(OMe)cod]_2_ and 4,4′-di-*tert*-butylbipyridine (dtbpy) leads to the formation of trialkylsilyl(pinacolato)boranes in good yields (Scheme 207).

**Scheme 208:**
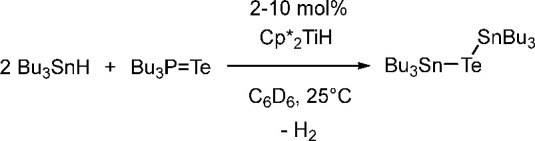
Preparation of Sn–Te Bonds by Heterodehydrocoupling

A titanocene catalyst (Cp*_2_TiH) has been used for the preparation of Sn–Te bonds by heterodehydrocoupling (Scheme 208).^517^ The reaction of tributylphosphine telluride (Te = PBu_3_) with tributylstannane led to Bu_3_Sn–Te–SnBu_3_, a potential single-source precursor for the synthesis of tin telluride (SnTe), a small band gap semiconductor.

**Scheme 209:**
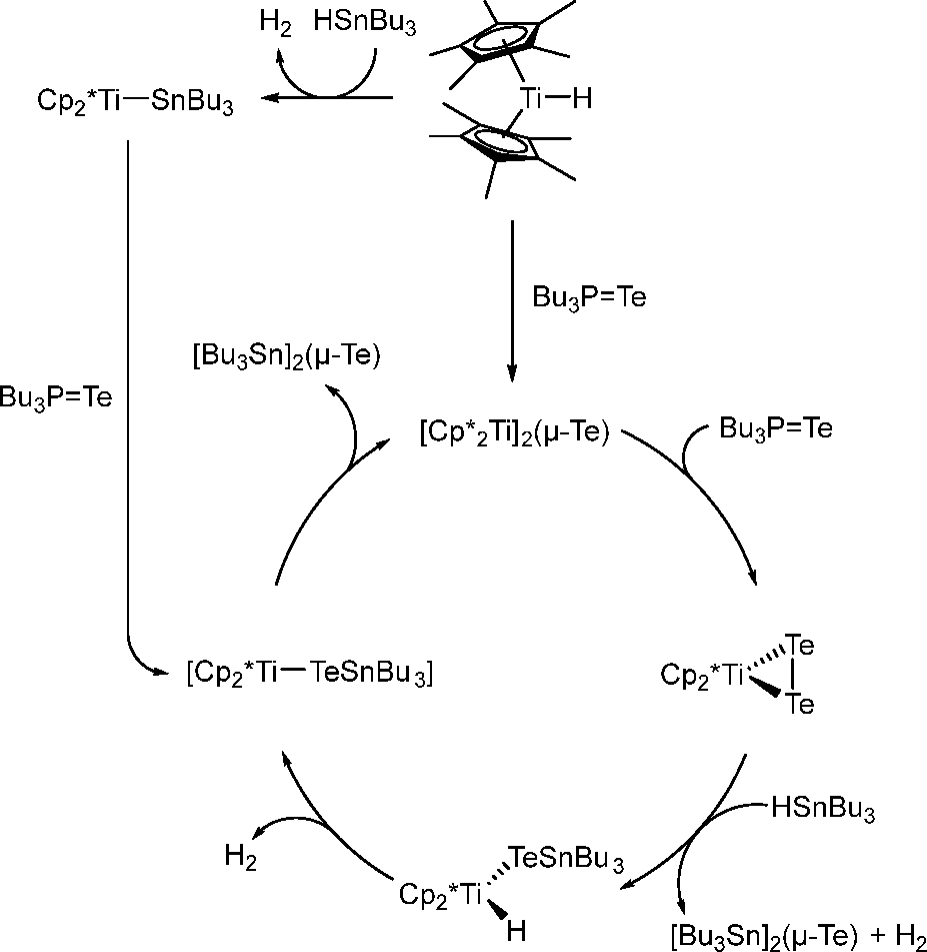
Mechanism of the Dehydrocoupling of Tributylphosphine Telluride with Tributylstannane

This reaction is postulated to proceed via [(Cp*_2_Ti)_2_(μ-Te)] and [Cp*_2_Ti(H)TeSnBu_3_] as intermediates (Scheme 209).

## 6 Activation (and Functionalization) of C–H Bonds with Vinylmetalloids

**Scheme 210:**
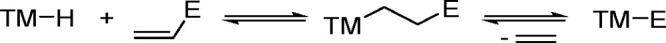
Formation of TM–E bonds via Sequential Insertion and β-E-Elimination

In the systems that contain hydride complexes or enable their generation in situ, vinylmetalloids can be used for TM-E bond formation (Scheme 210).

### 6.1 Borylation of C–H Bonds

**Scheme 211:**
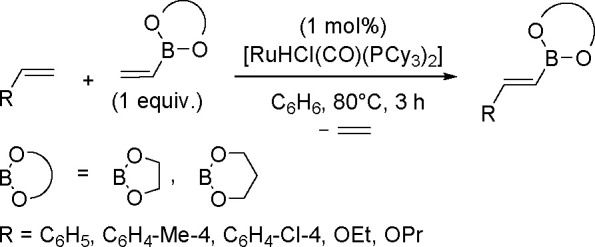
Ruthenium-Catalyzed Deethenative Borylation of Olefins

Vinyl-substituted boronates (i.e., vinyldioxaborolane and vinyldioxaborinane) have been reported to react with olefins in the presence of [RuHCl(CO)(PCy_3_)_2_] with formation of functionalized vinylboronic derivatives and evolution of ethene (Scheme 211).^518^

**Scheme 212:**
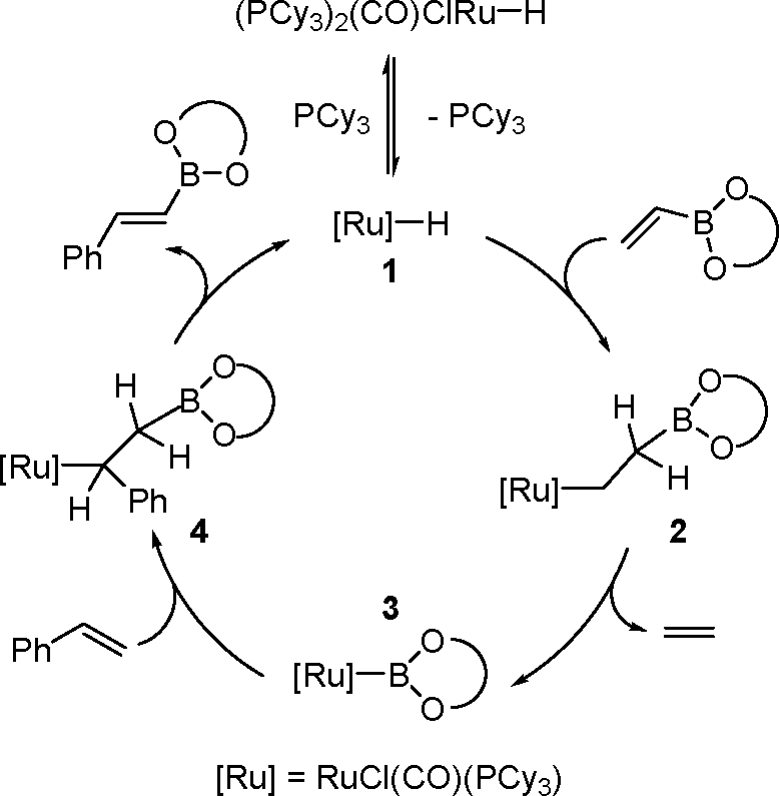
Mechanism for the Ru-Catalyzed Deethenative Borylation of Olefinic C–H Bonds^506^

Deethenative coupling achieves yields of 80%–95% and full E-stereoselectivity in the borylation of styrenes and vinyl ethers. Investigation of the reaction between the boryl complex [Ru(Bcat)Cl(CO)(PCy_3_)_2_] and styrene suggested a mechanism of the reaction (Scheme 212). It assumes that the active catalyst species is a monophosphine–chlorohydridocarbonyl–ruthenium complex (**1**). In the first step of the reaction, vinylboronate associates to complex **1** and undergoes migratory insertion into the Ru–H bond to produce **2**. Subsequently, a boryl complex (**3**) is formed, and ethylene is eliminated via β-boryl elimination. Styrene then undergoes association and migratory insertion into the ruthenium–boron bond to form **3**. Finally, vinylboronate is formed, and complex **1** is regenerated via β-hydrogen elimination.^518^

This mechanism was confirmed by DFT studies of the β-boryl elimination process.^519^ The studies conducted for a simplified model layout showed reversibility of deethenative borylations and relatively low energetic barriers for these processes. A relatively small effect of the phosphine nucleophilic character on the energy profile of the process was shown.

### 6.2 Silylation and Germylation of C–H Bonds

**Scheme 213:**
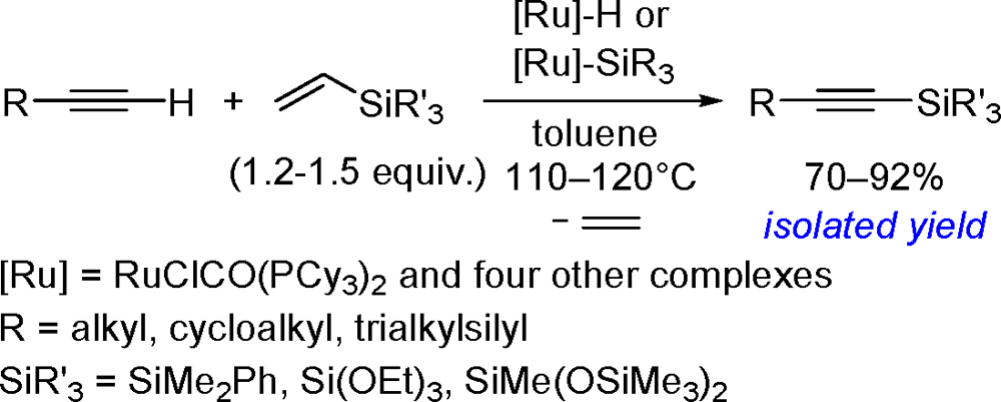
Ruthenium-Catalyzed Deethenative Silylation of Acetylenes

In 2006, the Marciniec group reported a new catalytic method for the synthesis of silylethynes with vinylsilanes as silylating agents (Scheme 213).^520^ This deethenative silylation of acetylenes was shown to occur in the presence of ruthenium complexes containing [Ru]–H or [Ru]–Si bonds, such as [RuHCl(CO)(PCy_3_)_2_], [RuHCl(CO)(P*i*-Pr_3_)_2_], [RuH(CO)(MeCN)_2_(PCy_3_)_2_][BF_4_], and [Ru(SiMe_3_)Cl(CO)(PPh_3_)_2_], with evolution of ethene and selective formation of silyl-substituted acetylenes with yields in the range of 70%–100%. The reaction is efficient in the presence of a series of vinylsilanes containing a variety of silyl groups, such as SiMe_2_Ph, SiMe_2_(OSiMe_3_), SiMe(OSiMe_3_)_2_, and Si(OEt)_3_, as well as vinylsiloxanes and vinylsilazanes.

**Scheme 214:**
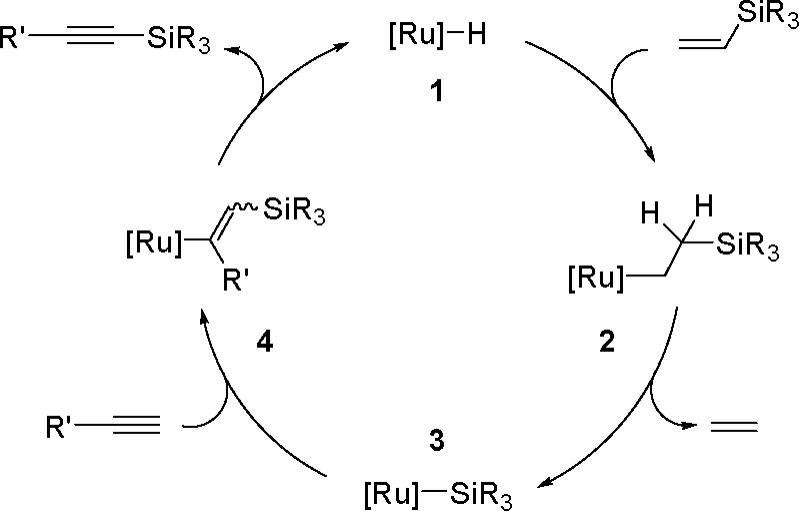
Mechanism of the Ru-Catalyzed Deethenative Silylation of C(sp)–H Bonds

On the basis of an analysis of the literature data on the formation of silyl complexes from ruthenium hydrides^521,522^ and studies of the stoichiometric reaction of silyl complexes with phenylacetylene, a reaction mechanism has been proposed. The mechanism involves vinylsilane insertion into the ruthenium–hydrogen bond followed by β-H elimination to form a ruthenium–silyl complex (**3**). The subsequent step is proposed to be the migratory insertion of terminal acetylene into the ruthenium–silicon bond (Scheme 214). However, the details of the isomerization of complex **4**, necessary for the postulated β-H elimination, require further investigation.^520^

**Scheme 215:**
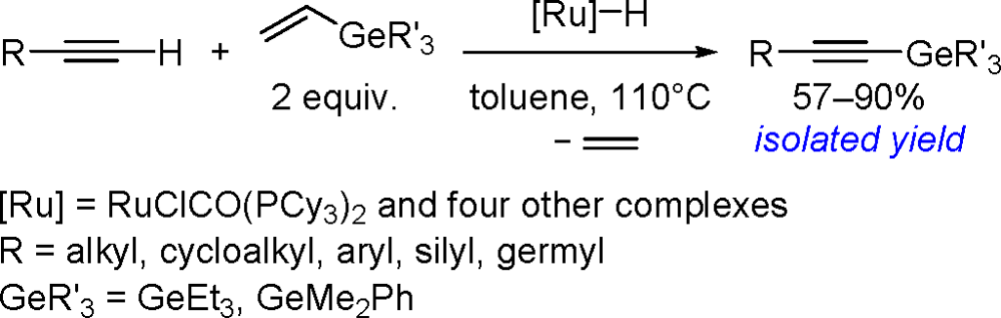
Ruthenium-Catalyzed Deethenative Germylation of Acetylenes

In subsequent papers, the Marciniec group showed that the reaction can be used for the effective synthesis of germylacetylenes (Scheme 215).^523^

Through the successful isolation of a germyl–ruthenium complex and the investigation of its stoichiometric reactions with acetylenes a mechanism has been proposed, analogous to that presented in Scheme 214.^523,524^

**Scheme 216:**
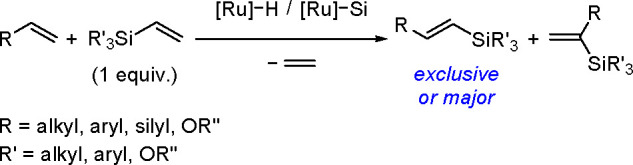
Deethenative Silylation of Olefins

The deethenative silylation of olefins has first been described by the Marciniec^525^ and Seki^526^ groups in the study of hydrosilylation processes in the presence of ruthenium complexes (Scheme 216).

The reaction has been addressed in several reviews.^527−529^ The process occurs in the presence of ruthenium, rhodium, cobalt, and iridium complexes. However, the highest yields have been reported for ruthenium-based catalysts, in particular [RuHCl(CO)(PCy_3_)_2_]. The most important application examples for organic synthesis have been described in ref (530). The catalytic system used tolerates many functional groups, including F, Cl, Br, OR, silyl, thiophenyl, ferrocenyl, carbazolyl, borolanyl, and borinanyl. In the reaction with vinylaromatic compounds, E isomers form exclusively. The applications of the process in synthesis are illustrated by the silylation of dienes,^531^*N*-vinylcarbazole,^532^ vinylferrocene,^533^ and vinyl ethers.^534^ An attractive possibility of using the reaction in the modification of vinylsilsesquioxanes has been demonstrated.^535^

**Scheme 217:**
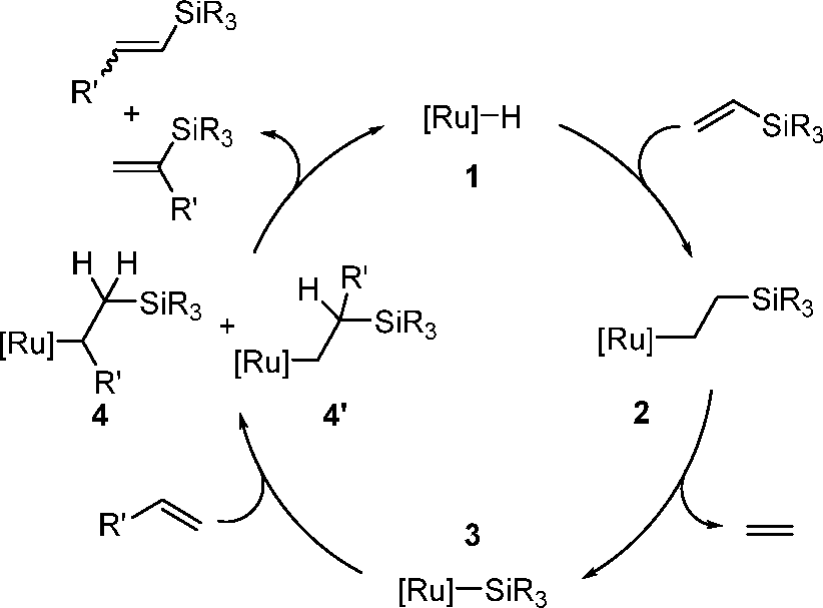
Mechanism of the Ru-Catalyzed Deethenative Silylation of Olefinic C(sp^2^)–H Bonds

The mechanism of deethenative coupling in the presence of ruthenium complexes has been proposed by Wakatsuki^521^ and Marciniec^536^ groups on the basis of studies on the reactivity of ruthenium–hydride complexes with vinylsilanes^521^ and silyl complexes with styrenes.^522,536^ In addition, studies were performed with deuterium-labeled reagents.^522^ According to the mechanism (Scheme 217), hydride and/or silyl complexes are the active catalysts of the reaction.

How easily a silyl complex forms through the sequence of vinylsilane migratory insertion into the Ru–H bond and β–SiR_3_ elimination, leading to a silyl complex and ethane, is of key importance for the silylation process with vinylsilanes. Removal of ethene from the reaction medium allows the process to run almost quantitatively. In the next step, the olefin undergoes insertion into the Ru–Si bond. Subsequent β-H elimination gives ruthenium hydride and a silylated olefin. Selectivity depends on the nature of the R group in the olefin and the substituents at silicon. The silylation of styrenes and vinylarenes leads almost exclusively to E-isomer formation.

**Scheme 218:**
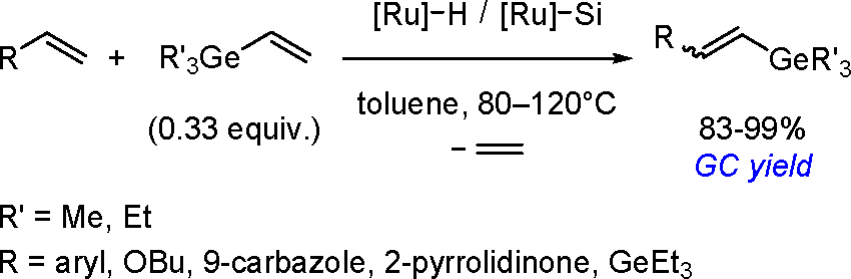
Deethenative Germylation of Olefins

Further studies on the reactivity of vinylmetalloids performed by Marciniec group revealed a similar transformation of vinylgermanes (Scheme 218),^524^ and a new method of olefin germylation was proposed.

**Scheme 219:**
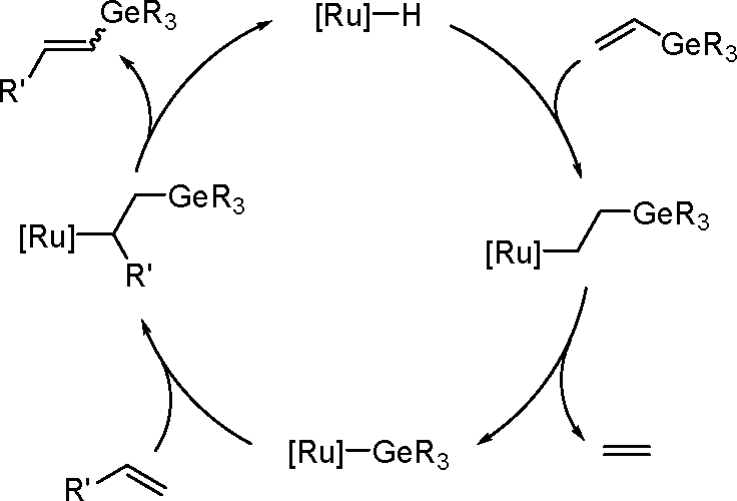
Mechanism of the Ru-Catalyzed Deethenative Germylation of Olefinic C(sp^2^)–H Bonds

The germylation of styrenes, 9-vinylcarbazole, 1-vinylpyrrolidin-2-one, and vinyltriethylgermane with H_2_C=CHGeEt_3_ runs stereoselectively, with the exclusive formation of E-isomers. A mechanism of the process has been proposed on the basis of studies on the reaction of the ruthenium–hydride complex with vinylgermane, isolation and characterization of the ruthenium–germyl complex, and the determination of its reactivity with olefins (Scheme 219). [RuCl(CO)(GeMe_3_)(PPh_3_)_2_] has been proved to be catalytically competent in the reaction. The proposed mechanistic scheme, by analogy with the previous schemes, involves sequential insertion of vinylgermane into the ruthenium–hydride complex followed by β-Ge-elimination and olefin insertion into the ruthenium–germyl complex followed by β-H-elimination.

**Scheme 220:**
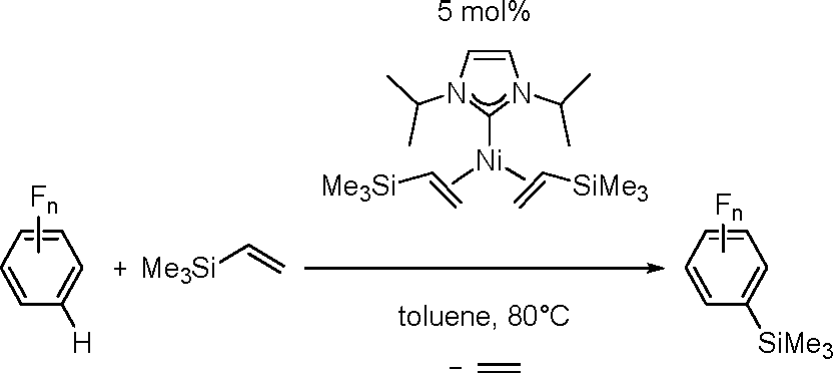
Nickel-Catalyzed Deethenative Silylation of Fluoroarenes

The first example of C–H bond silylation in aromatic compounds with vinylsilanes was provided in 2000 by the Murai group who reported the catalytic activity of [Ru_3_(CO)_12_] in the silylation of heteroaryls.^311^ In 2017, similar deethenative silylation of the aromatic C–H bond was described in the presence of a nickel complex, [Ni(NHC)(η^2^-H_2_C=CHSiMe_3_)_2_] (where NHC = 1,3-di(iso-propyl)imidazol-2-ylidene) (Scheme 220).^537^

Similar transformations were observed for vinylgermanes.^538^

### 6.3 Stannylation of C–H Bonds

**Scheme 221:**
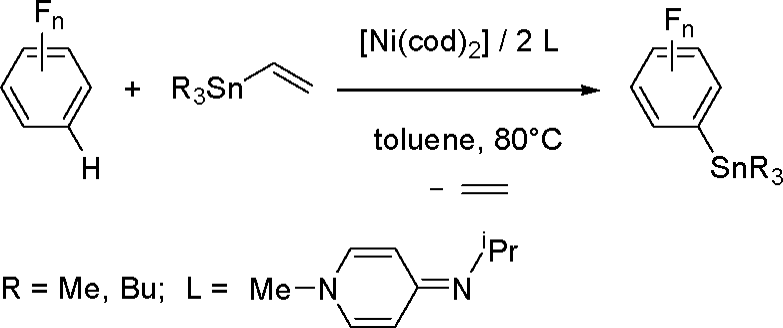
Nickel-Catalyzed Deethenative Stannylation of Fluoroarenes

Stannylation of the fluoroarene C–H bond with vinylstannane was reported by Johnson and co-workers (Scheme 221).^539^

**Scheme 222:**
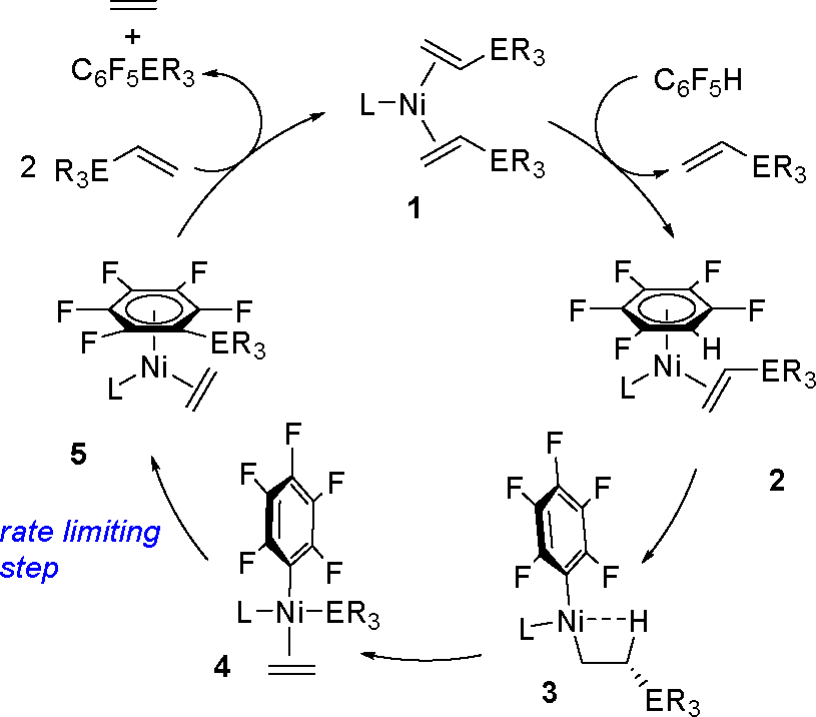
Mechanism of the Ni-Catalyzed Deethenative Metallation of Aryl C(sp^2^)–H Bonds^538^

The scope of arene C–H activation and functionalization with vinylmetalloids is limited to fluorinated aromatics and the trimethylsilyl group. The deethenative stannylation reaction activates one, two, or three C–H bonds in the ring. In the optimum conditions, the yields of stannylated arenes were 90%–99% in most cases.^539^ Silylation is the most difficult Ni-catalyzed arene metalation process with vinylmetalloids, but the proper choice of the catalyst and high temperature of the process permit high yields of arylsilanes. On the basis of kinetic studies and DFT computations, a mechanism of the deethenative metalation of arenes has been proposed (Scheme 222).^538^

For all vinylmetalloids, the mechanism involves a similar sequence of steps starting with the oxidative addition of the C–H bond to the π-olefin complex (**2**), leading to a σ-alkyl stabilized by the interaction of hydrogen at position β with the metal center (**3**). As a result of β-ER_3_ elimination, complex **4** is formed and undergoes reductive elimination of the carbon–metalloid bond. The cycle is closed by the dissociation of ethylene and the association of two vinylmetalloid molecules. The reductive elimination is the rate-limiting step. The energies of the key intermediates and the transition states for the key reaction steps differ for derivatives of different metalloids. Particularly for vinylsilanes, high energies of the transition states are observed, corresponding to β-SiMe_3_ elimination and reductive elimination of arylsilane. Complex **3**, in the presence of silicon as a metalloid and ligand L of high steric hindrance (e.g., IMes), may undergo a competing reductive elimination of the C–C bond and formation of a hydroarylation product. The choice of ligand L of lower steric hindrance (e.g., 1,3-diisopropylimidazol-2-ylidene) permits selective silylation.^538^

### 6.4 Functionalization of O–H and N–H Bonds with Vinylmetalloids

**Scheme 223:**
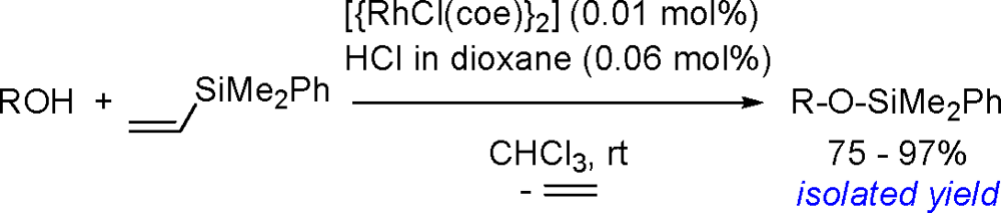
Rhodium-Catalyzed Deethenative Silylation of Alcohols and Phenols

Catalytic alcohol silylation methods involve hydrosilylation of the C=O bond, dehydrocoupling of alcohols with the H–Si bond, and a relatively new silylation with vinylsilanes. The first silylation of alcohols (and phenols) with vinylsilane in the presence of Wilkinson’s catalyst ([RhCl(PPh_3_)_3_]) has been reported by Jun.^540^ The scope of the reaction includes efficient silylation of phenol, pyrocatechol, benzyl alcohol, alkyl alcohols, diol, and triol with vinyltrimethylsilane. Corresponding silyl ethers were formed with high isolation yields (up to 97%). In a subsequent paper, Jun presented optimized reaction conditions and several examples illustrating the reactivities of primary, secondary, and tertiary alcohols (Scheme 223).^541^

In the conditions tested, no catalytic activity of ruthenium and platinum complexes was observed. The system ([{IrCl(coe)_2_}_2_]/HCl) enabled the catalyst loading decreased to 0.5 mol % and reaching a high TON of 5000 for the test reaction. Preliminary mechanistic studies indicate that in the system employed and in the presence of HCl, chlorosilane formation occurs, which then reacts with the alcohol.

**Scheme 224:**
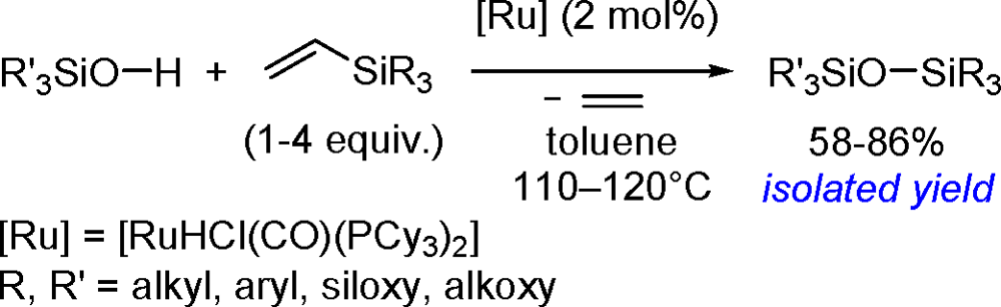
Ruthenium-Catalyzed Deethenative Silylation of Silanols

The Marciniec group has extended the scope of the reaction to silanols (R_3_SiOH) and demonstrated the catalytic activity of the ruthenium–hydride complex ([RuHCl(CO)(PCy_3_)_2_]) (Scheme 224).^542^ The reported reactions proceeded in good yields (up to 99%). In the procedure, as well as the siloxane, being the synthetic target, undesirable 1,2-bis(silyl)ethenes formed. Only with alkoxy-substituted vinylsilanes did the reaction permit selective formation of corresponding siloxanes.

**Scheme 225:**
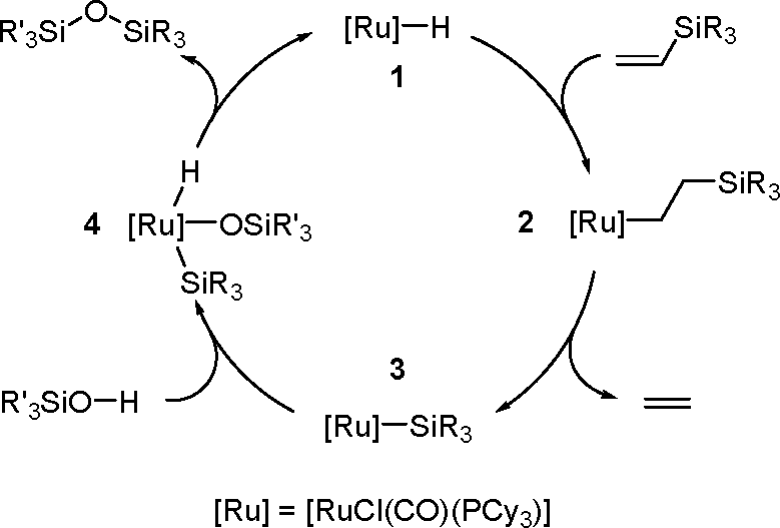
Mechanism of the Ru-Catalyzed Deethenative Silylation of Silanols

On the basis of literature data^521^ and studies of stoichiometric reactions, a mechanism of the process has been proposed (Scheme 225). It assumes vinylsilane insertion into the Ru–H bond and subsequent β-SiR_3_ elimination resulting in the formation of a ruthenium–silyl complex (**3**). Subsequently, oxidative addition leads to a ruthenium complex (**4**), followed by reductive elimination of a siloxane. The second part of the mechanism (**3** → **4** → **1**), supported by the investigation of stoichiometric reactions, needs further experimental and computational verification.

**Scheme 226:**
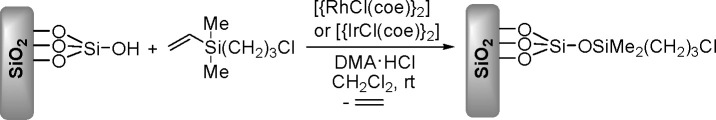
Immobilization of a Silica Surface via Catalytic Deethenative Silylation

The reaction has been used for the transition metal-catalyzed immobilization of organic functional groups onto solid supports. Jun has reported immobilization of functionalized vinylsilane onto a silica and glass surface (Scheme 226).^543^

Modification of a silica surface with vinylsilanes, using the catalytic activity of ruthenium complexes, has been proposed by Hreczycho et al.^544^

**Scheme 227:**
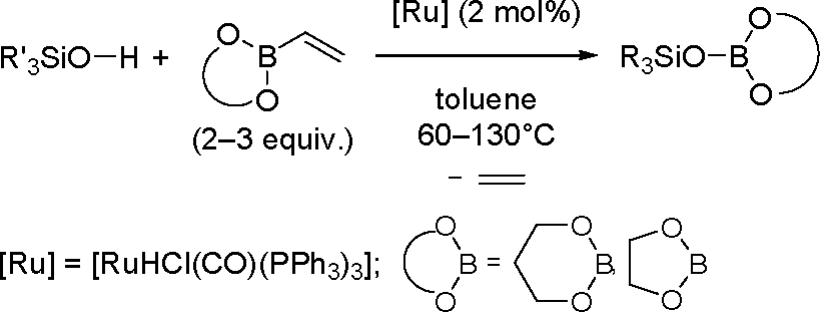
Ruthenium-Catalyzed Deethenative Borylation of Silanols

**Scheme 228:**
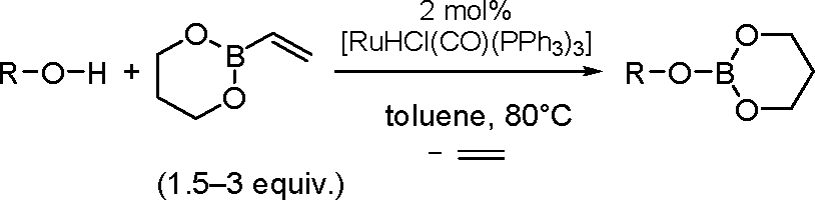
Ruthenium-Catalyzed Deethenative Borylation of Alcohols

Hydroxyl group functionalization with other vinylmetalloids was proposed by Marciniec. Both silanols^545^ and alcohols^546^ were demonstrated to react with vinylboronates to form borasiloxanes and boryl ethers, respectively (Schemes 227 and 228). Both reactions were accompanied by the competing deethenative silylation of vinylboronates; however, the less active [RuHCl(CO)(PPh_3_)_3_] ensured reaction selectivity. The scope of the reaction involves primary alkyl-, aryl-, siloxyl- and alkoxy-substituted silanols as well as primary alcohols including benzyl alcohol. The boron derivatives used were 2-vinyl-1,3,2-dioxaborinane and 2-vinyl-1,3,2-dioxaborolane.

In the presence of [Ru_3_(CO)_12_], 2-vinyldioxaborinane, 2-vinyldioxaborolane, and vinylboronic acid pinacol ester were transformed nearly quantitatively into corresponding borasiloxanes.^547^ A mechanism of the process was proposed on the basis of NMR monitoring of the [Ru_3_(CO)_12_] reaction with triethylsilanol and vinylboronate to include oxidative addition of silanol to ruthenium, insertion of vinylborate into the Ru–H bond, formation of a silyl complex as a result of β-SiEt_3_-elimination, and product release in reductive elimination. However, the mechanism proposed needs thorough corroboration by further studies.

**Scheme 229:**
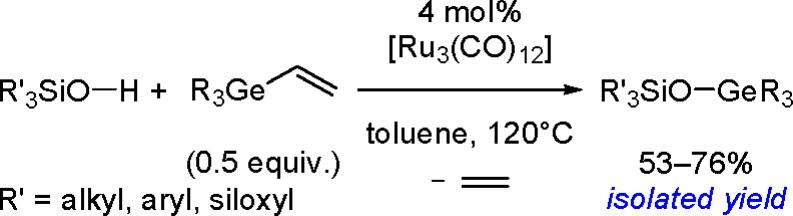
Ruthenium-Catalyzed Deethenative Germylation of Silanols

An analogous reaction of silanols with vinylgermanes in the presence of a ruthenium–carbonyl cluster ([Ru_3_(CO)_12_]) leads to the formation of silagermoxanes (Scheme 229).^548^ Selective transformations of trialkylvinylgermanes and diethyldivinylgermane have been described with moderate isolation yields (53%–76%).

**Scheme 230:**
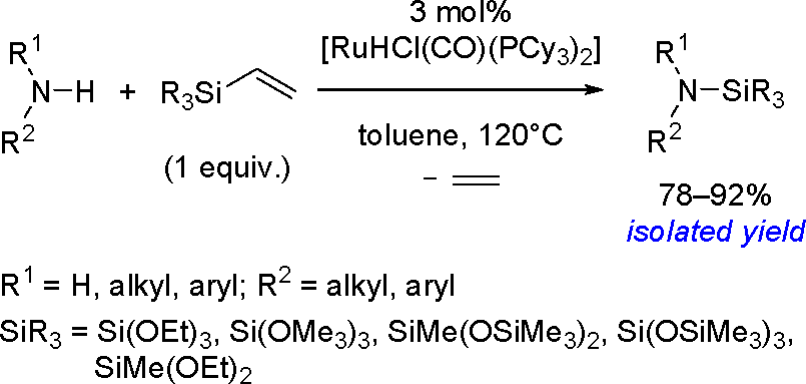
Ruthenium-Catalyzed Deethenative Silylation of Primary and Secondary Amines

To the best of our knowledge, the only example of amine *N*-silylation with vinylsilane is the one reported by Marciniec and co-workers.^549^ In the presence of the [RuHCl(CO)(PCy_3_)_2_] ruthenium hydride, primary and secondary amines undergo silylation by vinylsilanes, bis(silyl)ethene, or 1-alkenylsilanes to yield silylamines. Depending on the silylating agent used, the second product is ethene, terminal olefin, or vinylsilane (Scheme 230).

The scope of amine reagents includes examples of primary and secondary amines and carbazole. The reaction permits high yields of silylated amines (typical isolation yields of 78%–92%). A mechanism of the reaction has been proposed, but it needs to be corroborated by further experimental and computational studies.

## 7 Activation (and Functionalization) of C–H Bonds with Metalloid Halides and Sulfonates

This type of reactivity is possible through activation of the metalloid-halogen or sulfonate bond by oxidative addition to the transition metal complex.

### 7.1 Borylation of C–H Bonds

**Scheme 231:**
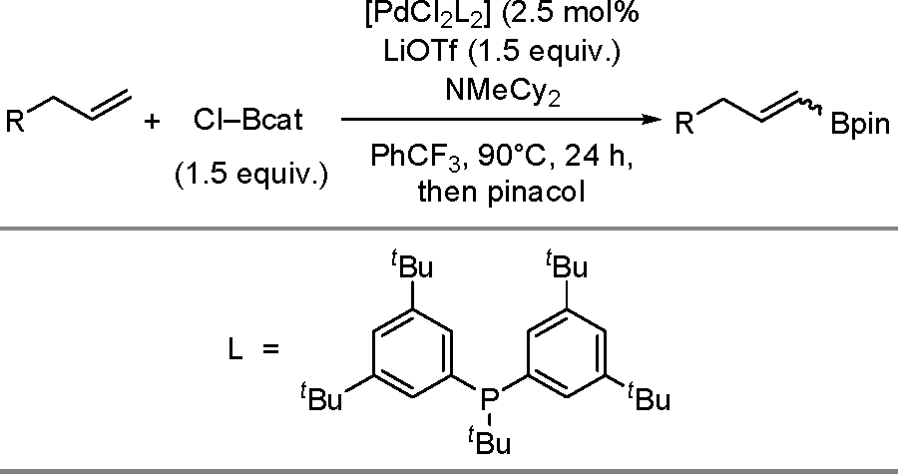
Borylation of Olefins with Chlorocatecholborate: Boryl-Heck Reaction

The first examples of the efficient palladium-catalyzed borylation of olefins using chlorocatecholborate as a borylating agent have been reported by Watson and co-workers (Scheme 231).^550^

A Heck-like reaction mechanism has been postulated that starts with the oxidative addition of a B–Cl bond to palladium(0).

### 7.2 Silylation and Germylation of C–H Bonds

The oxidative addition of bromosilane to platinum(0) complexes described by Tanaka in 1988 opened up new ways for using metalloid derivatives in synthesis.^551^ In subsequent papers, Tanaka^552^ and Murai^553^ reported the first applications of the process in palladium-catalyzed reactions. Several subsequent studies demonstrated that oxidative addition of a Si–halogen bond to metals such as Pd and Ni was possible. The results obtained by Watson, collected in several recent reviews,^554−556^ clearly show the great potential of halosilanes in catalyzed coupling processes.

**Scheme 232:**
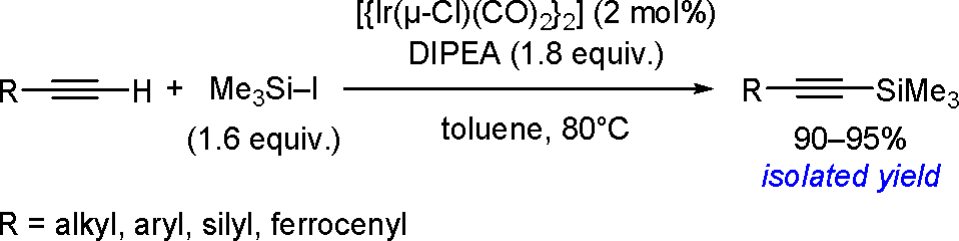
Silylation of Terminal Alkynes with Iodotrimethylsilane

**Scheme 233:**
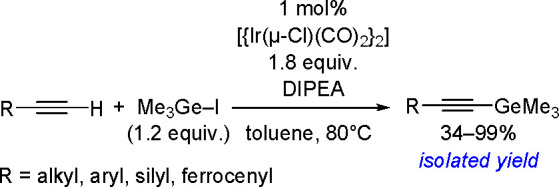
Germylation of Terminal Alkynes with Iodotrimethylgermane

Marciniec has reported that catalyzed treatment of terminal alkynes with iodotrimethylsilane (or iodotrimethylgermane) leads to the efficient synthesis of silylacetylenes and germylacetylenes, respectively. A series of silylacetylenes^557^ (Scheme 232) and germylacetylenes^558^ (Scheme 233) have been obtained in high yields in the presence of [{Ir(μ-Cl)(CO)_2_}_2_] and NEt(*i*-Pr)_2_.

**Scheme 234:**
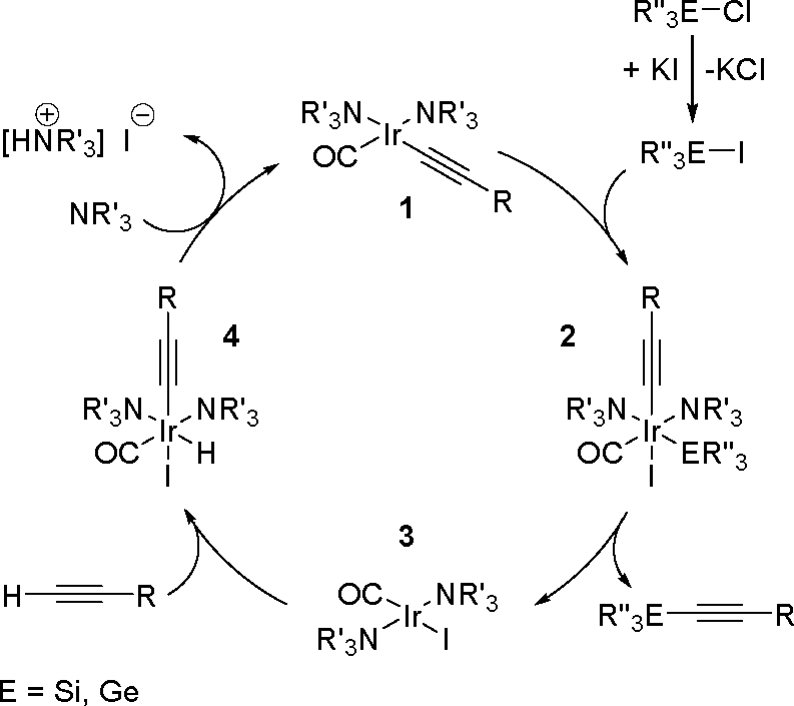
Mechanism of the Silylation (Germylation) of C(sp)–H Bonds with Chlorosilane (Chlorogermane)

Further research has shown that cheaper and more readily available chlorosilanes^559^ or chlorogermanes^560^ can be used as reaction partners. The synthetic method consists of an *in situ* generation of iodosilanes in a reaction with NaI or KI. The mechanism of the process has been proposed on the basis of the isolation of a hydridoalkynyl complex, studies of its stoichiometric reactions with reagents and DFT calculations (Scheme 234).

The active catalyst of the process is a square-planar σ-acetylene iridium(I) complex (**1**). Complex **1** undergoes oxidative addition of iodosilane and subsequent reductive elimination of the organosilicon product. Subsequently, the formed complex (**3**) undergoes oxidative addition of the C(sp)–H bond of acetylene. Finally, reductive elimination of hydrogen iodide (which is consumed by the reaction with amine) leads to the regeneration of the starting complex (**1**). An important fact in support of the proposed mechanism was the isolation of the [IrHCl(CO)(PhC≡C)py_2_] complex (model of complex **4**) and its characterization by X-ray diffraction and examination of stoichiometric reactions. DFT computations suggest that the reductive elimination of germylacetylene is the rate-limiting step of germylation.^560^ Still, further studies are needed to account for many details of the mechanism.

**Scheme 235:**
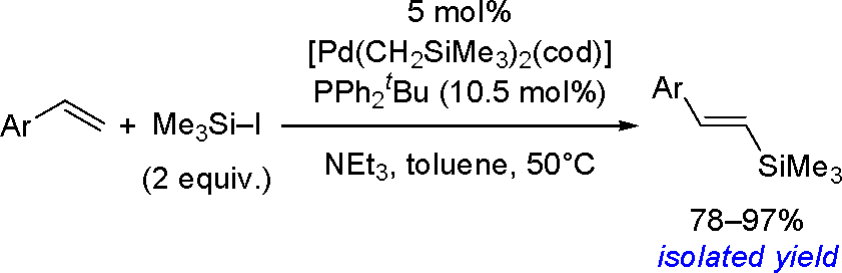
Silylation of Terminal Olefins with Iodotrimethylsilane

The coupling of iodotrimethylsilane with styrenes as olefinic partners proceeds stereoselectively and leads to efficient formation of E-silylstyrenes (Scheme 235).^561^ The results were obtained in the presence of the palladium(II) precursor [Pd(cod)(CH_2_SiMe_3_)_2_] and PPh_2_(*t*-Bu) used as a ligand.

**Scheme 236:**
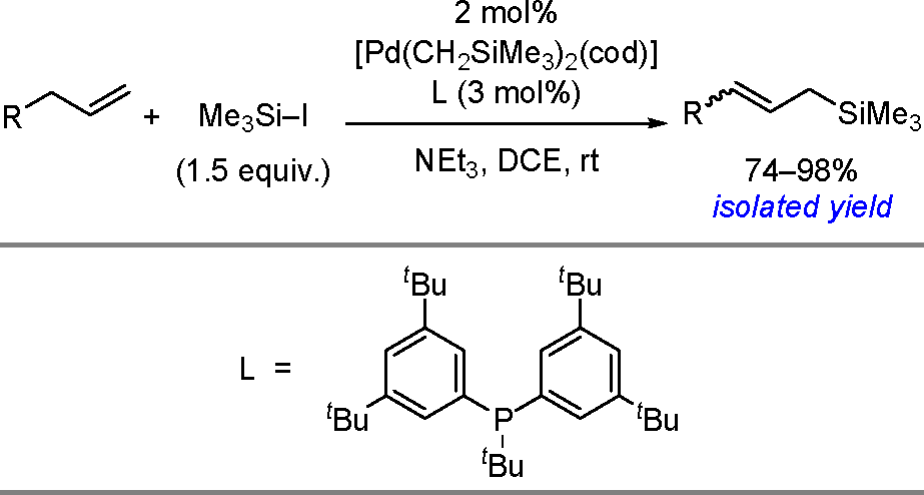
Stereoselective Silylation of Allyl Derivatives with Iodotrimethylsilane

The much cheaper chlorosilane can be used in the reaction in the presence of LiI, which enables the generation of iodosilane in situ.^561^ Coupling with alkyl-substituted α-olefins yields allylsilanes accompanied by vinylsilanes and the isomerized starting olefin. A strongly donating and bulky phosphine was used to achieve chemo- and stereoselective formation of allylsilanes (Scheme 236).^562^

The palladium dimer [{PdI_2_(P*t*-BuAr_2_)}_2_] (Ar = 3,5-di-*tert*-butylphenyl) developed by Watson enables efficient and stereoselective silylation of styrenes, selective silylation of terminal olefins to allylsilanes, and use of sterically demanding silyl groups such as SiMe_2_Ph, SiMePh_2_, and SiMe_2_CH_2_Ph.^563^

[Pd_2_(dba)_3_]/PtBuPh_2_ is an alternative catalytic system useful for the silylation of styrenes, which offers high efficiency and stereoselectivity and allows conversion with silyl bis(triflates). By using silyl bis(triflates) (in the presence of NaI to generate in situ monoiodosilanes), alkoxysilylderivatives could be obtained, which can easily undergo further transformations, such as Hiyama–Denmark coupling and Tamao–Fleming oxidation.^564^

**Scheme 237:**
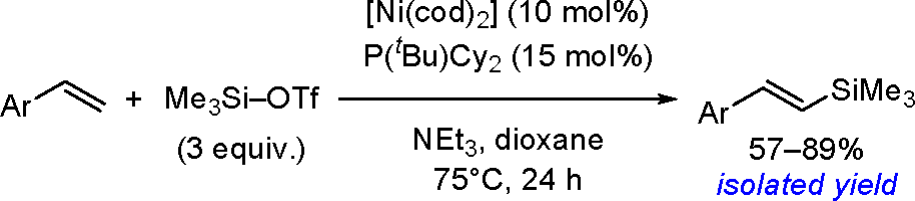
Olefin Silylation with Trimethylsilyltriflate

A commercially available nickel(0) catalyst precursor, [Ni(cod)_2_], used with the appropriate phosphine ligand enabled oxidative addition of silyl triflates (Scheme 237).^565^

Nickel catalysts show good functional group tolerance in the production of vinylsilanes; however, the electron-withdrawing groups present in the aromatic rings significantly reduce conversion. Appropriate selection of the phosphane ligand enabled the conversion of silanes bearing bulkier silyl groups than Me_3_Si. Trichlorosilanes, dichlorosilanes, and monochlorosilanes were shown to react with an olefin in the presence of [Ni(cod)_2_], PCy_3_, AlMe_3_, and NEt_3_ to yield corresponding alkenylsilanes. The process is the first example of a direct silyl-Heck reaction of chlorosilanes.^566^

**Scheme 238:**
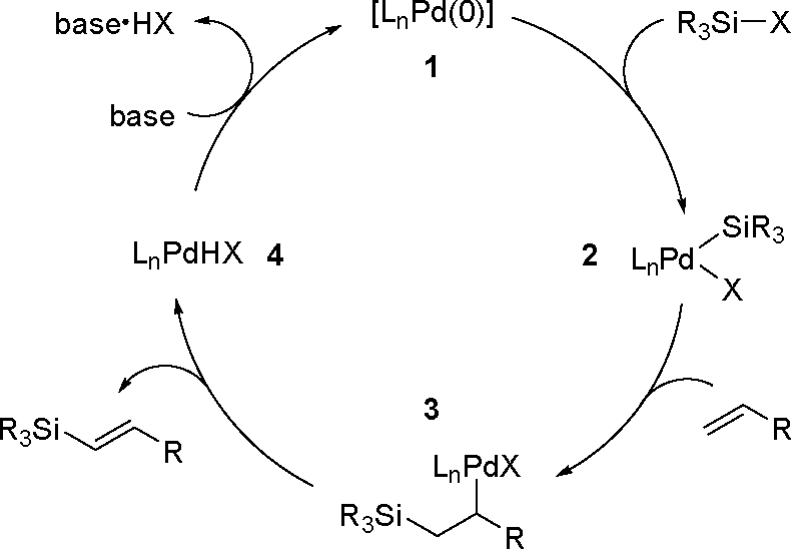
Mechanism of the Ni-Catalyzed Silylation of Olefinic C(sp^2^)–H Bonds with Halosilane (Silyl-Heck Reaction)^554^

In terms of the reaction mechanism (Scheme 238), the key step is the oxidative addition of the Si–X bond (X = halogen) to the metal. It has been found that [Pd(CH_2_SiMe_3_)_2_(cod)] undergoes oxidative addition of iodosilane to form [PdI(SiMe_3_){PAr_2_(*t*-Bu)}] (Ar = 4-hydroxy-3,5-dimethylphenyl), stabilized by a phosphine ligand. The complex was characterized by spectroscopic and X-ray diffraction methods. The complex was demonstrated to be a competent precatalyst in the reaction of iodosilane with 1-decene.^562^ The further steps in the process were proposed to follow the Heck reaction mechanism. The olefin is inserted into the resulting M–Si bond followed by β-silyl elimination. The last step is the reductive elimination of the hydrogen halide, which is favored when a base is present. However, the proposed mechanism still needs further detailed investigation.

## 8 Applications in Organic Synthesis

In the past two decades, the catalytic methods for the synthesis of organometalloid compounds have been rapidly developing, as described in detail in sections 1–7 of our Review. Through the in-depth understanding of the reaction mechanisms and the role of inorganometallic intermediates in catalysis, many new addition and coupling reactions involving hydro-, halogen-, vinyl-, and bis-metalloids have been discovered, which opened up new routes to the selective and effective preparation of several metalloid-containing derivatives.

However, it has been known for many years that the unique reactivity of organometalloids can successfully be used in organic synthesis. As a result, new synthetic strategies have been developed using mostly unsaturated or (hetero)aromatic boron and silicon derivatives as precursors for subsequent functionalization reactions to yield a wide variety of organic derivatives from available and inexpensive substrates (alkenes, alkynes, arenes, etc.). The application of tin and germanium compounds, although less spectacular, has also been intensively studied, and interesting applications in organic synthesis followed. The literature also provides some examples of using tellurium and antimony compounds.

**Scheme 239:**
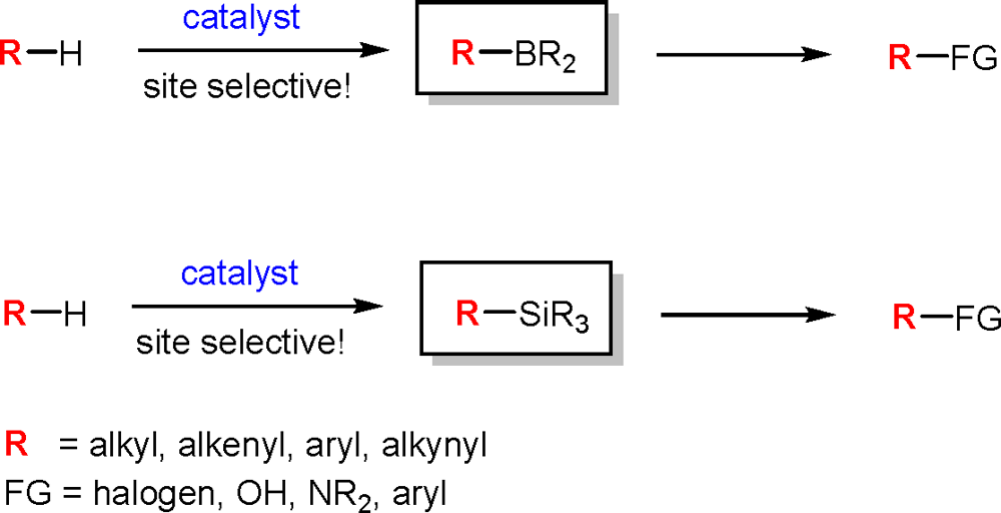
Functionalization of C–H Bonds via Borylation (Silylation) and Subsequent Derivatization of C–B (C–Si) Bonds

Among the organic derivatives of metalloids, organoboron compounds are typically used in organic synthesis. Silicon derivatives are used slightly less commonly. The procedures for the selective and efficient borylation and silylation of C–H bonds allow convenient and selective introduction of a number of functional groups into an organic molecule as a result of further functionalization (Scheme 239).^227,230,297,567^

The unique feature of these sequential methodologies is that the metalation step and further functionalization can usually be performed in a one-pot procedure. The selectivity of the overall processes can be controlled during the initial step (which is especially important for addition or aromatic substitution reactions) as the subsequent demetalation usually proceeds with retention of the configuration (*ipso*-substitution) at the carbon atom, and the desired products can be formed.^568^

**Scheme 240:**
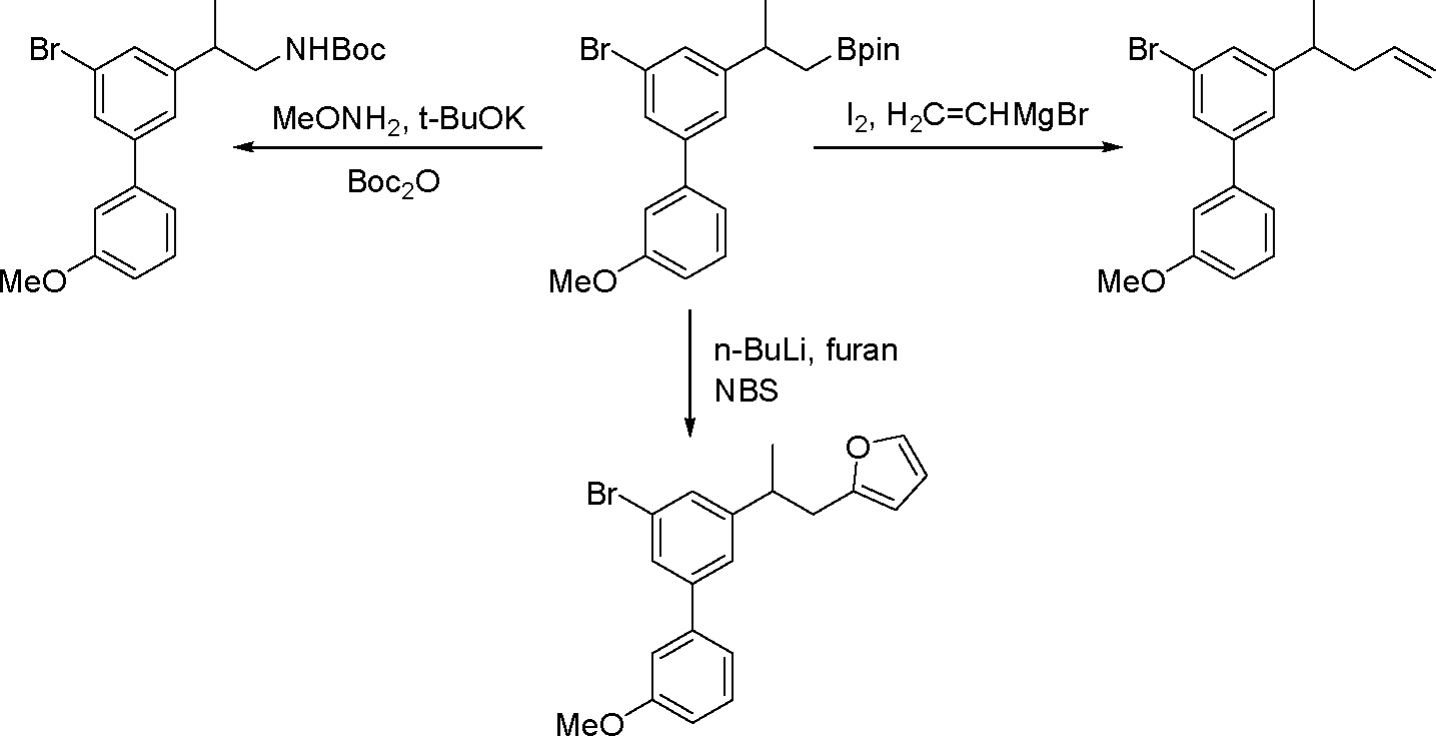
Synthetic Application of Alkylboronic Acids and Esters

The high selectivity and high yields of the metal-catalyzed borylation of alkanes enables the synthesis of functionalized materials that are hardly formed selectively by other methods. Owing to the selective borylation of primary C–H bonds or C–H bonds located alpha to heteroatoms or alpha to an aryl ring, alkylborates are widely used in organic synthesis with the borylation-functionalization sequence. A recent procedure reported by Hartwig and co-workers^567^ enables undirected borylation of primary C–H bonds or borylation of strong secondary C–H bonds when primary C–H bonds are absent or blocked. It opens up a range of possibilities for installing a vast variety of carbon–carbon and carbon–heteroatom bonds at previously inaccessible positions of organic molecules (Scheme 240).

**Scheme 241:**
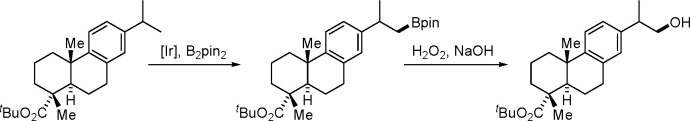
Borylation and Oxidation of Dehydroabietic Acid *tert*-Butyl Ester

As borylation can be carried out with the substrate as the limiting reagent, it enables borylation of multifunctional natural products or medically relevant molecules containing multiple C–H bonds (Scheme 241).^567^

**Scheme 242:**
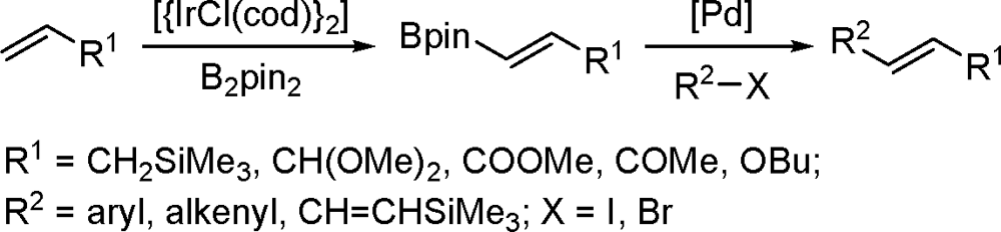
Synthesis of Allylsilanes and Dienylsilanes by a One-Pot C–H Borylation–Suzuki–Miyaura Coupling Sequence

The synthesis and applications of alkenylboronates were summarized in a recent review.^569^ Alkenylboronates are used primarily in Suzuki–Miyaura cross-coupling reactions. Olsson and Szabo demonstrated that a number of allylsilanes and dienes could be synthesized using a sequence of borylation of allylsilane (or selected olefins) and cross-coupling with an aryl or alkenyl halide (Scheme 242).^238^

The synthesis and reactivity of alkynyl boron compounds has been described in review articles.^231,570^ Catalytic transformations that occur via the activation of C–B bonds involve halodeboration, syn-alkynylboration of alkynes, and cross-coupling of alkynyl boronic esters with aryl, alkynyl and acyl halides. It has been demonstrated that alkynyl boronates can also be efficiently transformed into carboxylic acids, esters, and amides.^571^

**Scheme 243:**
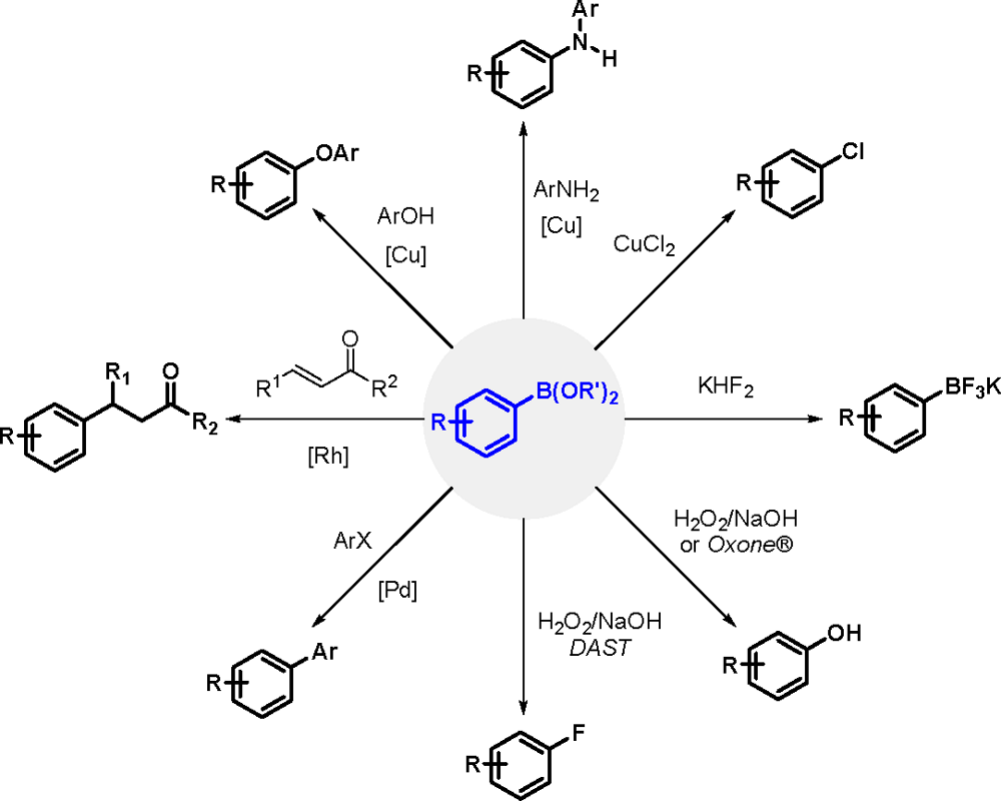
Synthetic Applications of Arylboronic Acids and Esters

Arylboronic acids and esters, synthesized by the catalytic borylation of C–H bonds with hydro- or diboranes, open up a gateway to a plethora of derivatization methods for the selective installation of complex and valuable functional groups through oxidation, amination, halogenation, arylation, etherification, homologation, and cross-coupling for the selective preparation of functionalized arenes (Scheme 243).^238^ The advantage of these synthetic protocols is that the sequences of borylation and deborylative functionalization can be implemented as a one-pot procedure without the isolation of organoboron intermediates.

Multiborylated compounds, readily available owing to convenient synthesis protocols (e.g., refs (572−575)) are valuable intermediates for the synthesis of multifunctional molecules.^575,576^

**Scheme 244:**
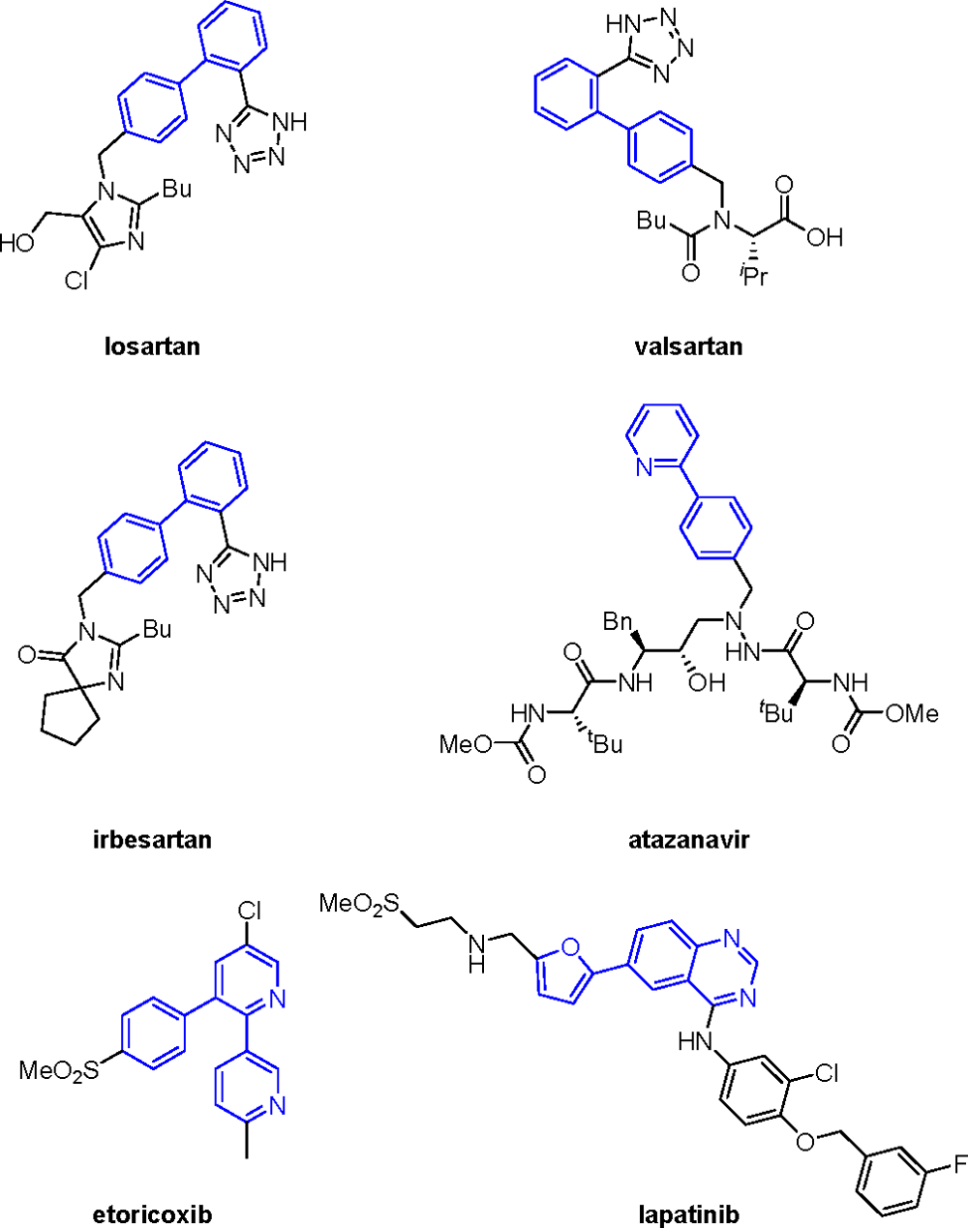
Structures of Commercial Drugs Produced with Suzuki–Miyaura Cross-coupling to Form Carbon–Carbon Bonds

Suzuki–Miyaura coupling, a major application of organoboron compounds, is widely used both in the synthesis of small molecules and as a key step in the overall synthesis of pharmaceuticals and natural products.^577−582^ Its growing popularity in pharmaceutical industry comes from its ability to drive a wide range of C(sp^2^)–C(sp^2^) couplings to generate a wide gamut of (hetero)biaryl motifs in a selective manner, while displaying a high level of functional group tolerance.^583,584^ Moreover, palladium-catalyzed coupling reactions, with boronic acids or boronates, ensure considerably shorter routes to corresponding products compared to other approaches, thus minimizing side products and waste. Examples of the approved drugs whose production employs Suzuki–Miyaura coupling to form critical carbon–carbon bonds with aromatic or heteroaromatic groups are presented in Scheme 244.

**Scheme 245:**
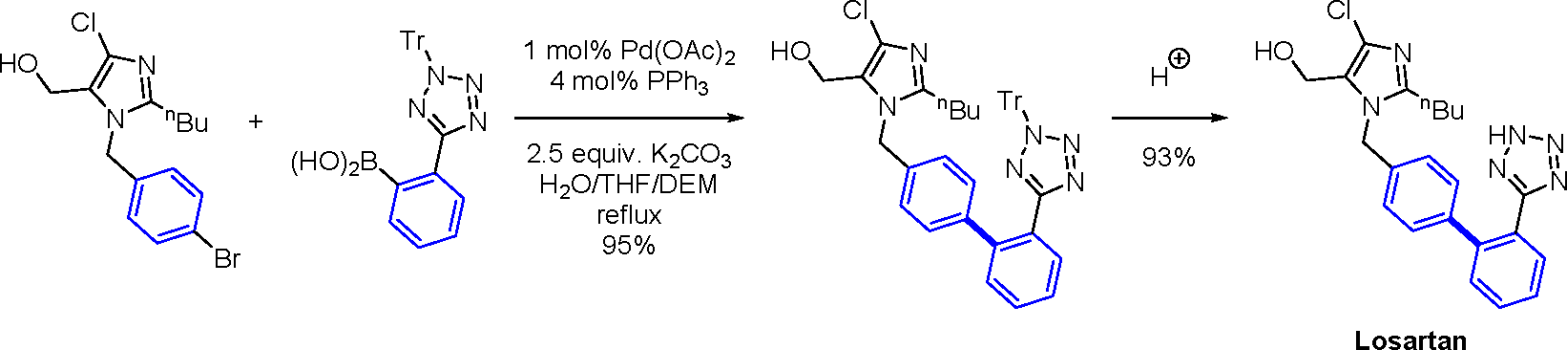
Application of Suzuki–Miyaura Coupling for the Synthesis of Losartan

As an example, the synthesis of *losartan* (Merck), an angiotensin II receptor antagonist prescribed worldwide for high blood pressure, is shown in Scheme 245. Owing to optimization of the cross-coupling reaction conditions, cost-effective solutions could be applied on a large scale with the Pd(OAc)_2_/PPh_3_ catalytic system and a mixture of water, THF, and dimethoxymethane as solvent.

**Scheme 246:**
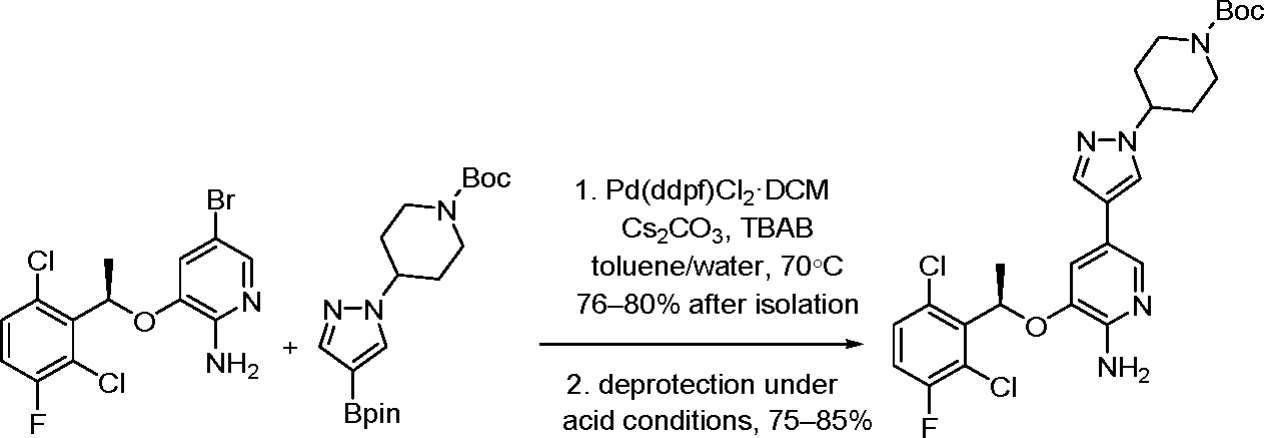
Application of Suzuki–Miyaura Coupling in the Large-Scale Synthesis of Crizotinib

Through phase-transfer catalytic conditions for the cross-coupling step, efficient large-scale synthesis of crizotinib could be developed (Scheme 246) as a mesenchymal epithelial transition factor/anaplastic lymphoma kinase inhibitor (about 100 kg via a six-step procedure in 40% yield from chiral (*S*)-1-(2,6-dichloro-3-fluorophenyl)ethanol).^584^

**Scheme 247:**
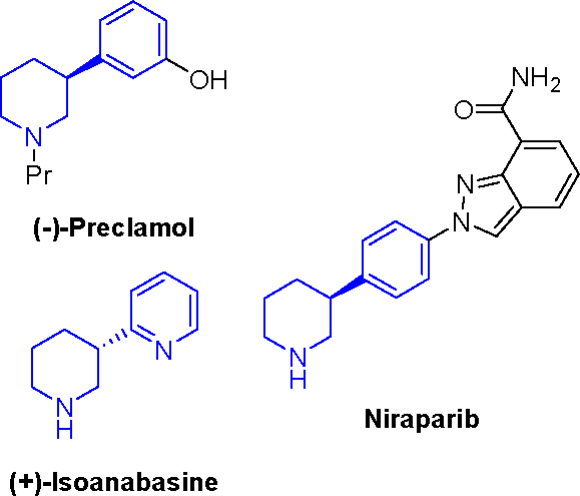
Examples of Molecules Synthesized via Asymmetric Suzuki–Miyaura Coupling Reactions

Cross-coupling chemistry has been vital for the rapid assessment of structure–activity relationships in the drug discovery phase to facilitate subsequent selection of drug candidates. A synthetic methodology based on the rhodium-catalyzed asymmetric Suzuki–Miyaura reaction was used for the synthesis of potential drug candidates containing chiral molecules, such as *preclamol* (studied for the treatment of schizophrenia), *niraparib* (ovarian cancer), and a natural alkaloid *isoanabasine* (Scheme 247).^583^

**Scheme 248:**
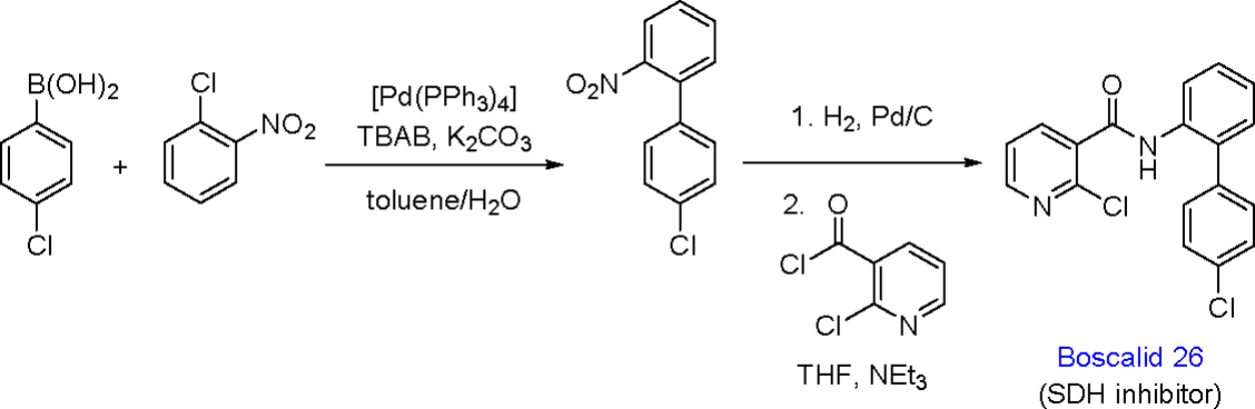
Application of Suzuki–Miyaura Coupling for the Synthesis of Boscalid

Suzuki–Miyaura coupling is also widely applied in the synthesis of agrochemicals such as fungicides (e.g., *boscalid*, *bixafen*, *pyriofenone*), insecticides (*bifenazate*) and herbicides (*halauxifen-methyl*).^585^ As an example, the cross-coupling of 4-chlorophenylboronic acid with 1-chloro-2-nitrobenzene mediated by Pd(PPh_3_)_4_ with the addition of tetrabutylammonium bromide (TBAB) as a phase-transfer catalyst is a key step to produce *boscalid* on a large-scale (>1000 tons per year). Low catalyst loading (0.25 mol % Pd), functional group tolerance and use of cost-effective aryl chlorides make this method one of the largest-known industrial applications of the Suzuki–Miyaura cross-coupling reaction (Scheme 248).

Recently, straightforward silylation of C–H bonds with hydrosilanes catalyzed by Rh, Ir, or Pt complexes has been extensively studied in parallel with similar transformations with boron reagents to achieve much easier access to novel organosilicon and organoboron compounds. Organosilicon compounds are in general superior to other organometalloid compounds in view of their stability, solubility, nontoxicity, and ease of handling.

**Scheme 249:**
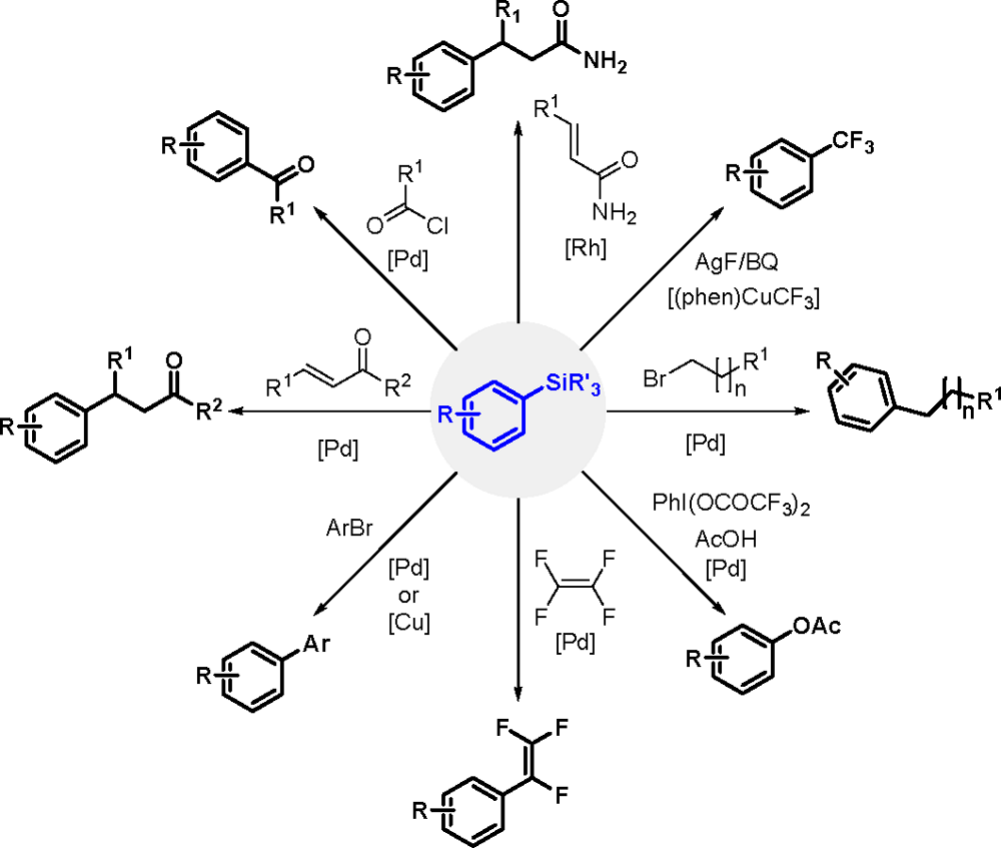
Synthetic Utility of Arylsilanes

Arylsilanes, heteroarylsilanes and alkenylsilanes obtained via C–H bond silylation reactions are highly versatile substrates that can be transformed into several compounds with useful functionalities, such as halides, ketones, and esters; they are also valuable precursors for cross-coupling with aryl, alkenyl, and alkyl halides (Scheme 249).^297,586,587^

**Scheme 250:**
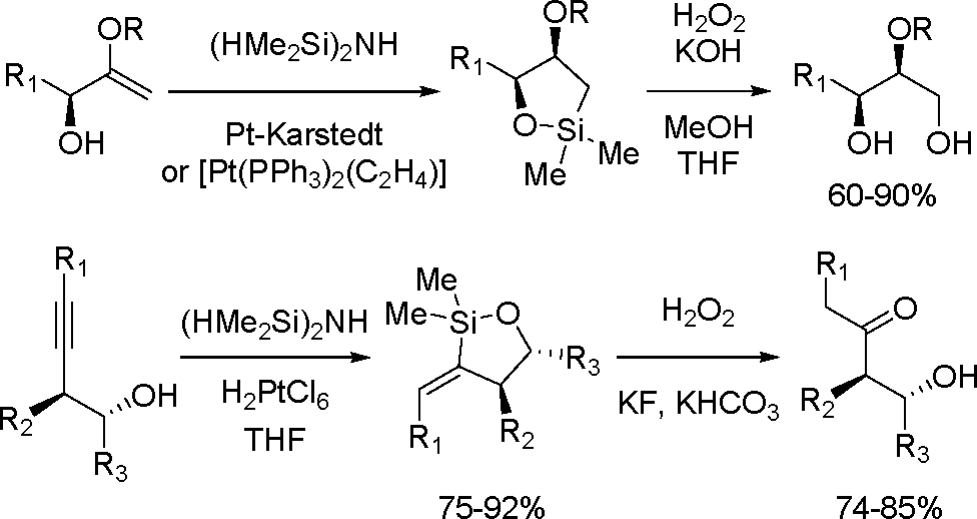
Synthesis of Alcohols via the Hydrosilylation/Tamao-Fleming Oxidation Sequence

The growing interest in the development of sequential or tandem processes, including intramolecular hydrometalation and bis-metalation as the initial steps, stems from the ability to assemble complex molecules from simple starting materials (functionalized alkenes, dienes, and alkynes) through organometalloid intermediates in a convergent and stereoselective manner. Classical examples involve the use of the platinum-catalyzed intramolecular hydrosilylation/Tamao–Fleming oxidation sequence as a powerful method for the stereoselective synthesis of various structurally diverse alcohols (1,3-diols, 2-alkoxy-1,3-diols, 1,3,5-triols, 2-aminoalcohols) and ketone derivatives (β-hydroxyketones, γ-hydroxyketones, α,β-dihydroxyketones, and α,γ-dihydroxyketones) from simple and readily available substituted allyl- or propargyl alcohols and their homologues (Scheme 250).

**Scheme 251:**
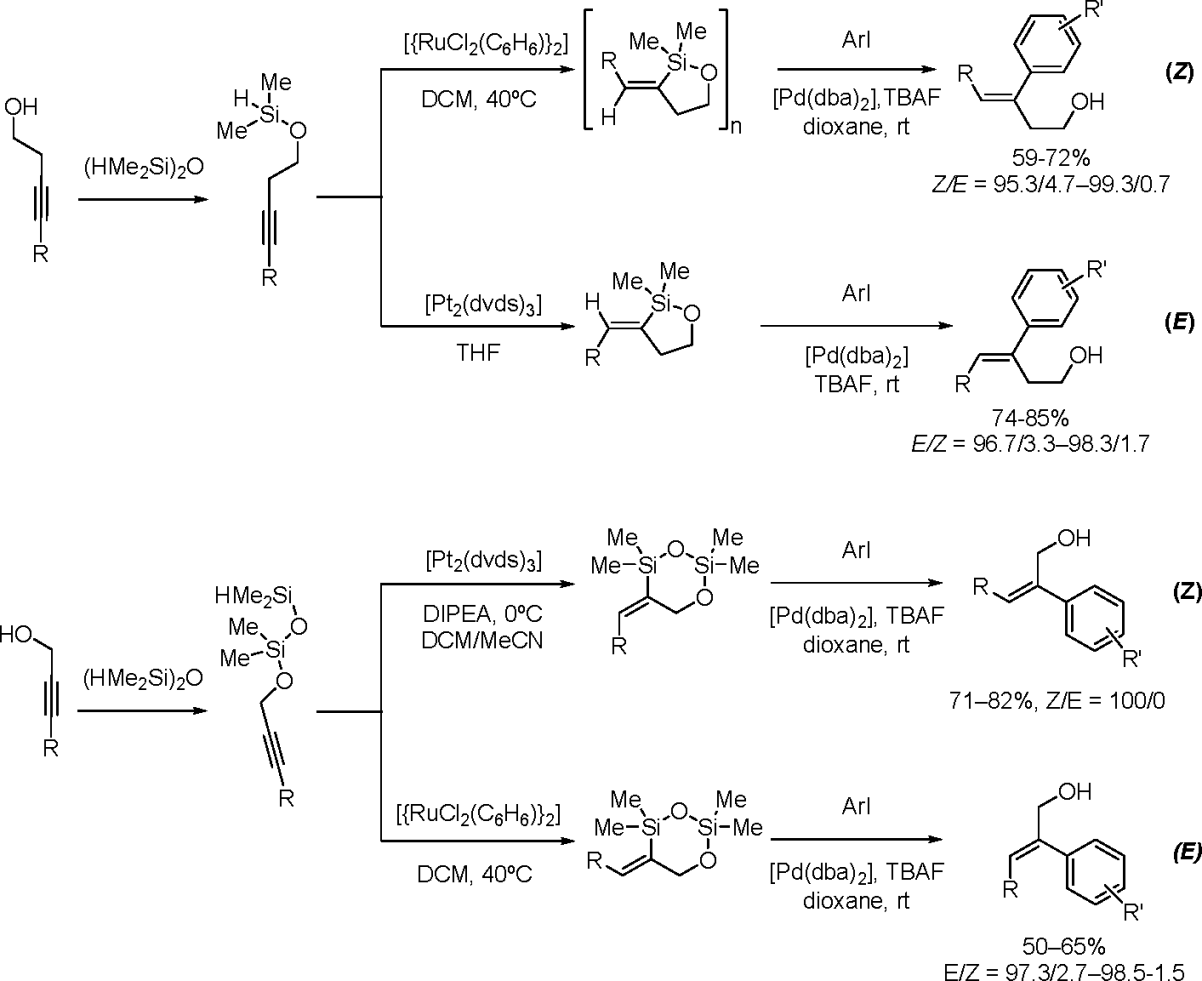
Synthesis of Aryl-Substituted Allyl and Homoallyl Alcohols by Sequential Hydrosilylation/Hiyama–Denmark Cross-coupling

Several protocols using a combination of TM-catalyzed hydrosilylation and a palladium-catalyzed cross-coupling (Hiyama–Denmark coupling) sequence leading to stereodefined π-conjugated alkene derivatives have been successfully developed in the past two decades.^587,588^ Consecutive platinum- or ruthenium-catalyzed intramolecular hydrosilylation of propargyl and homopropargyl alcohols in combination with palladium-catalyzed cross-coupling reactions has been shown as a powerful tool in the stereoselective synthesis of a wide variety of aryl-substituted (*E*)- and (*Z*)-allyl and homoallyl alcohols (Scheme 251)^589,590^ and in the synthesis of natural products.^591,592^

**Scheme 252:**
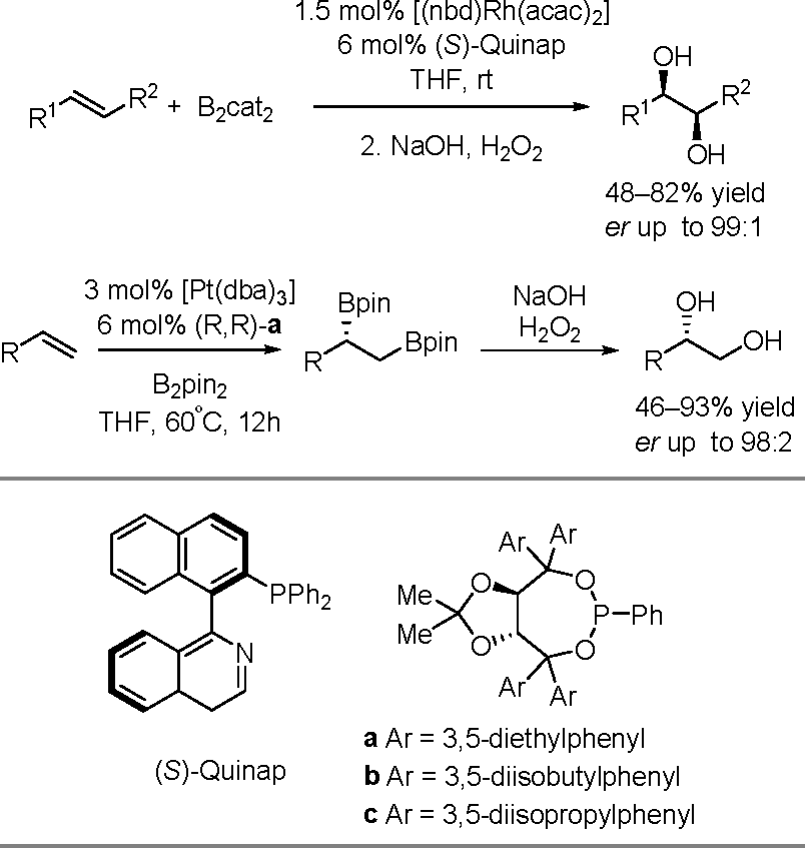
Enantioselective Bis-boration in the Synthesis of 1,2-Diols

A combination of the intramolecular bis-silylation of protected disilanyl homoallylic alcohols in the presence of a palladium catalyst and the oxidation of silacyclic products provides a unique method for diastereoselective alkene dihydroxylation.^593^ The development of enantioselective bis-boration gave the impetus to the selective synthesis of diols. An example is the bis-boration of terminal alkenes and *trans*-alkenes in the presence of rhodium and an (*S*)-Quinap ligand, followed by oxidation to efficiently give vicinal diols with high enantioselectivity.^190^ A combination of the bis-boration of terminal alkenes in the presence of a Pd(0) catalyst and chiral TADDOL-derived phosphonites with subsequent oxidation has been demonstrated to be an effective synthetic route to 1,2-diols with high enantioselectivity (Scheme 252).^594^ An alternative transformation applicable to 1,2-bis(boryl)alkane intermediates that involves tandem rhodium-catalyzed bis-boration and Suzuki cross-coupling has also been reported.^595^ A similar reaction sequence has been performed for palladium catalysts.^596^

**Scheme 253:**
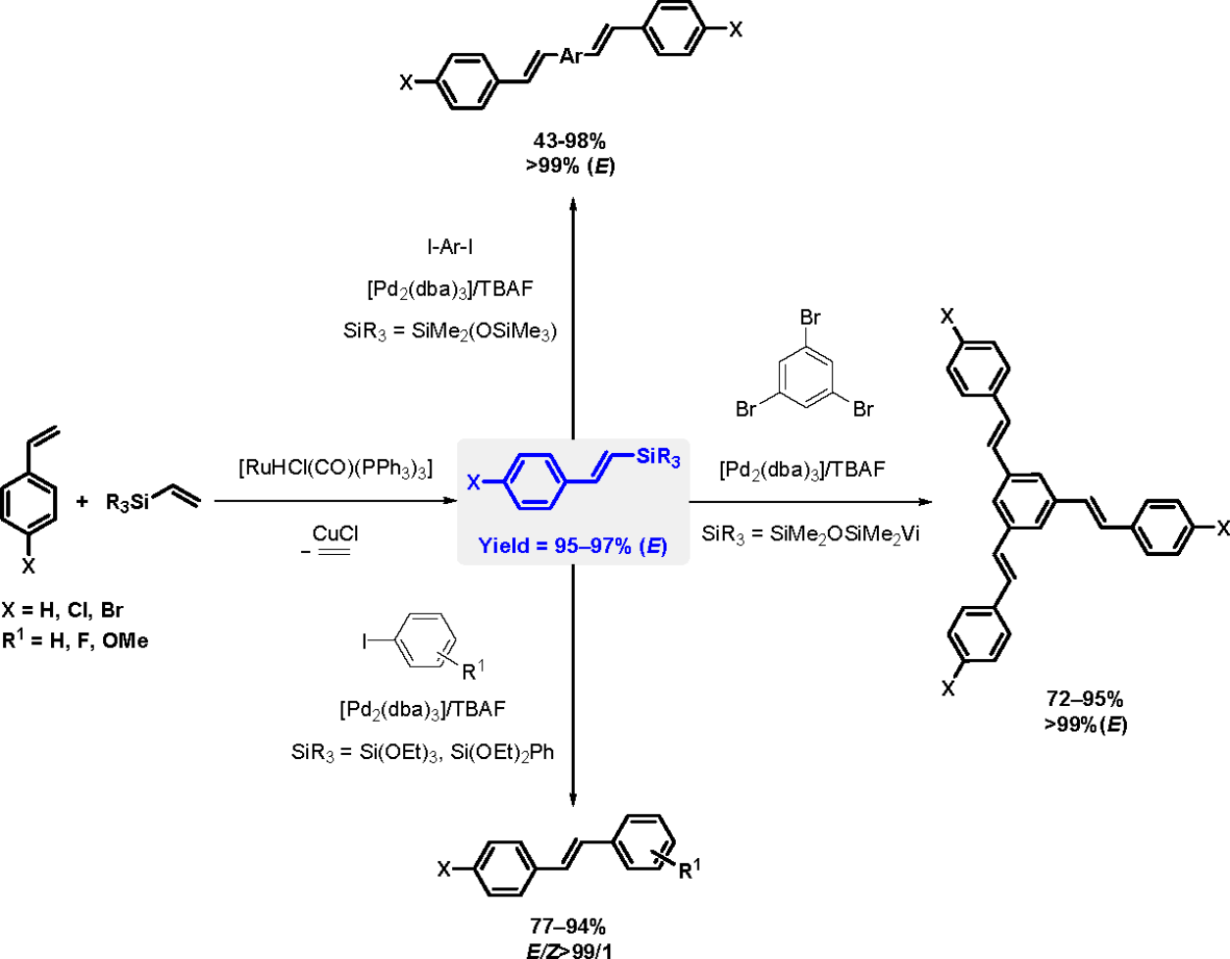
Sequential Deethenative Silylation/Hiyama–Denmark Cross-coupling in the Synthesis of π-Conjugated Derivatives

The deethenative silylation of olefins with vinyl-substituted organosilicon compounds catalyzed with ruthenium complexes has been a very attractive and highly efficient method for the synthesis of (*E*)-alkenylsilanes and bis(silyl)alkenes. These products have been used as effective platforms for further transformations, including palladium-catalyzed cross-coupling with aryl or alkenyl halides or electrophilic substitution.^529,597^ The combination of deethenative silylation and Hiyama–Denmark coupling has been used for the stereoselective synthesis of (*E*)-stilbenes, (*E*)-styrylarenes and arylene-vinylene oligomers (Scheme 253).

**Scheme 254:**
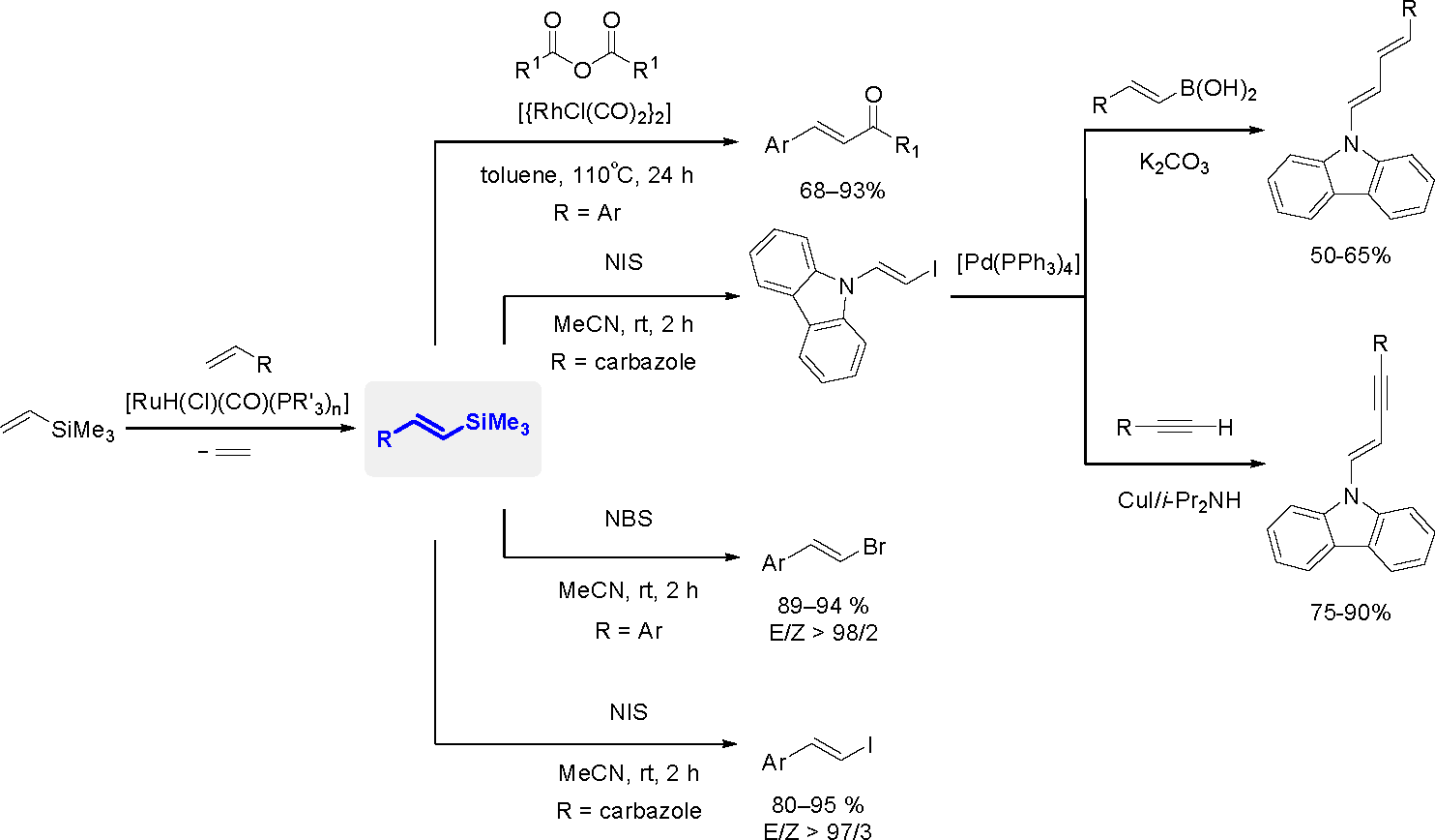
Deethenative Silylation of Olefins with Vinylsilanes in the Synthesis of Functionalized Organic Compounds

However, a combination of deethenative silylation with the electrophilic halodesilylation reaction has been applied in the selective one-pot preparation of synthetically useful alkenyl halides (e.g., (*E*)-styryl halides, (*E*)-*N*-2-iodovinylcarbazole, (*E*)-*N*-2-iodovinylamides). These halides are versatile coupling partners in palladium-catalyzed Suzuki and Sonogashira couplings, leading to a wide variety of stereodefined β-substituted (*E*)-enimides, (*E*,*E*)-dienimides, and (*E*)-enynimides as well as related π-conjugated *N*-substituted carbazole derivatives. The discovery of sequential deethenative silylation and rhodium- or iridium-catalyzed acylation reactions is important for the synthesis of (*E*)-α,β-unsaturated ketones (Scheme 254).

**Scheme 255:**
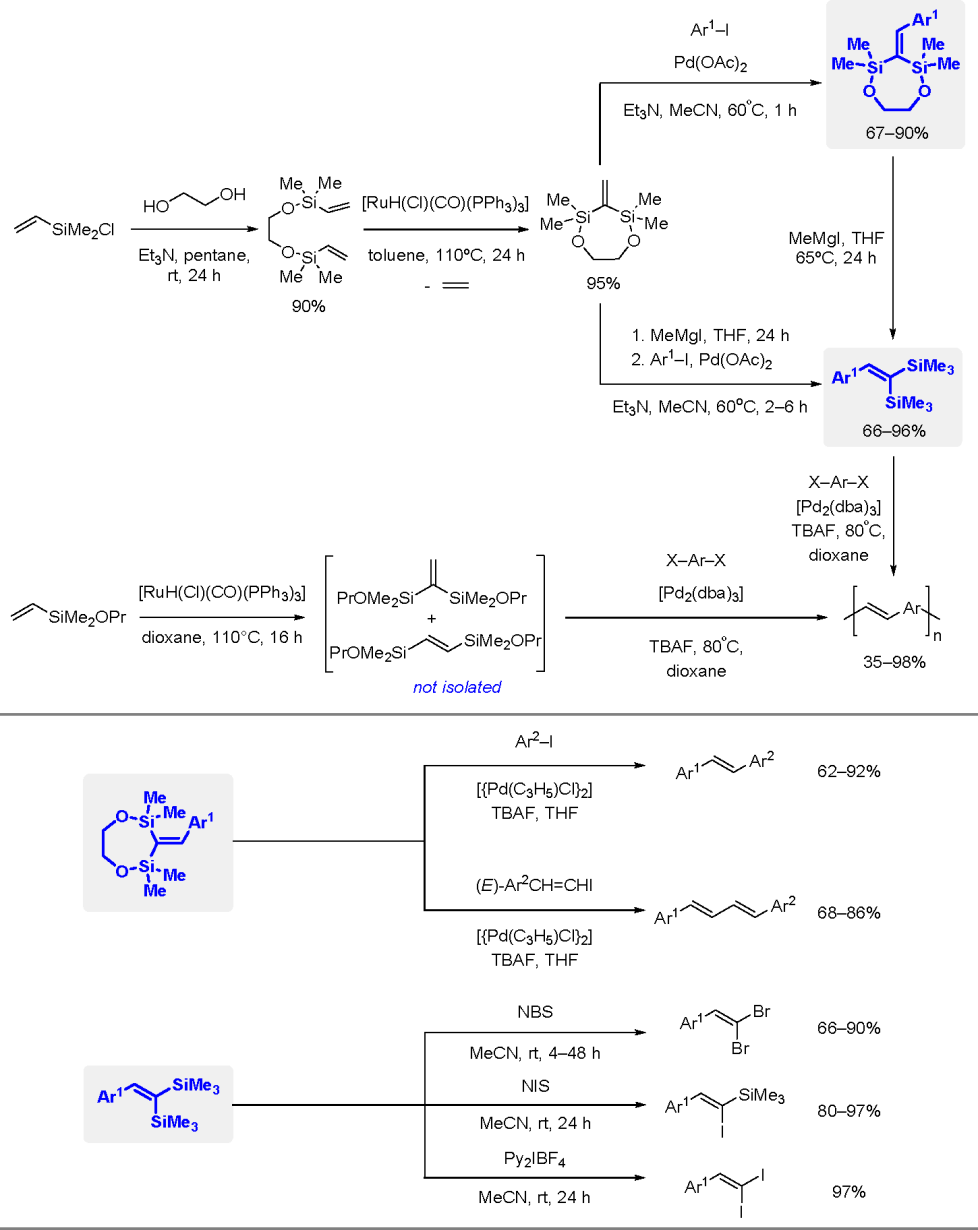
Application of Bis(silyl)alkenes in the Synthesis of Functionalized Organic Compounds

Isomeric bis(silyl)alkenes, easily obtained via the ruthenium-catalyzed deethenative silylation of divinyl-substituted organosilicon compounds or silylative homocoupling of vinylsilanes, are particularly attractive scaffolds for further transformations including palladium-catalyzed cross-coupling with organic halides or substitution with organic and inorganic electrophiles (Scheme 255).

**Scheme 256:**
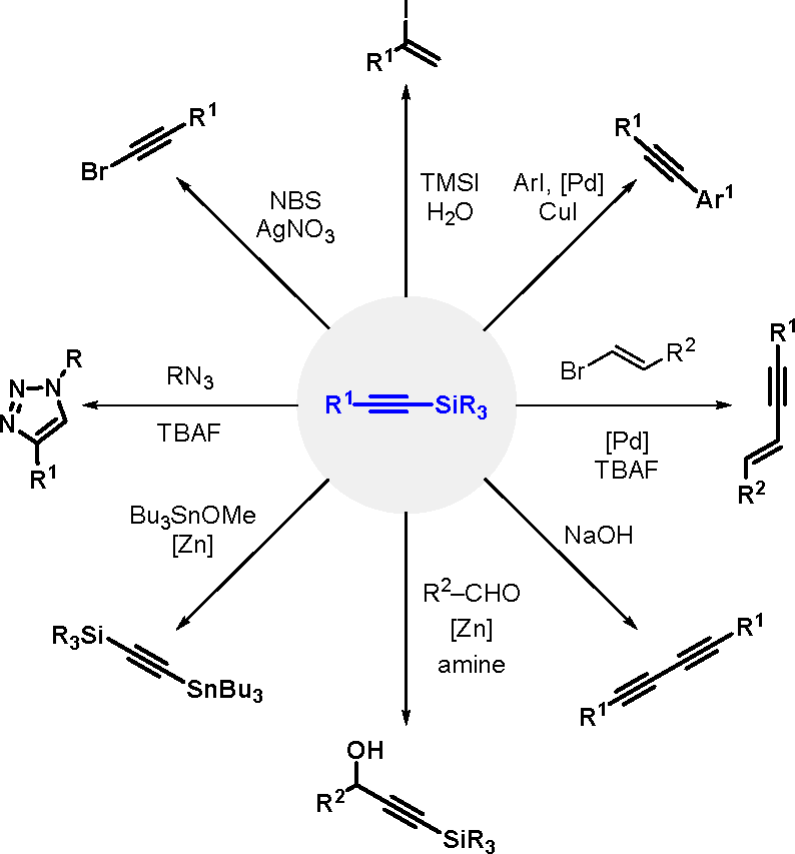
Synthetic Utility of Alkynylsilanes

Alkynylsilanes obtained through the catalytic silylation of terminal alkynes with hydro-, vinyl-, or halosilanes can act as versatile building blocks in the synthesis of π-conjugated derivatives with triple and double bonds.^301^ Selected examples of alkynylsilane transformations in desilylative substitution, cycloaddition, and cross-coupling reactions are presented in Scheme 256.

Germanium, despite its position in group 14 of the periodic table between silicon and tin, which causes organogermanium compounds to exhibit properties between those of organosilicon and organotin compounds, has not received significant interest from synthetic organic chemists. The state of the art on the use of germanium compounds in organic synthesis up to the early 2000s has been reviewed.^598^ The first application of organogermane derivatives in catalytic coupling was reported in 1996 by Kosugi and co-workers.^599^ It has been shown that variously substituted germatranes undergo coupling with tolyl (or phenyl) bromide in the presence of a palladium catalyst, [Pd_2_(dba)_3_CHCl_3_]/PPh_3_ or P(o-tol)_3_. In 2002, Oshima and co-workers described the Pd-catalyzed coupling of aryl iodides or bromides with aryltri(2-furyl)germane.^452^ Subsequent studies included proposals for efficient and selective cross-coupling reactions for organogermanes with organic halides and some other derivatives. Recently, dynamic progress in the application of trialkylarylgermanes has been made in the work of Schoenebeck and Fricke, as discussed in an excellent account.^600^ Schoenebeck has shown that organogermanium derivatives can be characterized by complementary reactivity in relation to that of other coupling agents. For example, it has been shown that aryltriethylgermanes exhibit efficient coupling with aryl halides in the presence of Pd nanoparticles as opposed to corresponding boronic acids or their esters. The reactivity series is reversed in a homogeneous system with palladium(0) and palladium(II) complexes. A useful property of aryltriethylgermanes is their tolerance to nucleophilic and electrophilic fluorinating agents, which distinguishes them from tin, silicon, and boron compounds.

**Scheme 257:**
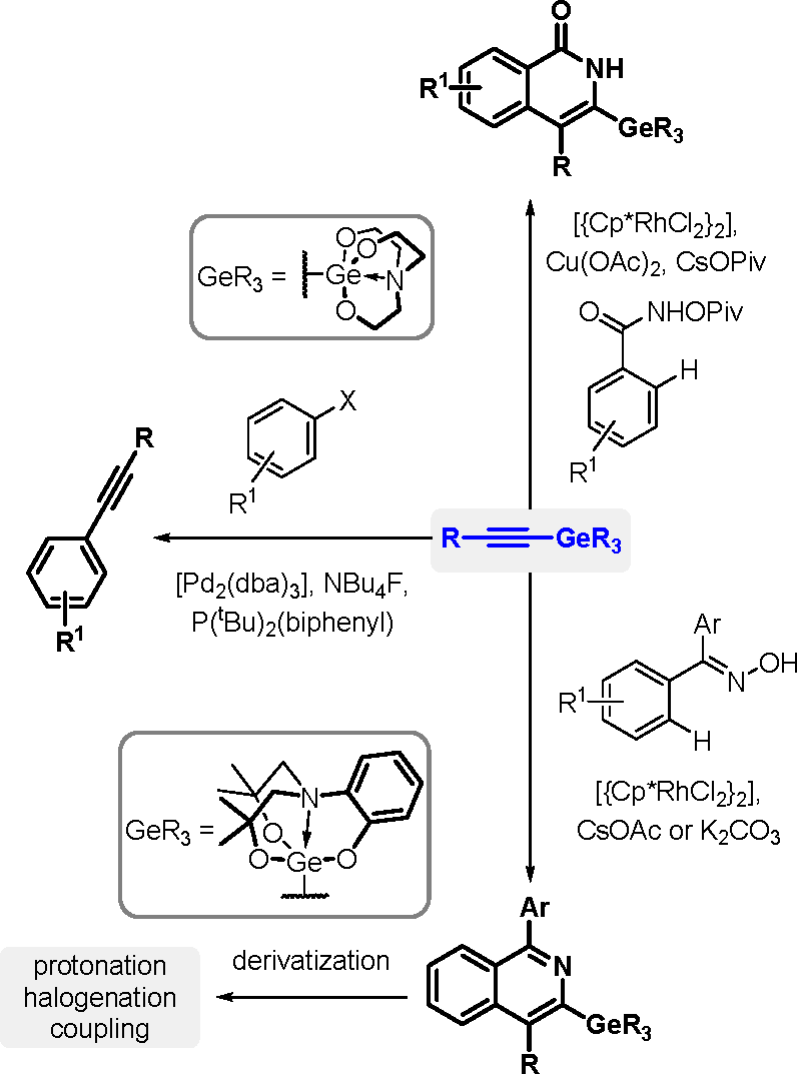
Alkynylgermanes in Organic Synthesis

Alkynyl germanium derivatives have been used in catalytic coupling reactions only in the form of germatranes (Scheme 257).

**Scheme 258:**
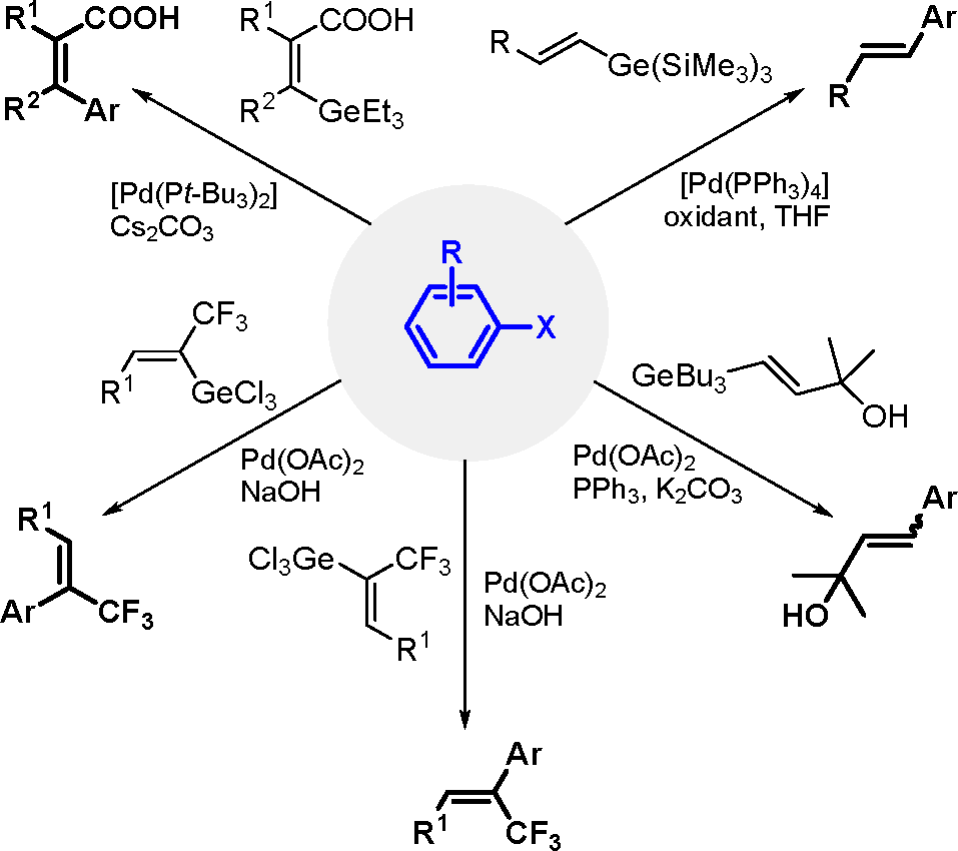
Catalyzed Cross-coupling of Vinylgermanes

Vinylgermanes play a limited role in organic synthesis. The literature describes in particular examples of palladium-catalyzed coupling processes (Scheme 258). The catalytic coupling of vinylgermatrane with p-bromotoluene by Kosugi and co-workers is the first reported example.^599^

**Scheme 259:**
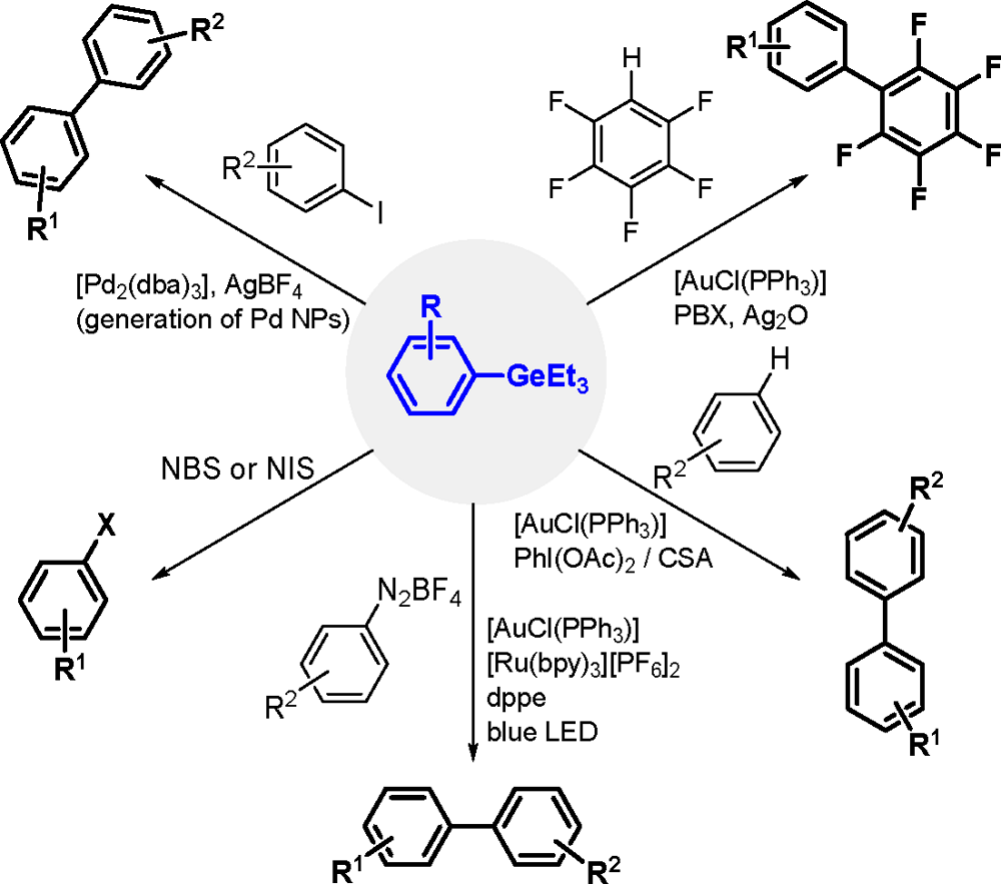
Synthetic Utility of Arylgermanes

Cross-coupling of Z-β-(trialkylgermyl)acrylic acid with 4-iodobenzaldehyde was used in the total synthesis of (Z)-tamoxifen.^601^ The observation of arylgermanium activity in gold-catalyzed coupling with an aryl C–H bond will hopefully make organogermanes more useful as transmetalation agents (Scheme 259).^602^

**Scheme 260:**
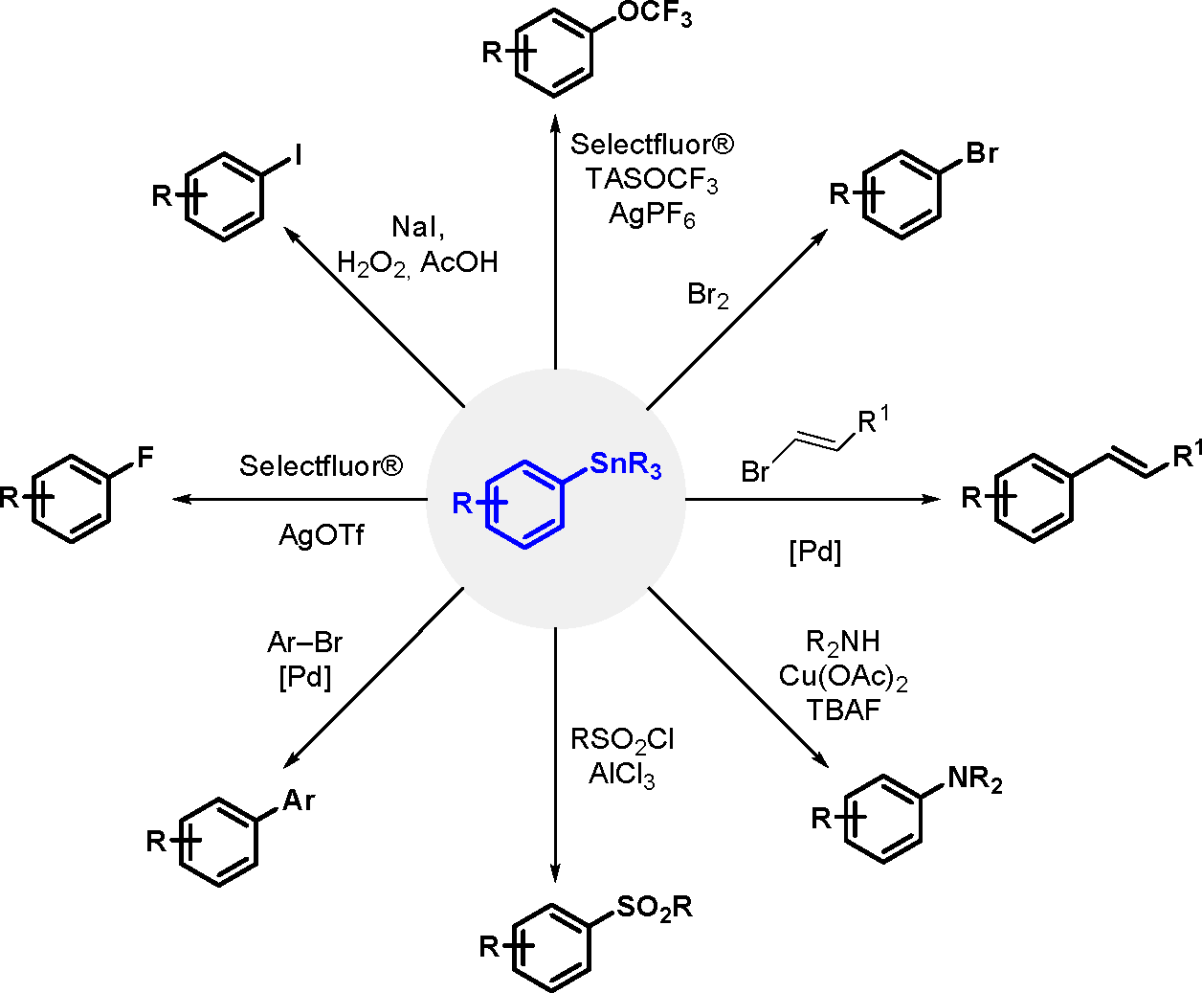
Synthetic Application of Arylstannanes

Arylstannanes that are readily synthesized using traditional methods with lithium or magnesium-based organometallic reagents or via TM-catalyzed stannylation of corresponding aryl electrophiles with distannanes or tertiary stannanes (HSnR_3_) have gained importance because of their application in Migita–Kosugi–Stille cross-coupling reactions with aryl and alkenyl halides.^603−606^ They are also frequently used in the construction of diverse carbon–heteroatom bonds, such as C–X, C–N, C–S, and C–O bonds (Scheme 260).

**Scheme 261:**
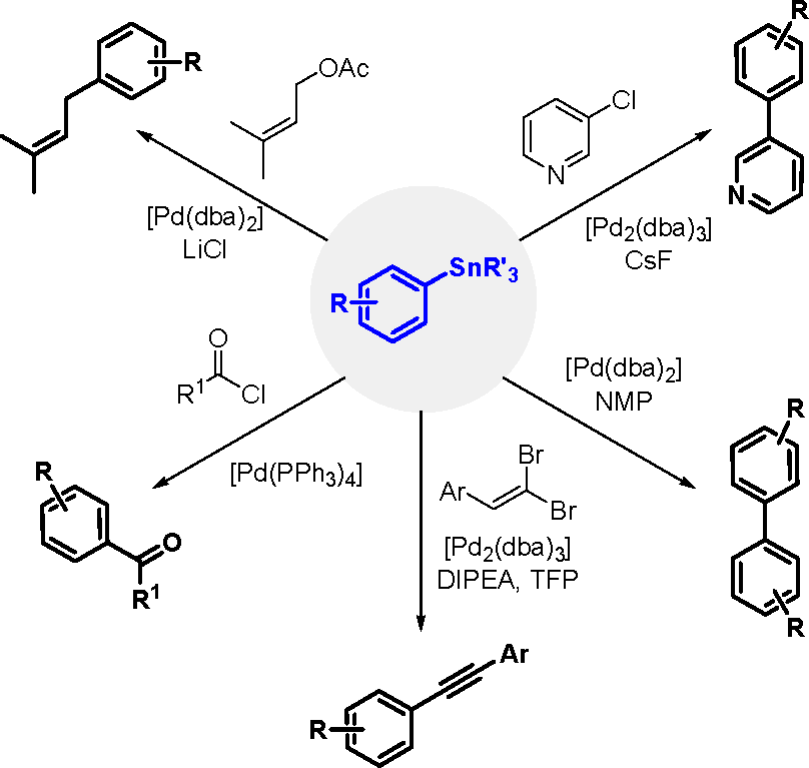
Application of Arylstannanes in Cross-coupling Reactions

Although other alternative Pd-catalyzed cross-coupling reactions have been rapidly developed, Migita–Kosugi–Stille coupling is still an extremely useful and competitive tool in organic synthesis. The synthetic process involves the coupling of organic electrophiles (halides, acyl chlorides, and pseudohalides, such as triflates, sulfonates, and phosphates) with organostannanes in the presence of catalytic amounts of Pd(0) or Pd(II) precatalysts (Scheme 261). The unique features of this cross-coupling reaction are the use of organostannanes (relatively insensitive to moisture and oxygen) and a diversity of organic electrophiles, mild reaction conditions (no requirement for a base), and tolerance to a wide variety of functional groups, which enables synthesis of complex heterocyclic molecules.

Migita–Kosugi–Stille coupling is widely applied for the total synthesis of natural products.^577,578,603,607^ The reaction tolerates air and moisture, and it is compatible with various functional groups.

**Scheme 262:**
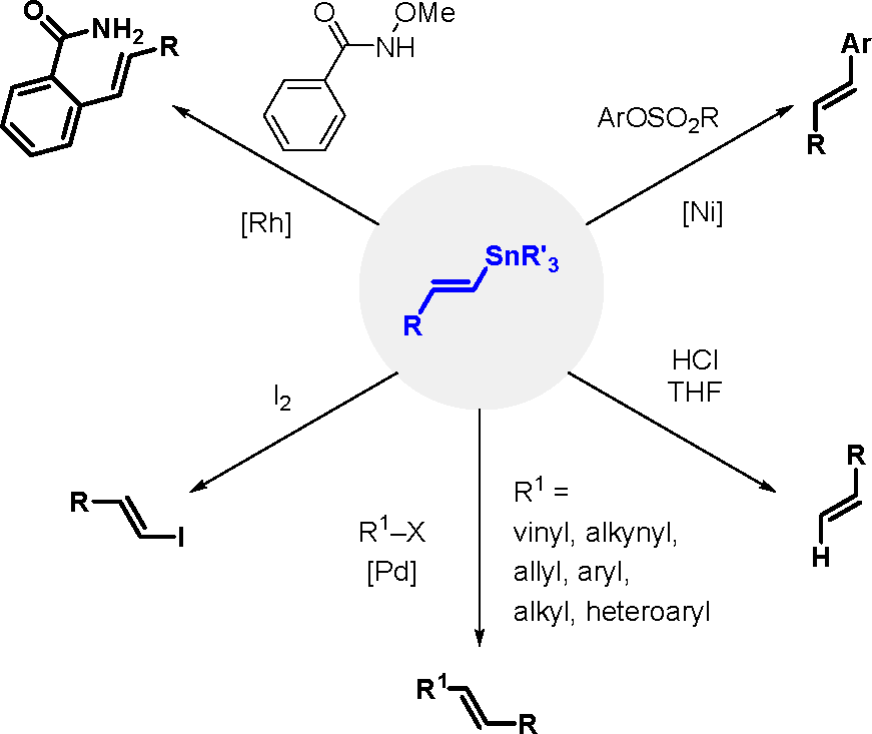
Synthetic Applications of Vinylstannanes

Alkenylstannanes, which can be easily and selectively prepared via hydrostannation of acetylenes or via palladium-catalyzed stereospecific cross-coupling of alkenyl halides or triflates with distannanes, are versatile precursors for disubstituted olefins (Scheme 262). Their most synthetically useful TM-catalyzed transformation is Stille coupling with a variety of organic electrophiles.

**Scheme 263:**
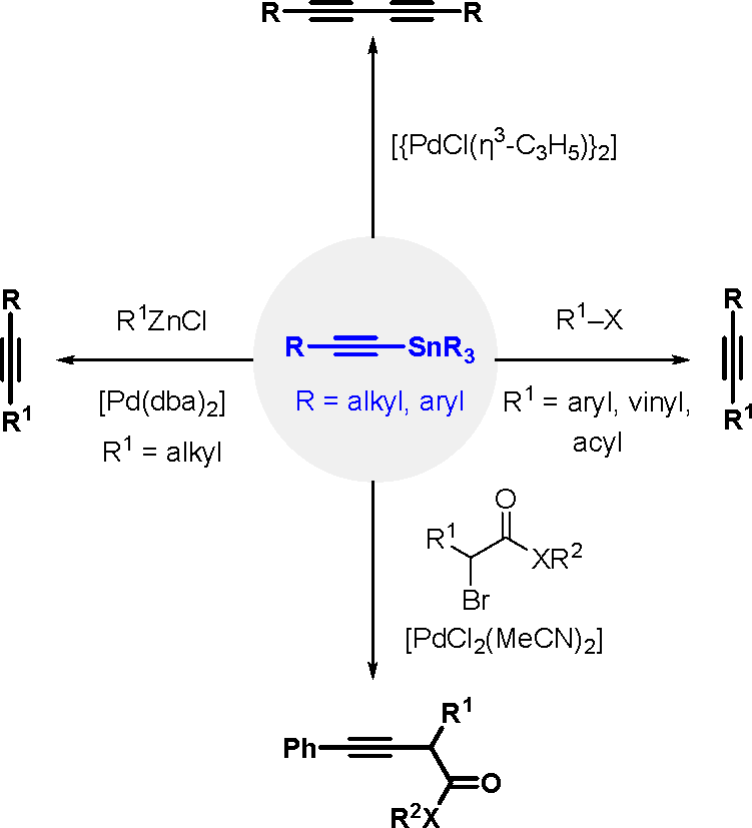
Alkynylstannanes in Organic Synthesis

Alkynylstannanes are formed either directly from terminal alkynes or through their metal derivatives. The activity of alkynylstannanes in cross-coupling is generally lower than that of other alkynylmetals. However, their stability and tolerance to a wide range of functionalities make them convenient coupling agents (Scheme 263).

**Scheme 264:**
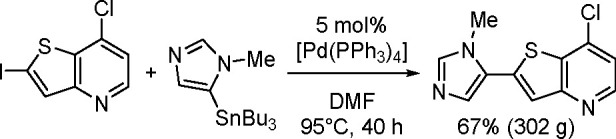
Synthetic Applications of Migita–Kosugi–Stille Coupling in the Synthesis of the VEGFR Kinase Inhibitor

Organostannanes have been found to be good coupling partners in the synthesis of complex heterocyclic systems. As an example, Migita–Kosugi–Stille cross-coupling has been applied in the large-scale preparation of an imidazole–thienopyridine VEGFR kinase inhibitor for pharmaceutical purposes (Scheme 264).

**Scheme 265:**
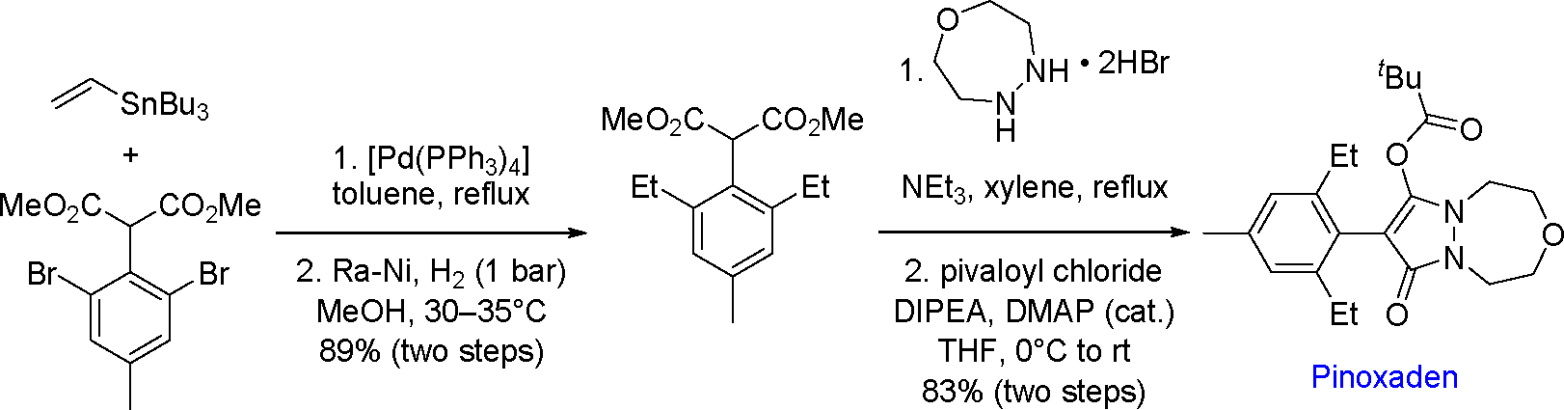
Synthesis of *Pinoxaden*

Likewise, catalytic vinylation of haloarenes is most efficient with vinylstannanes, as illustrated by the *pinoxaden* manufacturing method (Scheme 265). This is a selective postemergence herbicide for the control of annual grass weeds in cereal crops. However, the use of tin reagents in other industrial scale carbon–carbon bond formation reactions has been hampered by residue toxicity and purification costs.

**Scheme 266:**
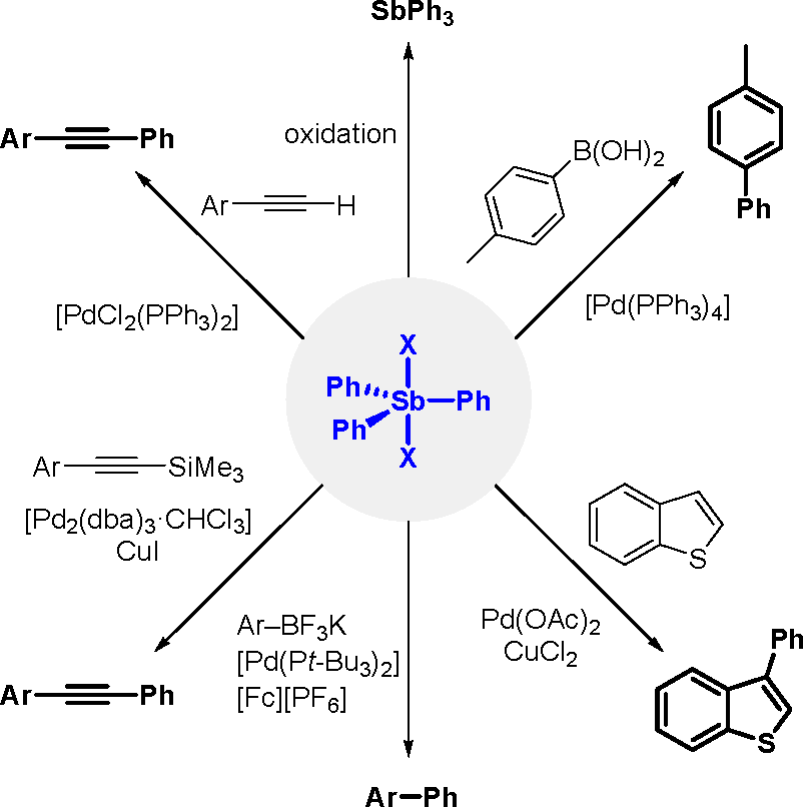
Triarylantimony Dicarboxylates in Catalytic Organic Synthesis

Early studies on the application of stibanes in catalytic organic synthesis confirmed that these compounds can be used in coupling reactions. The state of the art on the application of organoantimony compounds in organic synthesis, including the achievements up to the early 2000s, has been summarized.^608,609^ Although effective procedures for the catalytic formation of C–C bonds using tellurium compounds have recently been proposed, the related chemistry is still underdeveloped. Organoantimony(III) derivatives need to be oxidized to be active in Pd-catalyzed coupling. Triarylantimony dicarboxylates have been reported to be effective in TM-catalyzed carbon–carbon bond-forming reactions such as Heck, Suzuki, Stille, and Hiyama cross-couplings (Scheme 266).

Remarkable enhancement in reactivity has been observed for the reagents exhibiting intramolecular N → Sb interactions. This phenomenon has been used in the design of convenient antimony-based coupling reagents, which are easy to synthesize and readily undergo Suzuki cross-coupling.^610^

**Scheme 267:**
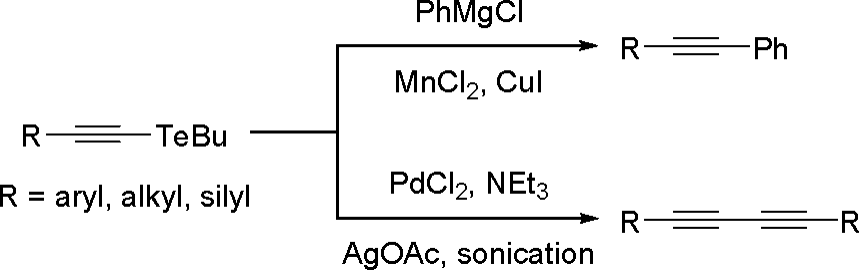
Synthetic Applications of Alkynyltellurides

The TM-catalyzed transformation of organotellurium compounds has remained largely unexplored. However, some progress in the application of tellurium has been observed in the past 20 years because these species have been identified as alternatives to halogens as electrophilic partners in palladium-catalyzed cross-coupling reactions. The application of organotellurium compounds in organic synthesis has been summarized in several reviews.^611−614^ Alkynyltellurides readily undergo ultrasound-assisted homocoupling in the presence of a palladium catalyst or may undergo Kumada coupling with a Grignard reagent in the presence of manganese(II) chloride and copper(I) iodide as catalysts (Scheme 267).

**Scheme 268:**
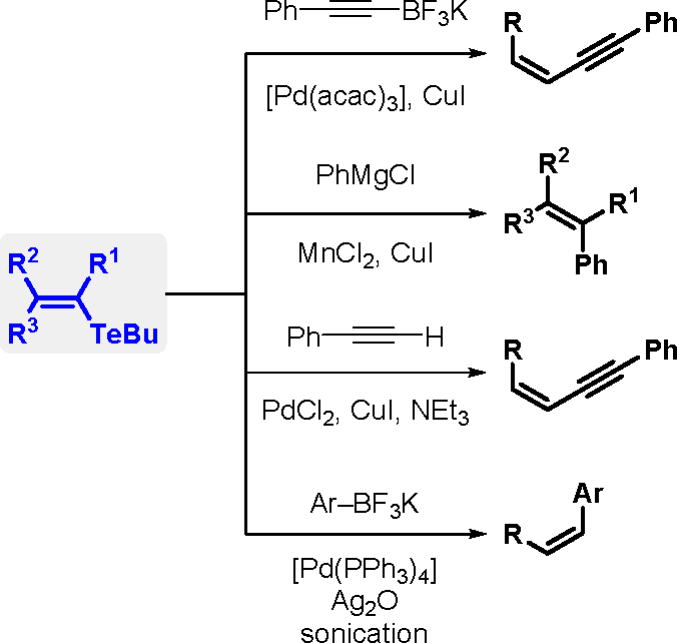
Applications of Vinyltellurides in Cross-coupling

Vinyltellurium derivatives undergo several palladium-catalyzed cross-coupling reactions (Scheme 268).

### Perspectives of Inorganometallic Catalysis

It can be concluded on the basis of this Review that inorganometallic compounds containing direct metal–metalloid bonds play a crucial role in catalysis. We considered only the compounds containing bonds between metals, mainly TMs, and seven other elements, typically metalloids (E) (E = B, Si, Ge, Sn, As, Sb, Te) in our Review. In terms of catalysis, all these bonds are important and are often found in intermediates that are essential parts of the catalytic cycles of many processes that furnish new or well-known organometalloid compounds. In most cases, they are derivatives that contain C–E but also O–E or N–E bonds. Through catalytic transformations involving such derivatives, a process can have certain regio-, chemo-, and stereoselectivity to produce compounds not available by other synthetic methods.

Analysis of the recent literature reports shows some specific trends in the catalytic processes that use inorganometallic compounds. First of all, interest in the first row TMs, in particular earth-abundant metals, has grown significantly. This is of course associated with much higher abundance, lower toxicity and, especially, price that is lower than that of the platinum group metals, which have long dominated this type of catalytic processes. The use of these TMs to form bonds with metalloids as effective catalysts has increased the interest in multidentate, and especially tridentate, ligands. These tridentate ligands in particular enable the formation of pincer complexes, which are highly active, selective, and, above all, stable.

However, the processes involving complexes that contain a double bond between a TM and a metalloid are also gaining importance. The associations of this type have not been addressed in our review, but there are many interesting articles on this topic.^30^ In particular, numerous studies by the Tilley group (for example, see ref (615)) on metal–silylene complexes and the studies by Braunschweig^616,617^ and Ghosh^618^ groups on metal–borylene complexes are extremely interesting and provide plenty of valuable information.

Despite the extremely rapid evolution of this field, there are still a few areas that will surely be developed in the near future. Hydrofunctionalization of alkenes and alkynes catalyzed by earth-abundant metal complexes with tridentate ligands has not yet found any large-scale industrial application. The formation of low-oxidation metal species is necessary to activate hydrogen-heteroatom bonds, but it is very sensitive to oxygen and moisture, which is a major obstacle to their industrial application. Consequently, to scale up the process for industrial applications, the issue of sensitivity must be addressed without compromising their catalytic performance. The development of highly efficient and easy-to-handle catalysts that can be used in the large-scale synthesis of commercially important products is certainly an investigation area for the near future. On the other hand, transition metal-free catalysts for alkyne hydroelementation using Si, B, Sn, and Ge hydrides, intensively reported over the past decade, seem to be an interesting alternative to the conventional catalytic regimes based on transition metal complexes.^619^

Some reviews emphasize the need for TM-complexes immobilized on inorganic or polymeric supports^18,19^ as recyclable heterogeneous catalysts. Although heterogeneous catalysts are not discussed in the review, their connections with well-defined surface-immobilized TM complexes illustrate the general strategy for this type of heterogeneous catalysis. The reported examples, particularly in the catalysis of hydrosilylation^50−52^ as well as Beller’s concept of SACs as materials with isolated TM centers stabilized by neighboring salts,^19,51^ have been convincing arguments for the initiation of extensive studies which can be applied in academia and industry.

In view of the known activity of metal nanoparticles in catalytic processes^620,621^ in some reactions described in this Review, metal clusters and/or metal nanoparticles can be considered as active intermediates. The activity of stabilized or immobilized metal nanoparticles has been well described for the formation of C–B bonds as a result of hydroboration (Ir, Fe), diboration (Au, Pd, Pt) silaboration (Pt) or borylation of C–H (Pd, Ir) and C–X bonds (Cu, Pd) [for review see ref (622, 623).]. Recently, Mandal reported arene C–H borylation with HBpin catalyzed by Ni nanoparticles generated in situ from abnormal *N*-heterocyclic carbene Ni(II) complex.^624^ Bose revealed catalytic activity of presynthesized copper nanoparticles in hydroboration of alkenes and β-borylation of α, β-enones with B_2_pin_2_^625^ as well as borylation of alkyl chlorides and bromides.^626^ On the other hand, Kleeberg’s works show that copper boron complexes recognized as key intermediates in copper catalyzed borylation may decompose under catalytic reaction conditions to form low valent copper clusters,^627^ which as sources of boron nucleophiles, have to be considered as potential reactive intermediates in borylation reactions.^628^ In the future research, this aspect of catalysis with transition metal complexes should be taken into greater consideration.

TM-catalyzed bis-metalations of C–C multiple bonds have received considerable attention during the past two decades because they provide a convenient and direct method for regio- and diastereodivergent as well as regio- and stereoselective access to difunctional building blocks. Significant progress in this area has been made by the application of new effective catalysts, deeper understanding of the reaction mechanisms, and introduction of selective chiral ligands so that bis-metalation chemistry can be applied in enantioselective synthesis. The developments over the past decade have made it possible to employ simple copper catalysts instead of the traditionally used platinum and palladium complexes and revealed many new variants of bis-metalation processes (also enantioselective) based on the three-component coupling of alkynes or alkenes with bis-metalloids and metalloid alkoxides. Special attention has been paid to the development of bis-metalations with heterodimetalloid compounds, among which silaboration plays a particularly important role as the resulting products can undergo chemoselective cross-coupling reactions with a high regiocontrol level to give biologically and pharmaceutically active molecules.

Arene C–H borylation in the presence of the most active catalysts proceeds effectively under mild conditions (rt) with a 1:1 ratio of the boron unit and arene, with functional group tolerance. The reaction offers a convenient method for arylboronic ester synthesis. Recent achievements from the Hartwig laboratory demonstrate selective procedures for the undirected borylation of primary C–H bonds (or secondary bonds if the primary C–H bonds are absent or blocked).^567^ The protocol enables the introduction of a range of functional groups at the strongest alkyl C–H bonds in organic molecules. More research will certainly be carried out on this system and other methods for the borylation of Csp^3^–H bonds. A further increase in the importance of boron compounds should be anticipated.

Silylation of arene C–H bonds is much less developed than the borylation. Catalysts have been developed for reactions with an arene as a limiting reagent. Further progress should be made for finding new more active catalysts for the process. Undirected intermolecular silylation of unactivated Csp^3^–H bonds is currently in the initial stage of research. The reaction, including the challenging methane silylation, is expected to be the subject of intensive research in the near future.

Methods for the borylation of C–X bonds (X = halogen, sulfonate) are well developed and represent an attractive alternative for the formation of metal–metalloid bonds. For example, they enable the synthesis of C–As or C–Te bonds. Progress in research on processes involving the activation of C–F bonds can be expected. Research on the functionalization of C–C and C–Het bonds will continue.

The asymmetric synthesis of secondary and tertiary boronic esters remains an area with great potential for the development of new methodologies. The versatility of alkylboronic esters and their importance in the synthesis of complex molecules mean that the challenge of introducing a carbon–boron bond in a stereodefined and site-selective manner will continue to be of great interest to many research groups. Enantioselective intermolecular borylation of Csp^2^–H and Csp^3^–H bonds is still underdeveloped. As it remains an area with great potential for the development of new methodologies, steady progress in this field can be expected.

The dehydrocoupling of hydrometalloids remains the most benign and atom-economical process for producing macromolecular compounds with metalloid–metalloid bonds with interesting electronic and optical properties. According to the general trend observed in inorganometallic catalysis, traditionally used d^0^ metallocene complexes and noble metal complexes are slowly being replaced by earth-abundant metal catalysts (e.g., Cr, Fe, and Ni complexes). However, the reported catalytic systems often suffer from catalyst sensitivity toward moisture and oxygen, complexity, and the high cost of the ligand used. High expectations are also related to the development of modern homo- and heterodehydrocoupling reactions leading to molecular bis-metalloids (e.g., diboranes or silylboranes), which are commonly used as reagents in bis-metalation and C–H metalation processes.

Metalloid vinyl derivatives react with presynthesized transition metal hydride complexes, which leads to a nearly quantitative formation of TM–E (E = B, Si, Ge) and ethylene bonding. They can be used as convenient sources of metalloids. Therefore, they can participate in reactions in which TM–H bonds are formed (e.g., as a result of the oxidative addition of CH, EH, or Het–H bonds to Ru, Ni transition metals), which ultimately leads to the formation of C–E, E–E, or Het–E bonds. The reaction potential has not been fully exploited, and further research is to be expected.

All the previously discussed catalytic processes show the importance of the TM–E bond, which was generally generated *in situ*. Therefore, a recent challenging problem is to prepare TM–E bond-containing complexes that can later be used as catalysts. In particular, it will be important to select a ligand for the metal and use it to activate nonreactive chemical bonds and molecules based on rational design of the ligand structure. This topic has been very well presented in Takaya’s review article.^16^

An important challenge for the future is to implement new and synthetically valuable molecular transformations that are not easy to achieve with standard transition metal catalysts, using the characteristic reactivity of TM–E complexes from the point of view of synthetic chemistry.

The best argument for the essentially different role of organometallic (TM–C) and inorganometallic (TM–E) complexes in catalysis is provided in a very recent review by the Whited and Taylor group,^30^ and it focuses on considerations for catalysis and similarities and differences between TM–C and TM–Si chemistry. The latter is the best representative of inorganometallics (TM–E complexes).^30^ Most reactions of TM–Si complexes can be predicted on the basis of several principles important in catalytic systems, such as electronegativity that is lower than that of carbon (and also of hydrogen and many TMs). However, the relatively large size of the silicon atom enables the formation of a variety of bonds at the metalloid (e.g., secondary interactions with hydride and other X-type ligands). Finally, the above features are quite powerful and allow silicon to behave in many instances more as a second TM than as an organometallic ligand.

**Scheme 269:**
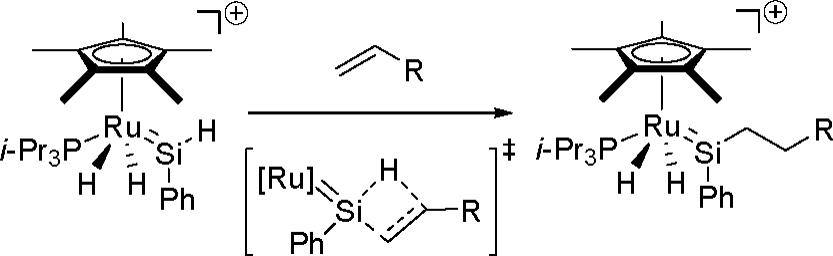
Insertion of Unsaturated Substrates into Si–X Bonds of Silylenes

**Scheme 270:**
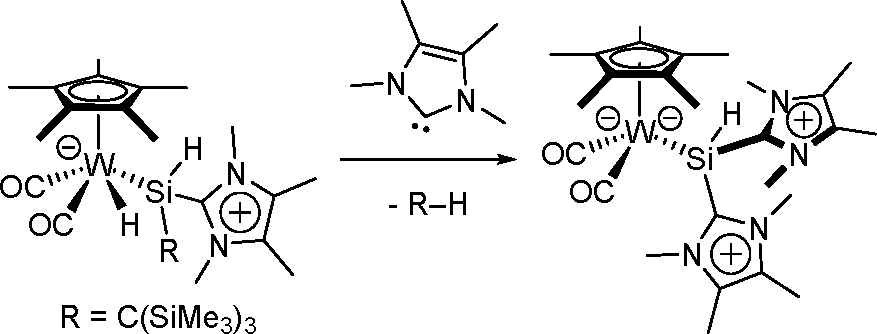
NHC-Promoted 1,2-Alkane Elimination from a W–Si Complex. Adapted from Ref (629). Copyright 2015 American Chemical Society

The concerted insertions of alkenes into Si–H bonds (in the Glaser–Tilley mechanism of hydrosilylation (Scheme 269) and the breaking of strong silicon–carbon bonds with 1,2-alkane elimination (Scheme 270)^629^ provide convincing evidence that the TM–Si complex may behave like a bimetallic system to develop cooperative catalysis, which has been demonstrated primarily for TM–boron systems.^30^

The examples show that the reaction pathways of TM and silicon share some features (e.g., flexible coordination environment and kinetically labile bonds) and can therefore be quite complementary.

As well as silicon compounds, boron compounds play the most important role among organometalloids. Direct borylation is a powerful synthetic methodology to generate organo-boron compounds. However, there are also many areas for improvement and further development on this topic. One of these is the limitation of the substrates. Increasing efforts have been made to use sustainable aliphatic feedstocks for syntheses of alkylboronates. Moreover, the borylation of simple raw materials using nonactivated OH, SH, NH_2_ and COOH as the leaving groups is still a huge challenge due to the kinetic and thermodynamic stability of these chemical bonds. The functionalization of internal alkenes without isomerization also requires further improvement. In Markovnikov hydroboration, the regioselectivity control of aliphatic alkenes is far from ideal. Outstanding regioselectivity control in the hydroboration of unsymmetrical 1,2-disubstituted alkenes remains a challenge. Overcoming this will likely require more modular ligand platforms for the accelerated evaluation of metal–ligand combinations to find a more preferred precatalyst system. The versatility of alkylboronic esters and their resulting importance in the synthesis of complex molecules means that the challenge of introducing a carbon–boron bond in a stereodefined and site-selective manner will continue to be of great interest to many research groups. Certainly, research will be carried out on the further improvement of selectivity in the late stage of modification of complex functional molecules as well as on the development of asymmetric transformations.

Catalytic hydrometalation processes–and above all, C–H bond activation reactions with hydro- and bis-metalloids, which have been intensively developed in recent years–have revolutionized modern synthetic organic chemistry ensuring straightforward access to a wide variety of organometalloids. Boronate esters, organosilanes, and organostannanes represent the most popular organometalloid nucleophiles that offer beneficial robustness and reactivity in cross-coupling reactions, and these have been applied in numerous sequential (tandem) processes of alkene, alkyne, and arene functionalization. The experimental advances in the reactivity of new organometalloid reagents (e.g., organogermanium and organoantimony derivatives) in C–C- and C–X-bond-forming transformations as well as the application of novel catalysts (based on gold but also coinage metal complexes) open up new routes for the intensive development of cross-coupling methods. The growing needs of organic synthesis will certainly stimulate the development of further synthesis methods of organometalloids, for which inorganometallic catalysis is an invaluable tool.

From the catalytic point of view, all transformations involving the TM–E bond are important, but when it comes to the production of materials, the silicon derivatives are of the greatest importance, as all other derivatives are in most cases raw materials for the production of new organometalloid compounds. Formation of the TM–Si bond is a key step in the catalytic process of hydrosilylation. It is concluded that hydrosilylation processes are commonly used in the production of silane coupling agents, in the cross-linking of silicones, and in the production of hybrid and preceramic materials. They are also used in the synthesis of nanoparticles with a strictly defined spatial structure (silsesquioxanes and dendrimers) and in the production of nanocomposites containing nanoparticles.^630^ The methods for synthesizing polysilanes (crucial raw materials for the production of sensors, LEDs, photovoltaic devices, and nonlinear optics) are also continuously developed, using new catalytic systems. Silicone materials and production technologies have steadily improved since the turn of the 21st century, with the majority of advances driven by new application needs, which have been met primarily by new products developed by established silicone manufacturers or specialty formulators.

Boron compounds also play an increasingly important role in applications such as small-molecule activation for fuel synthesis, organic light-emitting diodes (OLEDs), hydrogen production and storage, and electrolyte materials.^630−632^ The widespread use of boron-containing compounds is closely related to the unique properties of the element itself. Because of the electron deficiency of boron, its compounds may act as electrophiles under certain conditions, while under other conditions, they will act as nucleophiles. As a result, boron can form a variety of compounds whose properties can be tailored to specific applications.

It can be concluded on the basis of this Review that inorganometallic compounds containing direct transition metal–metalloid bonds play a crucial role in catalysis as well as in the synthesis and functionalization of many organic and inorganic derivatives. Such catalytic transformations produce compounds that cannot be obtained by other methods or permit running a process with certain regio-, chemo-, and stereoselectivity. The deep insight into the catalytic activity of TM–Si and TM–B complexes can be extended to other TM–E complexes, particularly when E is Ge or Sn, and a variety of supporting coligands at the TM complex. Based on the comprehensive information collected here, new catalysts of the known reactions could be designed and, at the same time, novel strategies for the development of new processes will be proposed.
